# Copper-catalyzed aerobic aliphatic C–H oxygenation with hydroperoxides

**DOI:** 10.3762/bjoc.9.138

**Published:** 2013-06-25

**Authors:** Pei Chui Too, Ya Lin Tnay, Shunsuke Chiba

**Affiliations:** 1 Division of Chemistry and Biological Chemistry, School of Physical and Mathematical Sciences, Nanyang Technological University, Singapore 637371, Singapore. Fax: +65-67911961; Tel: +65-65138013

**Keywords:** copper, 1,4-diols, free radical, 1,5-H radical shift, hydroperoxides, molecular oxygen

## Abstract

We report herein Cu-catalyzed aerobic oxygenation of aliphatic C–H bonds with hydroperoxides, which proceeds by 1,5-H radical shift of putative oxygen-centered radicals (O-radicals) derived from hydroperoxides followed by trapping of the resulting carbon-centered radicals with molecular oxygen.

## Introduction

**Scheme 1:**
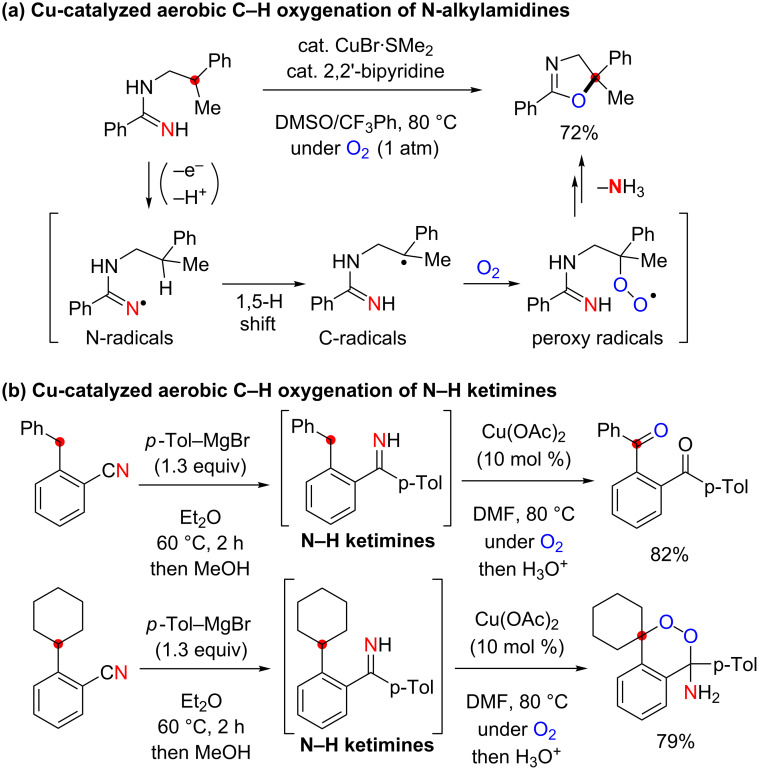
Aliphatic C–H oxidation with amidines and ketimines by 1,5-H radical shift.

Aliphatic sp^3^ C–H bonds are ubiquitous components in organic molecules but rather inert towards most of the chemical reactions. It thus remains as one of the most challenging topics in organic synthesis to develop catalytic oxidative sp^3^ C–H functionalization with predictable chemo- and regioselectivity [1–4]. To achieve this goal, we have recently utilized 1,5-H-radical shift [5–6] with iminyl radical species (N-radicals) generated under Cu-catalyzed aerobic reaction conditions, in which the resulting carbon-centered radicals (C-radicals) could be trapped by molecular oxygen to form new C–O bonds. For instance, the Cu-catalyzed aerobic reaction of *N*-alkylamidines afforded aminidyl radicals (N-radicals) by single-electron oxidation and deprotonation of the amidine moiety, which was followed by 1,5-H-radical shift to generate the corresponding C-radicals (Scheme 1a) [7]. The successive trapping of the resulting C-radicals with molecular O_2_ forms peroxy radicals (the C–O bond formation). Reduction of peroxy radicals generates alkoxides, cyclization of which with the amidine moiety finally affords dihydrooxazoles. Similarly, it was also revealed that the sp^3^ C–H oxygenation could proceed directed by the N–H ketimine moieties under Cu-catalyzed aerobic conditions via the corresponding iminyl radical species, where 1,2-diacylbenzenes and amino endoperoxides could be synthesized by C–H oxygenation of secondary and tertiary C–H bonds, respectively (Scheme 1b) [8–9].

**Scheme 2:**
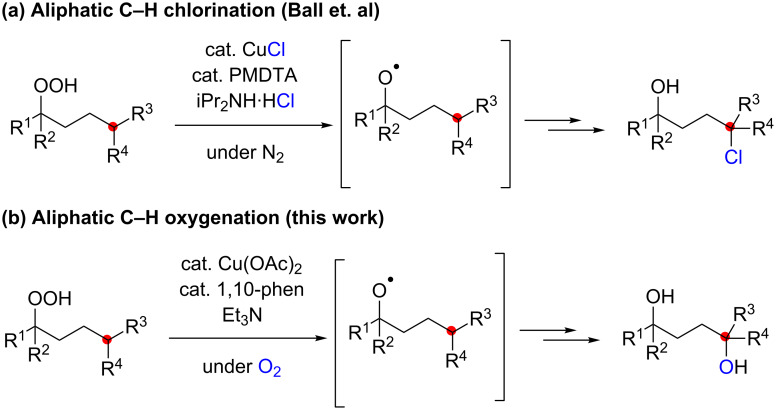
Aliphatic C–H oxidation with hydroperoxides.

Stimulated by these remote sp^3^ C–H oxidation reactions using the nitrogen-centered radicals derived from amidines and ketimines, we became interested to utilize oxygen-centered radicals (O-radicals) for the sp^3^ C–H functionalization. In this context, we envision employing hydroperoxides as precursors for O-radicals in the presence of Cu salts. The lower valent Cu(I) species could potentially undergo single-electron reduction of hydroperoxides to produce the corresponding O-radicals [10–13]. Ball recently reported CuCl-catalyzed aliphatic C–H chlorination using hydroperoxides as the O-radical source, in which the C-radicals generated by the 1,5-H radical shift were chlorinated (Scheme 2a) [14]. If reductive generation of the O-radicals from hydroperoxides could be achieved under an O_2_ atmosphere, the C-radicals generated by 1,5-H radical shift could be trapped by O_2_ to form the new C–O bonds. Herein, we report the realization of this concept mainly for the aerobic synthesis of 1,4-diols from hydroperoxides, which could be catalyzed by the Cu(OAc)_2_-1,10-phenanthroline system in the presence of Et_3_N as a terminal reductant of the Cu(II) species (Scheme 2b).

## Findings

We commenced our investigation with the Cu-catalyzed aerobic reactions of hydroperoxide **1a** in the presence of Et_3_N as a terminal reductant [15] to keep lower valent Cu(I) species in the reaction mixture (Table 1). As expected, when **1a** was treated with Cu(OAc)_2_ (20 mol %) in the presence of Et_3_N (2 equiv) in DMF, the reaction proceeded at room temperature for 17 h to afford C–H oxygenation products, cyclic hemiacetal **2a** and 1,4-diol **3a** in 49% and 8% yields, respectively (Table 1, entry 1). It was found that addition of nitrogen ligands such as 2,2’-bipyridine and 1,10-phenanthroline (1,10-phen) accelerated the reaction (Table 1, entries 2 and 3). The reactions with CuCl_2_ (Table 1, entry 4) as well as CuCl (Table 1, entry 5) resulted in comparable results with that by Cu(OAc)_2_. Further optimization of the reaction conditions by the solvent screening (Table 1, entries 6–11) revealed that the co-solvent system (benzene/MeCN or toluene/MeCN) performed best to give the highest yield (83–87% combined yields of **2a** and **3a**, Table 1, entries 9–11), in which the amount of Et_3_N could be reduced to 0.5 equiv (Table 1, entries 10 and 11). It is worthy of note that the catalytic loading of Cu(OAc)_2_-1,10-phen could be lowered to 5 mol % while maintaining good combined yields of **2a** and **3a** (89%, Table 1, entry 13). Upon completion of the C–H oxygenation under the reaction conditions in entry 13, the resulting crude residue after removal of the solvents was treated with LiAlH_4_ in THF, affording 1,4-diol **3a** as the sole product in 90% yield (Table 1, entry 14). The Cu-catalyzed reaction of **1a** under an inert (N_2_) atmosphere gave an intramolecular C–H oxygenation product, dihydroisobenzofuran **4a** in 47% yield (Table 1, entry 15).

**Table 1:** Optimization of the reaction conditions.^a^

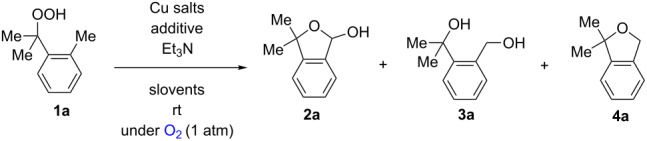								

Entry	Cu salts (mol %)	Additive (mol %)	Et_3_N (equiv)	Solvent (0.1 M)	Time (h)	Yield (%)^b^		

						**2a**	**3a**	**4a**

1	Cu(OAc)_2_ (20)	–	2.0	DMF	17	49	8	0
2	Cu(OAc)_2_ (20)	2,2’-bipyridine (20)	2.0	DMF	3	49	9	0
3	Cu(OAc)_2_ (20)	1,10-phen (20)	2.0	DMF	3	53	12	0
4	CuCl_2_ (20)	1,10-phen (20)	2.0	DMF	6	52	10	0
5	CuCl (20)	1,10-phen (20)	2.0	DMF	3	52	10	0
6	Cu(OAc)_2_ (20)	1,10-phen (20)	2.0	MeCN	2	55	20	0
7	Cu(OAc)_2_ (20)	1,10-phen (20)	2.0	benzene	5	71	13	0
8	Cu(OAc)_2_ (20)	1,10-phen (20)	2.0	toluene	9	70	14	0
9	Cu(OAc)_2_ (20)	1,10-phen (20)	2.0	benzene/MeCN (5:1)	2	76	11	0
10	Cu(OAc)_2_ (20)	1,10-phen (20)	0.5	benzene/MeCN (5:1)	3	74	9	0
11	Cu(OAc)_2_ (20)	1,10-phen (20)	0.5	toluene/MeCN (5:1)	3	74	10	0
12	Cu(OAc)_2_ (10)	1,10-phen (10)	0.5	toluene/MeCN (5:1)	3	75	16	0
13	Cu(OAc)_2_ (5)	1,10-phen (5)	0.5	toluene/MeCN (5:1)	3	75	14	0
14^c^	Cu(OAc)_2_ (5)	1,10-phen (5)	0.5	toluene/MeCN (5:1)	3	–	90	0
15^d^	Cu(OAc)_2_ (5)	1,10-phen (5)	0.5	toluene/MeCN (5:1)	1	0	0	47

^a^Reactions were carried out using 0.3 mmol of hydroperoxide **1a** in solvents (3 mL, 0.1 M) at room temperature under an O_2_ atmosphere. ^b^Isolated yields are recorded. ^c^After stirring 5 h, the volatile materials were removed in vacuo, and the resulting crude materials were further treated with LiAlH_4_ (1.2 equiv) in THF at rt for 1 h. ^d^The reaction was conducted under a N_2_ atmosphere.

**Scheme 3:**
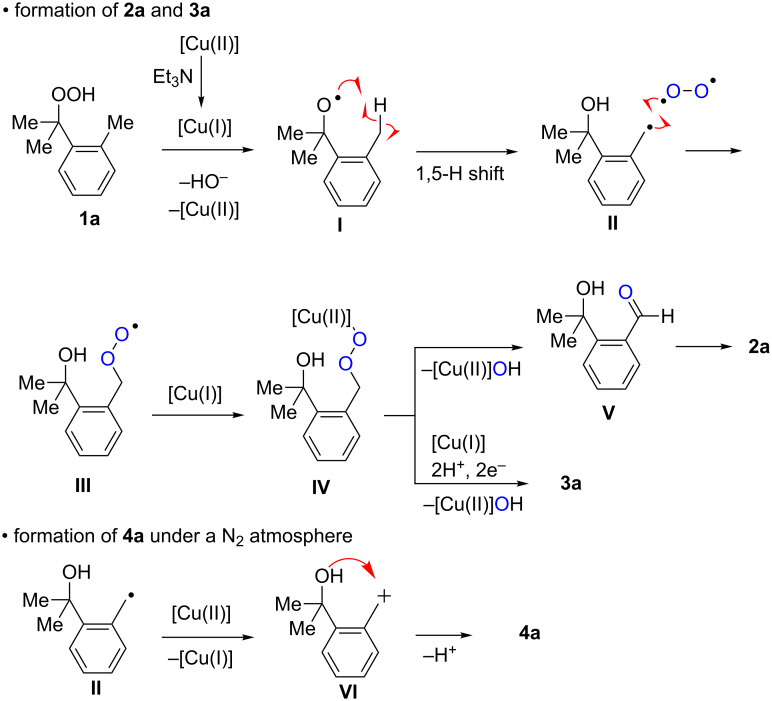
Proposed reaction mechanisms for the formation of **2a**, **3a**, and **4a**.

The proposed reaction pathways for the formation of **2a**, **3a**, and **4a** were described in Scheme 3. Single-electron reduction of the starting Cu(OAc)_2_ by Et_3_N forms the Cu(I) species, which reduces hydroperoxide **1a** to give O-radical **I** along with the generation of the Cu(II) species. 1,5-H-Radical shift of O-radical **I** generates C-radical **II**, which is trapped by molecular O_2_ to give peroxy radical **III**. Probably further reaction of **III** with Cu(I) species gives Cu(II)-peroxide **IV**, which undergoes fragmentation to give aldehyde **V** [16–18], which in turn cyclizes to afford hemiacetal **2a**. Protonation of Cu(II)-peroxide **IV** followed by the reduction of the resulting hydroperoxide deliver 1,4-diol **3a**. In the absence of molecular O_2_ (under a N_2_ atmosphere, Table 1, entry 15), the resulting C-radical **I** is oxidized by the Cu(II) species to give carbocation **VI** [19], which is trapped by the intramolecular hydroxy group to give **4a**.

**Scheme 4:**
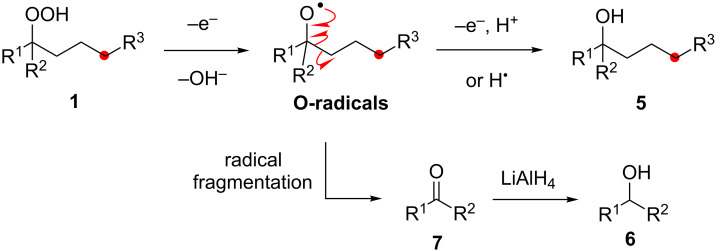
Proposed reaction mechanisms for the formation of **5** and **6**.

We next explored the substrate scope of the present aerobic strategy for the synthesis of 1,4-diols by targeting methylene C–H oxygenation with various tertiary hydroperoxides **1** (Table 2). Generally, oxygenation of benzylic methylene C–H bonds proceeded smoothly to give the corresponding 1,4-diols **3** in good to moderate yields (77–51% yields) (Table 2, entries 1–7). Moreover, the present method allowed for oxygenation of nonbenzylic methylene C–H bonds, while yields of obtained 1,4-diols **3** were relatively low (65–40% yields) (Table 2, entries 8–13). In most cases (except for Table 2, entries 3, 6, 7 and 11), either reduced alcohols **5** (up to 23% yield) or fragmented alcohols **6** (up to 23% yield), or both were observed as minor products. The formation of alcohols **5** occurs by two-electron reduction of hydroperoxides **1** followed by protonation or by H-radical abstraction of the transient O-radical generated by one-electron reduction of hydroperoxides **1** prior to the 1,5-H radical shift (Scheme 4). Presumably, radical fragmentation of O-radicals occurs to give the corresponding ketones **7**, which are reduced by LiAlH_4_ in the next step to give alcohols **6** (Scheme 4).

**Table 2:** Substrate scope: oxygenation of secondary C–H bonds.^a^

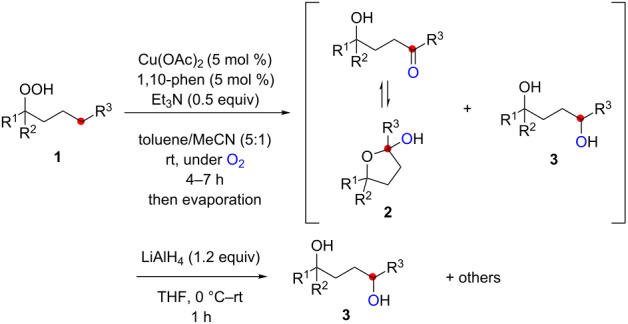				

Entry	Hydroperoxides **1**	1,4-Diols **3**	Others	

			Alcohols **5**	Alcohols **6**

	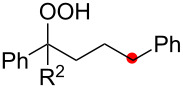	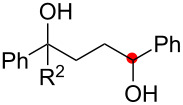		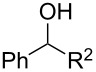
1	**1b** (R^2^ = Ph)	**3b** 77%	–	**6b** 8%^b^
2	**1c** (R^2^ = Me)	**3c** 72% (1:1)	–	**6c** 8%^b^
3	**1d** (R^2^ = Et)	**3d** 51% (1:1)	–	–
	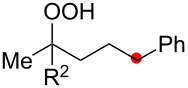	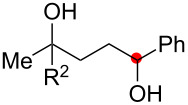	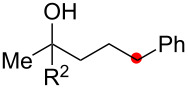	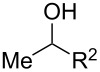
4	**1e** (R^2^ = 2-naphthyl)	**3e** 62% (1:1)	**5e** 9%	**6e** 17%^b^
5	**1f** (R^2^ = 4-Br-C_6_H_4_)	**3f** 70% (1:1)	**5f** 15%	**6f** 15%^b^
6	**1g** (R^2^ = CH_2_CH_2_Ph)	**3g** 51% (1:1)	–	–
7	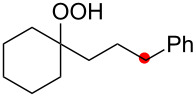 **1h**	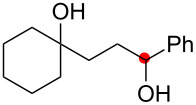 **3h** 56%	–	–
	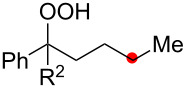	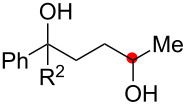	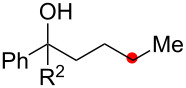	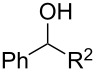
8	**1i** (R^2^ = Ph)	**3i** 65%	**5i** 18%	**6b** 14%^b^
9	**1j** (R^2^ = Me)	**3j** 62% (1.27:1)	**5j** 23%	**6c** 20%^b^
10	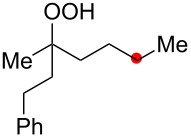 **1k**	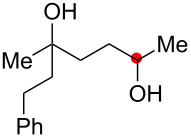 **3k** 40% (1:1)	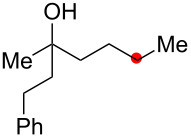 **5k** 16%	–
11	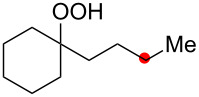 **1l**	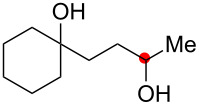 **3l** 48%	–	–
12	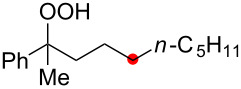 **1m**	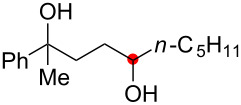 **3m** 45% (1.27:1)	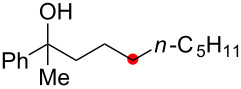 **5m** 18%	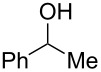 **6c** 20%^b^
13	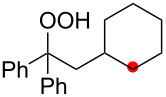 **1n**	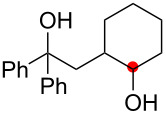 **3n** 51% (*trans*/*cis* = 1.37:1)	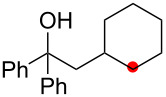 **5n** 13%	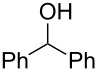 **6b** 23%^b^

^a^Reactions were carried out using 0.5 mmol of hydroperoxides **1** with Cu(OAc)_2_ (5 mol %), 1,10-phen (5 mol %), and Et_3_N (0.5 equiv) in toluene/MeCN (5:1, 0.1 M) at room temperature under an O_2_ atmosphere. After stirring for 4–7 h, the volatile materials were removed in vacuo, and the resulting crude materials were further treated with LiAlH_4_ (1.2 equiv) in THF at rt for 1 h. Isolated yields are recorded. The ratio in parentheses is the diastereomer ratio of the products **3**, where available. ^b 1^H NMR yields with 1,1,2,2-tetrachloroethane as an internal standard.

**Scheme 5:**
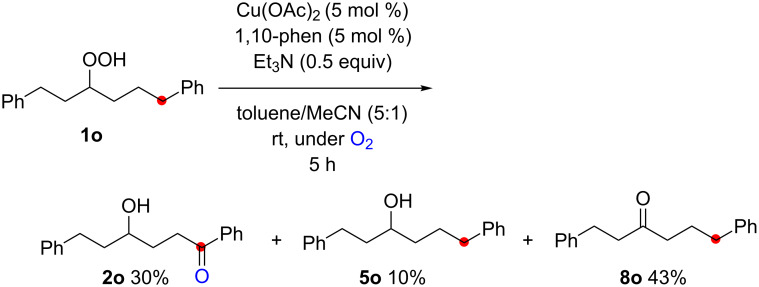
The reaction of secondary hydroperoxide **1o**.

The reaction of a secondary hydroperoxide such as **1o**, however, afforded C–H oxygenation product **2o** (as a keto form) only in 30% yield along with the formation of the corresponding reduced alcohol **5o** and ketone **8o** in 10% and 43% yields, respectively (Scheme 5).

Oxygenation of tertiary C–H bonds was also examined under the present aerobic conditions (Table 3). In these cases, the Cu-catalyzed aerobic reactions afford hydroperoxides **9** as an initial aerobic C–H oxygenation product. After stirring for 5–7 h, the reaction mixtures were successively treated with PPh_3_ (1 equiv) for the reduction of **9** to obtain 1,4-diols **3**. Although the desired 1,4-diols **3** were formed with this two-step one-pot procedure, the isolated yields of **3p–3r** were moderate (45–52% yields) (Table 3, entries 1–3). The oxygenation of the adamantyl C–H bond was especially sluggish, giving the desired 1,4-diol **3s** in only 23% yield (Table 3, entry 4). Similarly, in these reactions, the formation of reduced alcohols **5** (for Table 3, entries 1 and 2) and fragmented ketones **7** (for Table 3, entries 1–4) were observed as minor products.

**Table 3:** Substrate scope oxygenation of tertiary C–H bonds.^a^

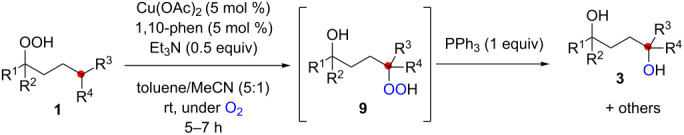				

Entry	Hydroperoxides **1**	1,4-Diols **3**	Others	

			Alcohols **5**	Ketones **7**

	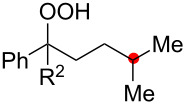	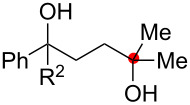	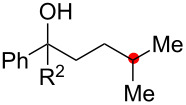	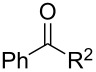
1	**1p** (R^2^ = Ph)	**3p** 52%	**5p** 16%	**7b** 28%^b^
2	**1q** (R^2^ = Me)	**3q** 45%	**5q** 23%	**7c** 24%^b^
3	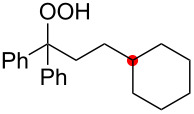 **1r**	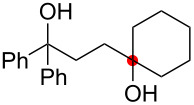 **3r** 47%	–	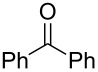 **7b** 12%^b^
4	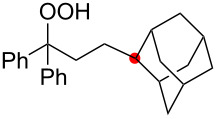 **1s**	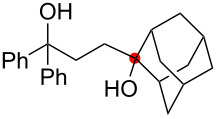 **3s** 23%	–	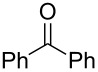 **7b** 12%^b^

^a^Reactions were carried out by using 0.5 mmol of hydroperoxides **1** with Cu(OAc)_2_ (5 mol %), 1,10-phen (5 mol %), and Et_3_N (0.5 equiv) in toluene/MeCN (5:1, 0.1 M) at room temperature under an O_2_ atmosphere. After stirring for 5–7 h, the reaction mixture was further treated with PPh_3_ (1 equiv) at rt. Isolated yields are recorded. ^b 1^H NMR yields with 1,1,2,2-tetrachloroethane as an internal standard.

**Scheme 6:**
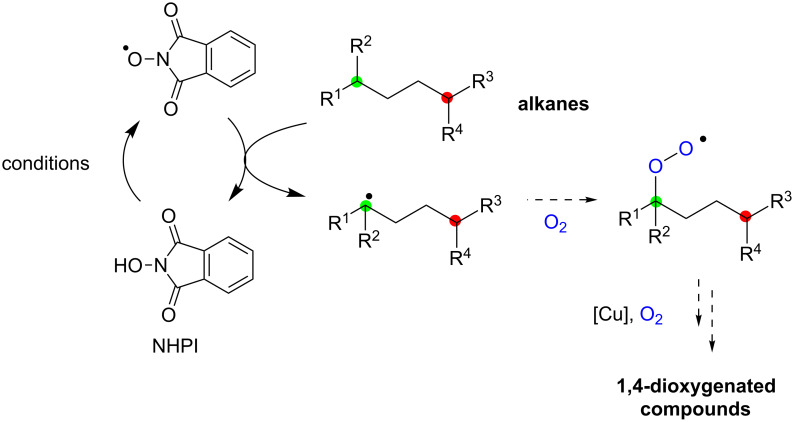
1,4-Dioxygenation of alkanes.

Our next challenge was to develop direct aerobic dioxygenation of alkanes using the present radical strategy. The generation of C-radicals from alkanes at C–H bonds bearing relatively weak bond-dissociation enthalpies (i.e., tertiary alkyl C–H bonds, benzylic C–H bonds, etc.), by phthalimide *N*-oxyl radicals generated oxidatively from *N*-hydroxyphthalimide (NHPI), has been reported [20–22]. The resulting C-radicals could be trapped with molecular oxygen to form hydroperoxides. It could be envisioned that this aerobic C–H bond oxygenation could be combined with the present remote C–H oxygenation with hydroperoxides, presumably resulting in direct formation of 1,4-dioxygenated compounds from nonoxygenated alkanes (Scheme 6).

**Scheme 7:**
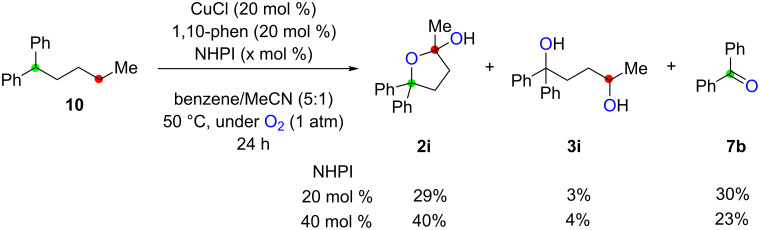
Aerobic 1,4-dioxygenation of alkanes in the CuCl–NHPI catalytic system.

With this hypothesis, alkane **10** bearing a dibenzylic tertiary C–H bond (marked in green) was treated with the catalytic system of CuCl-1,10-phen (20 mol %) with NHPI in benzene/MeCN solvent under an O_2_ atmosphere (1 atm) (Scheme 7). The reaction with 20 mol % of NHPI proceeded as expected at 50 °C to afford a mixture of lactol **2i** and 1,4-diols **3i** in 29% and 3% yields, respectively, via the desired 1,4-dioxygenation, while benzophenone (**7b**) was also formed in 30% yield through fragmentation of the transient alkoxy radical (Scheme 4). Use of 40 mol % of NHPI slightly improved the yields of 1,4-dioxygenation products **2i** and **3i** (40% and 4% yields, respectively).

In conclusion, we have developed the Cu-catalyzed aerobic oxygenation of aliphatic C–H bonds using hydroperoxides by a 1,5-H radical shift of putative O-radicals derived from hydroperoxides followed by trapping of the resulting C-radicals with molecular oxygen. A preliminary result involving the direct 1,4-dioxygenation of alkane **10** was demonstrated by using the present method. Further studies will be carried out to develop more robust and efficient catalytic aerobic radical transformations for polyol synthesis from rather simple alkanes.

## Supporting Information

File 1 Full experimental details and analytical data.
