# Supplementary material for: Copper-catalyzed aerobic aliphatic C–H oxygenation with hydroperoxides
Source: Beilstein J Org Chem. 2013 Jun 25;9:1217–25. doi: 10.3762/bjoc.9.138 (PMC3701382; doi:10.3762/bjoc.9.138)

Supporting Information

for

Copper-catalyzed aerobic aliphatic C–H oxygenation with hydroperoxides

Pei Chui Too, Ya Lin Tnay and Shunsuke Chiba*

Address: Division of Chemistry and Biological Chemistry, School of Physical and Mathematical Sciences, Nanyang Technological University, Singapore 637371, Singapore. Fax: +65-67911961; Tel: +65-65138013

Email: Shunsuke Chiba - shunsuke@ntu.edu.sg

* Corresponding author

Full experimental details and analytical data

1. General S2

2. Synthesis of alkyl hydroperoxide derivatives

- **2.1. Preparation of alkyl alcohols 5a–5s** S2
- **2.2. Preparation of 1-(2-hydroperoxypropan-2-yl)-2-methylbenzene (1a)** S9
- **2.3. Preparation of alkyl peroxides 1b–1n, 1p–1s** S9
- **2.4. Preparation of 1,6-diphenyl-3-hexyl hydroperoxide (1o)** S15
- **2.5. Preparation of 1,1-diphenylpentane (10)** S16

3. Copper-catalyzed aerobic aliphatic C–H oxygenation with hydroperoxides

- **3.1. A typical procedure for the reaction of hydroperoxide 1a (Table 1, entry 14)** S17
- **3.2. A procedure for the reaction of alkane 10 under CuCl-NHPI catalytic system (Scheme 7)** S24

4. ^{1}H and ^{13}C NMR spectrum of new compounds S26

1. General

^{1}H NMR (400MHz) spectra were recorded on a Bruker Avance 400 spectrometer in $CDCl_3$ [using $(CH_3)_4Si$ (for ^{1}H, δ = 0.00) as internal standard], ^{13}C NMR (100 MHz) spectra on a Bruker Avance 400 spectrometer in $CDCl_3$ [using $CDCl_3$ (for ^{13}C, δ = 77.00) as internal standard]. The following abbreviations were used to explain the multiplicities: s = singlet, d = doublet, t = triplet, q = quartet, sept = septet, m = multiplet, brd = broad singlet. IR spectra were recorded on a Shimazu IR Prestige-21 FTIR Spectrometer. High-resolution mass spectra were obtained with a Finnigan MAT 95 XP mass spectrometer (Thermo Electron Corporation). Melting points were uncorrected and were recorded on a Büchi B-54 melting point apparatus.

Flash column chromatography was performed using Merck silica gel 60 with distilled solvents. $Cu(OAc)_2$ (98%) was purchased from Sigma-Aldrich Co., Inc.

2. Synthesis of alkyl hydroperoxide derivatives

2.1. Preparation of alkyl alcohols 5a–5s: a typical procedure for the synthesis of 2-(*o*-tolyl)propan-2-ol (5a)

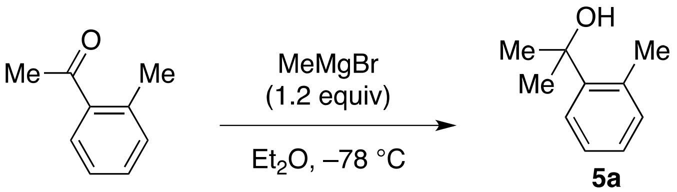

To a solution of 1-(2-methylphenyl)ethanone (2.01g, 15.0 mmol) in anhydrous diethyl ether (15 mL) was added 5.5 mL of MeMgBr (3.0 M in ether; 1.97g, 16.5 mmol) dropwise at −78 °C. The reaction was then allowed to warm up to room temperature and stirred for 1 h. After completion, the reaction was quenched with 1 N aqueous HCl at 0 °C and the organic materials were extracted twice with diethyl ether. The combined extracts were washed with brine, and dried over $MgSO_4$. Volatile materials were removed in vacuo and the crude material was purified by flash column chromatography (silica gel, hexane:ethyl acetate = 90:10) to give 2-(*o*-tolyl)propan-2-ol (**5a**) (1.94 g, 12.9 mmol) in 86% yield

2-(*o*-Tolyl)propan-2-ol (5a) [1]

White solid; ^{1}H NMR (400 MHz, $CDCl_3$) δ 1.64 (6H, s), 1.78 (1H, s), 2.58 (3H, s), 7.12-7.16 (3H, m), 7.41-7.46 (1H, m); ^{13}C NMR (100 MHz, $CDCl_3$) δ 22.2, 30.8, 73.6, 125.2, 125.6, 127.0, 132.6, 135.9, 145.7.

1,1,4-Triphenylbutan-1-ol (5b) [2]

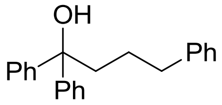

Prepared from benzophenone and $Ph(CH_2)_3MgBr$ and purified by flash column chromatography (silica gel, hexane:ethyl acetate = 90:10) in 42% yield; White solid; ^1H NMR (400 MHz, $CDCl_3$) δ 1.58-1.68 (2H, m), 2.06 (1H, brs), 2.26-2.33 (2H, m), 2.62 (2H, t, *J* = 7.6 Hz), 7.10-7.25 (7H, m), 7.28 (4H, dd, *J* = 7.6, 8.0 Hz), 7.36 (4H, d, *J* = 7.6 Hz); ^{13}C NMR (100 MHz, $CDCl_3$) δ 25.4, 36.0, 41.3, 78.2, 125.7, 126.0, 126.8, 128.1, 128.3, 128.4, 142.1, 147.0.

2,5-Diphenylpentan-2-ol (5c)

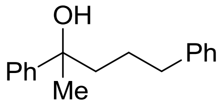

Prepared from 1-phenylethanone and $Ph(CH_2)_3MgBr$ and purified by flash column chromatography (silica gel, hexane:ethyl acetate = 90:10) in 94% yield; Colorless oil; IR (NaCl) 910, 1030, 1101, 1373, 1447, 1495, 1601, 2941, 2972, 3026, 3418 (brs) cm^{-1}; ^1H NMR (400 MHz, $CDCl_3$) δ 1.40-1.55 (1H, m), 1.53 (3H, s), 1.55-1.68 (1H, m), 1.78-1.90 (2H, m), 2.55 (2H, t, *J* = 7.6 Hz), 7.10 (2H, d, *J* = 7.2 Hz), 7.15 (1H, dd, *J* = 7.2, 7.6 Hz), 7.19-7.26 (3H, m), 7.32 (2H, ddd, *J* = 2.0, 7.6, 8.0 Hz), 7.39 (2H, dd, *J* = 1.2, 7.2 Hz); ^{13}C NMR (100 MHz, $CDCl_3$) δ 25.7, 30.2, 36.0, 43.6, 74.6, 124.7, 125.7, 126.5, 128.1, 128.2, 128.4, 142.2, 147.8; ESIHRMS: Found: *m*/*z* 241.1590. Calcd for $C_{17}H_{21}O$: $(M + H)^+$ 241.1592.

3,6-Diphenylhexan-3-ol (5d)

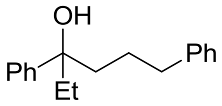

Prepared from propiophenone and $Ph(CH_2)_3MgBr$ and purified by flash column chromatography (silica gel, hexane:ethyl acetate = 90:10) in quantitative yield; Colorless oil; IR (NaCl) 953, 1030, 1447, 1454, 1495, 1603, 2938, 2967, 3024, 3470 (brs) cm^{-1}; ^1H NMR (400 MHz, $CDCl_3$) δ 0.73 (3H, t, *J* = 7.6 Hz), 1.33-1.45 (1H, m), 1.57-1.70 (2H, m), 1.74-1.91 (4H, m), 2.48-2.61 (2H, m), 7.09 (2H, dd, *J* = 1.2, 8.0 Hz), 7.14 (1H, dd, *J* = 7.2, 7.6 Hz), 7.18-7.26 (3H, m), 7.28-7.35 (4H, m); ^{13}C NMR (100 MHz, $CDCl_3$) δ 7.7, 25.2, 35.4, 36.1, 42.1, 77.1, 125.3, 125.7, 126.3, 128.0, 128.2, 128.3, 142.2, 145.8; ESIHRMS: Found: *m*/*z* 255.1759. Calcd for $C_{18}H_{23}O$: $(M + H)^+$ 255.1749.

2-(Naphthalen-2-yl)-5-phenylpentan-2-ol (5e)

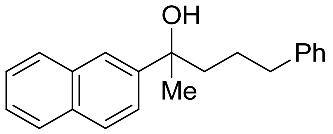

Prepared from 1-(2-naphthyl)ethanone and $Ph(CH_2)_3MgBr$ and purified by flash column chromatography (silica gel, hexane:ethyl acetate = 90:10) in 90% yield; Colorless oil; IR (NaCl) 1123, 1273, 1371, 1454, 1495, 1504, 1601, 2860, 2940, 2970, 3389 (brs) cm^{-1}; ^{1}H NMR (400 MHz, $CDCl_3$) δ 1.41-1.53 (1H, m), 1.58-1.70 (1H, m), 1.62 (3H, s), 1.84 (1H, brs), 1.92 (2H, dtt, J = 1.2, 11.6, 14.0 Hz), 2.55 (2H, ddd, J = 2.0, 7.0, 8.2 Hz), 7.08 (2H, d, J = 7.2 Hz), 7.14 (1H, ddd, J = 1.2, 7.2, 7.6 Hz), 7.18-7.25 (2H, m), 7.41-7.50 (3H, m), 7.77-7.84 (3H, m), 7.85 (1H, d, J = 1.2 Hz); ^{13}C NMR (100 MHz, $CDCl_3$) δ 25.8, 30.3, 36.0, 43.4, 74.8, 123.1, 123.6, 125.6, 125.7, 126.0, 127.4, 127.8, 128.1, 128.2, 128.4, 132.2, 133.1, 142.1, 145.1; ESIHRMS: Found: *m*/*z* 291.1754. Calcd for $C_{21}H_{23}O$: $(M + H)^+$ 291.1749.

2-(4-Bromophenyl)-5-phenylpentan-2-ol (5f)

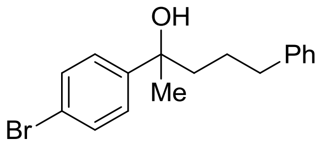

Prepared from 1-(4-bromophenyl)ethanone and $Ph(CH_2)_3MgBr$ and purified by flash column chromatography (silica gel, hexane:ethyl acetate = 90:10) in 91% yield; Colorless oil; IR (NaCl) 1395, 1452, 1487, 1495, 1591, 1603, 2860, 2941, 2972, 3416 (brs) cm^{-1}; ^{1}H NMR (400 MHz, $CDCl_3$) δ 1.39-1.50 (1H, m), 1.51 (3H, s), 1.55-1.67 (2H, m), 1.80 (2H, dtt, J = 5.6, 10.8, 11.2 Hz); 2.55 (2H, ddd, J = 2.4, 6.4, 8.8 Hz), 7.10 (2H, d, J = 6.8 Hz), 7.16 (1H, dd, J = 7.2, 7.6 Hz), 7.23-7.28 (4H, m), 7.43 (2H, td, J = 2.4, 8.4 Hz); ^{13}C NMR (100 MHz, $CDCl_3$) δ 25.6, 30.3, 35.9, 43.5, 74.4, 120.4, 125.8, 126.7, 128.27, 128.34, 131.1, 142.0, 146.8; ESIHRMS: Found: *m*/*z* 319.0695. Calcd for $C_{17}H_{20}O^{79}Br$: $(M + H)^+$ 319.0698.

3-Methyl-1,6-diphenylhexan-3-ol (5g)

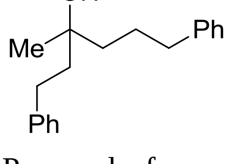

Prepared from 4-phenylbutan-2-one and $Ph(CH_2)_3MgBr$ and purified by flash column chromatography (silica gel, hexane:ethyl acetate = 90:10) in 99% yield; Colorless oil; IR (NaCl) 910, 1373, 1454, 1495, 1603, 2862, 2940, 3026, 3406 (brs) cm^{-1}; ^{1}H NMR (400 MHz, $CDCl_3$) 1.22 (3H, s), 1.52-1.57 (2H, m), 1.64-1.78 (4H, m), 2.59-2.66 (4H, m), 7.14-7.21 (6H, m), 7.24-7.31 (4H, m); ^{13}C NMR (100 MHz, $CDCl_3$) δ 25.9, 26.9, 30.3, 36.3, 41.5, 43.7, 72.6, 125.7, 125.8, 128.29,

128.31, 128.4, 142.3, 142.5; ESIHRMS: Found: *m/z* 269.1901. Calcd for $C_{19}H_{25}O$: $(M + H)^+$ 269.1905.

1-(3-Phenylpropyl)cyclohexanol (5h)

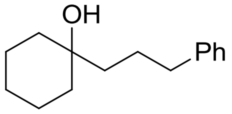

Prepared from cyclohexanone and $Ph(CH_2)_3MgBr$ and purified by flash column chromatography (silica gel, hexane:ethyl acetate = 90:10) in 81% yield; Colorless oil; IR (NaCl) 968, 1171, 1256, 1454, 1495, 1603, 1697, 2857, 2932, 3404 (brs) cm^{-1}; ^1H NMR (400 MHz, $CDCl_3$) δ 1.36-1.60 (12H, m), 1.66-1.74 (2H, m), 2.61 (2H, t, *J* = 7.6 Hz), 7.18 (1H, t, *J* = 8.0 Hz), 7.19 (2H, d, *J* = 7.6 Hz), 7.27 (2H, dd, *J* = 7.2, 8.0 Hz); ^{13}C NMR (100 MHz, $CDCl_3$) δ 22.2, 24.8, 25.8, 36.4, 37.4, 42.0, 71.4, 125.7, 128.2, 128.4, 142.5; ESIHRMS: Found: *m/z* 219.1752. Calcd for $C_{15}H_{23}O$: $(M + H)^+$ 219.1749.

1,1-Diphenylpentan-1-ol (5i) [3]

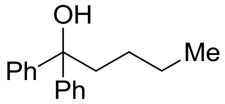

Prepared from benzophenone and *n*-BuLi (1.6 M in hexanes) and purified by flash column chromatography (silica gel, hexane:ethyl acetate = 90:10) in 48% yield; Colorless oil; ^1H NMR (400 MHz, $CDCl_3$) δ 0.87 (3H, t, *J* = 7.2 Hz), 1.20-1.30 (2H, m), 1.30-1.40 (2H, m), 2.10 (1H, s), 2.27 (2H, t, *J* = 8.0 Hz), 7.21 (2H, dd, *J* = 6.8, 7.6 Hz), 7.30 (4H, dd, *J* = 7.2, 8.0 Hz), 7.41 (4H, d, *J* = 8.0 Hz); ^{13}C NMR (100 MHz, $CDCl_3$) δ 14.0, 23.1, 25.9, 41.7, 78.2, 126.0, 126.7, 128.1, 147.2.

2-Phenylhexan-2-ol (5j) [4]

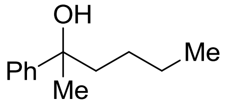

Prepared from 2-hexanone and PhMgBr and purified by flash column chromatography (silica gel, hexane:ethyl acetate = 90:10) in quantitative yield; Colorless oil; ^1H NMR (400 MHz, $CDCl_3$) δ 0.84 (3H, t, *J* = 7.2 Hz), 1.06-1.17 (1H, m), 1.18-1.31 (3H, m), 1.55 (3H, s), 1.74 (1H, s), 1.75-1.88 (2H, m), 7.23 (1H, d, *J* = 7.2, 7.6 Hz), 7.33 (2H, dd, *J* = 7.2, 8.0 Hz), 7.43 (2H, dd, *J* = 1.2, 8.0 Hz); ^{13}C NMR (100 MHz, $CDCl_3$) δ 14.0, 23.0, 26.1, 30.1, 43.9, 74.7, 124.8, 126.4, 128.1, 148.1.

3-Methyl-1-phenylheptan-3-ol (5k) [5]

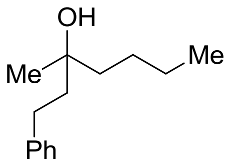

Prepared from 2-hexanone and $Ph(CH_2)_3MgBr$ and purified by flash column chromatography (silica gel, hexane:ethyl acetate = 90:10) in quantitative yield; Colorless oil; ^{1}H NMR (400 MHz, $CDCl_3$) δ 0.93 (3H, t, J = 7.2 Hz), 1.24 (3H, s), 1.25 (1H, s), 1.30-1.39 (4H, m), 1.48-1.56 (2H, m), 1.73-1.80 (2H, m), 2.64-2.70 (2H, m), 7.16-7.22 (3H, m), 7.28 (2H, ddd, J = 1.6, 7.2, 7.6 Hz); ^{13}C NMR (100 MHz, $CDCl_3$) δ 14.1, 23.3, 26.2, 26.9, 30.3, 41.8, 43.7, 72.7, 125.7, 128.3, 128.4, 142.6.

1-Butylcyclohexanol (5l) [5]

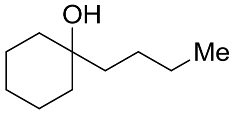

Prepared from cyclohexanone and n-BuLi (1.6 M in hexanes) and purified by flash column chromatography (silica gel, hexane:ethyl acetate = 90:10) in 90% yield; Colorless oil; ^{1}H NMR (400 MHz, $CDCl_3$) δ 0.91 (3H, t, J = 7.2 Hz), 1.20-1.37 (6H, m), 1.37-1.60 (11H, m); ^{13}C NMR (100 MHz, $CDCl_3$) δ 14.1, 22.3, 23.3, 25.1, 25.9, 37.4, 42.2, 71.4.

2-Phenyldecan-2-ol (5m) [6]

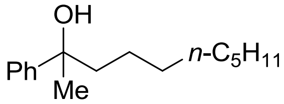

Prepared from 1-phenylethanone and $CH_3(CH_2)_7MgBr$ and purified by flash column chromatography (silica gel, hexane:ethyl acetate = 90:10) in 98% yield; Colorless oil; ^{1}H NMR (400 MHz, $CDCl_3$) δ 0.86 (3H, t, J = 6.8 Hz), 1.08-1.18 (1H, m), 1.18-1.30 (11H, m), 1.55 (3H, s), 1.75 (1H, s), 1.72-1.84 (2H, m), 7.23 (1H, tt, J = 1.2, 7.6 Hz), 7.33 (2H, dt, J = 1.6, 7.6 Hz), 7.42 (2H, dd, J = 1.2, 8.0 Hz); ^{13}C NMR (100 MHz, $CDCl_3$) δ 14.1, 22.6, 23.9, 29.2, 29.5, 29.9 30.1, 31.8, 44.2, 74.7, 124.7, 126.4, 128.1, 148.1.

2-Cyclohexyl-1,1-diphenylethanol (5n)

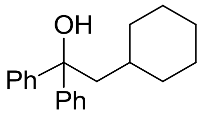

Prepared from ethyl 2-cyclohexylacetate and PhMgBr (2.2 equiv) and purified by flash column chromatography (silica gel, hexane:ethyl acetate = 90:10) in quantitative yield; Colorless oil; IR (NaCl) 1032, 1059, 1447, 1493, 1599, 2849, 2922, 3445 (brs) cm^{-1}; ^{1}H NMR (400 MHz, $CDCl_3$) δ 0.90-1.05 (2H, m), 1.05-1.20 (3H, m), 1.38-1.48 (1H, m), 1.48-1.62 (5H, m), 2.03 (1H, s), 2.21 (2H, d, J = 5.2 Hz), 7.20 (2H, t, J = 7.2 Hz), 7.29 (4H, dd, J = 7.2, 8.0 Hz), 7.41 (4H, d, J = 7.6 Hz); ^{13}C

NMR (100 MHz, $CDCl_3$) δ 26.2, 26.3, 33.5, 35.2, 49.2, 78.8, 126.0, 126.6, 128.0, 147.7; ESIHRMS: Found: *m*/*z* 281.1911. Calcd for $C_{20}H_{25}O$: $(M + H)^+$ 281.1905.

1,6-Diphenylhexan-3-ol (5o)

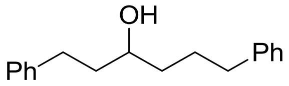

Prepared from 3-phenylpropanal and $Ph(CH_2)_3MgBr$ and purified by flash column chromatography (silica gel, hexane:ethyl acetate = 90:10) in 95% yield; White solid; mp. 37–38 °C; IR (NaCl) 1454, 1495, 1603, 2860, 2938, 3015, 3429 (brs) cm^{-1}; ^1H NMR (400 MHz, $CDCl_3$) δ 1.45 (1H, brs), 1.47-1.57 (2H, m), 1.57-1.82 (4H, m), 2.55-2.67 (3H, m), 2.76 (1H, ddd, *J* = 5.6, 9.6, 13.6 Hz), 3.57-3.65 (1H, m), 7.12-7.22 (6H, m), 7.22-7.30 (4H, m); ^{13}C NMR (100 MHz, $CDCl_3$) δ 27.3, 32.0, 35.8, 37.0, 39.0, 71.1, 125.7, 125.8, 128.26, 128.35 (overlapped×2), 142.1, 142.3; ESIHRMS: Found: *m*/*z* 255.1754. Calcd for $C_{18}H_{23}O$: $(M + H)^+$ 255.1749.

4-Methyl-1,1-diphenylpentan-1-ol (5p)

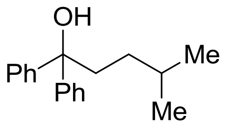

Prepared from benzophenone and $(CH_3)_2CH(CH_2)_2MgBr$ and purified by flash column chromatography (silica gel, hexane:ethyl acetate = 90:10) in 29% yield; White solid; mp. 49–50 °C; IR (NaCl) 1032, 1165, 1447, 1599, 1653, 2868, 2953, 3472 (brs) cm^{-1}; ^1H NMR (400 MHz, $CDCl_3$) δ 0.87 (6H, d, *J* = 6.4 Hz), 1.14-1.20 (2H, m), 1.50-1.60 (1H, m), 2.11 (1H, s), 2.25-2.30 (2H, m), 7.21 (2H, tt, *J* = 1.2, 7.6 Hz), 7.29 (4H, ddd, *J* = 2.0, 6.4, 7.6 Hz), 7.38-7.43 (4H, m); ^{13}C NMR (100 MHz, $CDCl_3$) δ 22.6, 28.4, 32.6, 39.8, 78.2, 126.0, 126.7, 128.1, 147.1; ESIHRMS: Found: *m*/*z* 255.1754. Calcd for $C_{18}H_{23}O$: $(M + H)^+$ 255.1749.

5-Methyl-2-phenylhexan-2-ol (5q) [7]

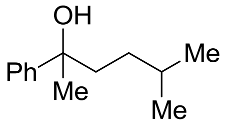

Prepared from 1-phenylethanone and $(CH_3)_2CH(CH_2)_2MgBr$ and purified by flash column chromatography (silica gel, hexane:ethyl acetate = 90:10) in quantitative yield; Colorless oil; ^1H NMR (400 MHz, $CDCl_3$) δ 0.83 (3H, d, *J* = 6.4 Hz), 0.84 (3H, d, *J* = 6.4 Hz), 0.96-1.07 (1H, m), 1.10-1.20 (1H, m), 1.40-1.51 (1H, m), 1.55 (3H, s), 1.73-1.87 (2H, m), 1.76 (1H, s), 7.23 (1H, tt, *J* = 1.6, 7.6 Hz), 7.33 (2H, dt, *J* = 1.6, 8.0 Hz), 7.42 (2H, dd, *J* = 1.6, 8.0 Hz); ^{13}C NMR (100 MHz, $CDCl_3$) δ 22.5, 22.6, 28.3, 30.2, 32.8, 41.9, 74.7, 124.8, 126.4, 128.1, 148.1.

3-Cyclohexyl-1,1-diphenylpropan-1-ol (5r)

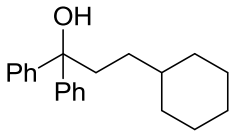

Prepared from benzophenone and $Cy(CH_2)_2MgBr$ and purified by flash column chromatography (silica gel, hexane:ethyl acetate = 90:10) in 28% yield; White solid; mp. 61–62 °C; IR (NaCl) 1279, 1319, 1447, 1493, 1655, 2849, 2922, 3472 (brs) cm^{-1}; ^1H NMR (400 MHz, $CDCl_3$) δ 0.80-0.90 (2H, m), 1.08-1.27 (7H, m), 1.60-1.73 (4H, m), 2.13 (1H, brs), 2.25-2.31 (2H, m), 7.20 (2H, dd, J = 6.8, 7.2 Hz), 7.29 (4H, dd, J = 7.2, 7.6 Hz), 7.40 (4H, d, J = 8.0 Hz); ^{13}C NMR (100 MHz, $CDCl_3$) δ 26.3, 26.6, 31.2, 33.3, 38.1, 39.3, 78.3, 126.0, 126.7, 128.1, 147.2; ESIHRMS: Found: *m*/*z* 295.2061. Calcd for $C_{21}H_{27}O$: $(M + H)^+$ 295.2062.

3-(Adamantan-2-yl)-1,1-diphenylpropan-1-ol (5s)

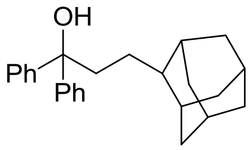

Prepared from 3-(adamantan-2-yl)-1-phenylpropan-1-one (see below) and PhMgBr and purified by flash column chromatography (silica gel, hexane:ethyl acetate = 90:10) in 94% yield; White solid; mp. 114–115 °C; IR (NaCl) 1031, 1057, 1447, 1493, 1597, 2849, 2905, 3447 (brs) cm^{-1}; ^1H NMR (400 MHz, $CDCl_3$) δ 1.38-1.46 (4H, m), 1.62 (1H, t, J = 7.2 Hz), 1.66-1.76 (9H, m), 1.78.1.87 (3H, m), 2.14 (1H, s), 2.23-2.28 (2H, m), 7.21 (2H, tt, J = 1.2, 7.2 Hz), 7.30 (4H, ddd, J = 2.0, 6.4, 7.2 Hz), 7.40-7.45 (4H, m); ^{13}C NMR (100 MHz, $CDCl_3$) δ 26.5, 28.0, 28.3, 31.6, 31.9, 38.4, 39.2, 40.2, 44.8, 78.3, 126.0, 126.7, 128.1, 147.2; ESIHRMS: Found: *m*/*z* 347.2373. Calcd for $C_{25}H_{31}O$: $(M + H)^+$ 347.2375.

3-(Adamantan-2-yl)-1-phenylpropan-1-one

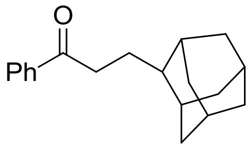

Prepared from (2-(adamantan-2-yl)ethyl)magnesium bromide and benzonitrile in THF and followed by hydrolysis with 1 mL HCl at reflux temperature for 2 days; White solid; mp. 109–111 °C; IR (NaCl) 1447, 1580, 1597, 1682, 2849, 2899 cm^{-1}; ^1H NMR (400 MHz, $CDCl_3$) δ 1.52 (2H, d, J = 12.4 Hz), 1.68-1.79 (7H, m), 1.79-1.95 (8H, m), 2.96 (2H, t, J = 8.0 Hz), 7.46 (2H, dt, J = 1.2, 6.4 Hz), 7.55 (1H, tt, J = 1.6, 7.2 Hz), 7.95-8.00 (2H, m); ^{13}C NMR (100 MHz, $CDCl_3$) δ 27.3, 28.0, 28.3, 31.6, 31.9, 37.0, 38.3, 39.2, 44.3, 128.1, 128.5, 132.8, 137.1, 200.9; ESIHRMS: Found: *m*/*z* 269.1919. Calcd for $C_{19}H_{25}O$: $(M + H)^+$ 269.1905.

2.2. Preparation of 2-(*o*-tolyl)propyl 2-hydroperoxide (1a)

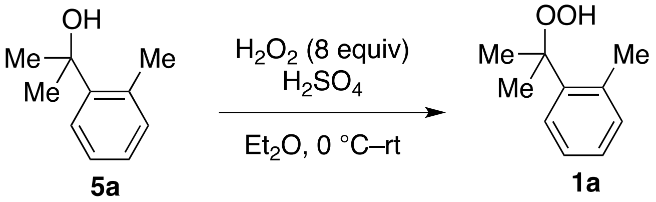

To a stirred solution of 2-(*o*-tolyl)propan-2-ol (**5a**) (1.94 g, 12.9 mmol) in 3 mL of diethyl ether at 0 °C was added 11.7 mL of H_2O_2 (30 wt % in H_2O; 3.51 g, 103.2 mmol) and 13 drops of H_2SO_4 (conc) dropwise. The reaction mixture was stirred at room temperature for 24 h. The reaction was quenched with H_2O and the organic materials were extracted three times with diethyl ether. The combined extracts were washed with H_2O (3 times) and dried over $MgSO_4$. Volatile materials were removed in vacuo and the crude material was purified by flash column chromatography (silica gel, hexane:ethyl acetate = 90:10) to give hydroperoxide **1a** (1.72 g, 10.3 mmol) in 80% yield.

2-(*o*-Tolyl)propyl 2-hydroperoxide (1a) [8]

Colorless oil; ^{1}H NMR (400 MHz, $CDCl_3$) δ 1.66 (6H, s), 2.58 (3H, s), 7.16-7.20 (3H m), 7.31 (1H, s), 7.34-7.38 (1H, m); ^{13}C NMR (100 MHz, $CDCl_3$) δ 21.6, 26.1, 85.5, 125.9, 126.9, 127.6, 132.8, 136.0, 141.5.

2.3. Preparation of alkyl peroxides 1b–1n, 1p–1s: a typical procedure for the synthesis of 1,1,4-triphenylbutyl 1-hydroperoxide (1b)

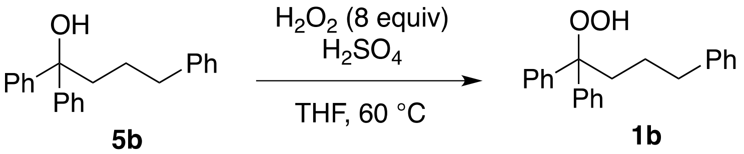

To a stirred solution of 1,1,4-triphenylbutan-1-ol (**5b**) (2.54 g, 8.4 mmol) in 3 mL of THF at 0 °C was added 7.6 mL of H_2O_2 (30 wt % in H_2O; 2.29 g, 67.2 mmol) and 8 drops of H_2SO_4 (conc) dropwise. The reaction mixture was stirred at 60 °C for 24 h. The reaction was quenched with H_2O and the organic materials were extracted three times with diethyl ether. The combined extracts were washed with H_2O (3 times) and dried over $MgSO_4$. (The combined aqueous extracts were quenched with an excess amount of $Na_2S_2O_2$.) Volatile materials were removed in vacuo and the crude material was purified by flash column chromatography (silica gel, hexane:ethyl acetate = 90:10) to give hydroperoxide **1b** (2.59 g, 8.1 mmol) in 97% yield.

1,1,4-Triphenylbutyl hydroperoxide (1b)

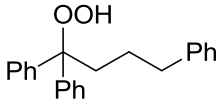

White solid; mp. 61–62 °C; IR (NaCl) 1030, 1059, 1337, 1447, 1495, 1601, 2955, 3061, 3472 (brs) cm^{-1}; ^{1}H NMR (400 MHz, $CDCl_3$) δ 1.57-1.67 (2H, m), 2.43-2.49 (2H, m), 2.62 (2H, t, *J* = 7.6 Hz), 7.04 (1H, s), 7.10-7.18 (3H, m), 7.22-7.27 (4H, m), 7.27-7.33 (8H, m); ^{13}C NMR (100 MHz, $CDCl_3$) δ 24.8, 35.6, 35.9, 89.5, 125.7, 126.8, 127.3, 128.15, 128.24, 128.4, 142.2, 143.1; ESIHRMS: Found: *m*/*z* 319.1705. Calcd for $C_{22}H_{23}O_2$: $(M + H)^+$ 319.1698.

2,5-Diphenylpentyl 2-hydroperoxide (1c)

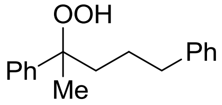

Prepared from 1-phenylethanone and 2,5-diphenylpentan-2-ol (**5c**) for 12 h and purified by flash column chromatography (silica gel, hexane:ethyl acetate = 90:10) in 85% yield; Colorless oil; IR (NaCl) 1030, 1373, 1447, 1495, 2862, 2945, 3024, 3424 (brs) cm^{-1}; ^{1}H NMR (400 MHz, $CDCl_3$) δ 1.46-1.58 (1H, m), 1.58-1.71 (1H, m), 1.63 (3H, s), 1.78-1.90 (2H, m), 2.49-2.62 (2H, m), 7.11 (2H, d, *J* = 7.2 Hz), 7.15 (1H, dd, *J* = 7.2, 7.6 Hz), 7.19 (1H, s), 7.22-7.30 (3H, m), 7.33-7.41 (4H, m); ^{13}C NMR (100 MHz, $CDCl_3$) δ 22.6, 25.6, 36.0, 39.2, 86.3, 125.6, 125.8, 127.3, 128.27, 128.34, 128.5, 142.0, 143.8; ESIHRMS: Found: *m*/*z* 257.1553. Calcd for $C_{17}H_{21}O_2$: $(M + H)^+$ 257.1542.

3,6-Diphenylhexyl 3-hydroperoxide (1d)

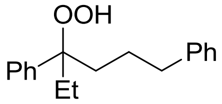

Prepared from 3,6-diphenylhexan-3-ol (**5d**) for 4 days and purified by flash column chromatography (silica gel, hexane:ethyl acetate = 90:10) in 86% yield; Colorless oil; IR (NaCl) 1030, 1327, 1447, 1495, 1603, 2878, 2941, 2967, 3474 (brs) cm^{-1}; ^{1}H NMR (400 MHz, $CDCl_3$) δ 0.77 (3H, t, *J* = 7.2 Hz), 1.45-1.55 (1H, m), 1.55-1.67 (1H, m), 1.83-1.98 (4H, m), 2.59 (2H, t, *J* = 7.6 Hz), 7.01 (1H, s), 7.12-7.19 (3H, m), 7.22-7.30 (3H, m), 7.30-7.38 (4H, m); ^{13}C NMR (100 MHz, $CDCl_3$) δ 7.6, 24.9, 27.8, 34.0, 36.0, 88.5, 125.8, 125.9, 127.1, 128.3, 128.4, 142.2, 142.9; ESIHRMS: Found: *m*/*z* 271.1690. Calcd for $C_{18}H_{23}O_2$: $(M + H)^+$ 271.1698.

2-(Naphthalen-2-yl)-5-phenylpentyl 2-hydroperoxide (1e)

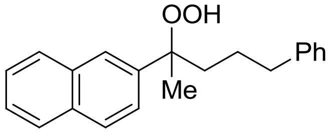

Prepared from 2-(naphthalen-2-yl)-5-phenylpentan-2-ol (**5e**) for 7 h and purified by flash column chromatography (silica gel, hexane:ethyl acetate = 90:10) in 34% yield; Colorless oil; IR (NaCl) 1375, 1454, 1495, 1601, 2862, 2945, 2982, 3059, 3441 (brs) cm^{-1}; ^{1}H NMR (400 MHz, $CDCl_3$) δ 1.47-1.57 (1H, m), 1.61-1.73 (1H, m), 1.74 (3H, s), 1.90-1.97 (2H, m), 2.50-2.63 (2H, m), 7.09 (2H, dd, *J* = 1.2, 7.2 Hz), 7.15 (1H, tt, *J* = 2.0, 7.2 Hz), 7.20-7.28 (3H, m), 7.44-7.52 (2H, m), 7.55 (1H, dd, *J* = 2.0, 8.4 Hz), 7.79-7.87 (4H, m); ^{13}C NMR (100 MHz, $CDCl_3$) δ 22.6, 25.7, 36.0, 39.1, 86.5, 123.7, 124.8, 125.8, 126.0, 126.2, 127.5, 128.2, 128.26, 128.35 (overlapped), 132.6, 133.1, 141.2, 142.0; ESIHRMS: Found: *m/z* 307.1703. Calcd for $C_{21}H_{23}O_2$: $(M + H)^+$ 307.1698.

2-(4-Bromophenyl)-5-phenylpentyl 2-hydroperoxide (1f)

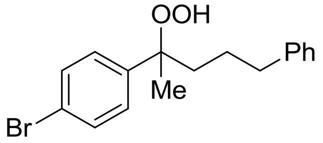

Prepared from 2-(4-bromophenyl)-5-phenylpentan-2-ol (**5f**) and 1 mL of H_2SO_4 (conc) for 24 h and purified by flash column chromatography (silica gel, hexane:ethyl acetate = 90:10) in 68% yield; Colorless oil; IR (NaCl) 1009, 1086, 1396, 1452, 1487, 1591, 1603, 2862, 2945, 3416 (brs) cm^{-1}; ^{1}H NMR (400 MHz, $CDCl_3$) δ 1.43-1.54 (1H, m), 1.54-1.67 (1H, m), 1.60 (3H, s), 1.74-1.85 (2H, m), 2.50-2.61 (2H, m), 7.11 (2H, dd, *J* = 1.2, 7.2 Hz), 7.17 (1H, tt, *J* = 2.0, 7.2 Hz), 7.21-7.28 (5H, m), 7.48 (2H, dt, *J* = 1.6, 8.8 Hz); ^{13}C NMR (100 MHz, $CDCl_3$) δ 22.6, 25.5, 35.9, 39.0, 85.9, 121.3, 125.8, 127.5, 128.30, 128.32, 131.5, 141.8, 143.0; ESIHRMS: Found: *m/z* 335.0640. Calcd for $C_{17}H_{20}O_2{}^{79}Br$: $(M + H)^+$ 335.0647.

3-Methyl-1,6-diphenylhexyl 3-hydroperoxide (1g) [9]

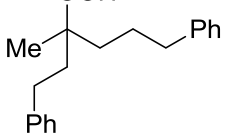

Prepared from 3-methyl-1,6-diphenylhexan-3-ol (**5g**) and 1 mL of H_2SO_4(conc) for 24 h and purified by flash column chromatography (silica gel, hexane:ethyl acetate = 90:10) in 51% yield; Colorless oil; ^{1}H NMR (400 MHz, $CDCl_3$) 1.22 (3H, s), 1.60-1.75 (4H, m), 1.78-1.92 (2H, m), 2.57-2.65 (4H, m), 6.99 (1H, s), 7.15-7.22 (6H, m), 7.24-7.31 (4H, m); ^{13}C NMR (100 MHz, $CDCl_3$) δ 21.5, 25.5, 29.9, 36.0, 36.2, 38.4, 84.4, 125.79, 125.82, 128.30, 128.34, 128.4, 142.3, 142.5.

1-(3-Phenylpropyl)cyclohexyl hydroperoxide (1h)

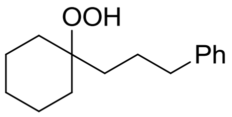

Prepared from 1-(3-phenylpropyl)cyclohexanol (**5h**) and 1 mL of H_2SO_4 (conc) for 18 h and purified by flash column chromatography (silica gel, hexane:ethyl acetate = 90:10) in 26% yield; White solid; mp. 35–36 °C; IR (NaCl) 1315, 1452, 1495, 1603, 2862, 2936, 3024, 3406 (brs) cm^{-1}; ^{1}H NMR (400 MHz, $CDCl_3$) δ 1.21-1.45 (5H, m), 1.48-1.62 (5H, m), 1.62-1.78 (4H, m), 2.62 (2H, t, *J* = 7.6 Hz), 6.93 (1H, brs), 7.15-7.22 (3H, m), 7.28 (2H, t, *J* = 7.6 Hz); ^{13}C NMR (100 MHz, $CDCl_3$) δ 21.9, 24.6, 25.8, 32.5, 35.8, 36.2, 83.1, 125.7, 128.3, 128.4, 142.5; ESIHRMS: Found: *m*/*z* 235.1705. Calcd for $C_{15}H_{23}O_2$: $(M + H)^+$ 235.1698.

1,1-Diphenylpentan hydroperoxide (1i)

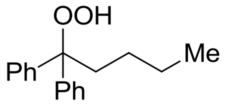

Prepared from 1,1-diphenylpentan-1-ol (**5i**) for 8 h and purified by flash column chromatography (silica gel, hexane:ethyl acetate = 90:10) in 82% yield; Colorless oil; IR (NaCl) 1032, 1337, 1447, 1493, 1599, 2870, 2955, 3470 (brs) cm^{-1}; ^{1}H NMR (400 MHz, $CDCl_3$) δ 0.87 (3H, t, *J* = 7.2 Hz), 1.19-1.28 (2H, m), 1.28-1.38 (2H, m), 2.38-2.45 (2H, m), 7.11 (1H, brs), 7.23-7.29 (2H, m), 7.29-7.38 (8H, m); ^{13}C NMR (100 MHz, $CDCl_3$) δ 14.0, 23.0, 25.5, 36.1, 89.6, 126.9, 127.2, 128.1, 143.3; ESIHRMS: Found: *m*/*z* 257.1531. Calcd for $C_{17}H_{21}O_2$: $(M + H)^+$ 257.1542.

2-Phenylhexyl 2-hydroperoxide (1j) [10]

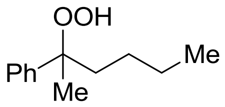

Prepared from 2-phenylhexan-2-ol (**5j**) for 5 h and purified by flash column chromatography (silica gel, hexane:ethyl acetate = 90:10) in 90% yield; Colorless oil; ^{1}H NMR (400 MHz, $CDCl_3$) δ 0.85 (3H, t, *J* = 7.2 Hz), 1.08-1.17 (1H, m), 1.20-1.33 (3H, m), 1.65 (3H, s), 1.72-1.84 (2H, m), 7.22 (1H, s), 7.29 (1H, tt, *J* = 1.6, 7.2 Hz), 7.38 (2H, ddd, *J* = 1.6, 7.2, 8.0 Hz), 7.43 (2H, dd, *J* = 1.2, 8.4 Hz); ^{13}C NMR (100 MHz, $CDCl_3$) δ 13.9, 22.4, 23.0, 26.0, 39.6, 86.5, 125.7, 127.3, 128.5, 144.0.

3-Methyl-1-phenylheptyl 3-hydroperoxide (1k)

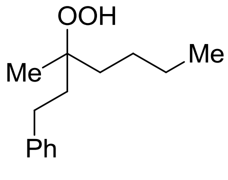

Prepared from 3-methyl-1-phenylheptan-3-ol (**5k**) and 1 mL of H_2SO_4(conc) for 48 h and purified by flash column chromatography (silica gel, hexane:ethyl acetate = 90:10) in 42% yield; Colorless

oil; IR (NaCl) 1373, 1454, 1495, 1603, 1705, 2868, 2936, 2953, 3393 (brs) cm^{-1}; ^{1}H NMR (400 MHz, $CDCl_3$) δ 0.92 (3H, t, *J* = 7.2 Hz), 1.24 (3H, s), 1.28-1.38 (4H, m), 1.56-1.64 (2H, m), 1.86 (2H, ddd, *J* = 1.6, 6.4, 10.4 Hz), 2.65 (2H, t, *J* = 8.4 Hz), 7.11 (1H, brs), 7.15-7.23 (3H, m), 7.28 (2H, dd, *J* = 7.2, 7.6 Hz); ^{13}C NMR (100 MHz, $CDCl_3$) δ 14.1, 21.5, 23.2, 25.9, 29.9, 36.3, 38.4, 84.5, 125.8, 128.3, 128.4, 142.6; ESIHRMS: Found: *m*/*z* 223.1707. Calcd for $C_{14}H_{23}O_2$: $(M + H)^+$ 223.1698.

1-Butylcyclohexyl hydroperoxide (1l)

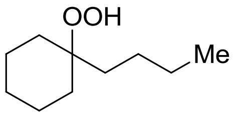

Prepared from 1-butylcyclohexanol (**5l**) for 2 days and purified by flash column chromatography (silica gel, hexane:ethyl acetate = 90:10) in 35% yield; Colorless oil; IR (NaCl) 959, 1150, 1260, 1377, 1447, 2860, 2932, 3385 (brs) cm^{-1}; ^{1}H NMR (400 MHz, $CDCl_3$) δ 0.92 (3H, t, *J* = 6.8 Hz), 1.24-1.48 (9H, m), 1.48-1.60 (5H, m), 1.72-1.80 (2H, m), 7.03 (1H, t, *J* = 3.6 Hz); ^{13}C NMR (100 MHz, $CDCl_3$) δ 14.1, 21.9, 23.3, 25.1, 25.8, 32.5, 36.1, 83.2; ESIHRMS: Found: *m*/*z* 173.1550. Calcd for $C_{10}H_{21}O_2$: $(M + H)^+$ 173.1542.

2-Phenyldecyl 2-hydroperoxide (1m)

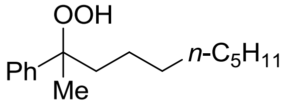

Prepared from 2-phenyldecan-2-ol (**5m**) for 20 h and purified by flash column chromatography (silica gel, hexane:ethyl acetate = 90:10) in 79% yield; Colorless oil; IR (NaCl) 1028, 1070, 1373, 1447, 1466, 1495, 2853, 2924, 3416 (brs) cm^{-1}; ^{1}H NMR (400 MHz, $CDCl_3$) δ 0.86 (3H, t, *J* = 6.8 Hz), 1.00-1.18 (1H, m), 1.18-1.32 (11H, m), 1.65 (3H, s), 1.72-1.83 (2H, m), 7.16 (1H, s), 7.29 (1H, t, *J* = 7.2 Hz), 7.38 (2H, dd, *J* = 7.2, 8.0 Hz), 7.43 (2H, dd, *J* = 1.6, 7.2 Hz); ^{13}C NMR (100 MHz, $CDCl_3$) δ 14.1, 22.4, 22.6, 23.8, 29.2, 29.4, 30.0, 31.8, 39.8, 86.5, 125.7, 127.3, 128.5, 144.1; ESIHRMS: Found: *m*/*z* 251.2010. Calcd for $C_{16}H_{27}O_2$: $(M + H)^+$ 251.2011.

2-Cyclohexyl-1,1-diphenylethyl hydroperoxide (1n)

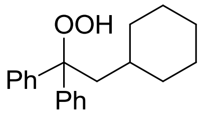

Prepared from 2-cyclohexyl-1,1-diphenylethanol (**5n**) for 24 h and purified by flash column chromatography (silica gel, hexane:ethyl acetate = 90:10) in 94% yield; White solid; mp. 60–62 °C; IR (NaCl) 1339, 1449, 1491, 2851, 2924, 3474 (brs) cm^{-1}; ^{1}H NMR (400 MHz, $CDCl_3$) δ 0.82-1.00 (3H, m), 1.02-1.14 (3H, m), 1.28-1.40 (1H, m), 1.50-1.60 (4H, m), 2.33 (2H, d, *J* = 5.2 Hz), 7.04 (1H, s), 7.24 (2H, tt, *J* = 1.6, 7.2 Hz), 7.31 (4H, ddd, *J* = 1.6, 6.8, 8.0 Hz), 7.35 (4H, dd, *J* = 2.0, 8.0

Hz); ^{13}C NMR (100 MHz, $CDCl_3$) δ 26.28. 26.35, 32.9, 34.7, 43.4, 89.9, 126.9, 127.2, 128.1, 143.7; ESIHRMS: Found: *m*/*z* 297.1849. Calcd for $C_{20}H_{25}O_2$: $(M + H)^+$ 297.1855.

4-Methyl-1,1-diphenylpentyl hydroperoxide (1p)

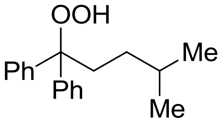

Prepared from 4-methyl-1,1-diphenylpentan-1-ol (**5p**) for 10 h and purified by flash column chromatography (silica gel, hexane:ethyl acetate = 90:10) in 83% yield; White solid; mp. 63–65 °C; IR (NaCl) 1337, 1447, 1468, 1493, 2870, 2932, 2957, 3472 (brs) cm^{-1}; ^{1}H NMR (400 MHz, $CDCl_3$) δ 0.86 (6H, d, *J* = 6.8 Hz), 1.10-1.18 (2H, m), 1.53 (1H, sept, *J* = 2.8 Hz), 2.40-2.45 (2H, m), 7.09 (1H, s), 7.25 (2H, tt, *J* = 1.6, 7.2 Hz), 7.28-7.37 (8H, m); ^{13}C NMR (100 MHz, $CDCl_3$) δ 22.6, 28.3, 32.1, 34.1, 89.6, 126.9, 127.2, 128.1, 143.3; ESIHRMS: Found: *m*/*z* 271.1709. Calcd for $C_{18}H_{23}O_2$: $(M + H)^+$ 271.1698.

5-Methyl-2-phenylhexyl 2-hydroperoxide (1q) [7]

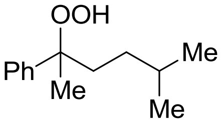

Prepared from 5-methyl-2-phenylhexan-2-ol (**5q**) for 5 h and purified by flash column chromatography (silica gel, hexane:ethyl acetate = 90:10) in 94% yield; Colorless oil; ^{1}H NMR (400 MHz, $CDCl_3$) δ 0.82 (3H, d, *J* = 6.8 Hz), 0.84 (3H, d, *J* = 6.8 Hz), 0.98-1.07 (1H, m), 1.14-1.24 (1H, m), 1.40-1.50 (1H, m), 1.65 (3H, s), 1.72-1.84 (2H, m), 7.19 (1H, s), 7.29 (1H, dd, *J* = 6.8, 7.2 Hz), 7.38 (2H, dd, *J* = 7.2, 8.0 Hz), 7.43 (2H, dd, *J* = 1.6, 7.2 Hz); ^{13}C NMR (100 MHz, $CDCl_3$) δ 22.36, 22.44, 22.5, 28.3, 32.7, 37.6, 86.5, 125.7, 127.3, 128.5, 144.1.

3-Cyclohexyl-1,1-diphenylpropyl hydroperoxide (1r)

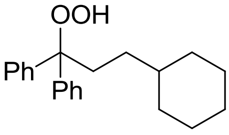

Prepared from 3-cyclohexyl-1,1-diphenylpropan-1-ol (**5r**) for 24 h and purified by flash column chromatography (silica gel, hexane:ethyl acetate = 90:10) in 86% yield; Colorless oil; IR (NaCl) 1032, 1059, 1325, 1447, 1493, 2849, 2922, 3470 (brs) cm^{-1}; ^{1}H NMR (400 MHz, $CDCl_3$) δ 0.78-0.90 (2H, m), 1.07-1.25 (6H, m), 1.58-1.72 (5H, m), 2.40-2.46 (2H, m), 7.10 (1H, s), 7.24 (2H, dd, *J* = 6.0, 7.6 Hz), 7.31 (4H, dd, *J* = 7.2, 7.6 Hz), 7.34 (4H, d, *J* = 7.6 Hz); ^{13}C NMR (100 MHz, $CDCl_3$) δ 26.4, 26.6, 30.6, 33.3, 33.7, 38.0, 89.6, 126.9, 127.2, 128.1, 143.3; ESIHRMS: Found: *m*/*z* 311.2015. Calcd for $C_{21}H_{27}O_2$: $(M + H)^+$ 311.2011.

3-(Adamantan-2-yl)-1,1-diphenylpropyl hydroperoxide (1s)

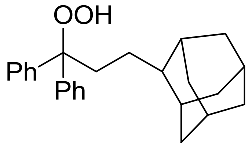

Prepared from 3-(adamantan-2-yl)-1,1-diphenylpropan-1-ol (**5s**) for 8 h and purified by flash column chromatography (silica gel, hexane:ethyl acetate = 90:10) in 91% yield; White solid; mp. 146–148 °C; IR (NaCl) 1337, 1446, 1493, 2849, 2905, 3480 (brs) cm^{-1}; ^{1}H NMR (400 MHz, $CDCl_3$) δ 1.36-1.45 (4H, m), 1.59 (1H, t, J = 7.2 Hz), 1.65-1.75 (9H, m), 1.78.1.88 (3H, m), 2.37-2.43 (2H, m), 7.11 (1H s), 7.24 (2H, tt, J = 1.6, 7.2 Hz), 7.31 (4H, ddd, J = 2.0, 7.2, 7.6 Hz), 7.35-7.39 (4H, m); ^{13}C NMR (100 MHz, $CDCl_3$) δ 25.9, 28.0, 28.3, 31.5, 31.9, 34.4, 38.4, 39.2, 44.6, 89.6, 126.8, 127.2, 128.1, 143.3; ESIHRMS: Found: m/z 363.2332. Calcd for $C_{25}H_{31}O_2$: $(M + H)^+$ 363.2324.

2.4. Preparation of 1,6-diphenylhexyl 3-hydroperoxide (1o)

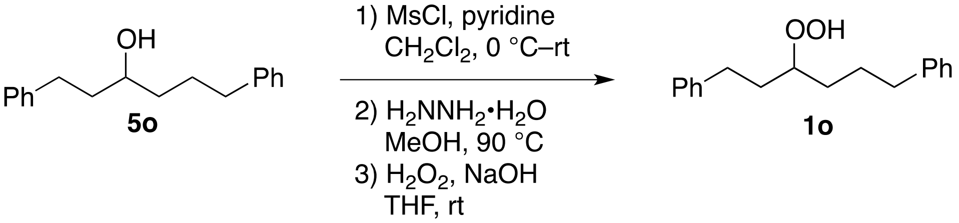

Previously reported procedure from literature [11] for the synthesis of analogous compounds was employed for the synthesis of 1,6-diphenylhexyl 3-hydroperoxide (**1o**). To a solution of 1,6-diphenylhexan-3-ol (**5o**) (1.27 g, 5.0 mmol) in CH_2Cl_2 (2 mL), was added 0.43 mL of methanesulfonyl chloride (0.63 g, 5.5 mmol) and followed by 0.8 mL of pyridine (0.79 g, 10.0 mmol). After completion (2 h), the reaction was quenched with 10% HCl (40 mL). The organic layer was extracted with ether and the combined extracts were washed with saturated $NaHCO_3$, followed by brine and water. After drying over magnesium sulfate, volatile materials were removed in vacuo to afford 1,6-diphenylhexan-3-yl methanesulfonate. The crude product was used directly for the subsequent step without purification. Partial characterization data: ^{1}H NMR (400 MHz, $CDCl_3$) δ 1.68-1.82 (4H, m), 1.92-2.08 (2H, m), 2.58-2.78 (4H, m), 2.94 (3H, s), 4.78 (1H, tt, J = 5.6, 6.0 Hz), 7.13-7.22 (6H, m), 7.22-7.32 (4H, m); ^{13}C NMR (100 MHz, $CDCl_3$) δ 26.5, 31.2, 33.9, 35.4, 36.0, 38.7, 82.9, 126.0, 126.1, 128.3, 128.4 (overlapped), 128.5, 140.8, 141.6. A reaction mixture of methanesulfonate along with hydrazine monohydrate (50–60%; 4.7 mL, 150 mmol) and methanol (8 mL) in sealed tube was heated at 90 ºC for 4 h. After consumption of starting material, the reaction mixture was extracted with ether and washed with KOH (50 wt % aq soln), followed by washing with water and brine. The organic extracts were dried over magnesium sulfate and volatile materials were removed in vacuo to give (1,6-diphenylhexan-3-yl)hydrazine. The crude product was immediately used for the next step without any purification. Partial characterization data: ^{1}H NMR (400 MHz, $CDCl_3$) δ 1.40-1.58 (2H, m), 1.58-1.80 (4H, m), 2.55 (1H, tt, J = 6.0, 6.0 Hz), 2.62 (4H, t, J = 7.6 Hz), 2.89 (2H, brs), 7.14-7.24 (6H, m), 7.24-7.30 (4H, m); ^{13}C NMR (100 MHz, $CDCl_3$) δ

27.3, 31.2, 31.9, 33.5, 36.0, 62.2, 125.75, 125.79, 128.2, 128.28, 128.33, 128.4, 142.1. To a solution of hydrazine in 50 mL of THF, NaOH (0.8 g, 20.0 mmol) was added, followed by hydrogen peroxide (90 mL, 30 wt % aq soln). The reaction mixture was stirred at rt for 3 d. The reaction mixture was extracted with ether, and the combined organic phase was washed with water (100 mL × 5) and brine and dried over magnesium sulfate. Volatile materials were removed in vacuo and the resulting crude material was subjected to flash column chromatography (hexane:ethyl acetate = 90:10) to furnish 1,6-diphenylhexyl 3-hydroperoxide (**1o**) (0.81 g, 3.0 mmol) in 60% yield over 3 steps.

1,6-Diphenylhexyl 3-hydroperoxide (1o)

Colorless oil; IR (NaCl) 1344, 1454, 1495, 1603, 2860, 2940, 3024, 3404 (brs) cm^{-1}; ^{1}H NMR (400 MHz, $CDCl_3$) δ 1.50-1.63 (1H, m), 1.63-1.86 (4H, m), 1.90-2.00 (1H, m), 2.62 (2H, t, *J* = 7.2 Hz), 2.65-2.78 (2H, m), 3.90-3.97 (1H, m), 7.14-7.22 (6H, m), 7.22-7.30 (4H, m), 7.40 (1H, s); ^{13}C NMR (100 MHz, $CDCl_3$) δ 27.0, 31.5, 31.6, 33.7, 35.8, 84.6, 125.78, 125.85, 128.3, 128.4 (overlapped×2), 142.0, 142.2; ESIHRMS: Found: *m*/*z* 271.1698. Calcd for $C_{18}H_{23}O_2$: $(M + H)^+$ 271.1698.

2.5. Preparation of 1,1-diphenylpentane (10)

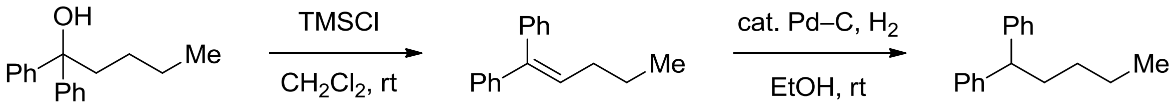

5i **10**

To a stirring solution 1,1-diphenylpentan-1-ol (**5i**) (2.4 g, 10 mmol) in CH_2Cl_2 (20 mL), was added 1.9 mL of $ClSiMe_3$ (1.6 g, 15 mmol). The reaction mixture was allowed to stir for 2 h and then the solvent was removed in vacuo and the crude material was subjected to flash column chromatography (100% hexane) to afford 1,1-diphenylpent-1-ene (2.0 g, 9 mmol) in 90% yield.

1,1-Diphenylpent-1-ene [12]

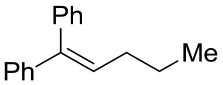

Colorless oil; ^{1}H NMR (400 MHz, $CDCl_3$) δ 0.90 (3H, t, *J* = 7.2 Hz), 1.46 (2H, qt, *J* = 7.2, 7.6 Hz), 2.09 (2H, td, *J* = 7.6, 7.6 Hz), 6.08 (1H, t, *J* = 7.6 Hz), 7.15-7.25 (7H, m), 7.28 (1H, t, *J* = 7.2 Hz), 7.35 (2H, dd, *J* = 6.8, 7.6 Hz); ^{13}C NMR (100 MHz, $CDCl_3$) δ 13.8, 23.1, 31.8, 126.7, 126.8, 127.2, 128.0, 128.1, 129.9, 130.1, 140.3, 141.6, 142.9.

To a 2-neck flask with Pd (10 wt %) (0.19 g, 0.18 mmol) under nitrogen atmosphere, was added a solution of 1,1-diphenylpent-1-ene (1.96 g, 8.8 mmol) in EtOH (44 mL). The reaction mixture was stirred for 6 h and then filtered through a pad of celite. Afterward, solvent was

removed in vacuo, and the crude material was subjected to flash column chromatography (100% hexane) to afford 1,1-diphenylpentane (**10**) in quantitative yield.

1,1-Diphenylpentane (10)

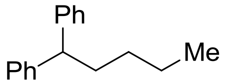

Colorless oil; IR (NaCl) 1450, 1493, 1599, 2857, 2928, 2955, 3024, 3059 cm^{-1}; ^{1}H NMR (400 MHz, $CDCl_3$) δ 0.85 (3H, t, J = 7.2 Hz), 1.18-1.28 (2H, m), 1,32 (2H, tt, J = 7.2, 7.4 Hz), 2.03 (2H, td, J= 7.2, 8.0 Hz), 3.87 (1H, t, J = 8.0 Hz), 7.14 (2H, t, J = 6.8 Hz), 7.20-7.28 (8H, m); ^{13}C NMR (100 MHz, $CDCl_3$) δ 14.0, 22.7, 30.2, 35.4, 51.4, 126.0, 127.9, 128.3, 145.4; ESIHRMS: Found: m/z 225.1632. Calcd for $C_{17}H_{21}$: $(M + H)^+$ 225.1643.

3. Copper-catalyzed aerobic aliphatic C–H oxygenation with hydroperoxides

3.1. A typical procedure for the reaction of hydroperoxide 1a (Table 1, entry 14)

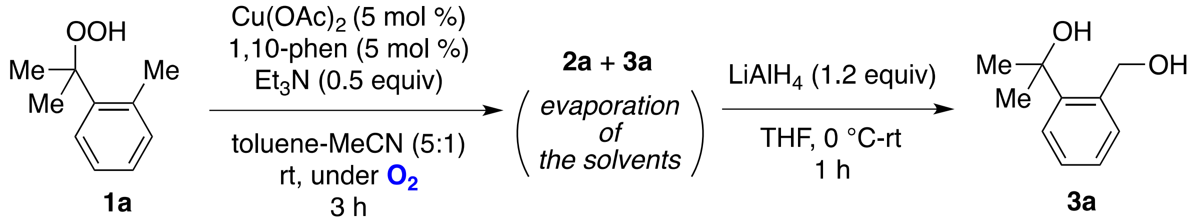

To a stirring solution of $Cu(OAc)_2$ (4.5 mg, 0.025 mmol), 1,10-phenanthroline (4.5 mg, 0.025 mmol) and hydroperoxide **1a** (83.1 mg, 0.50 mmol) in 5.0 mL of solvent (toluene:MeCN = 5:1), were added NEt_3 (35 μL, 0.25 mmol) and the reaction mixture was stirred at room temperature under an oxygen atmosphere for 3 h. After the complete consumption of hydroperoxide **1a**, the solvent was removed in vacuo. The crude residue was subjected to $LiAlH_4$ (22.8 mg, 0.6 mmol) at 0 °C in THF. The reaction was stirred at room temperature for another 1 h before quenching with pH 9 buffer at 0 °C and the organic materials were extracted three times with diethyl ether. The combined extracts were washed with brine, and dried over $MgSO_4$. Volatile materials were removed in vacuo and the resulting crude material was subjected to flash column chromatography (hexane:ethyl acetate = 60:40) to afford 2-(2-(hydroxymethyl)phenyl) propan-2-ol (**3a**) (75.0 mg, 0.45 mmol) in 90% yield.

3,3-Dimethyl-1,3-dihydroisobenzofuran-1-ol (2a) [13]

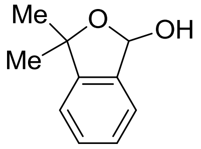

White solid; ^{1}H NMR (400 MHz, $CDCl_3$) δ 1.50 (3H, s), 1.61 (3H, s), 3.65 (1H, d, J = 6.8 Hz), 6.44 (1H, d, J = 6.8 Hz), 7.17 (1H, d, J = 7.2 Hz), 7.31-7.42 (3H, m); ^{13}C NMR (100 MHz, $CDCl_3$) δ 29.0, 30.9, 85.8, 99.7, 120.5, 123.0, 127.9, 129.4, 138.4, 147.5.

2-(2-(Hydroxymethyl)phenyl)propan-2-ol (3a) [14]

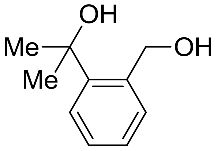

Colorless oil; ^{1}H NMR (400 MHz, $CDCl_3$) δ 1.64 (6H, s), 3.97 (2H, brs), 4.76 (2H, s), 7.17-7.30 (4H, m); ^{13}C NMR (100 MHz, $CDCl_3$) δ 32.4, 65.3, 74.5, 126.2, 127.2, 127.9, 132.0, 137.9, 146.3.

1,1-Dimethyl-1,3-dihydroisobenzofuran (4) [15]

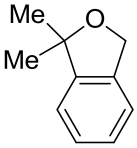

Colorless oil; ^{1}H NMR (400 MHz, $CDCl_3$) δ 1.50 (6H, s), 5.07 (2H, s), 7.10-7.15 (1H, m), 7.17-7.21 (1H, m), 7.22-7.29 (2H, m); ^{13}C NMR (100 MHz, $CDCl_3$) δ 28.4, 70.7, 85.7, 120.5, 121.0, 127.1, 127.3, 138.5, 146.9.

Compound data in Table 2

1,1,4-Triphenylbutane-1,4-diol (3b)

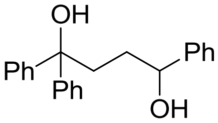

White solid; mp. 97–99 °C; IR (NaCl) 1449, 1493, 2872, 2929, 2955, 3404 (brs) cm^{-1}; ^{1}H NMR (400 MHz, $CDCl_3$) δ 1.56 (3H, s), 1.80 (2H, dt, *J* = 7.2, 7.6 Hz), 2.21 (1H, brs), 2.35-2.52 (2H, m), 2.86 (1H, brs), 4.72 (1H, t, *J* = 6.0 Hz), 7.18-7.24 (2H, m), 7.24-7.35 (9H, m), 7.36-7.42 (4H, m); ^{13}C NMR (100 MHz, $CDCl_3$) δ 33.3, 37.9, 74.5, 77.9, 125.8, 126.0, 126.1, 126.66, 126.72, 127.4, 128.07, 128.09, 128.4, 144.4, 146.8, 147.1; ESIHRMS: Found: *m*/*z* 319.1709. Calcd for $C_{22}H_{23}O_2$: $(M + H)^+$ 319.1698.

1,4-Diphenylpentane-1,4-diol (3c)

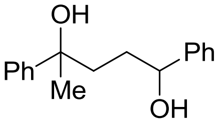

A mixture of 2 diastereomers in 0.5:0.5 (1:1) ratio

Yellowish oil; IR (NaCl) 1028, 1057, 1454, 1493, 2930, 2972, 3362 (brs) cm^{-1}; ^{1}H NMR (400 MHz, $CDCl_3$) δ 1.55 (3H×1.0, s), 1.60-1.88 (2.5H×1.0, m), 1.94-2.05 (1.5H×1.0, m), 2.45 (2H×1.0, brs), 4.60-4.69 (1H×1.0, m), 7.20-7.36 (8H×1.0, m), 7.38-7.43 (2H×1.0, m); ^{13}C NMR (100 MHz, $CDCl_3$) δ 30.2, 30.9, 33.4, 33.6, 39.8, 40.4, 74.2, 74.3, 74.4, 74.7, 124.7, 124.8, 125.8 (overlapped), 126.35, 126.45, 127.3, 127.4, 128.08, 128.10, 128.3 (overlapped), 144.5, 144.6, 147.6, 147.8; ESIHRMS: Found: *m*/*z* 257.1549. Calcd for $C_{17}H_{21}O_2$: $(M + H)^+$ 257.1542.

1,4-Diphenylhexane-1,4-diol (3d)

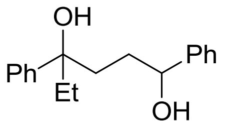

A mixture of 2 diastereomers in 0.5:0.5 (1:1) ratio

Yellowish oil; IR (NaCl) 1028, 1057, 1309, 1454, 1493, 1603, 2934, 2967, 3362 (brs) cm^{-1}; ^{1}H NMR (400 MHz, $CDCl_3$) δ 0.70 (3H×0.5, t, *J* = 7.6 Hz), 0.72 (3H×0.5, t, *J* = 7.2 Hz), 1.48-1.87 (4.5H×1.0, m), 1.92-2.02 (1.5H×1.0, m), 2.82 (2H×1.0, brs), 4.56 (1H×0.5, dd, *J* = 4.4, 8.8 Hz), 4.63 (1H×0.5, dd, *J* = 4.8, 7.2 Hz), 7.16-7.35 (10H×1.0, m); ^{13}C NMR (100 MHz, $CDCl_3$) δ 7.7, 7.8, 32.9, 33.3, 35.6, 36.1, 38.0, 39.1, 74.1, 75.0, 76.89, 76.93, 125.4, 125.5, 125.7, 125.8, 126.2, 126.3, 127.3, 127.4, 127.97, 128.01, 128.3 (overlapped), 144.5, 144.6, 145.7 (overlapped); ESIHRMS: Found: *m*/*z* 271.1693. Calcd for $C_{18}H_{23}O_2$: $(M + H)^+$ 271.1698.

4-(Naphthalen-2-yl)-1-phenylpentane-1,4-diol (3e)

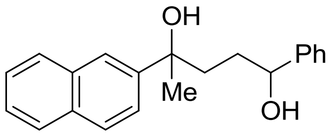

A mixture of 2 diastereomers in 0.5:0.5 (1:1) ratio

Yellowish oil; IR (NaCl) 820, 858, 1055, 1273, 1375, 1452, 1504, 2974, 3011, 3362 (brs) cm^{-1}; ^{1}H NMR (400 MHz, $CDCl_3$) δ 1.62 (3H×1.0, s), 1.57-1.67 (1H×0.5, m), 1.67-1.81 (3H×0.5, m), 1.91 (1H×0.5, ddd, *J* = 5.6, 9.2, 14.8 Hz), 2.03-2.16 (3H×0.5, m), 2.66 (2H×1.0, brs), 4,63 (1H×0.5, dd, *J* = 4.4, 8.4 Hz), 4.66 (1H×0.5, dd, *J* = 4.8, 7.2 Hz), 7.20-7.32 (5H×1.0, m), 7.42-7.50 (3H×1.0, m), 7.77-7.84 (3H×1.0, m), 7.89 (1H×1.0, s); ^{13}C NMR (100 MHz, $CDCl_3$) δ 30.3, 31.0, 33.4, 33.6, 39.5, 40.2, 74.1, 74.48, 74.53, 74.8, 123.1, 123.3, 123.61, 123.63, 125.5, 125.6, 125.73, 125.74, 125.91, 125.95, 127.3, 127.4 (overlapped), 127.77, 127.82, 128.1, 128.29, 128.31, 132.09, 132.14, 133.11, 133.13, 144.4, 144.5, 145.0, 145.2; ESIHRMS: Found: *m*/*z* 307.1702. Calcd for $C_{21}H_{23}O_2$: $(M + H)^+$ 307.1698.

4-(4-Bromophenyl)-1-phenylpentane-1,4-diol (3f)

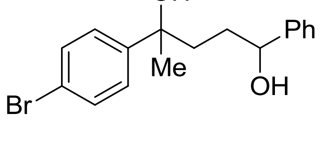

A mixture of 2 diastereomers in 0.5:0.5 (1:1) ratio

Yellowish oil; IR (NaCl) 827, 1009, 1078, 1393, 1454, 1487, 1589, 2930, 2972, 3345 (brs) cm^{-1}; ^{1}H NMR (400 MHz, $CDCl_3$) δ 1.50 (3H×1.0, s), 1.53-1.84 (5H×0.5, m), 1.90-2.00 (3H×0.5, m), 2.69 (2H×1.0, brs), 4.61 (1H×0.5, dd, *J* = 4.4, 8.4 Hz), 4.65 (1H×0.5, dd, *J* = 4.8, 7.2 Hz), 7.21-7.35 (7H×1.0, m), 7.40-7.45 (2H×1.0, m); ^{13}C NMR (100 MHz, $CDCl_3$) δ 30.2, 31.0, 33.2, 33.5, 39.6, 40.4, 74.0, 74.1 (overlapped), 74.7, 120.2, 120.3, 125.68, 125.71, 126.7, 126.9, 127.4, 127.5, 128.4

(overlapped), 131.09, 131.11, 144.2, 144.4, 146.7, 147.0; ESIHRMS: Found: *m*/*z* 335.0641. Calcd for $C_{17}H_{20}O_2{}^{79}B$: $(M + H)^+$ 335.0647.

1,4,6-Triphenylhexane-1,4-diol (3g)

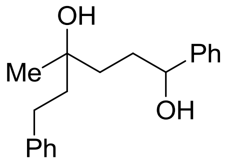

A mixture of 2 diastereomers in 0.5:0.5 (1:1) ratio

Yellowish oil; IR (NaCl) 1057, 1373, 1454, 1495, 2864, 2945, 3026, 3350 (brs) cm^{-1}; ^1H NMR (400 MHz, $CDCl_3$) 1.20 (3H×0.5, s), 1.21 (3H×0.5, s), 1.47-1.57 (1H×1.0, m), 1.63-1.93 (5H×1.0, m), 2.40-2.98 (4H×1.0, m), 4.64 (1H×0.5, t, *J* = 7.6 Hz), 4.65 (1H×0.5, t, *J* = 7.6 Hz), 7.13-7.19 (3H×1.0, m), 7.22-7.28 (3H×1.0, m), 7.30-7.35 (4H×1.0, m); ^{13}C NMR (100 MHz, $CDCl_3$) δ 26.6, 26.9, 30.26, 30.33, 33.4, 33.5, 37.8, 37.9, 43.6, 44.2, 72.28, 72.32, 74.6, 74.8, 125.7, 125.8, 127.42, 127.43, 128.27, 128.35 (overlapped), 128.39, 142.40, 142.44, 144.70, 144.74; ESIHRMS: Found: *m*/*z* 285.1854. Calcd for $C_{19}H_{25}O_2$: $(M + H)^+$ 285.1855.

1-(3-Hydroxy-3-phenylpropyl)cyclohexanol (3h) [16]

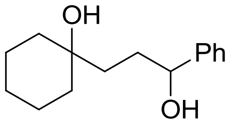

White solid; ^1H NMR (400 MHz, $CDCl_3$) δ 1.19-1.30 (1H, m), 1.32-1.65 (11H, m), 1.75-1.92 (2H, m), 2.65 (2H, brs), 4.65 (1H, dd, *J* = 4.8, 8.0 Hz), 7.22-7.28 (1H, m), 7.29-7.36 (4H, m); ^{13}C NMR (100 MHz, $CDCl_3$) δ 22.1, 22.2, 25.7, 32.6, 37.1, 37.7, 38.1, 71.1, 74.7, 125.8, 127.3, 128.3, 144.9.

1,1-Diphenylpentane-1,4-diol (3i)

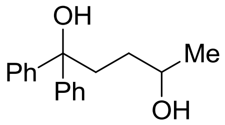

White solid; mp. 77–79 °C; IR (NaCl) 988, 1032, 1061, 1377, 1447, 1493, 1599, 2967, 3011, 3412 (brs) cm^{-1}; ^1H NMR (400 MHz, $CDCl_3$) δ 1.17 (3H, d, *J* = 6.0 Hz), 1.39-1.58 (2H, m), 1.59 (2H, brs), 2.44 (2H, t, *J* = 7.6 Hz), 3.81-3.89 (1H, m), 7.19-7.24 (2H, m), 7.28-7.34 (4H, m), 7.43 (4H, d, *J* = 8.0 Hz); ^{13}C NMR (100 MHz, $CDCl_3$) δ 23.6, 33.2, 38.0, 68.3, 77.8, 126.0, 126.1, 126.6, 126.7, 128.0, 147.0, 147.3; ESIHRMS: Found: *m*/*z* 257.1544. Calcd for $C_{17}H_{21}O_2$: $(M + H)^+$ 257.1542.

2-Phenylhexane-2,5-diol (3j)

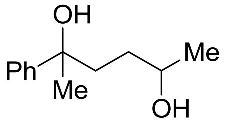

A mixture of 2 diastereomers in 0.56:0.44 (1.27:1) ratio

Yellowish oil; IR (NaCl) 1028, 1061, 1128, 1375, 1447, 1495, 2930, 2970, 3374 (brs) cm^{-1}; ^{1}H NMR (400 MHz, $CDCl_3$) δ 1.14 (3H×0.56, d, *J* = 6.0 Hz), 1.16 (3H×0.44, d, *J* = 6.4 Hz), 1.30-1.52 (2H×1.0, m), 1.56 (3H×0.56, s), 1.58 (3H×0.44, s), 1.84-2.05 (2H×1.0, m), 3.73-3.83 (1H×1.0, m), 7.24 (1H×1.0, t, *J* = 6.8 Hz), 7.34 (2H×1.0, ddd, *J* = 2.0, 7.6, 8.0 Hz), 7.41-7.47 (2H×1.0, m); ^{13}C NMR (100 MHz, $CDCl_3$) δ 23.3, 23.7, 30.3, 31.0, 33.3, 33.4, 39.8, 40.6, 67.9, 68.5, 74.3, 74.4, 124.8, 124.9, 126.3, 126.4, 128.08, 128.09, 147.8, 148.1; ESIHRMS: Found: *m*/*z* 195.1384. Calcd for $C_{12}H_{19}O_2$: $(M + H)^+$ 195.1385.

5-Methyl-7-phenylheptane-2,5-diol (3k)

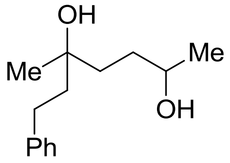

A mixture of 2 diastereomers in 0.5:0.5 (1:1) ratio

Yellowish oil; IR (NaCl) 1053, 1070, 1375, 1454, 1495, 1603, 2934, 2968, 3362 (brs) cm^{-1}; ^{1}H NMR (400 MHz, $CDCl_3$) δ 1.215 (3H×0.5, d, *J* = 6.4 Hz), 1.218 (3H×0.5, d, *J* = 6.4 Hz), 1.25 (3H×1.0, s), 1.52-1.70 (4H×1.0, m), 1.75-1.82 (2H×1.0, m), 2.37 (2H×1.0, brs), 2.64-2.72 (2H×1.0, m), 3.77-3.86 (1H×1.0, m), 7.16-7.22 (3H×1.0, m), 7.25-7.30 (2H×1.0, m); ^{13}C NMR (100 MHz, $CDCl_3$) δ 23.6, 23.7, 26.6, 27.0, 30.35, 30.41, 33.2, 33.3, 37.9, 38.0, 43.7, 44.3, 68.4, 68.5, 72.3, 72.4, 125.7 (overlapped), 128.3 (overlapped), 128.4 (overlapped), 142.4, 142.5; ESIHRMS: Found: *m*/*z* 223.1701. Calcd for $C_{14}H_{23}O_2$: $(M + H)^+$ 223.1698.

1-(3-Hydroxybutyl)cyclohexanol (3l) [17]

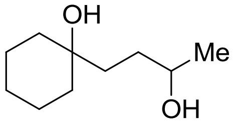

Yellowish solid; ^{1}H NMR (400 MHz, $CDCl_3$) δ 1.20 (3H, d, *J* = 6.4 Hz), 1.23-1.35 (1H, m), 1.38-1.62 (13H, m), 3.76-3.85 (1H, m); ^{13}C NMR (100 MHz, $CDCl_3$) δ 22.2, 22.3, 23.6, 25.8, 32.4, 37.2, 37.9, 38.1, 68.5, 71.2.

2-Phenyldecane-2,5-diol (3m)

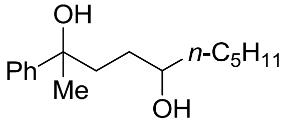

A mixture of 2 diastereomers in 0.56:0.44 (1.27:1) ratio

White solid; mp. 50–53 °C; IR (NaCl) 1028, 1063, 1375, 1445, 1495, 2857, 2930, 3331 (brs) cm^{-1}; ^{1}H NMR (400 MHz, $CDCl_3$) δ 0.86 (3H×0.56, t, *J* = 6.8 Hz), 0.87 (3H×0.44, t, *J* = 6.8 Hz), 1.18-1.52 (10H×1.0, m), 1.55 (3H×0.56, s), 1.56 (3H×0.44, s), 1.83-2.07 (2H×1.0, m), 2.69 (2H×1.0, brs), 3.50-3.60 (1H×1.0, m), 7.19-7.25 (1H×1.0, m), 7.33 (2H×1.0, dd, *J* = 7.6, 8.0 Hz), 7.40-7.45 (2H×1.0, m); ^{13}C NMR (100 MHz, $CDCl_3$) δ 14.0, 22.6, 25.3, 25.4, 30.5, 31.1, 31.5, 31.6,

31.77, 31.79, 37.2, 37.6, 39.8, 40.6, 71.9, 72.6, 74.4 (overlapped), 124.8, 124.9, 126.3, 126.4, 128.1 (overlapped), 148.0, 148.1; ESIHRMS: Found: *m*/*z* 251.2013. Calcd for $C_{16}H_{27}O_2$: $(M + H)^+$ 251.2011.

***trans*-2-(2-Hydroxy-2,2-diphenylethyl)cyclohexanol (3n-major)**

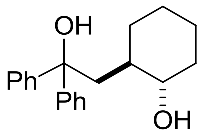

The NMR reported is the major isomer of compound **3n** (*trans*:*cis* = 1.37:1). White solid; mp. 143–144 °C; IR (NaCl) 1003, 1061, 1447, 1493, 2857, 2930, 3331 (brs) cm^{-1}; ^1H NMR (400 MHz, $CDCl_3$) δ 0.90-1.03 (1H, m), 1.04-1.24 (3H, m), 1.25-1.35 (1H, m), 1.48-1.68 (3H, m), 1.83-1.90 (1H, m), 2.35 (1H, dd, *J* = 6.0, 14.8 Hz), 2.41 (1H, dd, *J* = 2.4, 14.8 Hz), 2.84 (1H, brs), 3.23 (1H, dt, *J* = 4.0, 10.0 Hz), 5.57 (1H, brs), 7.14-7.23 (2H, m), 7.26 (2H, dd, *J* = 7.2, 8.0 Hz), 7.32 (2H, t, *J* = 7.6 Hz), 7.42 (2H, dd, *J* = 1.2, 8.8 Hz), 7.47 (2H, dd, J = 1.2, 8.4 Hz); ^{13}C NMR (100 MHz, $CDCl_3$) δ 24.7, 25.5, 34.3, 35.8, 40.6, 48.4, 76.1, 77.1, 125.9, 126.3, 126.4, 126.6, 127.9, 128.0, 146.7, 149.1; ESIHRMS: Found: *m*/*z* 297.1861. Calcd for $C_{20}H_{25}O_2$: $(M + H)^+$ 297.1855.

Compound Data in Scheme 5

4-Hydroxy-1,6-diphenylhexan-1-one (2o)

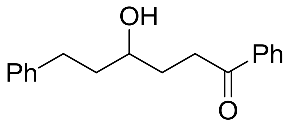

White solid; mp. 77–78 °C; IR (NaCl) 1449, 1495, 1599, 1682, 2928, 3017, 3063, 3445 (brs) cm^{-1}; ^1H NMR (400 MHz, $CDCl_3$) δ 1.78-1.92 (3H, m), 1.96-2.05 (2H, m), 2.70 (1H, td, *J* = 8.0, 16.0 Hz), 2.82 (1H, td, *J* = 8.0, 13.6 Hz), 3.13 (1H, td, *J* = 7.2, 17.6 Hz), 3.18 (1H, td, *J* = 7.2, 17.2 Hz), 3.67-3.75 (1H, m), 7.18 (1H, t, *J* = 7.2 Hz), 7.21 (2H, d, *J* = 6.8 Hz), 7.26-7.31 (2H, m), 7.45 (2H, dd, *J* = 7.2, 8.0 Hz), 7.56 (1H, tt, *J* = 1.2, 7.6 Hz), 7.97 (2H, dd, *J* = 1.6, 8.0 Hz); ^{13}C NMR (100 MHz, $CDCl_3$) δ 31.4, 32.1, 34.9, 39.5, 70.9, 125.8, 128.1, 128.40, 128.41, 128.6, 133.1, 136.8, 141.9; ESIHRMS: Found: *m*/*z* 269.1542. Calcd for $C_{18}H_{21}O_2$: $(M + H)^+$ 269.1542.

1,6-Diphenylhexan-3-one (8o)

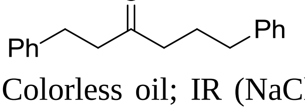

Colorless oil; IR (NaCl) 1369, 1452, 1497, 1603, 1699, 2859, 2928, 3024, 3059 cm^{-1}; ^1H NMR (400 MHz, $CDCl_3$) δ 1.89 (2H, tt, *J* = 7.2, 7.6 Hz), 2.38 (2H, t, *J* = 7.2 Hz), 2.58 (2H, t, *J* = 7.6 Hz), 2.69 (2H, t, *J* = 7.6 Hz), 2.87 (2H, t, *J* = 7.6 Hz), 7.11-7.20 (6H, m), 7.26 (4H, dd, *J* = 7.2, 7.6 Hz), ; ^{13}C NMR (100 MHz, $CDCl_3$) δ 25.1, 29.7, 35.0, 42.0, 44.3, 125.9, 126.0, 128.27, 128.33, 128.4 (overlapped), 141.1, 141.5, 209.8; ESIHRMS: Found: *m*/*z* 253.1591. Calcd for $C_{18}H_{21}O$: $(M + H)^+$ 253.1592.

Compound Data in Table 3

Reactions were carried out using 0.5 mmol of hydroperoxides **1** with $Cu(OAc)_2$ (5 mol %), 1,10-phen (5 mol %), and Et_3N (0.5 equiv) in toluene/MeCN (5:1, 0.1 M) at room temperature under an O_2 atmosphere. After stirring 5–7 h, the reaction mixture was further treated with PPh_3 (1 equiv) at rt.

4-Methyl-1,1-diphenylpentane-1,4-diol (3p)

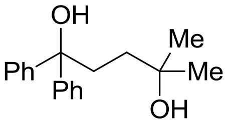

White solid; mp. 128–131 °C; IR (NaCl) 702, 1026, 1049, 1371, 1446, 1493, 2932, 2972, 3370 (brs) cm^{-1}; ^1H NMR (400 MHz, $CDCl_3$) δ 1.18 (6H, s), 1.48 (2H, t, *J* = 7.6 Hz), 1.92 (1H, brs), 2.40 (2H, t, *J* = 7.6 Hz), 3.42 (1H, brs), 7.19 (2H, t, *J* = 7.2 Hz), 7.28 (4H, t, *J* = 7.6 Hz), 7.41 (4H, dd, *J* = 1.2, 7.6 Hz); ^{13}C NMR (100 MHz, $CDCl_3$) δ 29.4, 36.1, 37.3, 70.8, 77.8, 126.0, 126.6, 128.1, 147.2; ESIHRMS: Found: *m*/*z* 271.1693. Calcd for $C_{18}H_{23}O_2$: $(M + H)^+$ 271.1698.

2-Methyl-5-phenylhexane-2,5-diol (3q)

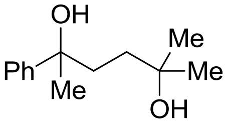

White solid; mp. 63–65 °C; IR (NaCl) 1067, 1375, 1445, 1493, 2932, 2974, 3393 (brs), 3599 cm^{-1}; ^1H NMR (400 MHz, $CDCl_3$) δ 1.16 (3H, s), 1.18 (3H, s), 1.34 (1H, ddd, *J* = 6.4, 9.2, 15.6 Hz), 1.46 (1H, ddd, *J* = 6.0, 9.2, 15.6 Hz), 1.55 (3H, s), 1.91 (1H, ddd, *J* = 6.0, 9.2, 15.6 Hz), 1.97 (1H, ddd, *J* = 6.4, 9.2, 15.6 Hz), 2.29 (2H, brs), 7.22 (1H, t, *J* = 7.2 Hz), 7.33 (2H, ddd, *J* = 1.6, 7.2, 8.0 Hz), 7.43 (2H, dd, *J* = 1.2, 8.8 Hz); ^{13}C NMR (100 MHz, $CDCl_3$) δ 29.0, 29.8, 30.7, 37.5, 38.2, 70.7, 74.3, 124.8, 126.4, 128.1, 148.0; ESIHRMS: Found: *m*/*z* 209.1544. Calcd for $C_{13}H_{21}O_2$: $(M + H)^+$ 209.1542.

1-(3-Hydroxy-3,3-diphenylpropyl)cyclohexanol (3r)

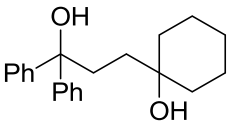

White solid; mp. 87–90 °C; IR (NaCl) 1007, 1059, 1260, 1449, 1491, 2857, 2934, 3393 (brs) cm^{-1}; ^1H NMR (400 MHz, $CDCl_3$) δ 1.18-1.30 (1H, m), 1.30-1.58 (11H, m), 1.64 (1H, brs), 2.42 (2H, t, *J* = 7.2 Hz), 3.34 (1H, brs), 7.20 (2H, t, *J* = 7.2 Hz), 7.30 (4H, ddd, *J* = 1.6, 7.2, 8.0 Hz), 7.44 (4H, dd, *J* = 1.6, 8.8 Hz); ^{13}C NMR (100 MHz, $CDCl_3$) δ 22.2, 25.7, 34.8, 35.6, 37.5, 71.5, 77.7, 126.0, 126.5, 128.0, 147.3; ESIHRMS: Found: *m*/*z* 311.2003. Calcd for $C_{21}H_{27}O_2$: $(M + H)^+$ 311.2011.

2-(3-Hydroxy-3,3-diphenylpropyl)adamantan-2-ol (3s)

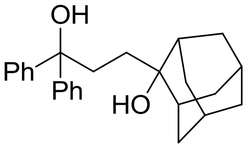

White solid; mp. 47–50 °C; IR (NaCl) 700, 980, 1032, 1449, 2857, 2911, 3447 (brs) cm^{-1}; ^1H NMR (400 MHz, $CDCl_3$) δ 1.50-1.83 (16H, m), 2.10 (2H, d, J = 12.0 Hz), 2.41 (2H, t, J = 7.2 Hz), 7.20 (2H, t, J = 7.2 Hz), 7.30 (4H, dd, J = 7.2, 8.0 Hz), 7.45 (4H, d, J = 7.6 Hz); ^{13}C NMR (100 MHz, $CDCl_3$) δ 27.1, 27.3, 32.1, 32.9, 34.46, 34.51, 36.9, 38.2, 75.2, 77.9, 126.1, 126.6, 128.1, 147.4; ESIHRMS: Found: m/z 363.2321. Calcd for $C_{25}H_{31}O_2$: $(M + H)^+$ 363.2324.

3.2. A procedure for the reaction of alkane 10 under CuCl-NHPI catalytic system (Scheme 7)

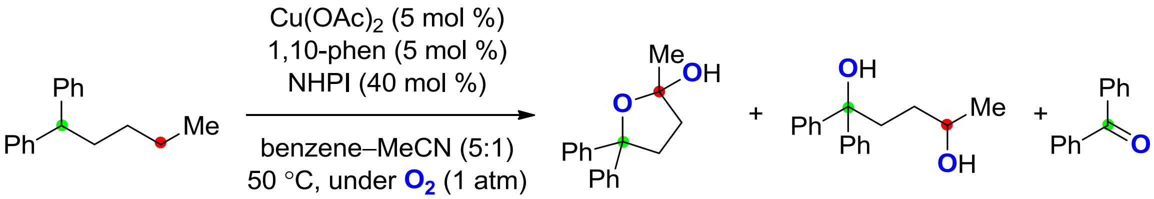

A solution of alkane **10** (67.3 mg, 0.30 mmol) in 3.0 mL of solvent (toluene:MeCN = 5:1) was added to a mixture of CuCl (5.9 mg, 0.060 mmol), 1,10-phenanthroline (10.8 mg, 0.060 mmol) and *N*-hydroxyphthalimide (NHPI) (19.6 mg, 0.12 mmol) at room temperature under an oxygen atmosphere, then heated to 50 °C and left to be stirred for 24 h. The reaction was then allowed to cool to room temperature before quenching with pH 9 buffer and the organic materials were extracted three times with ethyl acetate. The combined extracts were washed with brine, and dried over $MgSO_4$. Volatile materials were removed in vacuo and the resulting crude material was subjected to flash column chromatography (hexane:ethyl acetate = 70:30) to afford 5-hydroxy-5,5-diphenylpentan-2-one (**2i**) (31.2 mg, 0.123 mmol), 1,1-diphenylpentane-1,4-diol (**3i**) (3.8 mg, 0.015 mmol) and benzophenone (**7b**) (13.1 mg, 0.072 mmol) in 41%, 5%, and 24% yields respectively.

2i: Equilibrium mixture of 2-methyl-5,5-diphenyltetrahydrofuran-2-ol (cyclic) and 5-hydroxy-5,5-diphenylpentan-2-one (acyclic) [18]

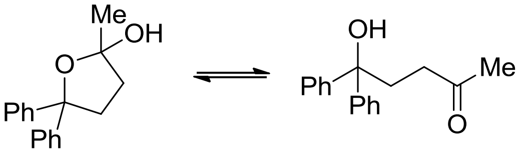

cyclic:acyclic = 1:1

A mixture of 2 equilibrium acyclic and cyclic isomers in 0.5:0.5 (1:1) ratio
White solid; ^1H NMR (400 MHz, $CDCl_3$) δ 1.64 (3H×0.5, s), 1.89 (1H×0.5, dt, J = 6.8, 11.6 Hz), 2.08 (3H×0.5, s), 2.05-2.12 (1H×0.5, m), 2.40 (1H×0.5, s), 2.47 (2H×0.5, t, J = 7.2 Hz), 2.59 (2H×0.5, t, J = 7.2 Hz), 2.66 (1H×0.5, ddd, J = 2.8, 7.2, 12.4 Hz), 2.76 (1H×0.5, s), 2.87 (1H×0.5, dt, J = 6.8, 11.6 Hz), 7.15-7.36 (12H×0.5, m), 7.38-7.46 (8H×0.5, m); ^{13}C NMR (100 MHz, $CDCl_3$)

δ 27.7, 30.1, 35.0, 38.0, 38.4, 38.6, 77.4, 89.2, 105.7, 125.6, 125.9 (overlapped), 126.1, 126.6, 126.8, 126.9 (overlapped), 128.0, 128.1, 128.2 (overlapped), 146.3, 146.7 (overlapped), 147.2, 209.9.

References:

[1] Yamamoto, Y.; Hasegawa, H.; Yamataka, H. *J. Org. Chem.* 2011, *76*, 4652.

[2] Vitale, A. A.; Doctorovich, F.; Nudelman, N. S. *J. Organomet. Chem.* **1987**, *332*, 9.

[3] Eisch, J. J.; Sohn, J. U.; Rabinowitz, E. J. *Eur. J. Org. Chem.* **2010**, 2971.

[4] Zong, H.; Huang, H.; Liu, J.; Bian, G.; Song, L. *J. Org. Chem.* **2012**, *77*, 4645.

[5] Evans, W. J.; Workman, P. S.; Allen, N. T. *Org. Lett.* **2003**, *5*, 2041.

[6] Hata, S.; Yano, Y.; Matsuda, H.; Matsuda, S. *Kogyo Kagaku Zasshi* **1968**, *71*, 704.

[7] Kropf, H.; Von Wallis, H. *Liebigs Ann. Chem.* **1983**, 610.

[8] Hsu, Y. F. ; Cheng, C. P. *J. Mol. Catal. A: Chem.* **1997**, *120*, 109.

[9] Kropf, H.; Von Wallis, H. *Synthesis* **1981**, 633.

[10] Driver, T. G.; Harris, J. R.; Woerpel, K. A. *J. Am. Chem. Soc.* **2007**, *129*, 3836.

[11] Kundu, R.; Ball, Z. T. *Org. Lett.* **2010**, *12*, 2460.

[12] Firouzabadi, H.; Iranpoor, N.; Hazarkhani, H.; Karimi, B. *Synthetic Commun.* **2003**, *33*, 3653.

[13] Horaguchi, T.; Tsukada, C.; Hasegawa, E.; Shimizu, T.; Suzuki, T.; Tanemura, K. *J. Heterocycl. Chem.* **1991**, *28*, 1261.

[14] Ito, M.; Osaku, A.; Shiibashi, A.; Ikariya, T. *Org. Lett.* **2007**, *9*, 1821.

[15] Parham, W. E.; Sayed, Y. A. *Synthesis* **1976**, 116.

[16] Foubelo, F.; Gutierrez, A.; Yus, M. *Synthesis* **1999**, 503.

[17] Fleming, F. F.; Gudipati, S.; Vu, V. A.; Mycka, R. J.; Knochel, P. *Org. Lett.* **2007**, *9*, 4507.

[18] Fujioka, H.; Yahata, K.; Kubo, O.; Sawama, Y.; Hamada, T.; Maegawa, T. *Angew. Chem. Int. Ed.* **2011**, *50*, 12232.

4. ^{1}H and ^{13}C NMR spectrum of new compounds

^{1}H NMR spectrum of **1b** (400 MHz, $CDCl_3$)

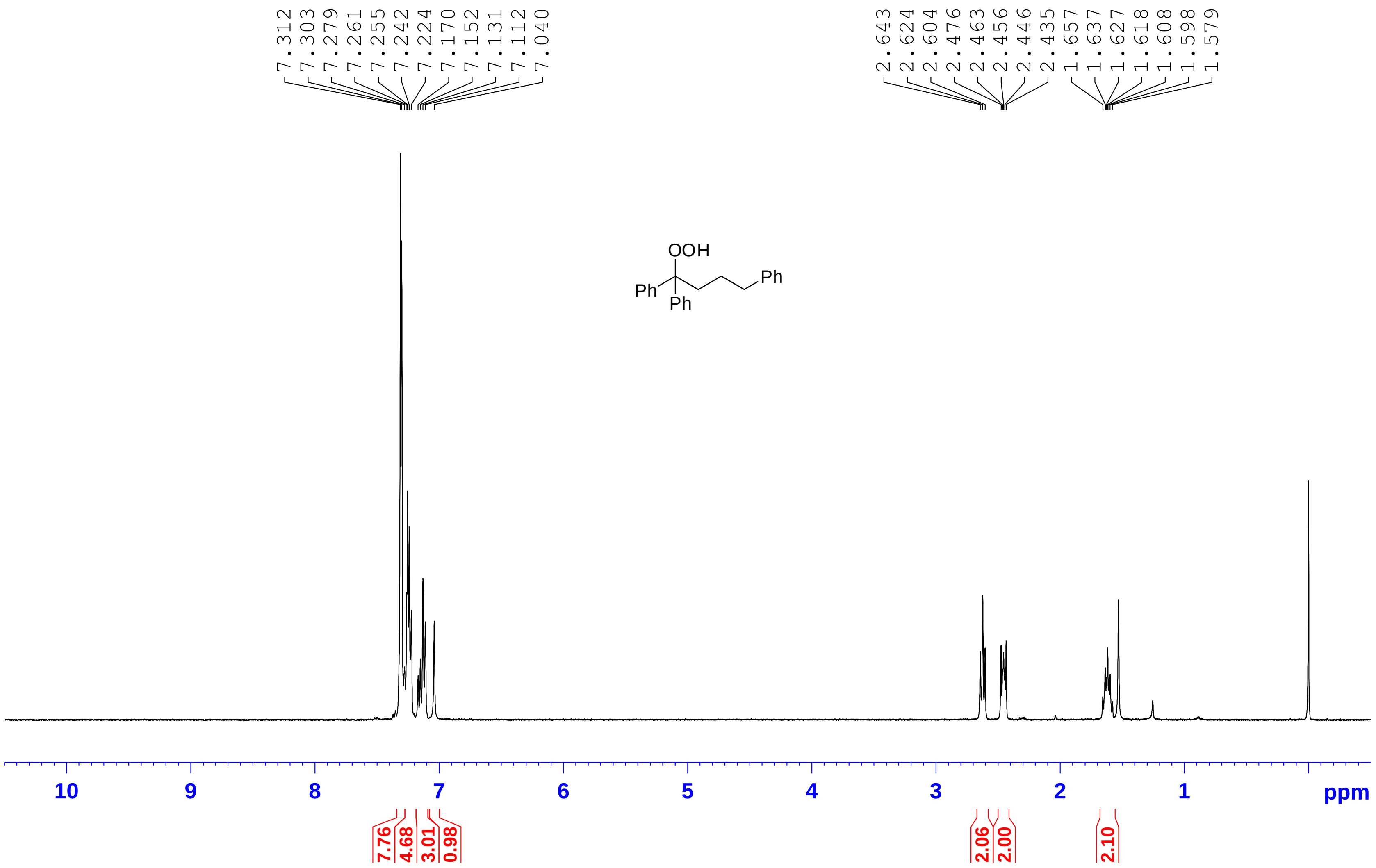

4. 1H and 13C NMR spectrum of new compounds

^{13}C NMR spectrum of **1b** (100 MHz, $CDCl_3$)

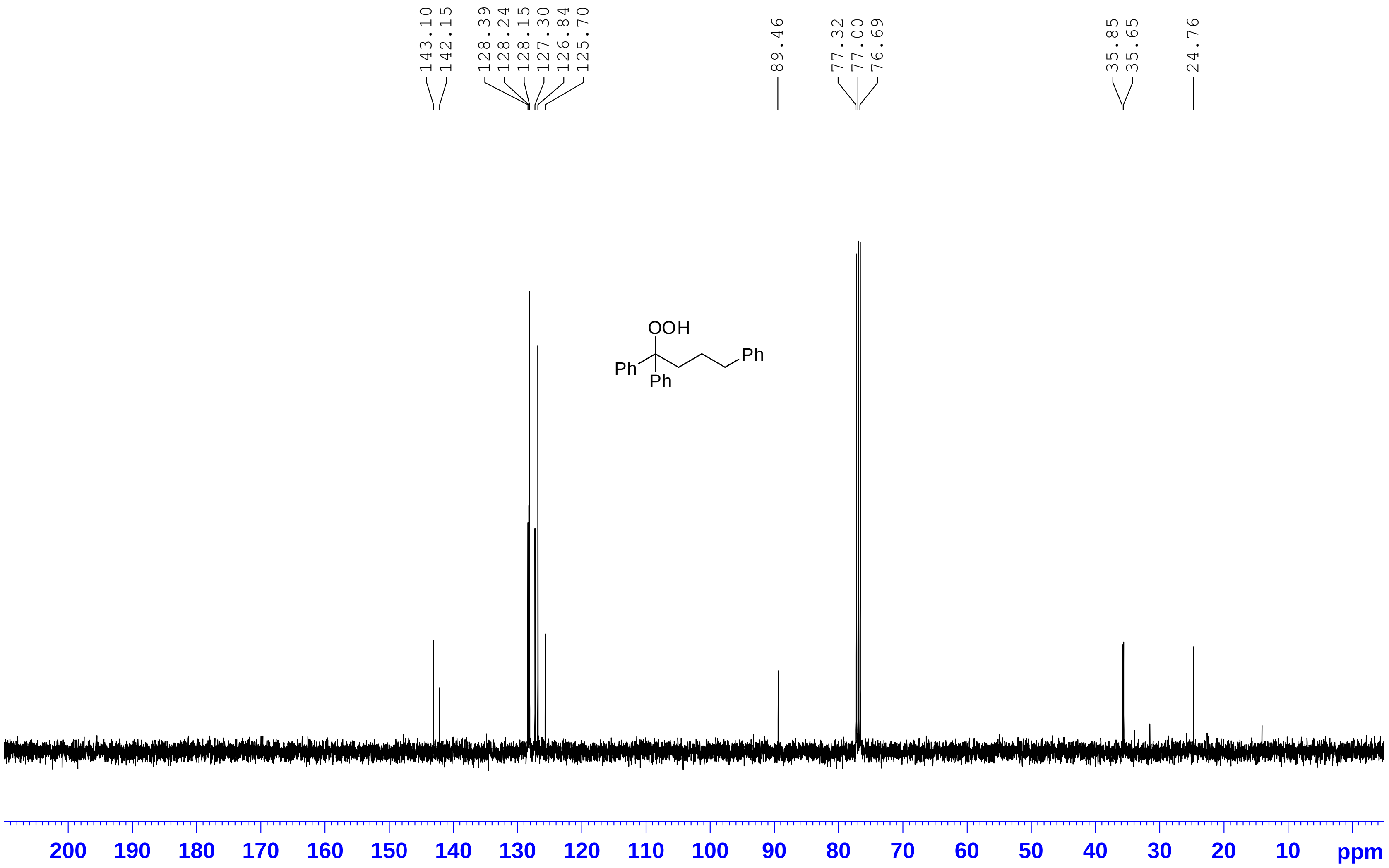

4. ^{1}H and ^{13}C NMR spectrum of new compounds

^{1}H NMR spectrum of **1c** (400 MHz, $CDCl_3$)

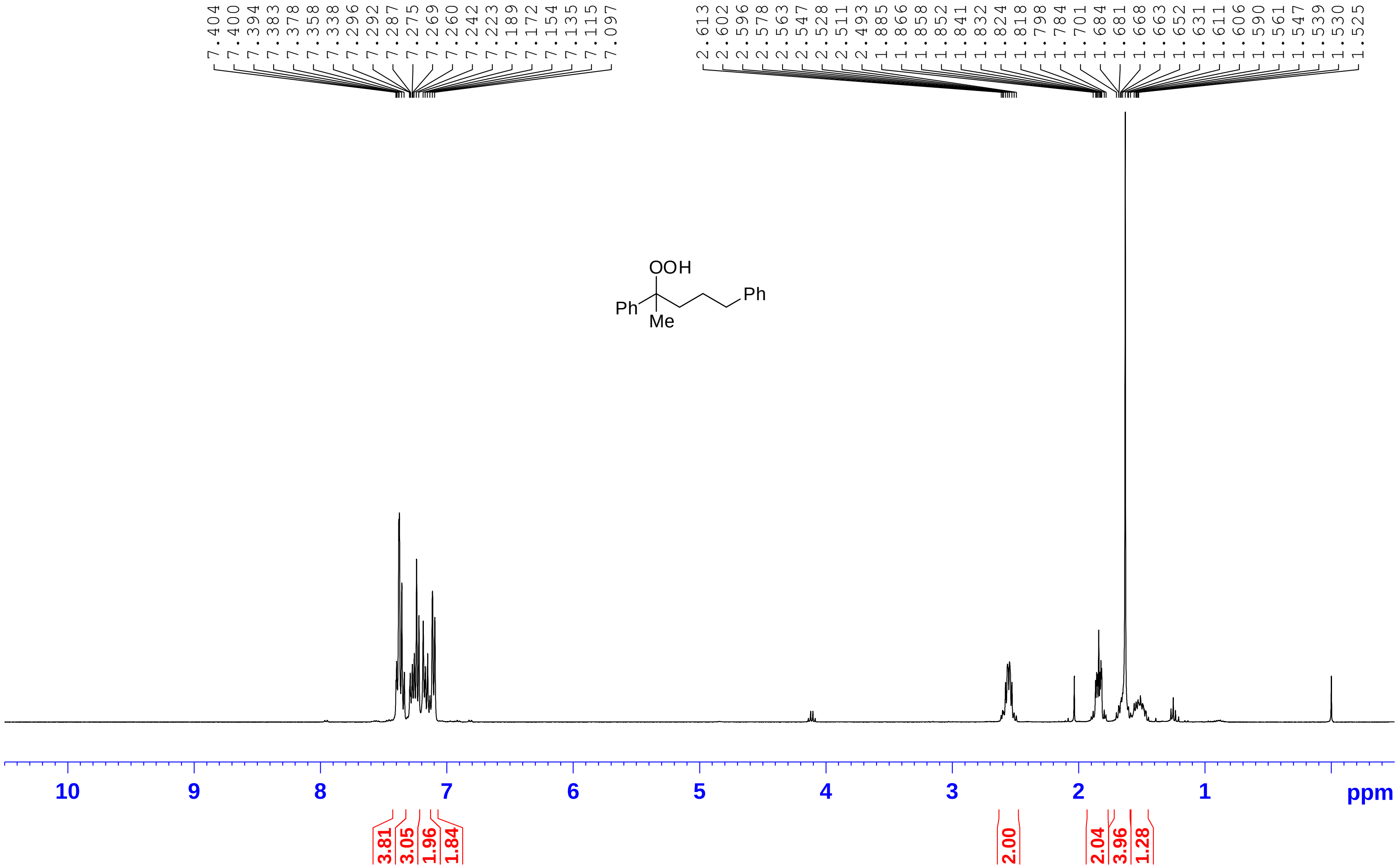

4. ^{1}H and ^{13}C NMR spectrum of new compounds

^{13}C NMR spectrum of **1c** (100 MHz, $CDCl_3$)

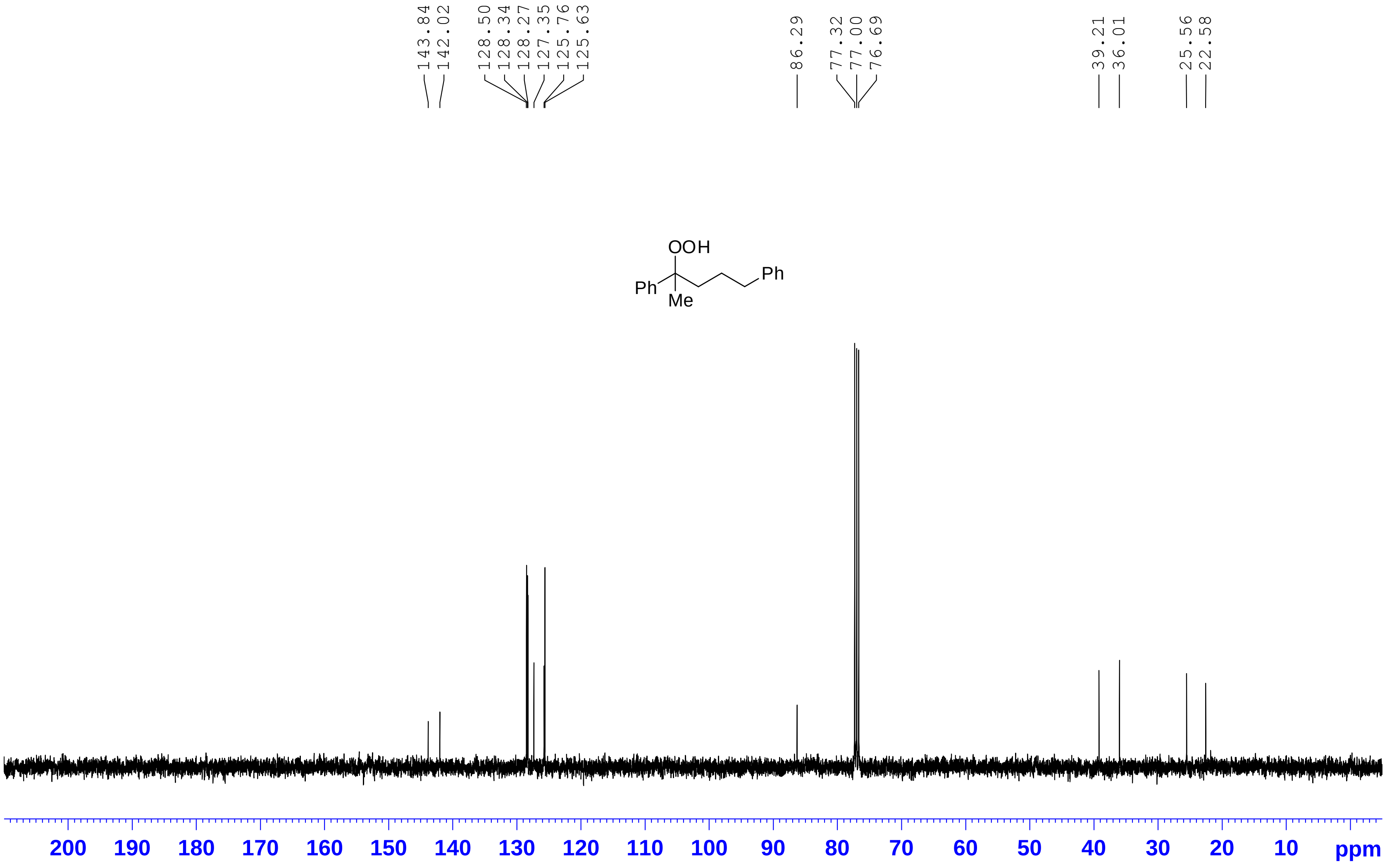

4. ^{1}H and ^{13}C NMR spectrum of new compounds

^{1}H NMR spectrum of **1d** (400 MHz, $CDCl_3$)

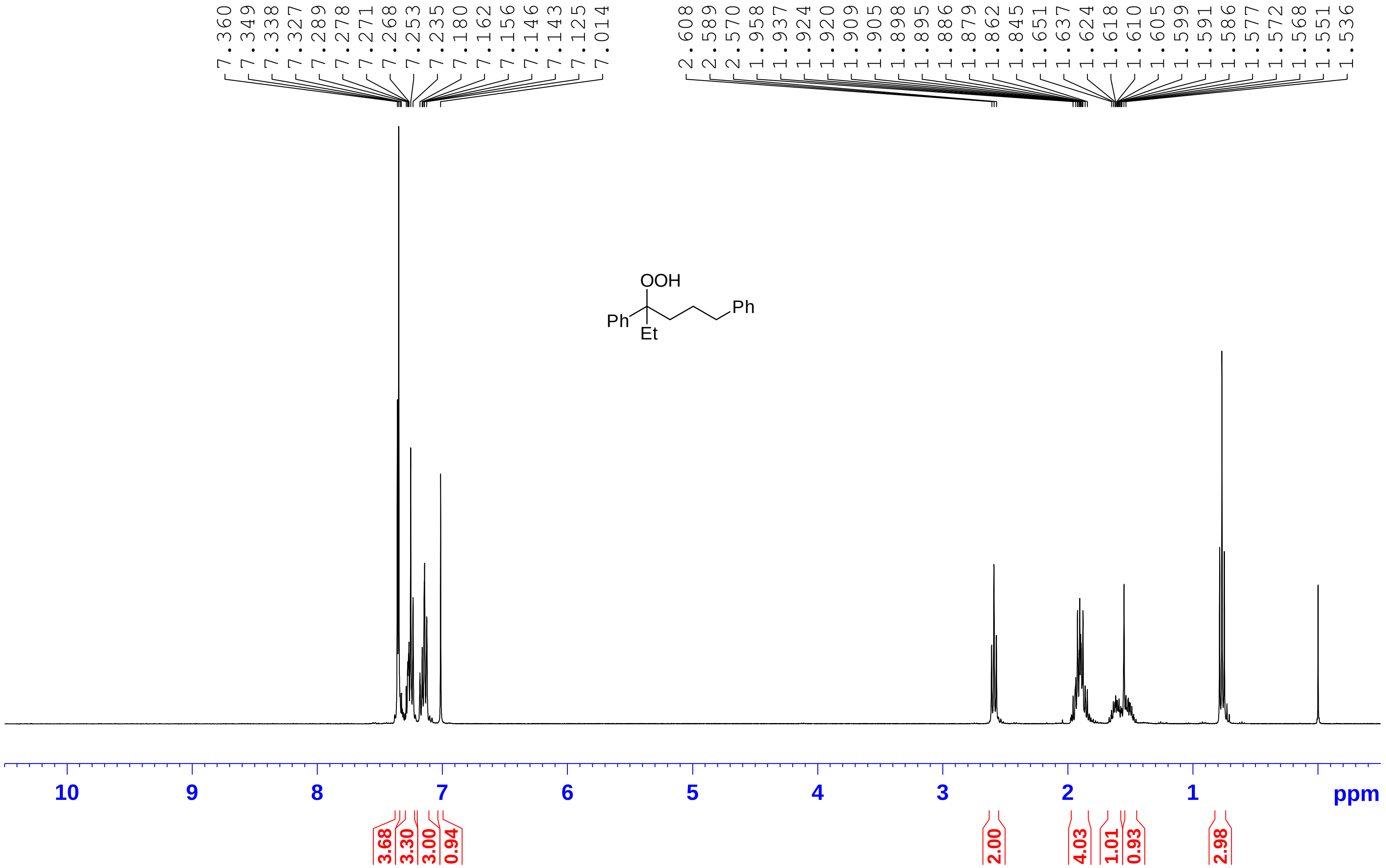

4. ^{1}H and ^{13}C NMR spectrum of new compounds

^{13}C NMR spectrum of **1d** (100 MHz, $CDCl_3$)

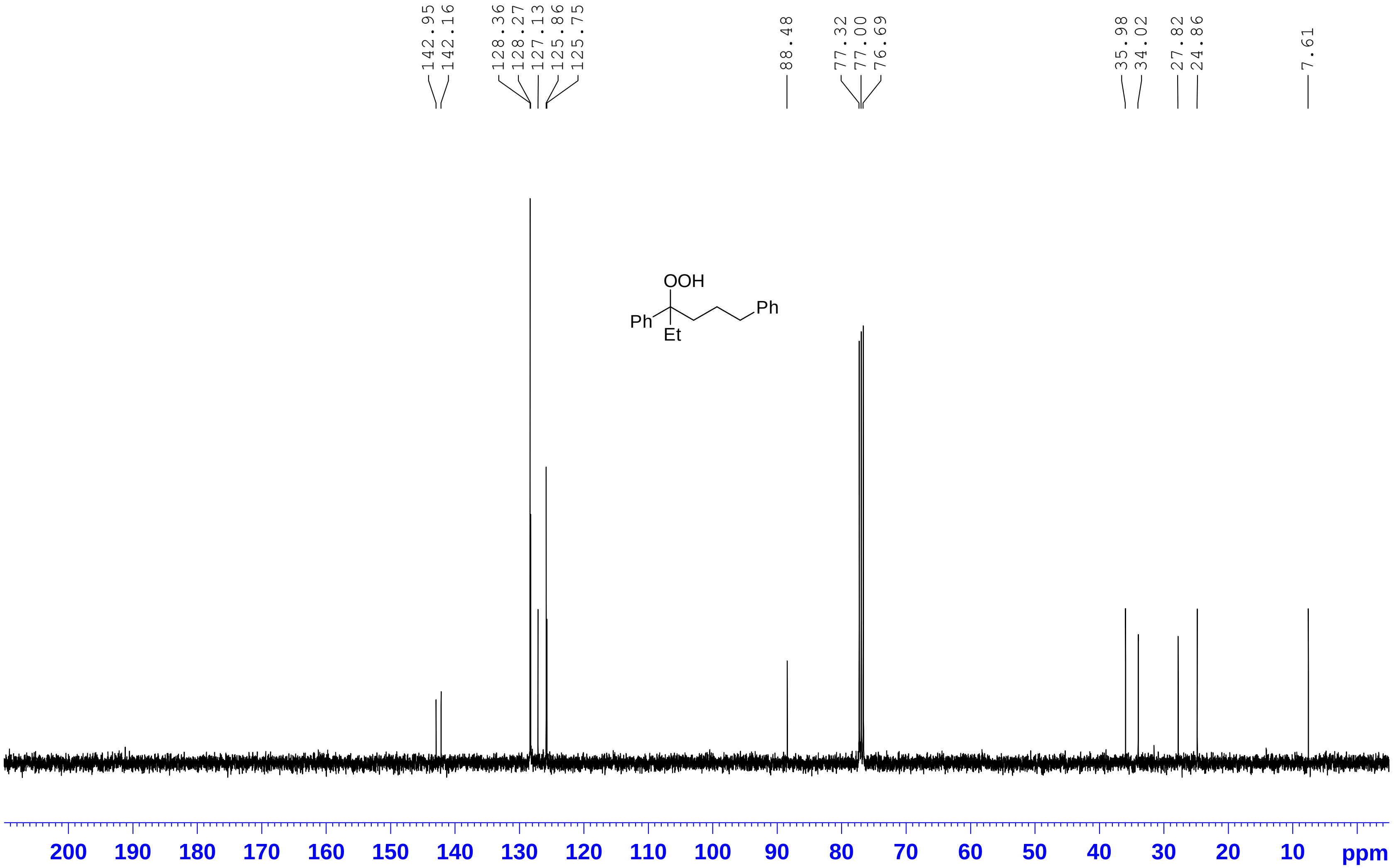

4. ^{1}H and ^{13}C NMR spectrum of new compounds

^{1}H NMR spectrum of **1e** (400 MHz, $CDCl_3$)

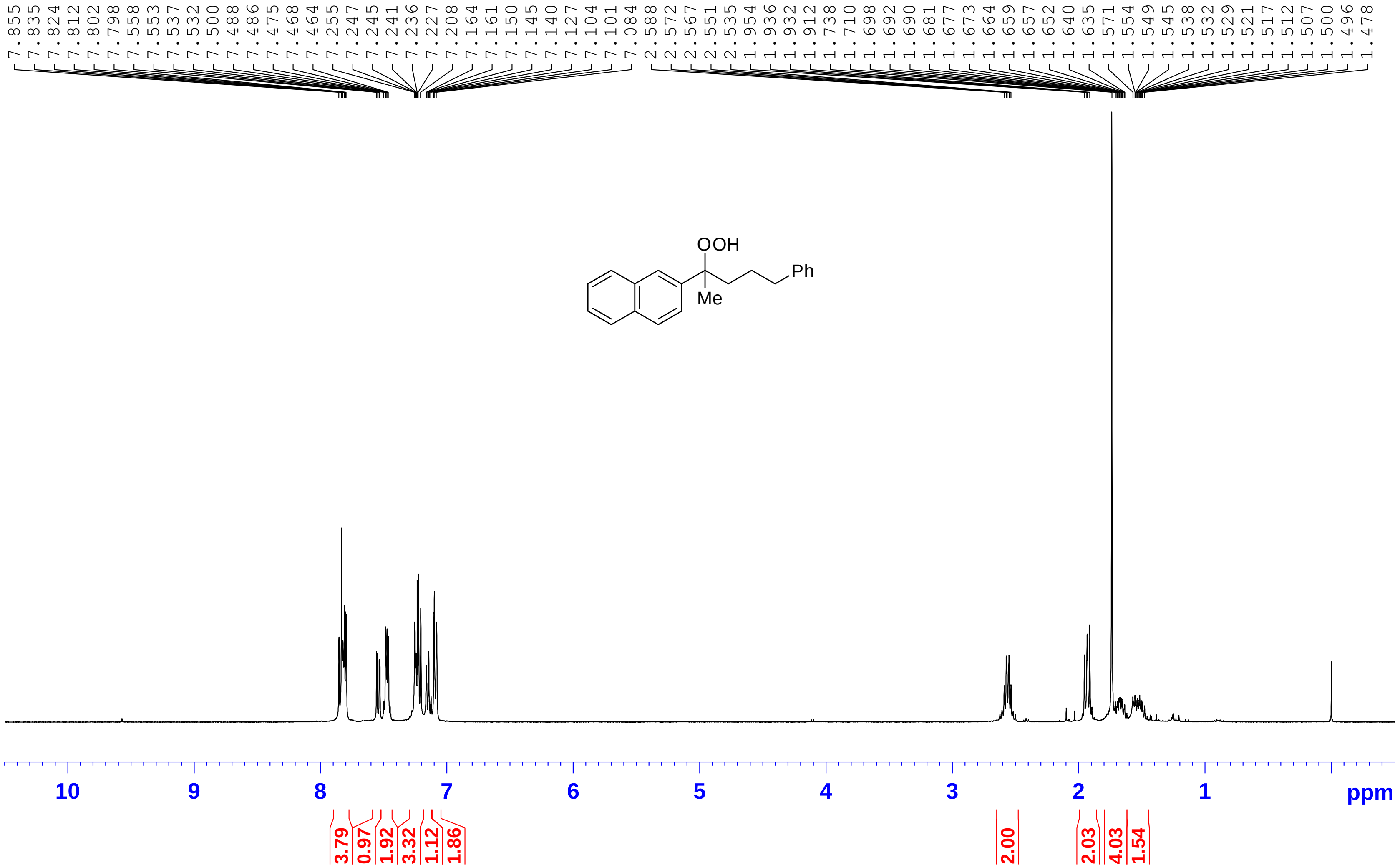

4. ^{1}H and ^{13}C NMR spectrum of new compounds

^{13}C NMR spectrum of **1e** (100 MHz, $CDCl_3$)

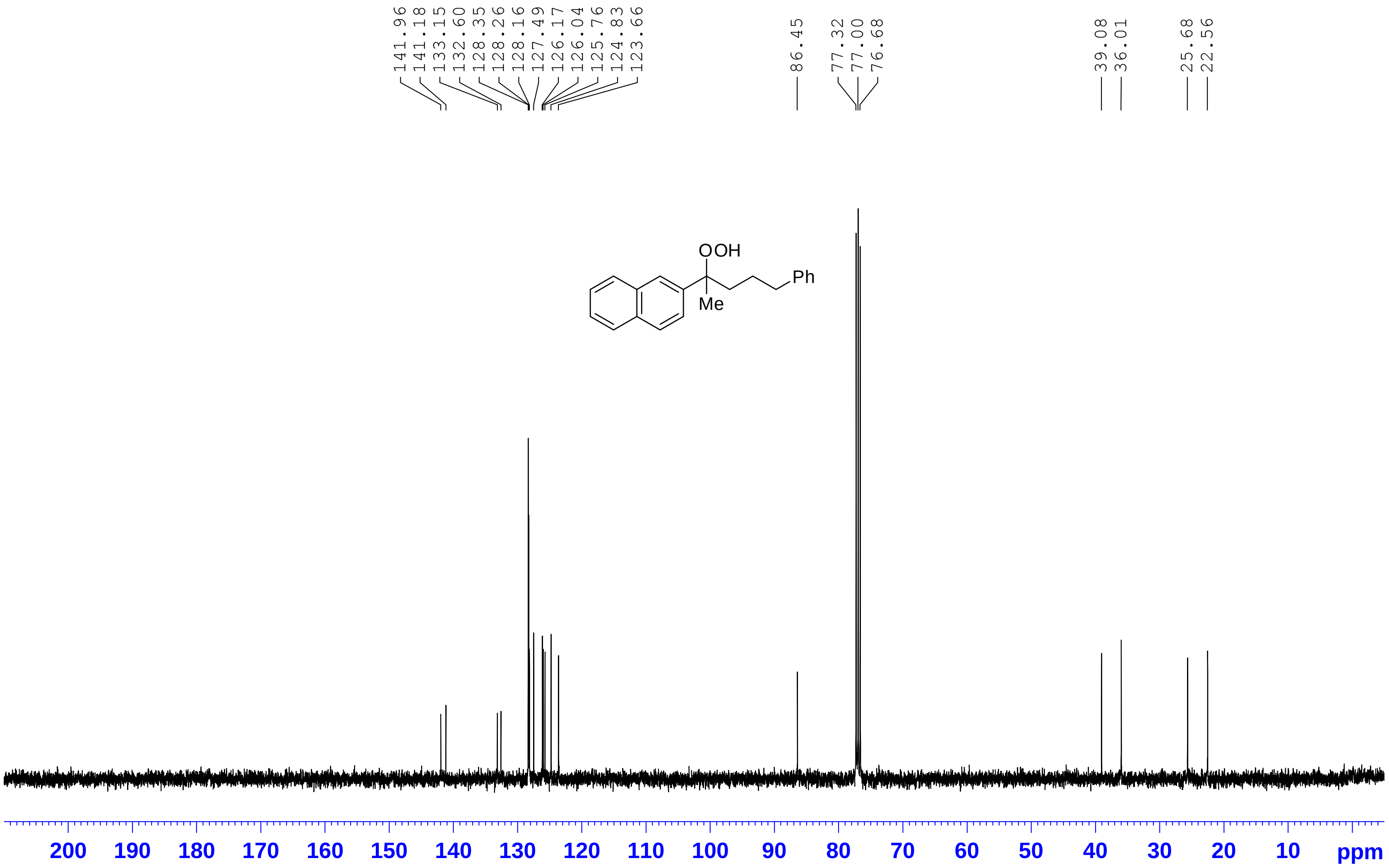

4. ^{1}H and ^{13}C NMR spectrum of new compounds

^{1}H NMR spectrum of **1f** (400 MHz, $CDCl_3$)

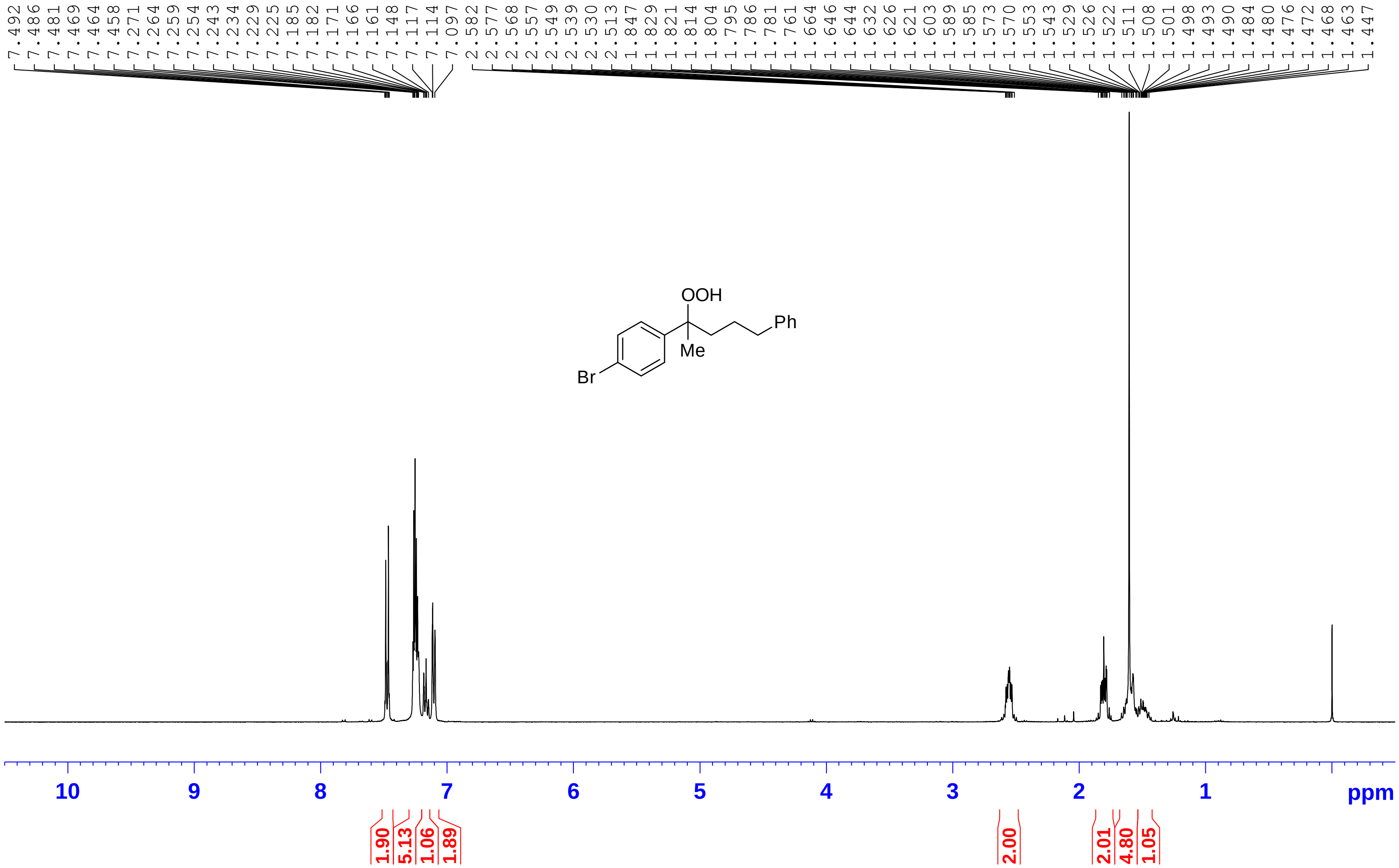

4. ^{1}H and ^{13}C NMR spectrum of new compounds

^{13}C NMR spectrum of **1f** (100 MHz, $CDCl_3$)

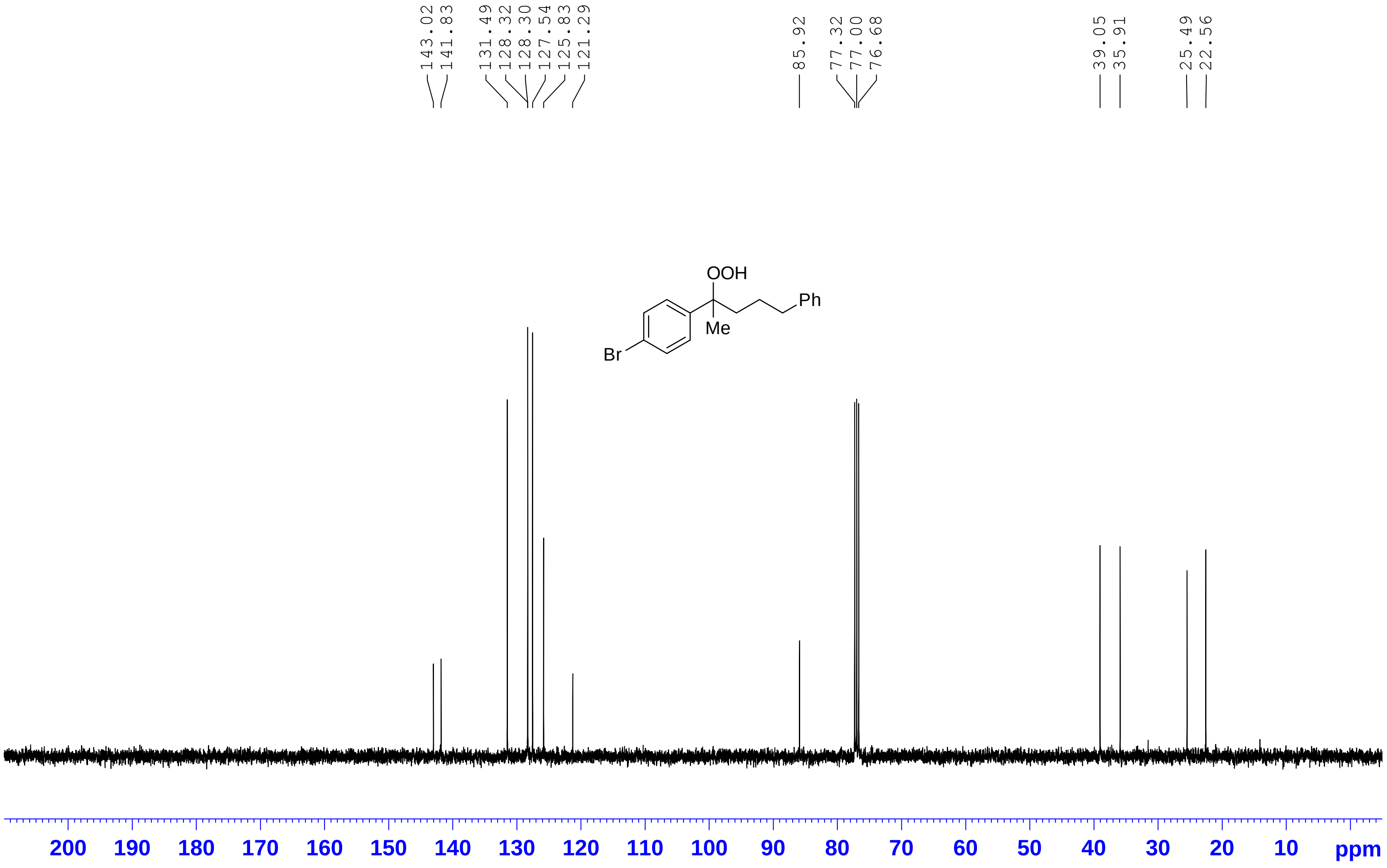

4. ^{1}H and ^{13}C NMR spectrum of new compounds

^{1}H NMR spectrum of **1h** (400 MHz, $CDCl_3$)

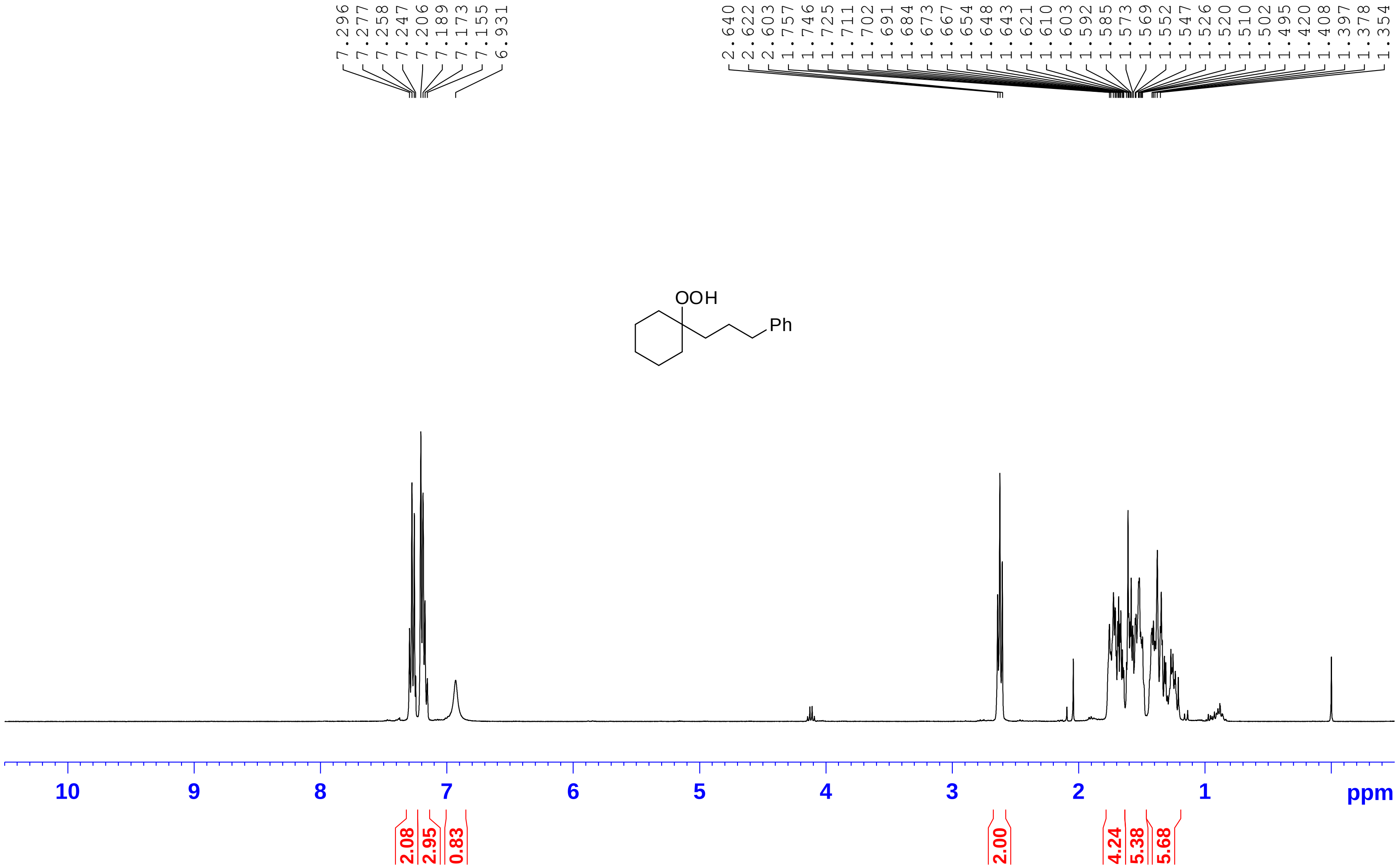

4. ^{1}H and ^{13}C NMR spectrum of new compounds

^{13}C NMR spectrum of **1h** (100 MHz, $CDCl_3$)

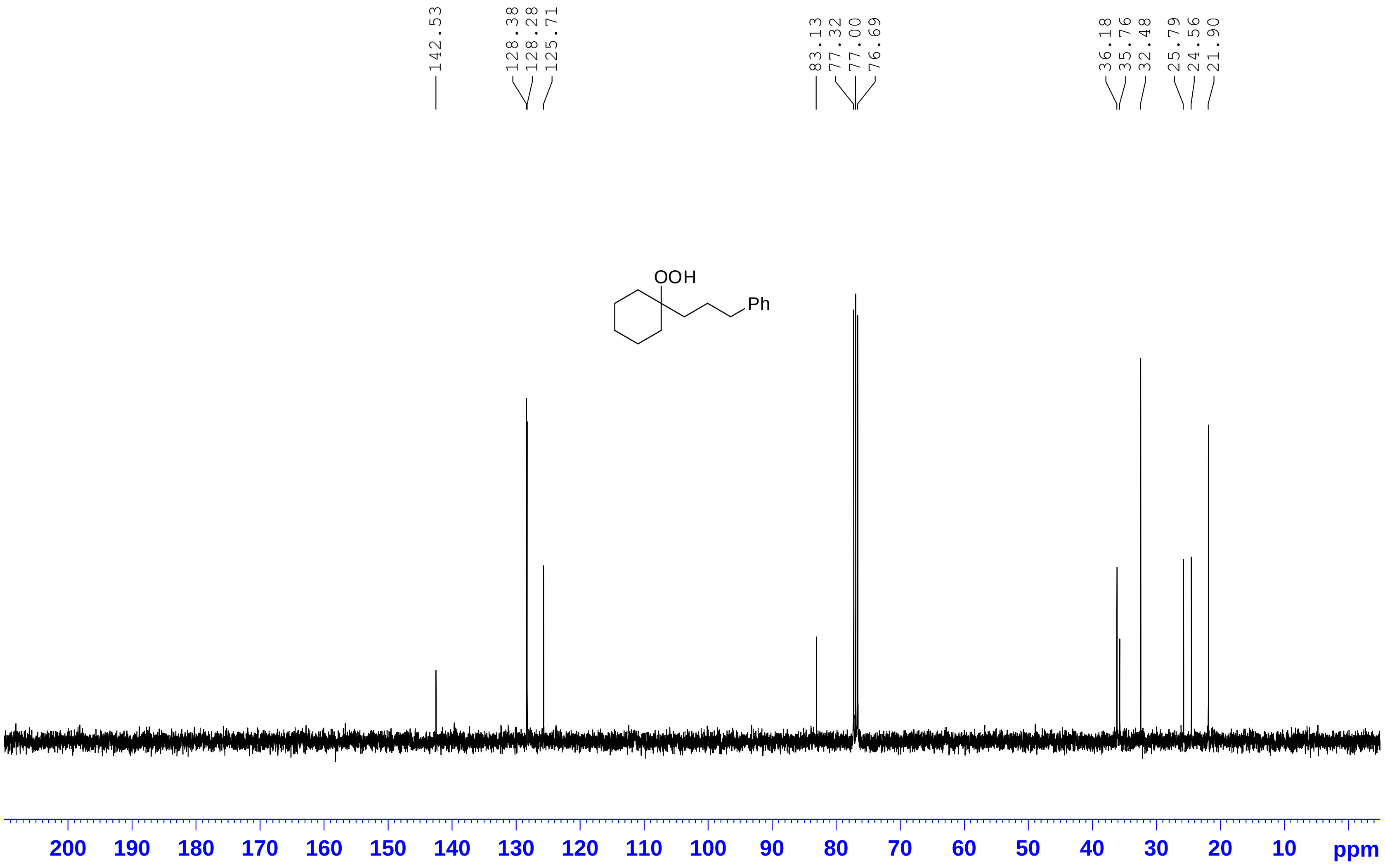

4. ^{1}H and ^{13}C NMR spectrum of new compounds

^{1}H NMR spectrum of **1i** (400 MHz, $CDCl_3$)

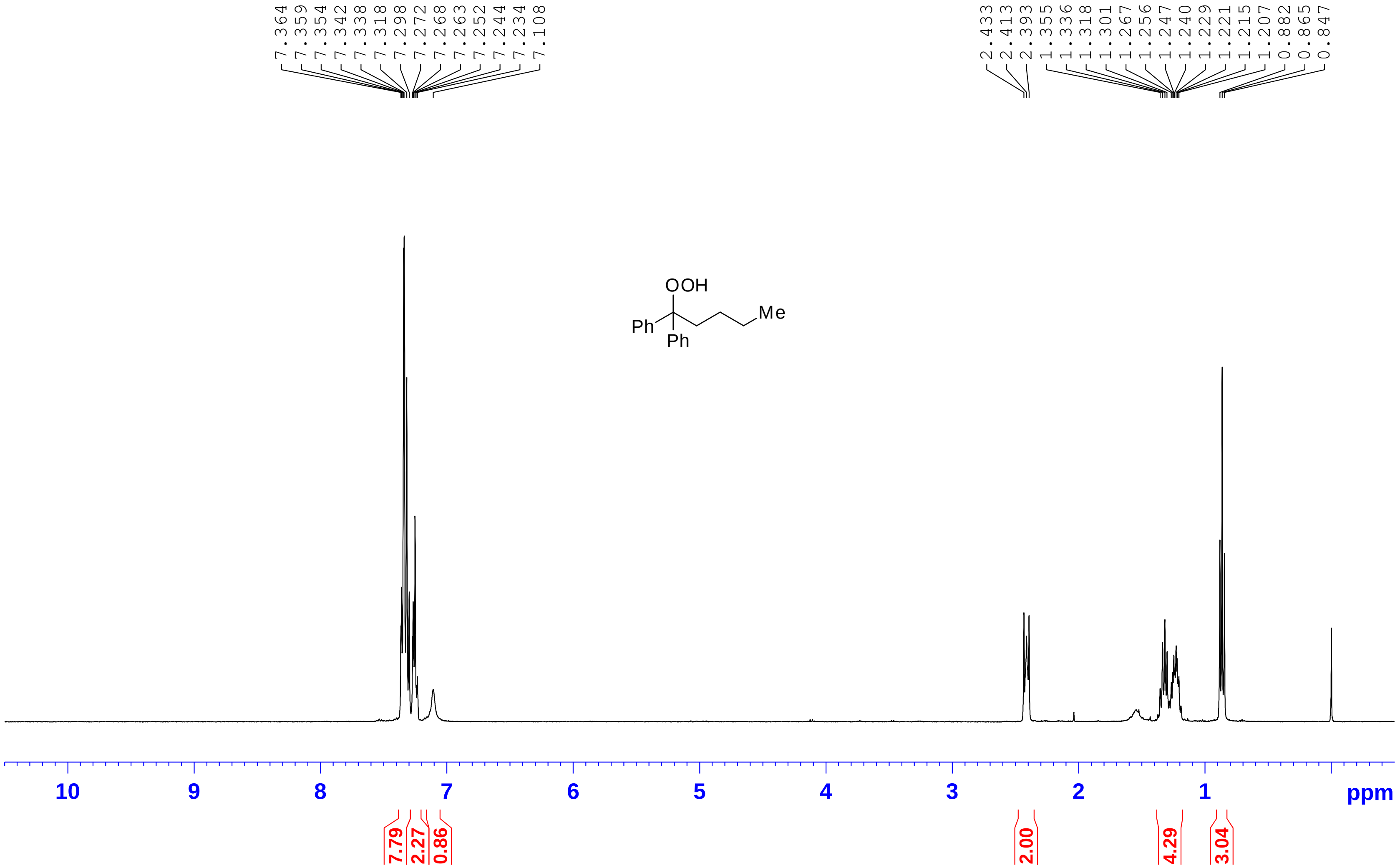

4. ^{1}H and ^{13}C NMR spectrum of new compounds

^{13}C NMR spectrum of **1i** (100 MHz, $CDCl_3$)

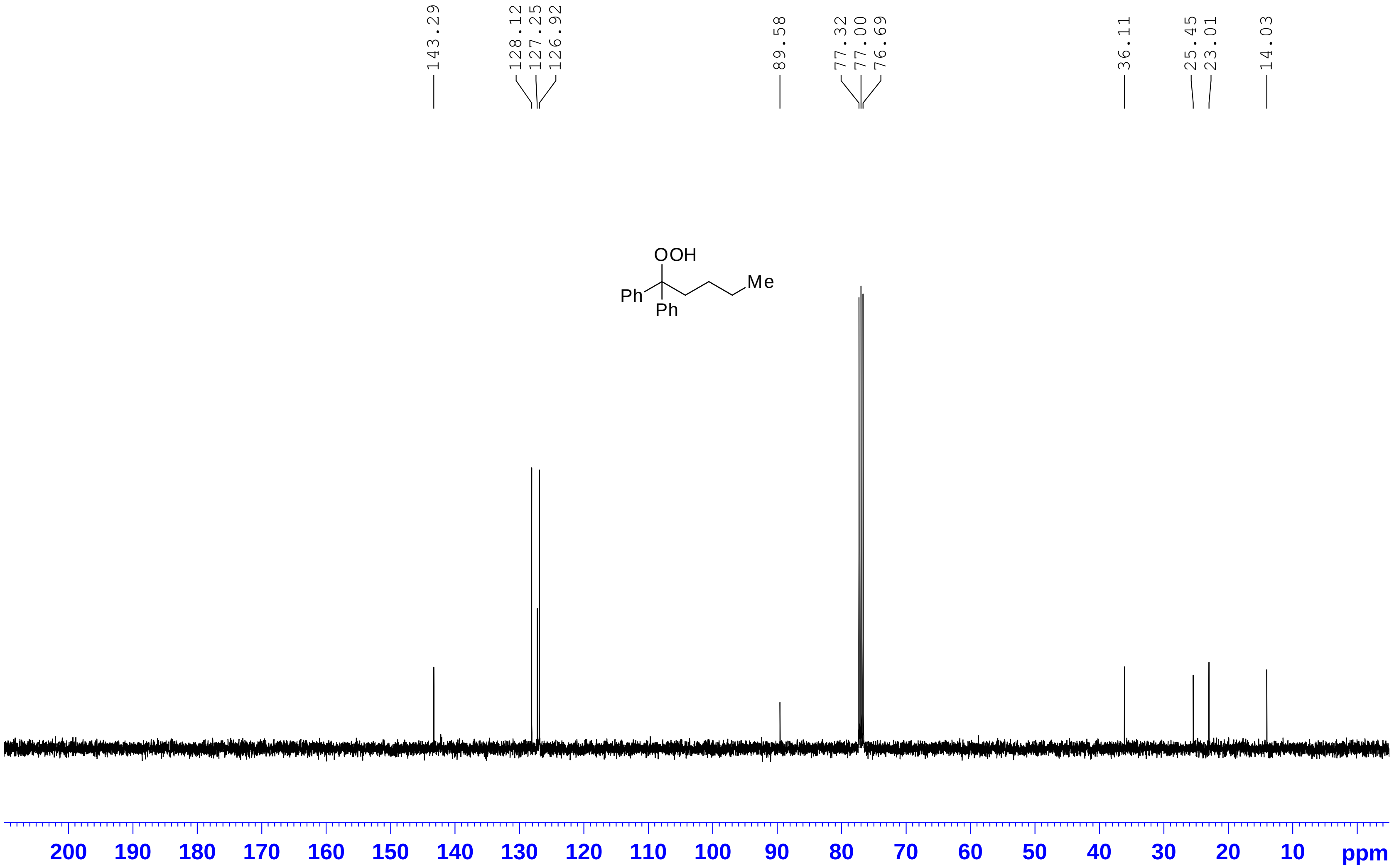

4. ^{1}H and ^{13}C NMR spectrum of new compounds

^{1}H NMR spectrum of **1k** (400 MHz, $CDCl_3$)

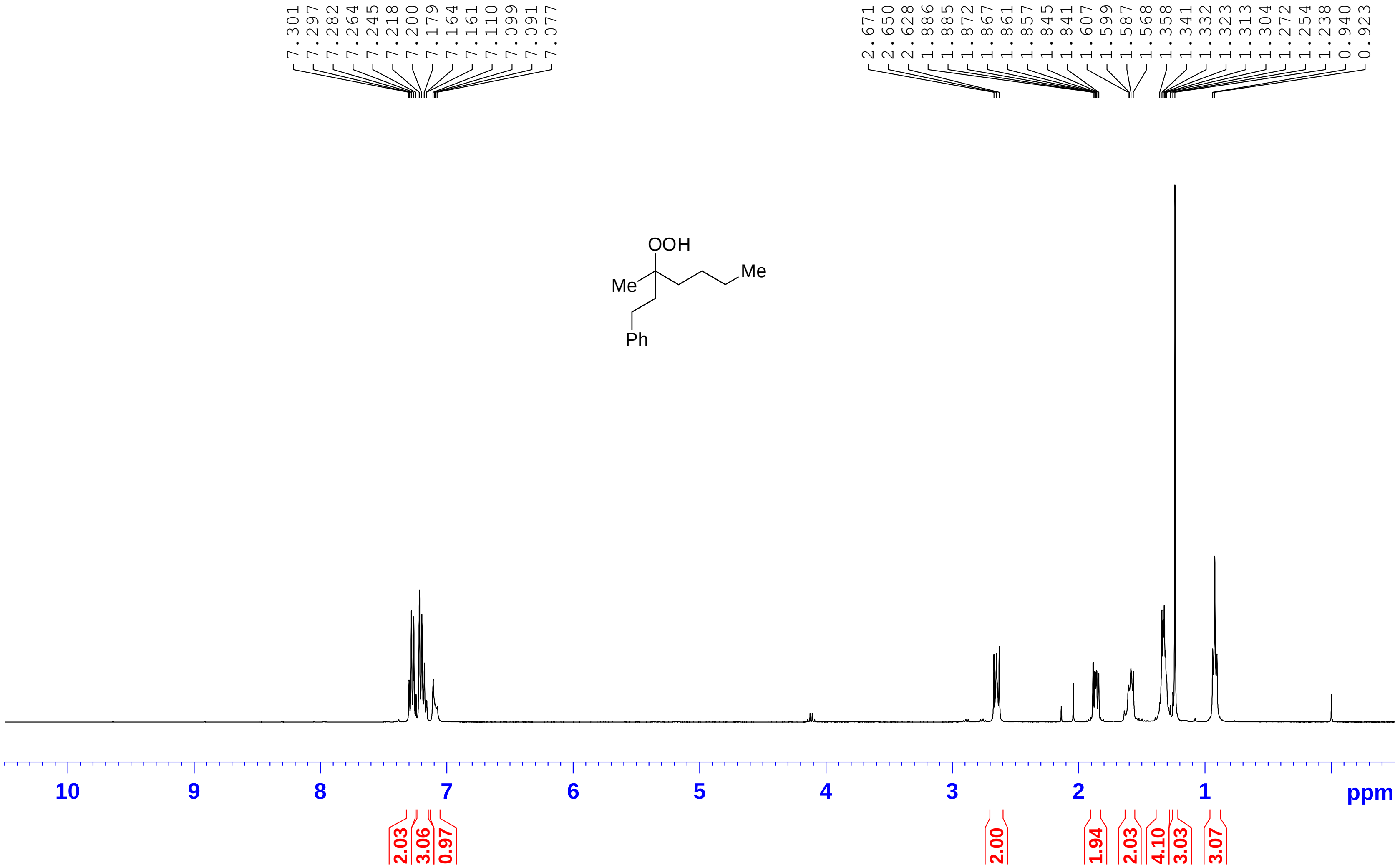

4. ^{1}H and ^{13}C NMR spectrum of new compounds

^{13}C NMR spectrum of **1k** (100 MHz, $CDCl_3$)

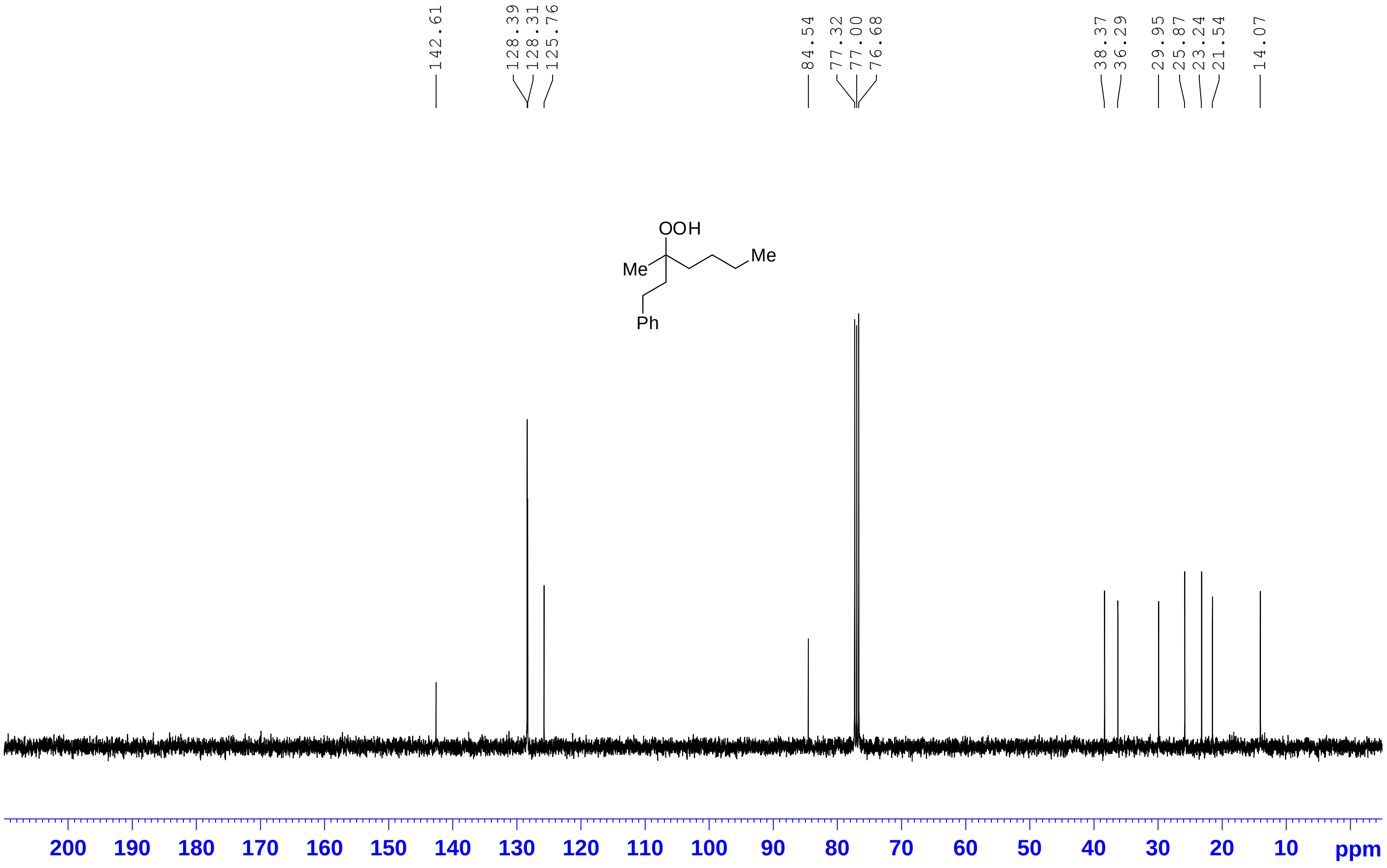

4. ^{1}H and ^{13}C NMR spectrum of new compounds

^{1}H NMR spectrum of **1l** (400 MHz, $CDCl_3$)

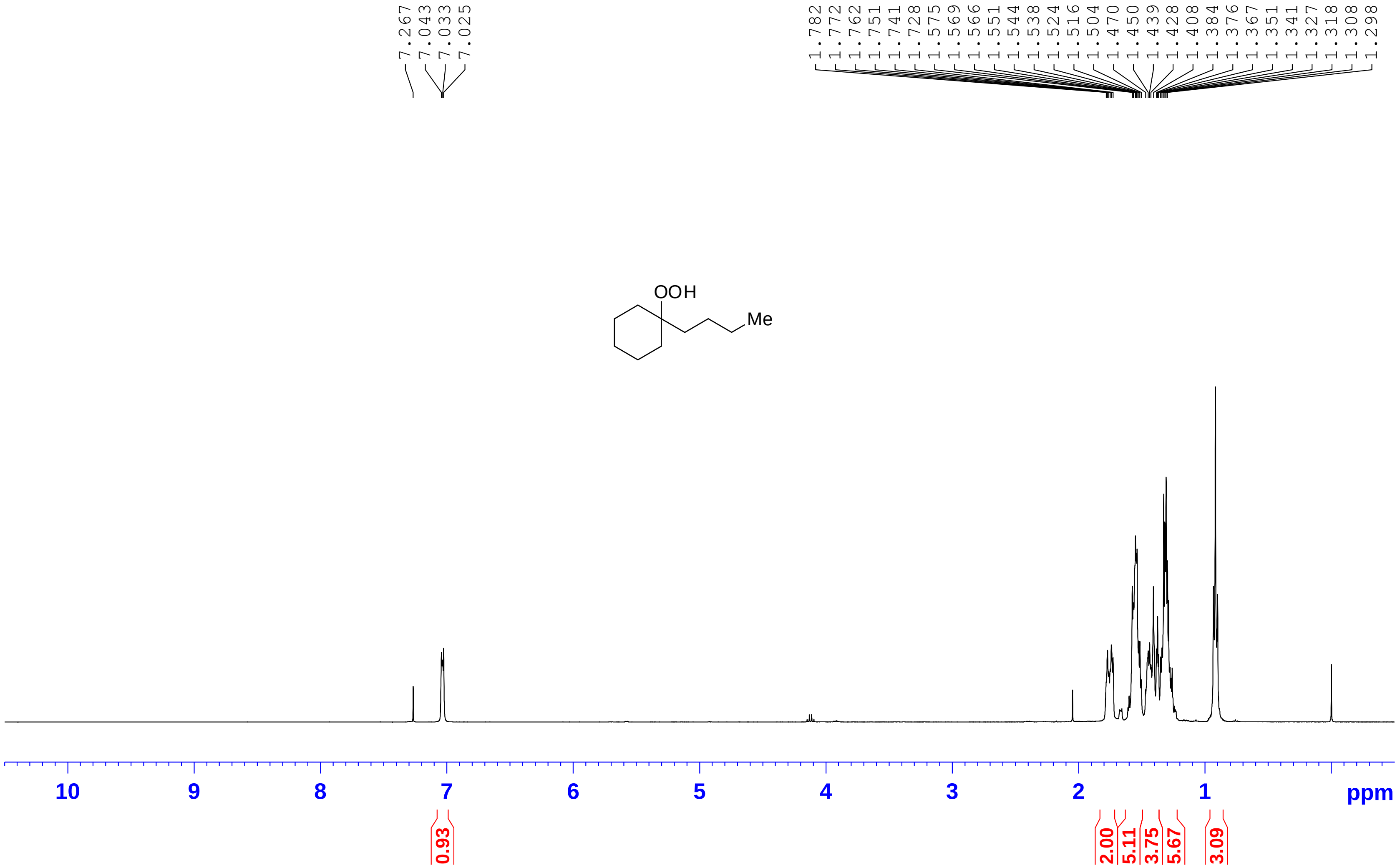

4. ^{1}H and ^{13}C NMR spectrum of new compounds

^{13}C NMR spectrum of **1l** (100 MHz, $CDCl_3$)

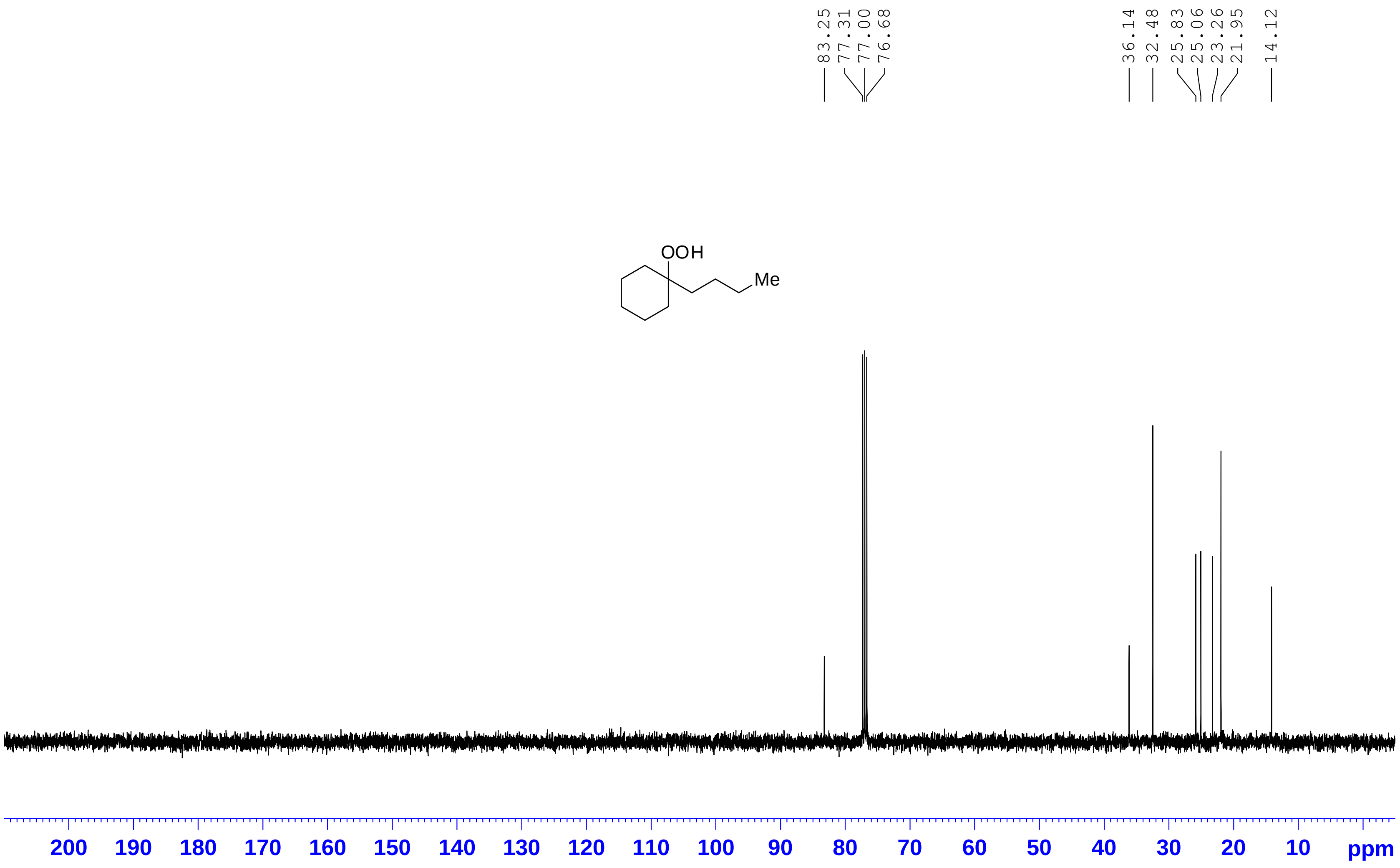

4. ^{1}H and ^{13}C NMR spectrum of new compounds

^{1}H NMR spectrum of **1m** (400 MHz, $CDCl_3$)

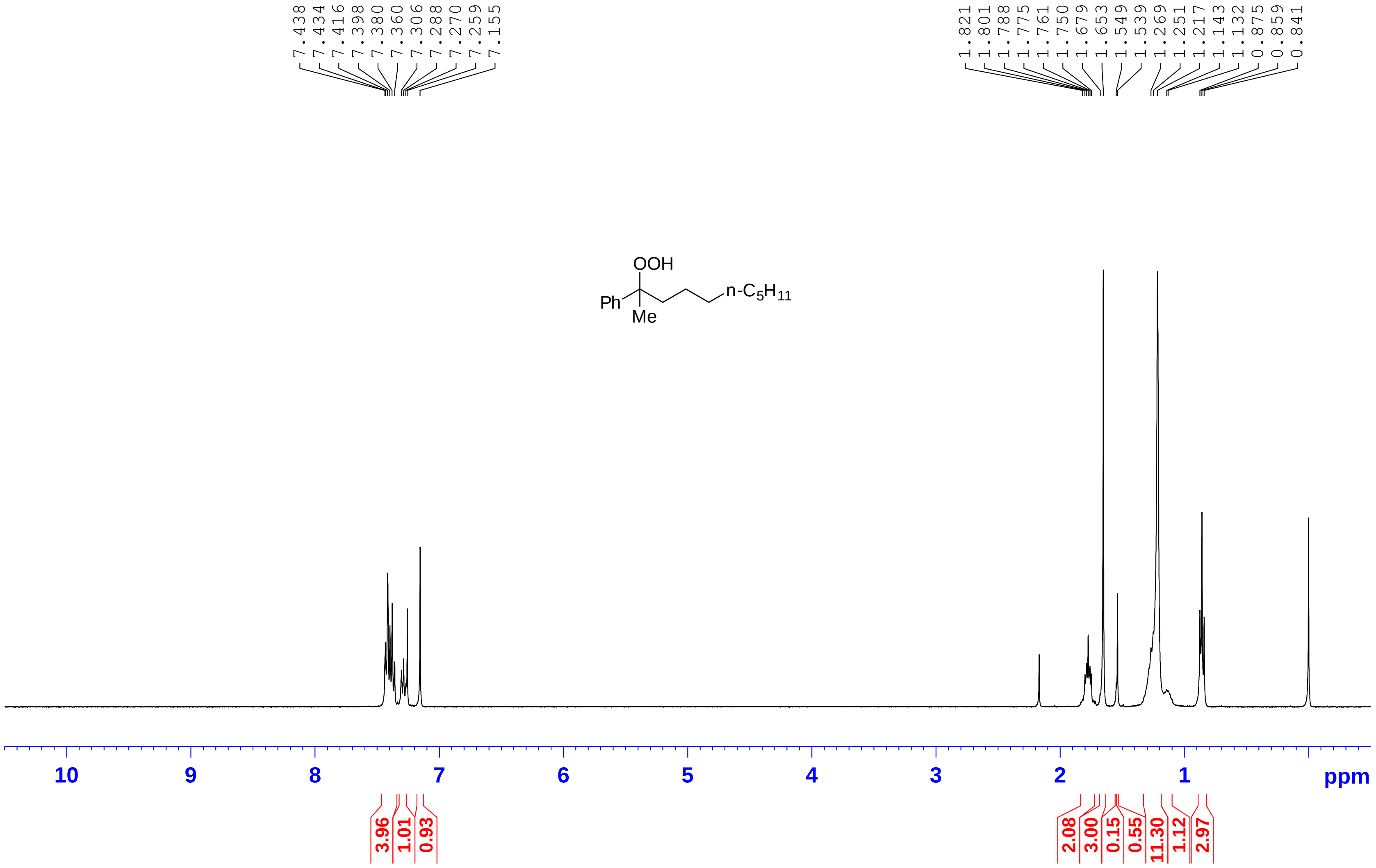

4. ^{1}H and ^{13}C NMR spectrum of new compounds

^{13}C NMR spectrum of **1m** (100 MHz, CDCl$_3$)

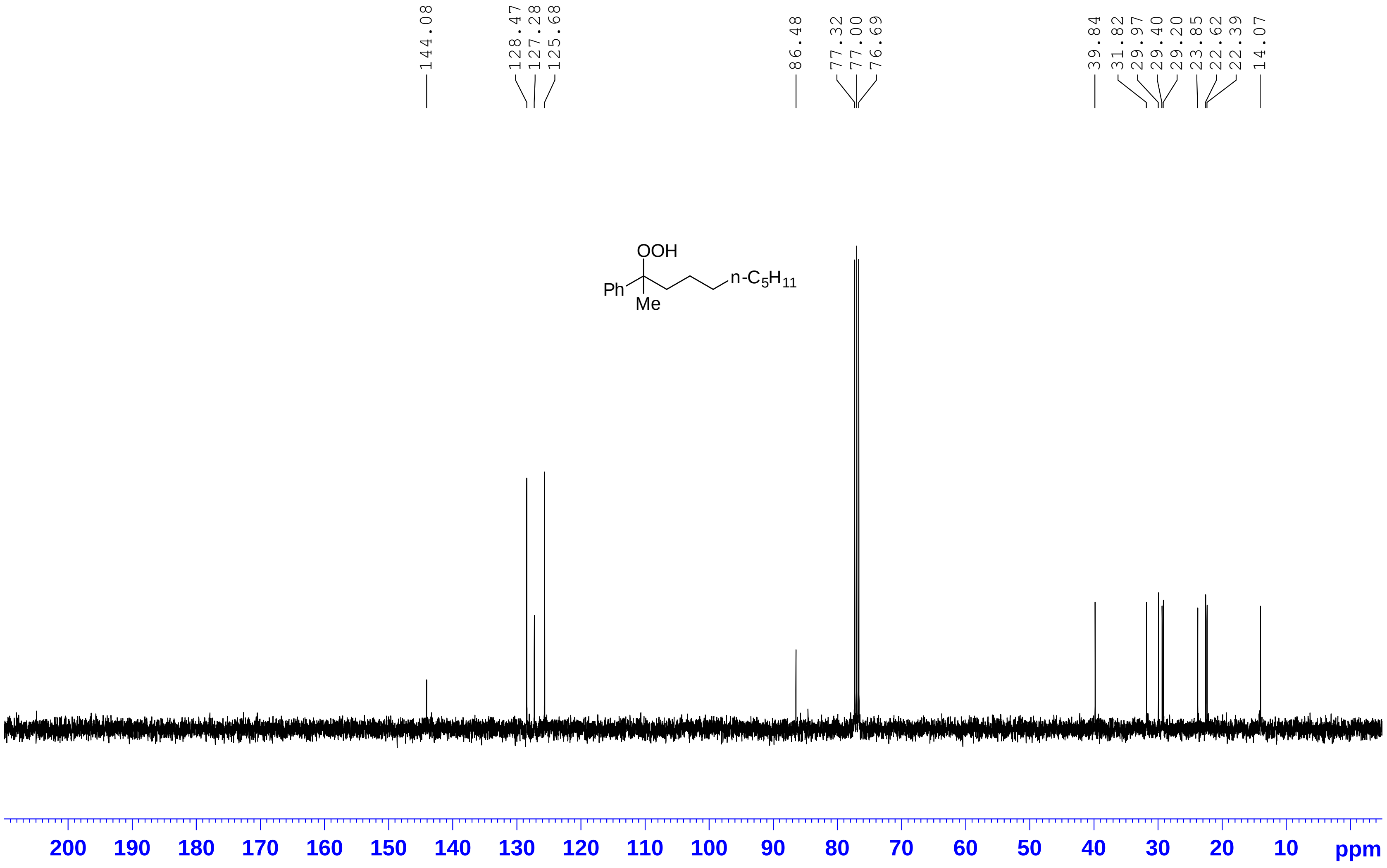

4. ^{1}H and ^{13}C NMR spectrum of new compounds

^{1}H NMR spectrum of **1n** (400 MHz, $CDCl_3$)

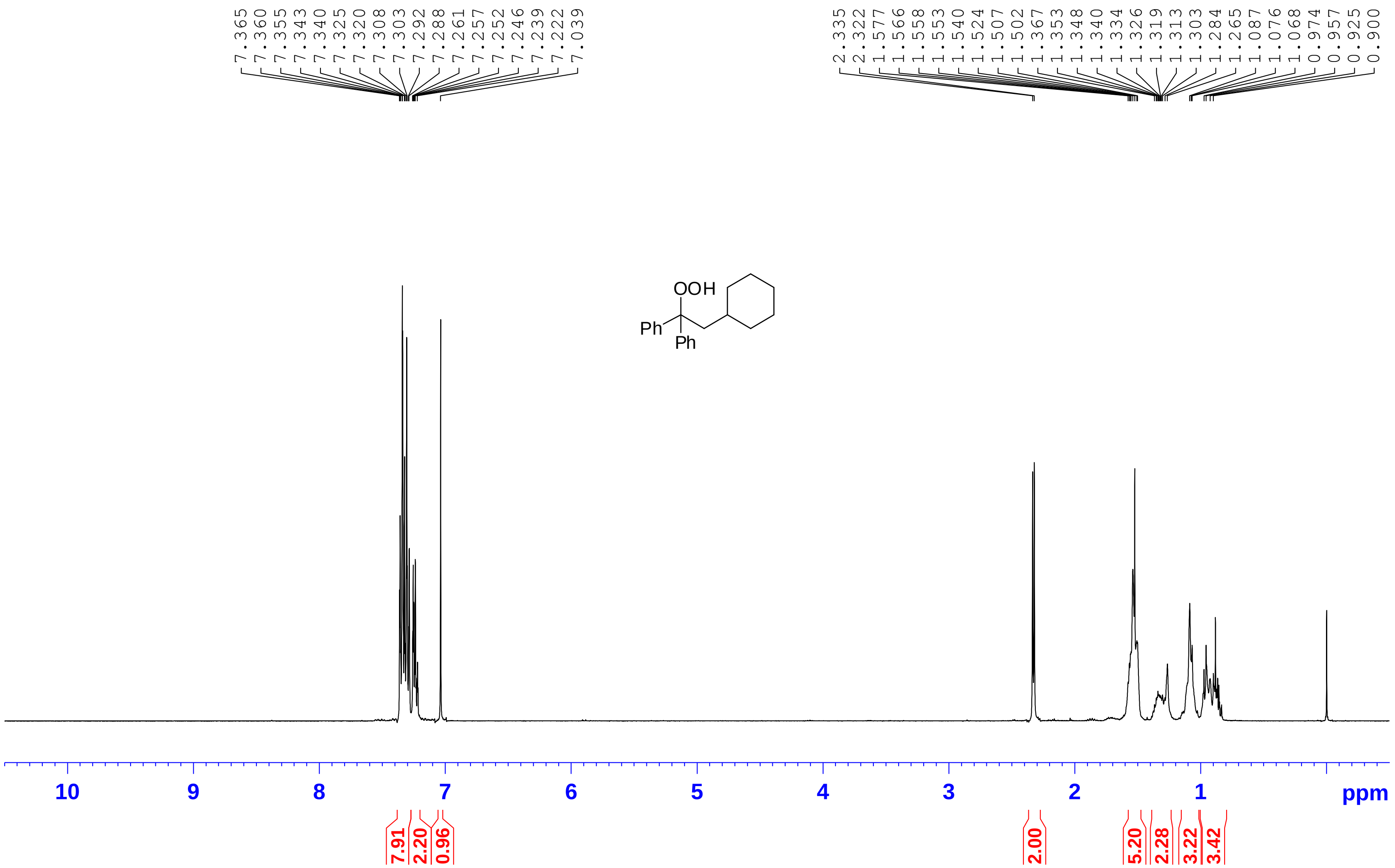

4. ^{1}H and ^{13}C NMR spectrum of new compounds

^{13}C NMR spectrum of **1n** (100 MHz, $CDCl_3$)

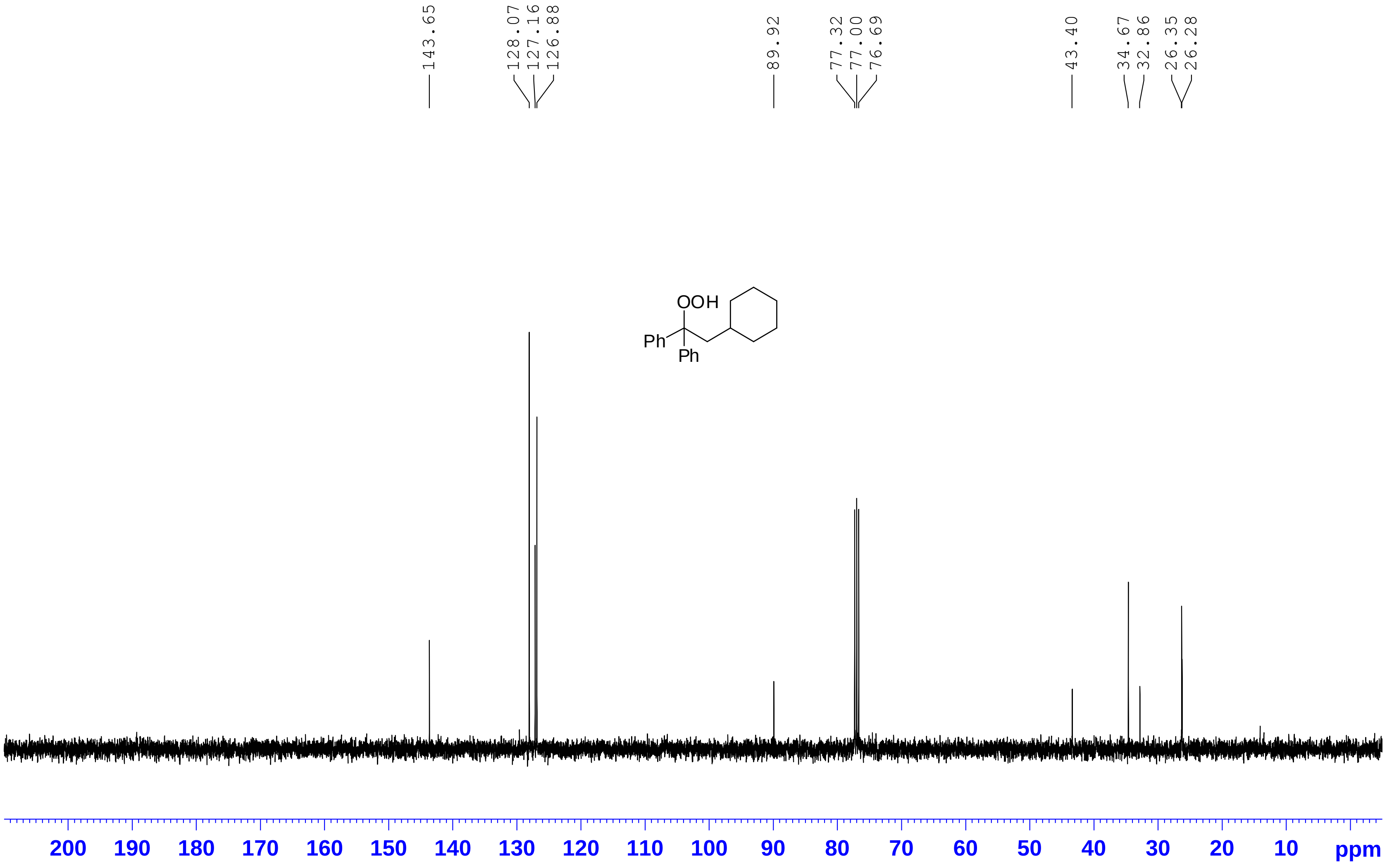

4. ^{1}H and ^{13}C NMR spectrum of new compounds

^{1}H NMR spectrum of **1o** (400 MHz, $CDCl_3$)

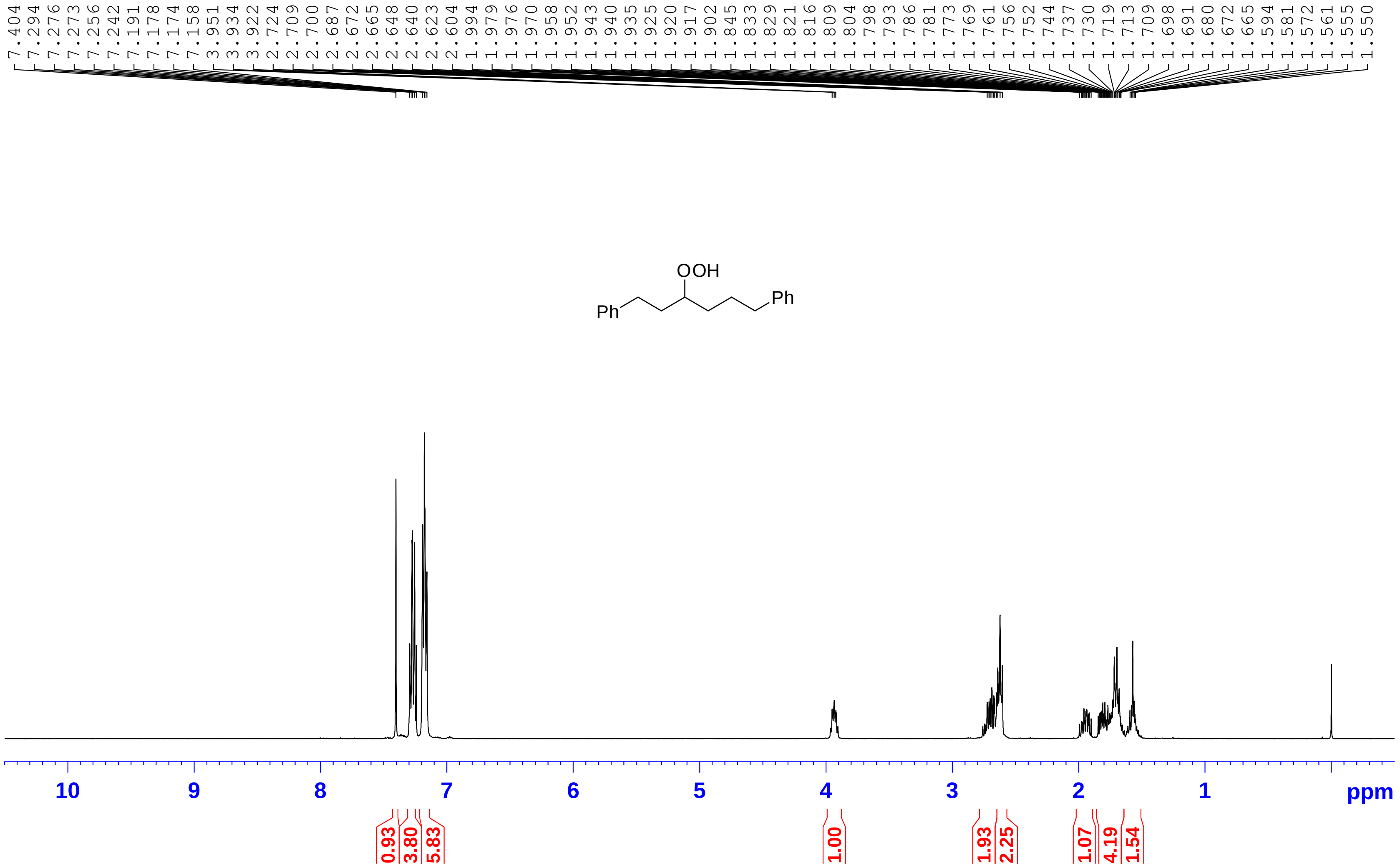

4. ^{1}H and ^{13}C NMR spectrum of new compounds

^{13}C NMR spectrum of **1o** (100 MHz, $CDCl_3$)

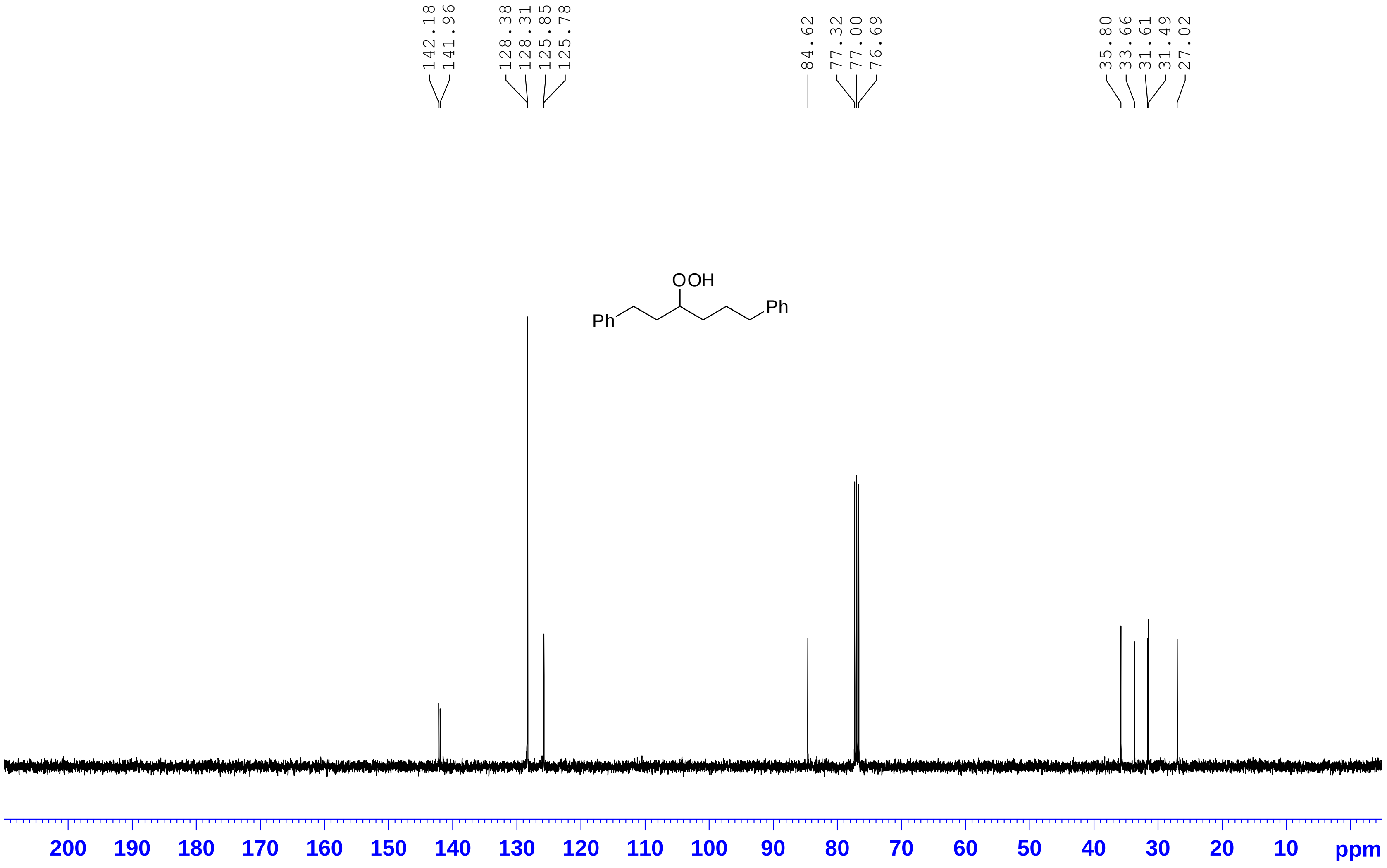

4. ^{1}H and ^{13}C NMR spectrum of new compounds

^{1}H NMR spectrum of **1p** (400 MHz, $CDCl_3$)

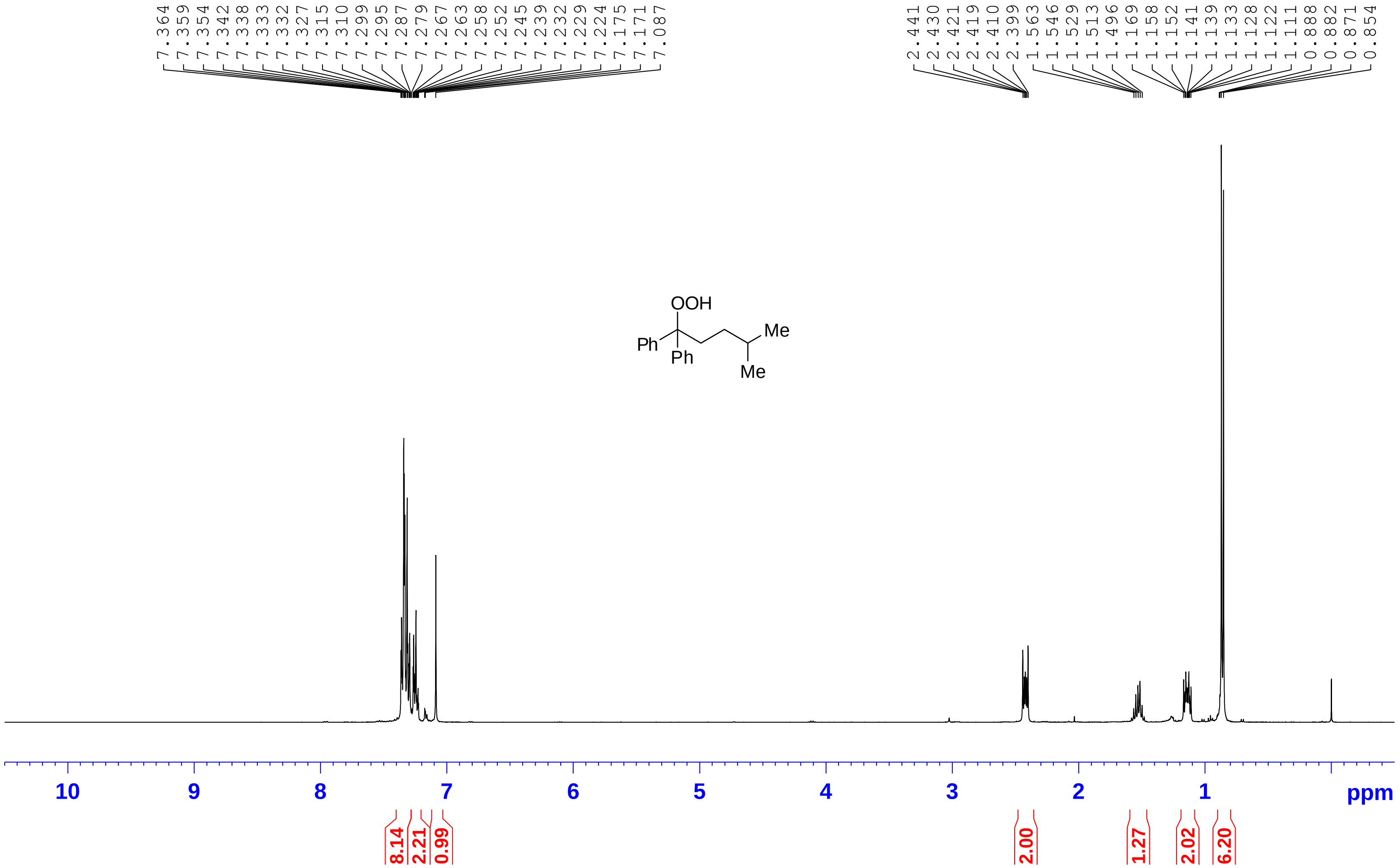

4. ^{1}H and ^{13}C NMR spectrum of new compounds

^{13}C NMR spectrum of **1p** (100 MHz, $CDCl_3$)

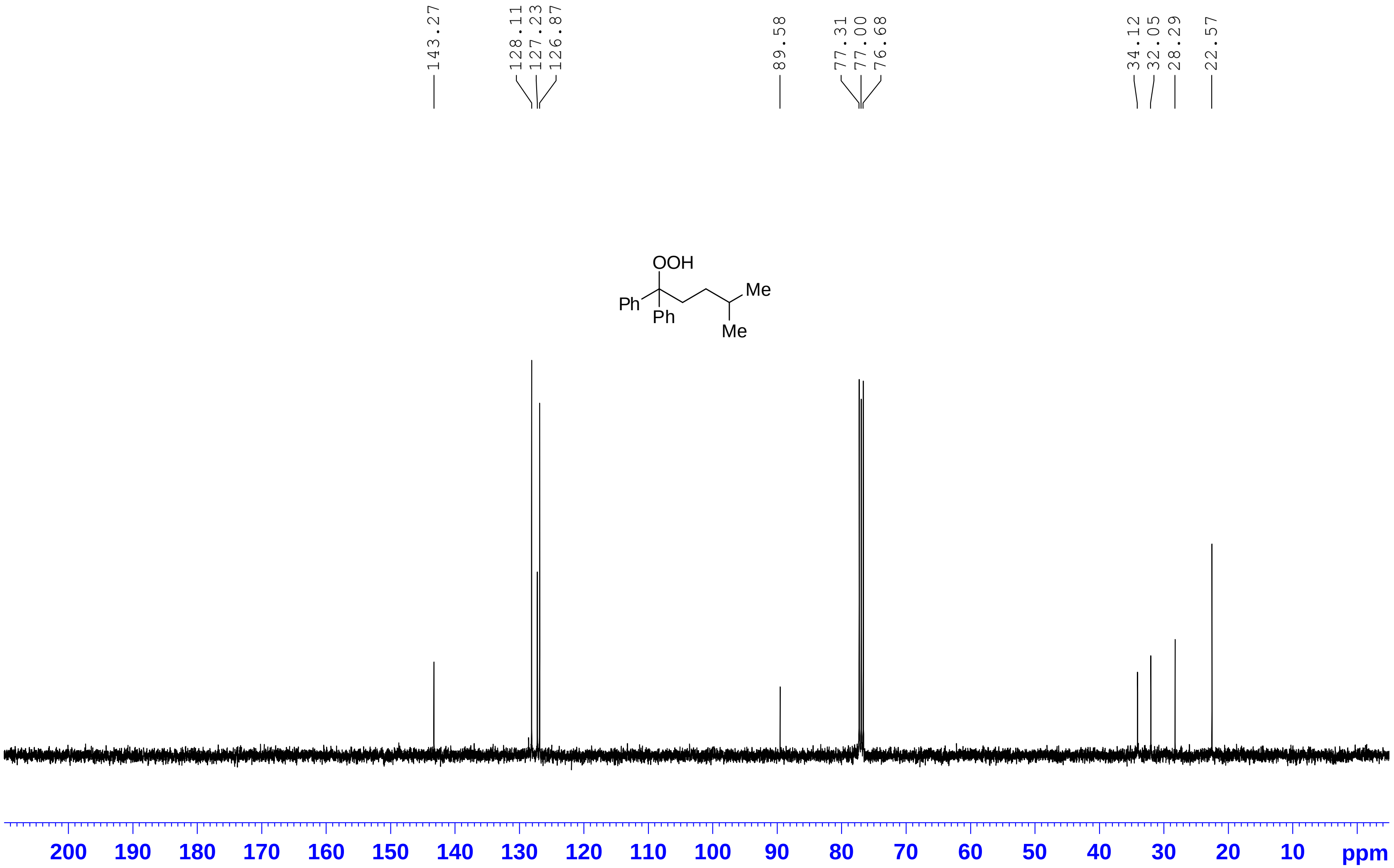

4. ^{1}H and ^{13}C NMR spectrum of new compounds

^{1}H NMR spectrum of **1r** (400 MHz, $CDCl_3$)

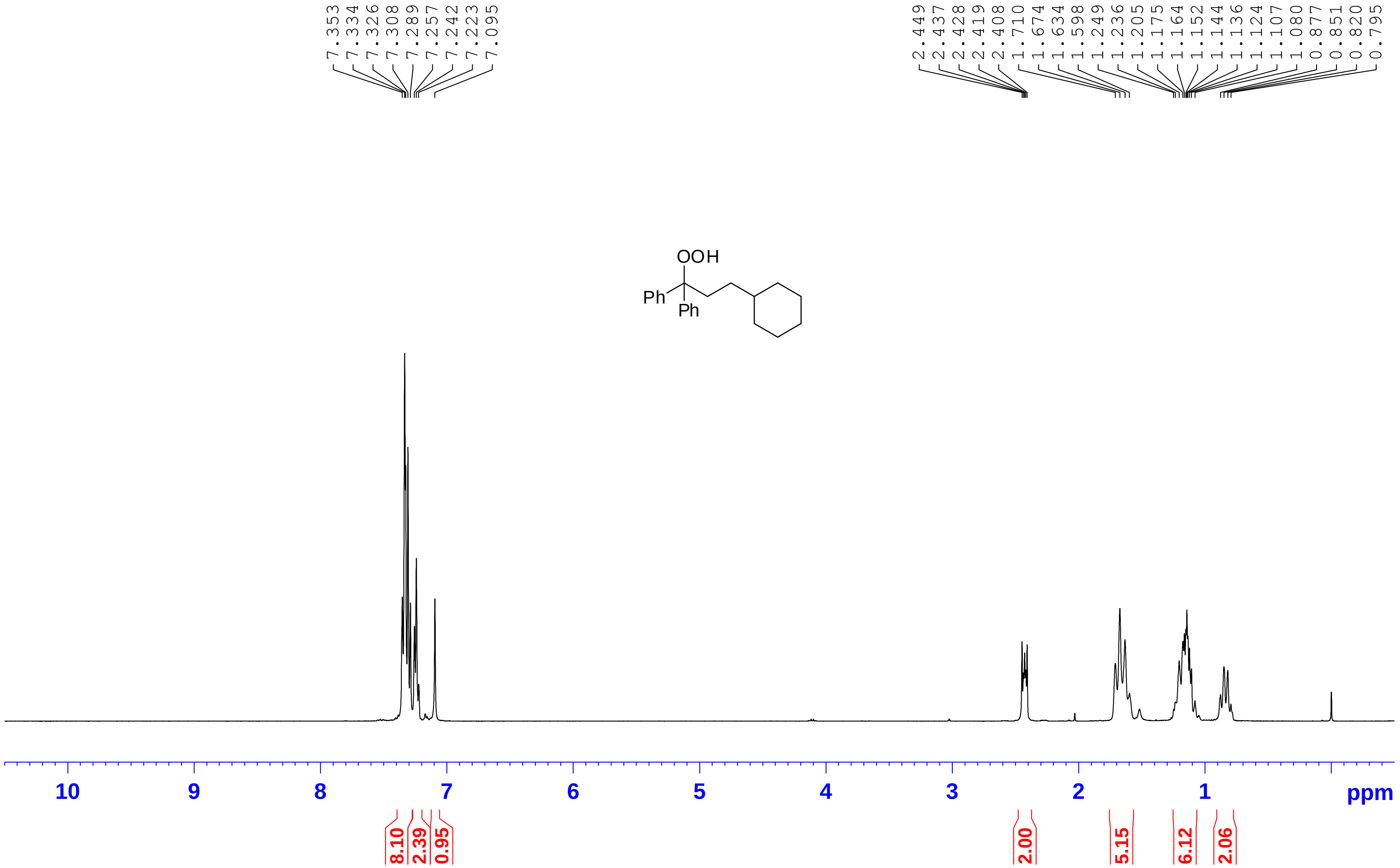

4. ^{1}H and ^{13}C NMR spectrum of new compounds

^{13}C NMR spectrum of **1r** (100 MHz, $CDCl_3$)

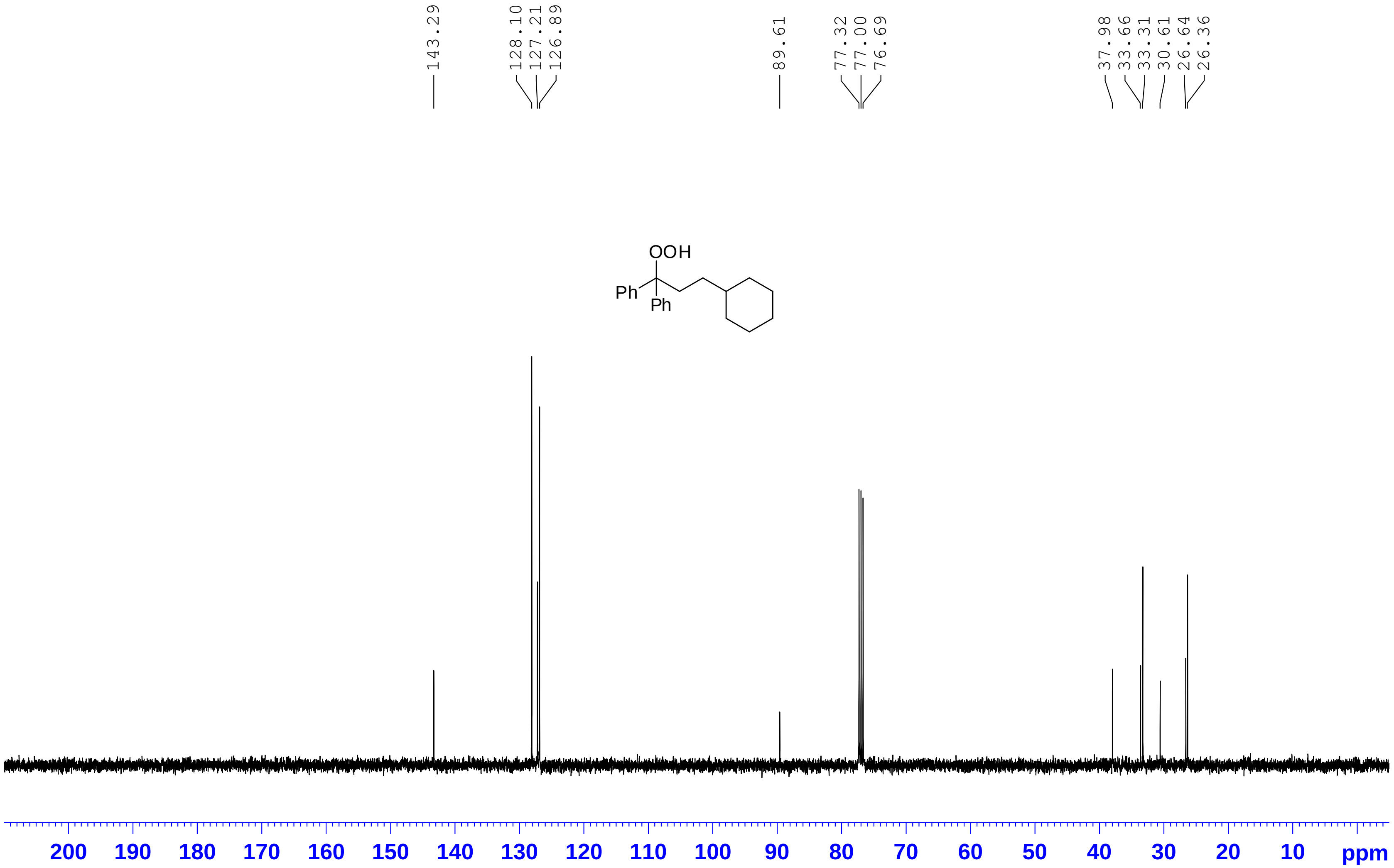

4. ^{1}H and ^{13}C NMR spectrum of new compounds

^{1}H NMR spectrum of **1s** (400 MHz, $CDCl_3$)

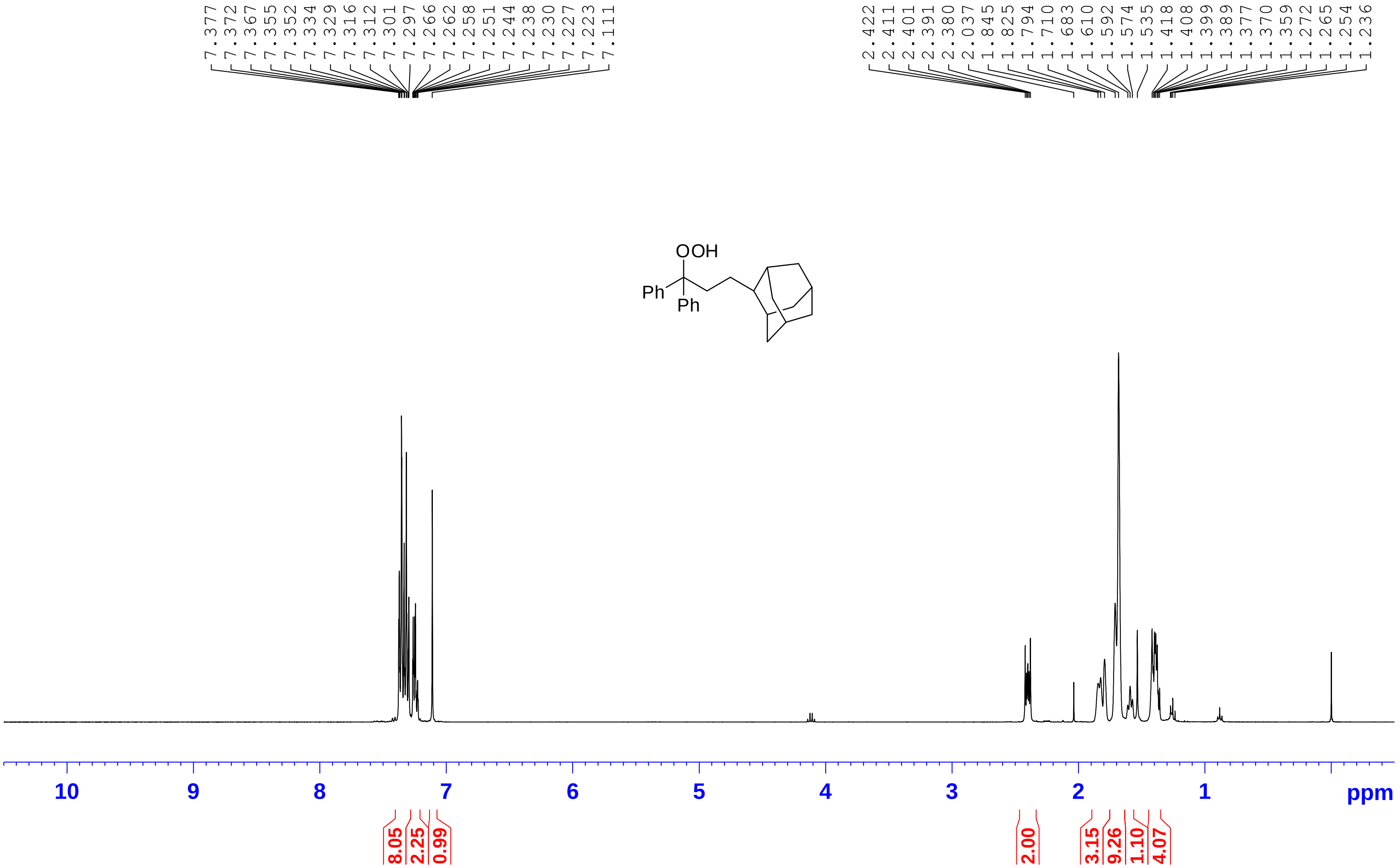

4. ^{1}H and ^{13}C NMR spectrum of new compounds

^{13}C NMR spectrum of **1s** (100 MHz, $CDCl_3$)

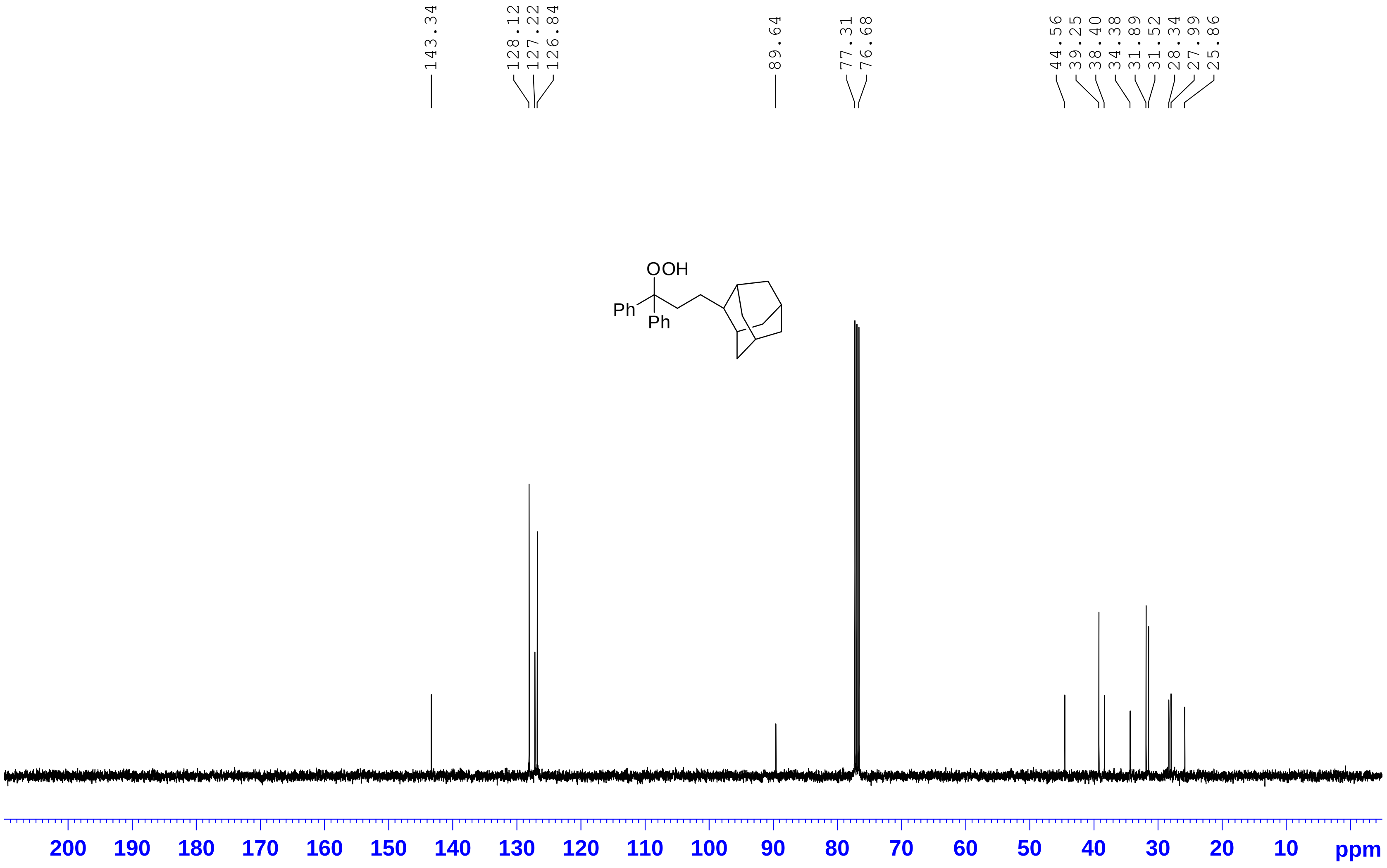

4. ^{1}H and ^{13}C NMR spectrum of new compounds

^{1}H NMR spectrum of **2i** (400 MHz, $CDCl_3$)

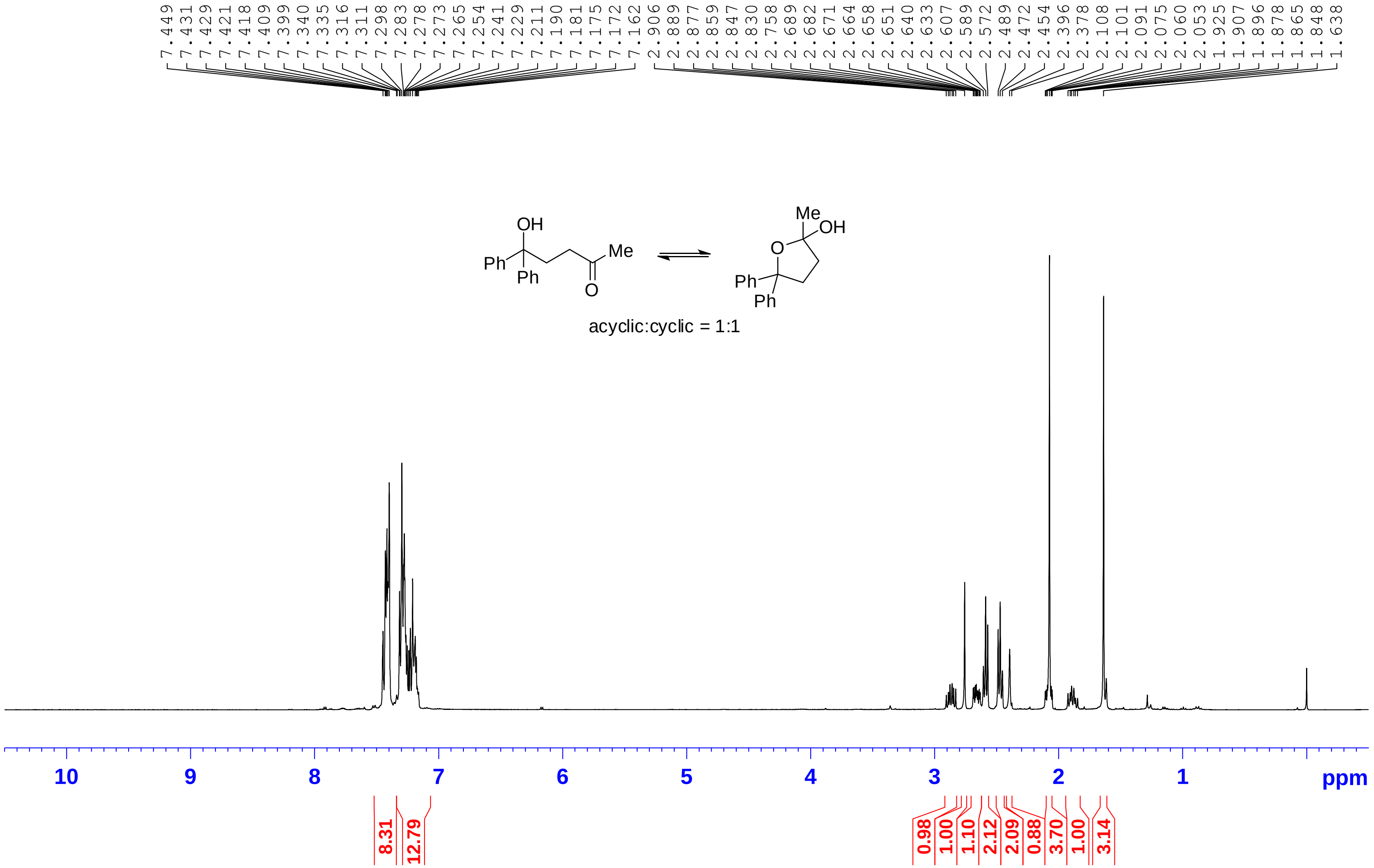

4. ^{1}H and ^{13}C NMR spectrum of new compounds

^{13}C NMR spectrum of **2i** (400 MHz, $CDCl_3$)

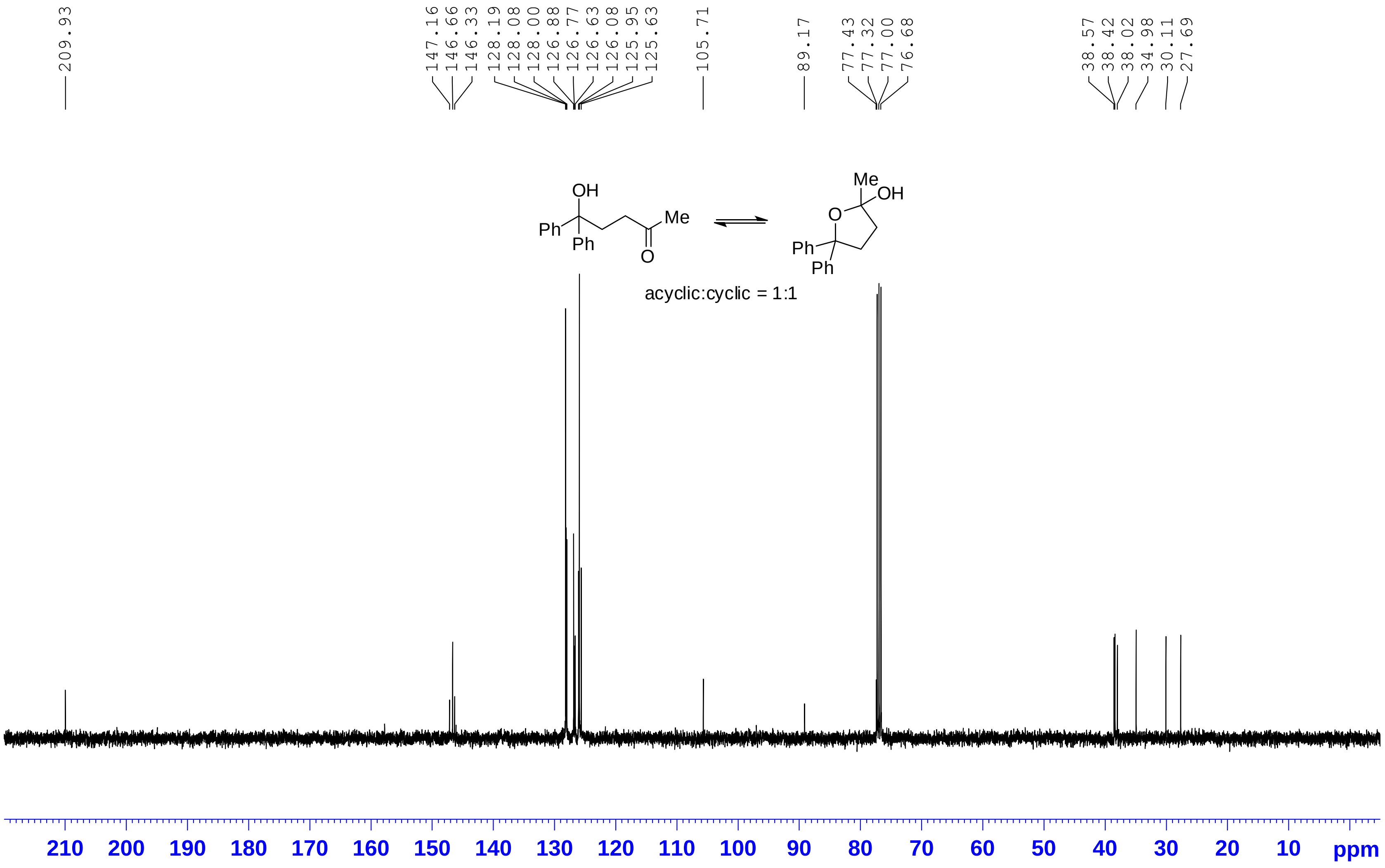

4. ^{1}H and ^{13}C NMR spectrum of new compounds

^{1}H NMR spectrum of **2o** (400 MHz, $CDCl_3$)

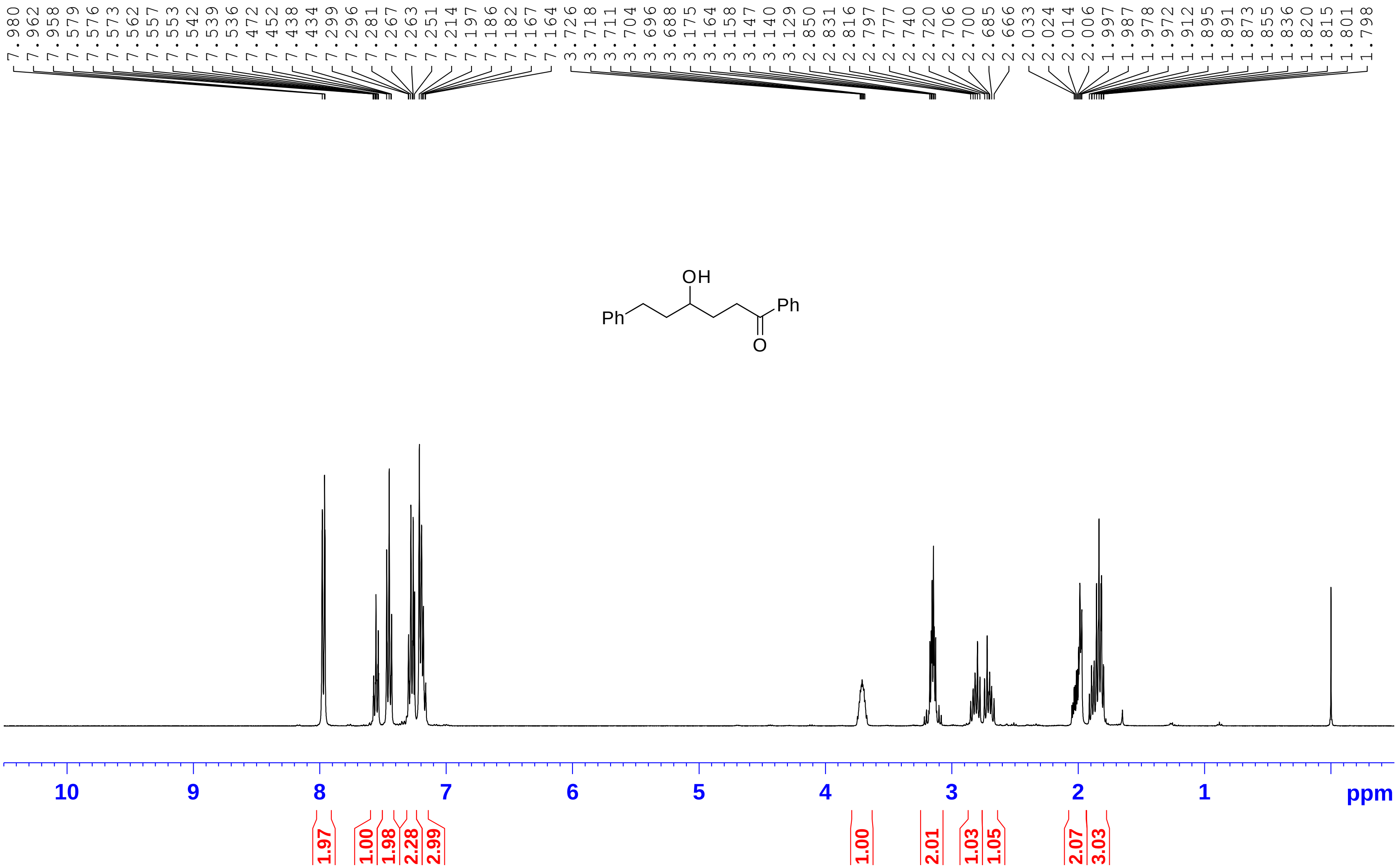

4. ^{1}H and ^{13}C NMR spectrum of new compounds

^{13}C NMR spectrum of **2o** (100 MHz, $CDCl_3$)

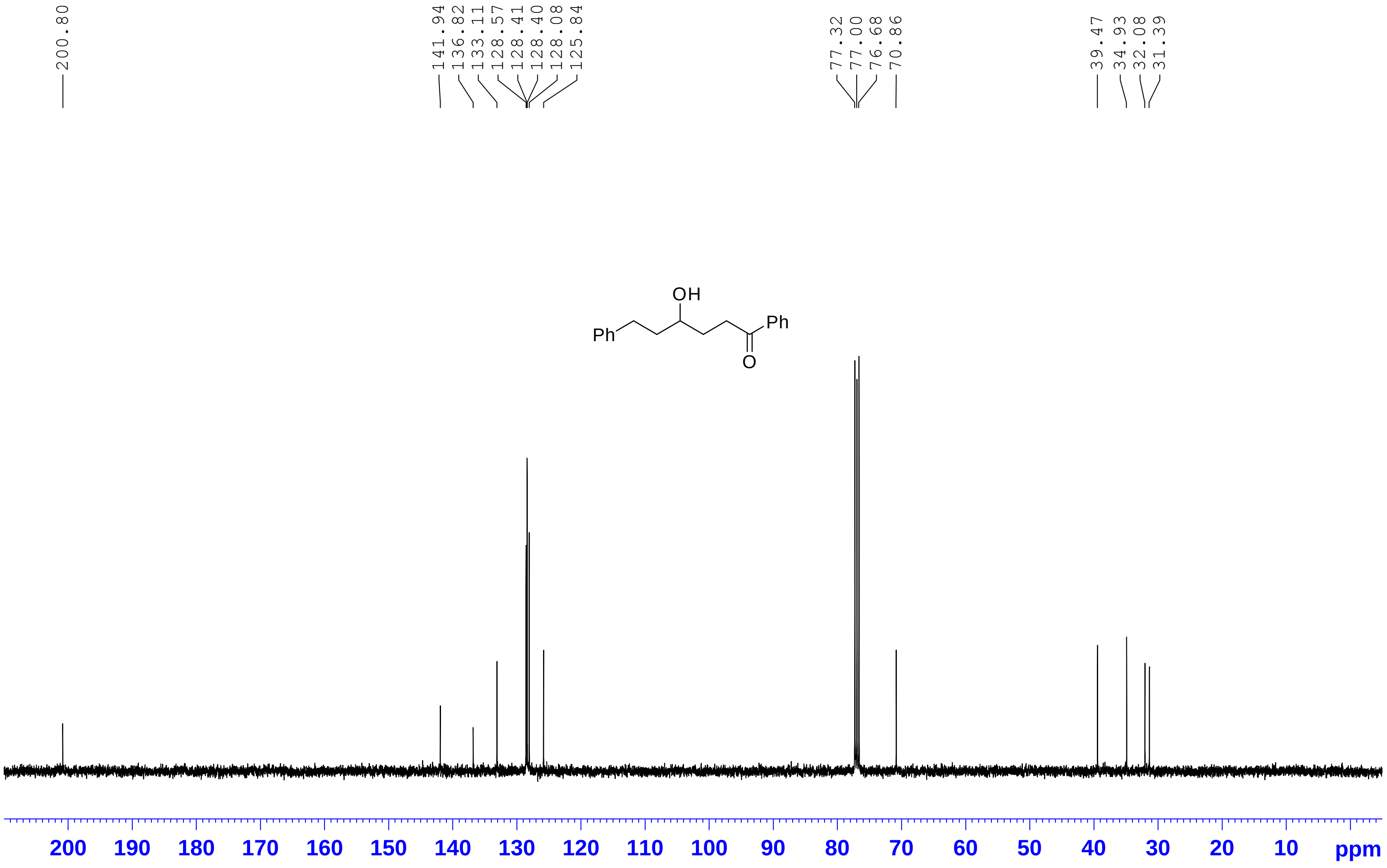

4. ^{1}H and ^{13}C NMR spectrum of new compounds

^{1}H NMR spectrum of **3a** (400 MHz, $CDCl_3$)

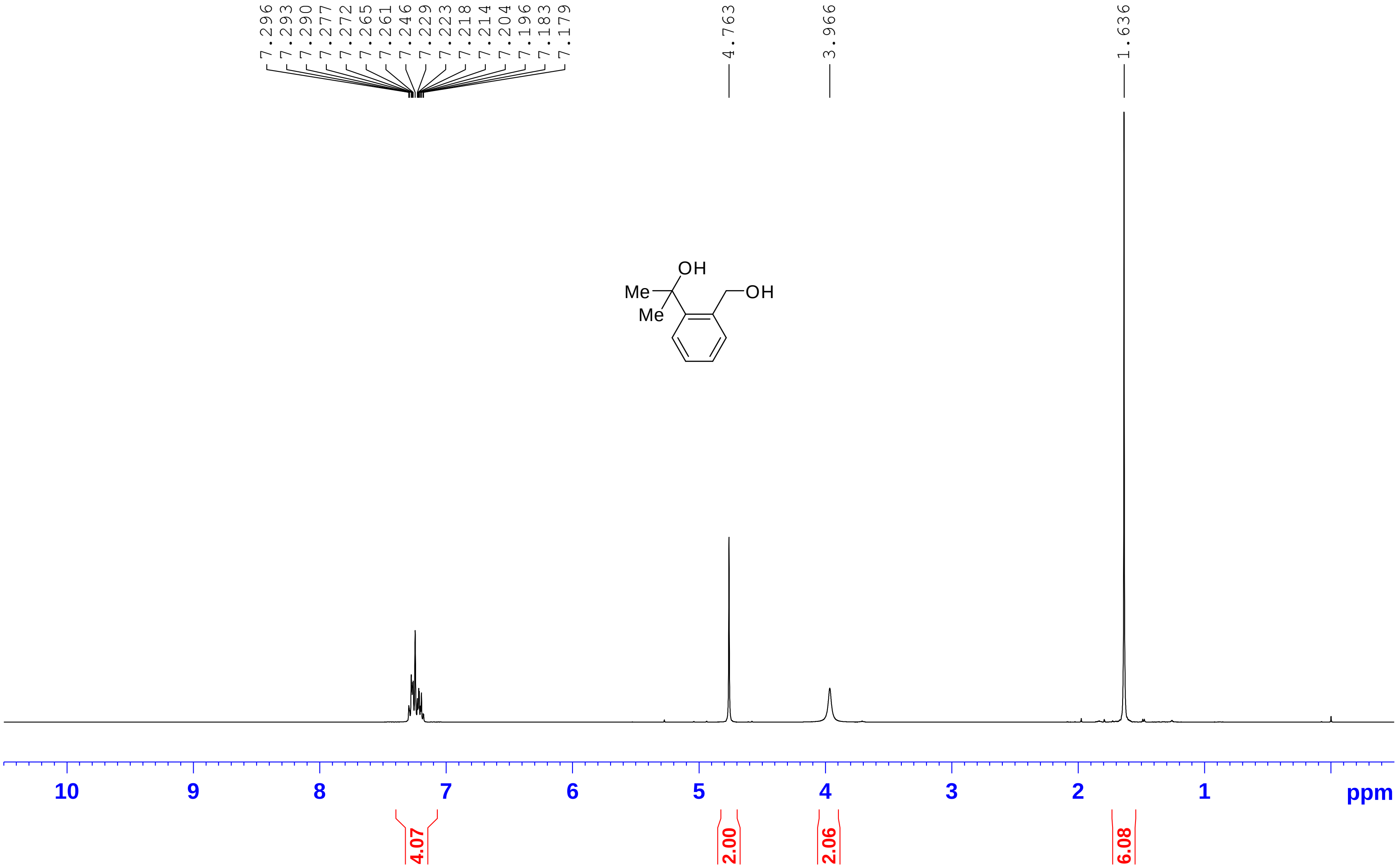

4. ^{1}H and ^{13}C NMR spectrum of new compounds

^{13}C NMR spectrum of **3a** (100 MHz, $CDCl_3$)

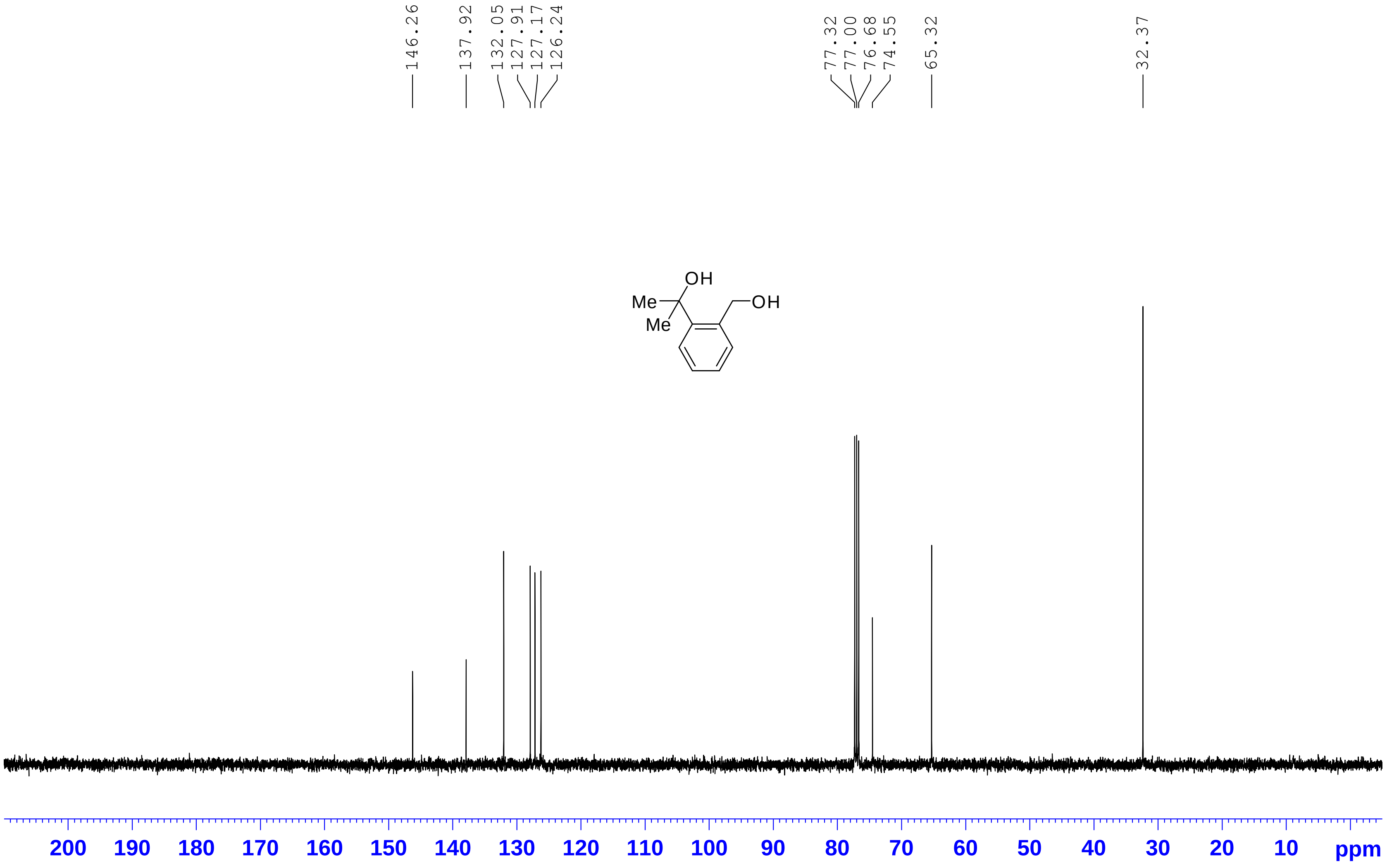

4. ^{1}H and ^{13}C NMR spectrum of new compounds

^{1}H NMR spectrum of **3b** (400 MHz, $CDCl_3$)

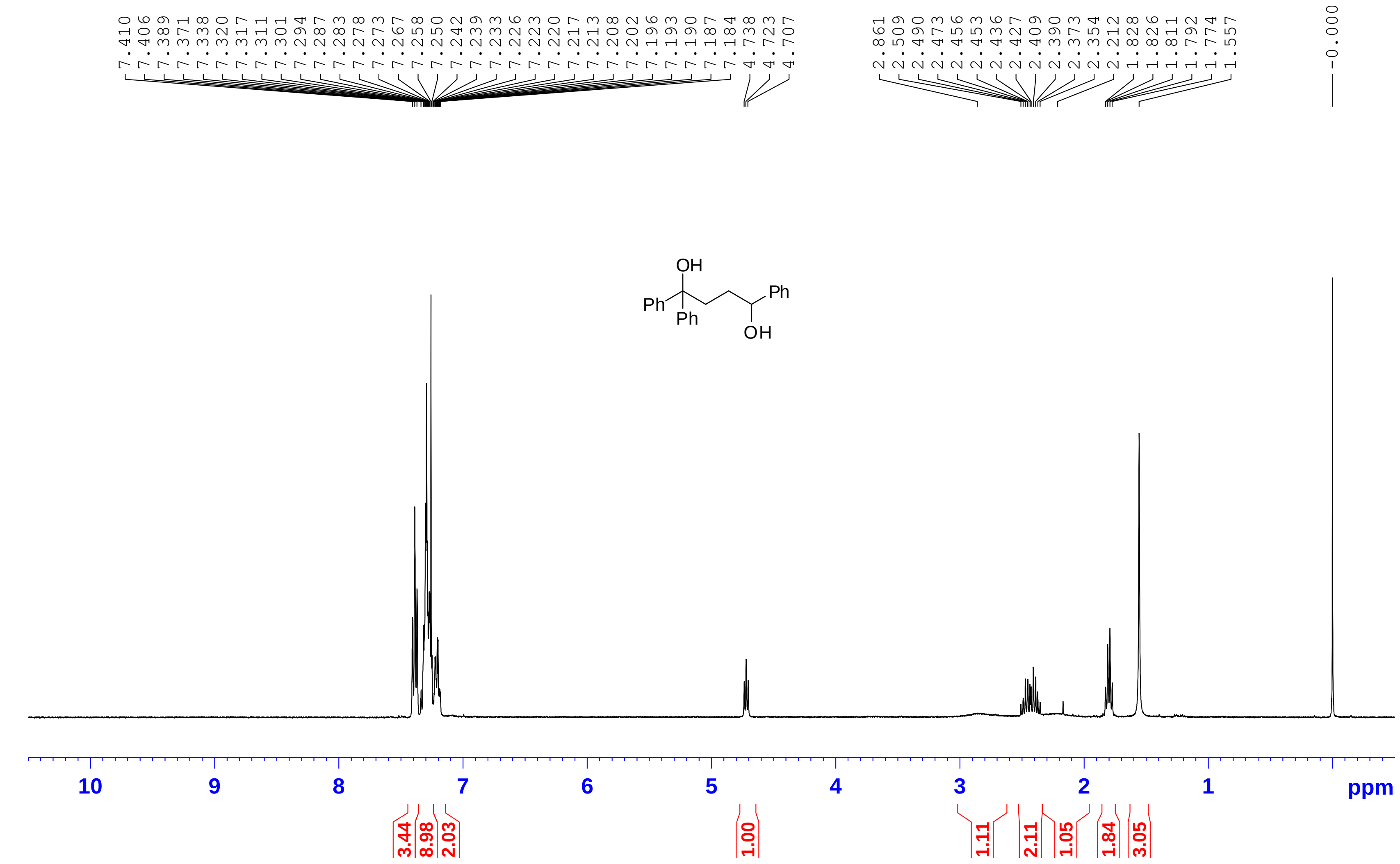

4. ^{1}H and ^{13}C NMR spectrum of new compounds

^{13}C NMR spectrum of **3b** (100 MHz, $CDCl_3$)

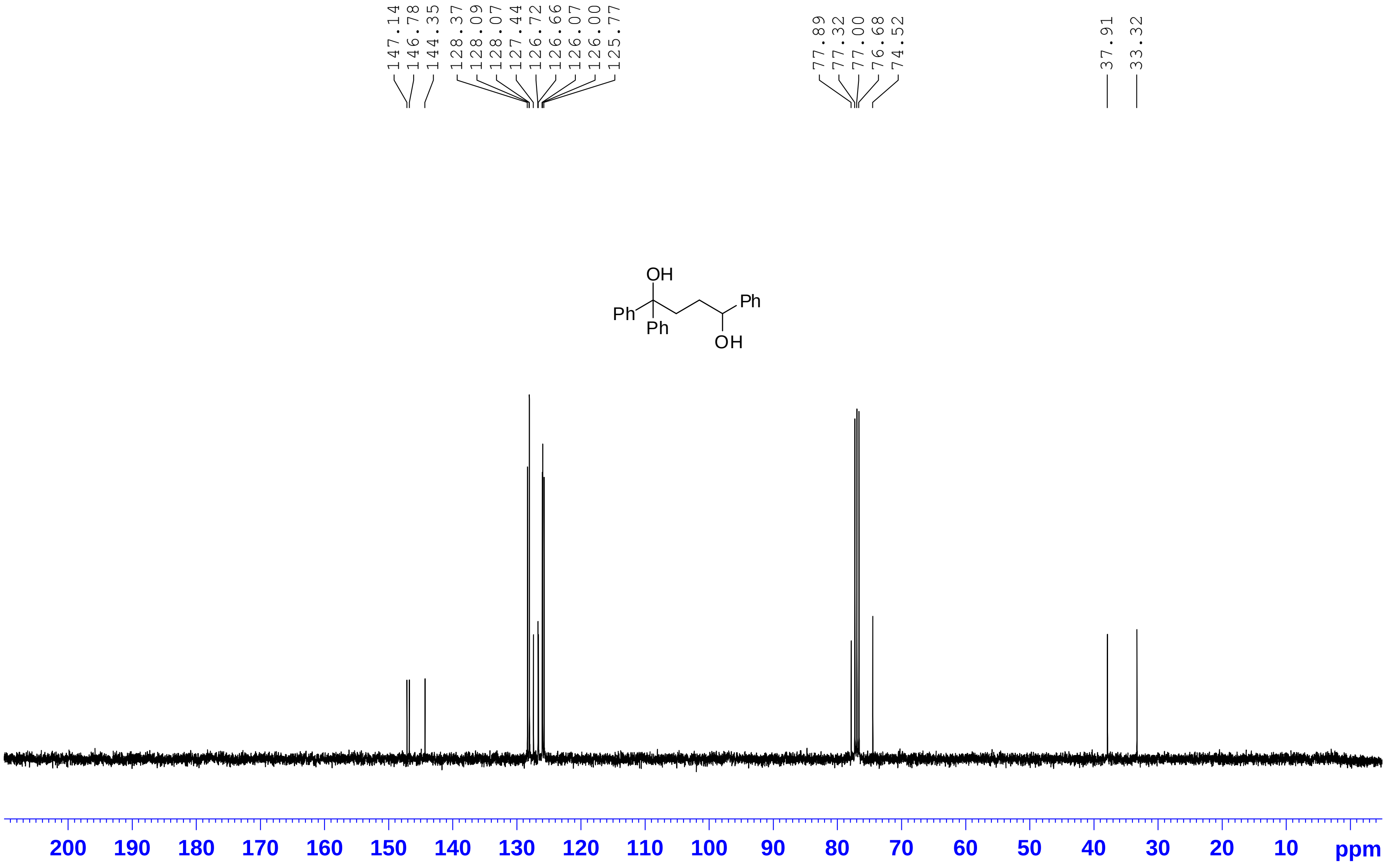

4. ^{1}H and ^{13}C NMR spectrum of new compounds

^{1}H NMR spectrum of **3c** (400 MHz, $CDCl_3$)

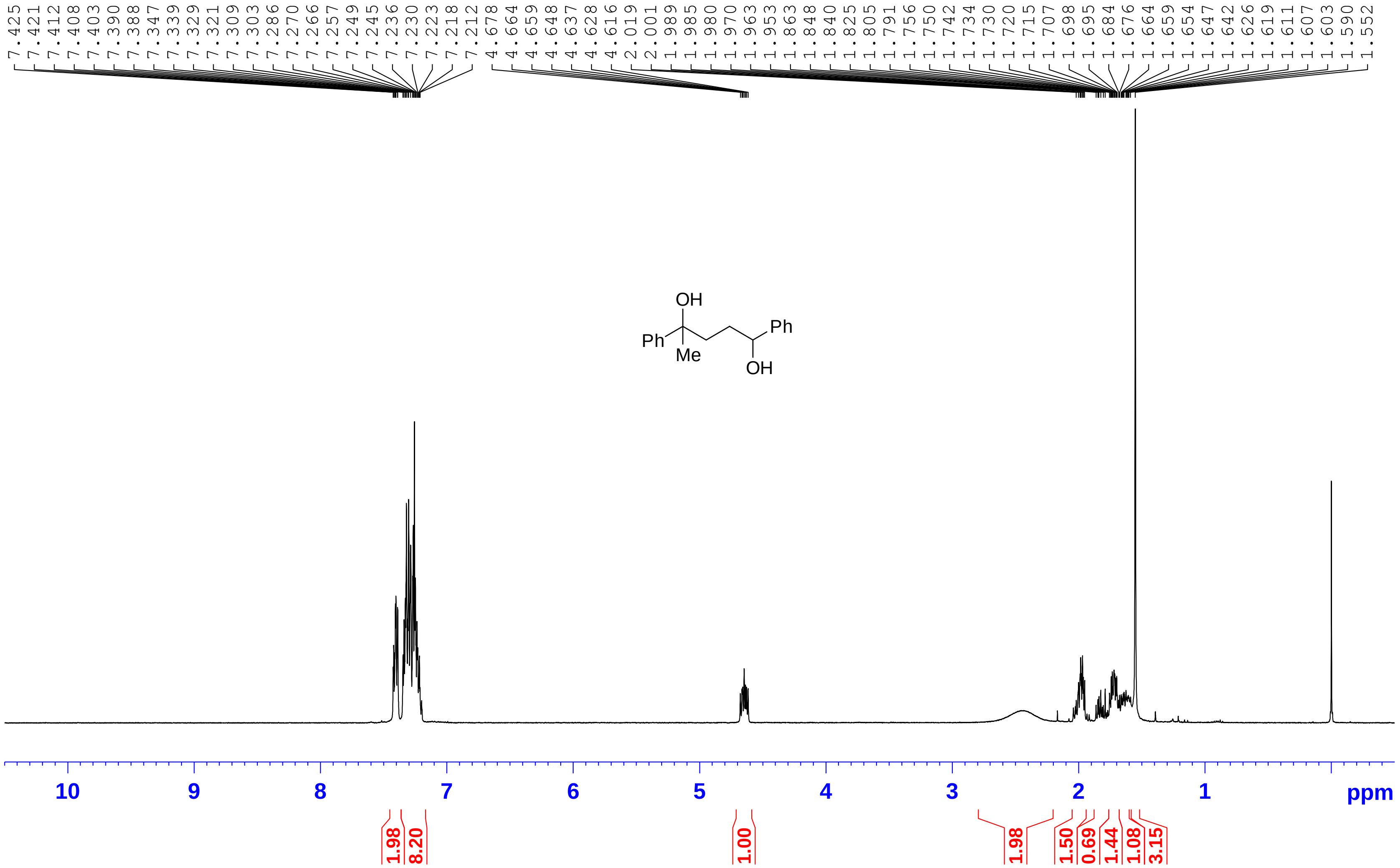

4. ^{1}H and ^{13}C NMR spectrum of new compounds

^{13}C NMR spectrum of **3c** (100 MHz, $CDCl_3$)

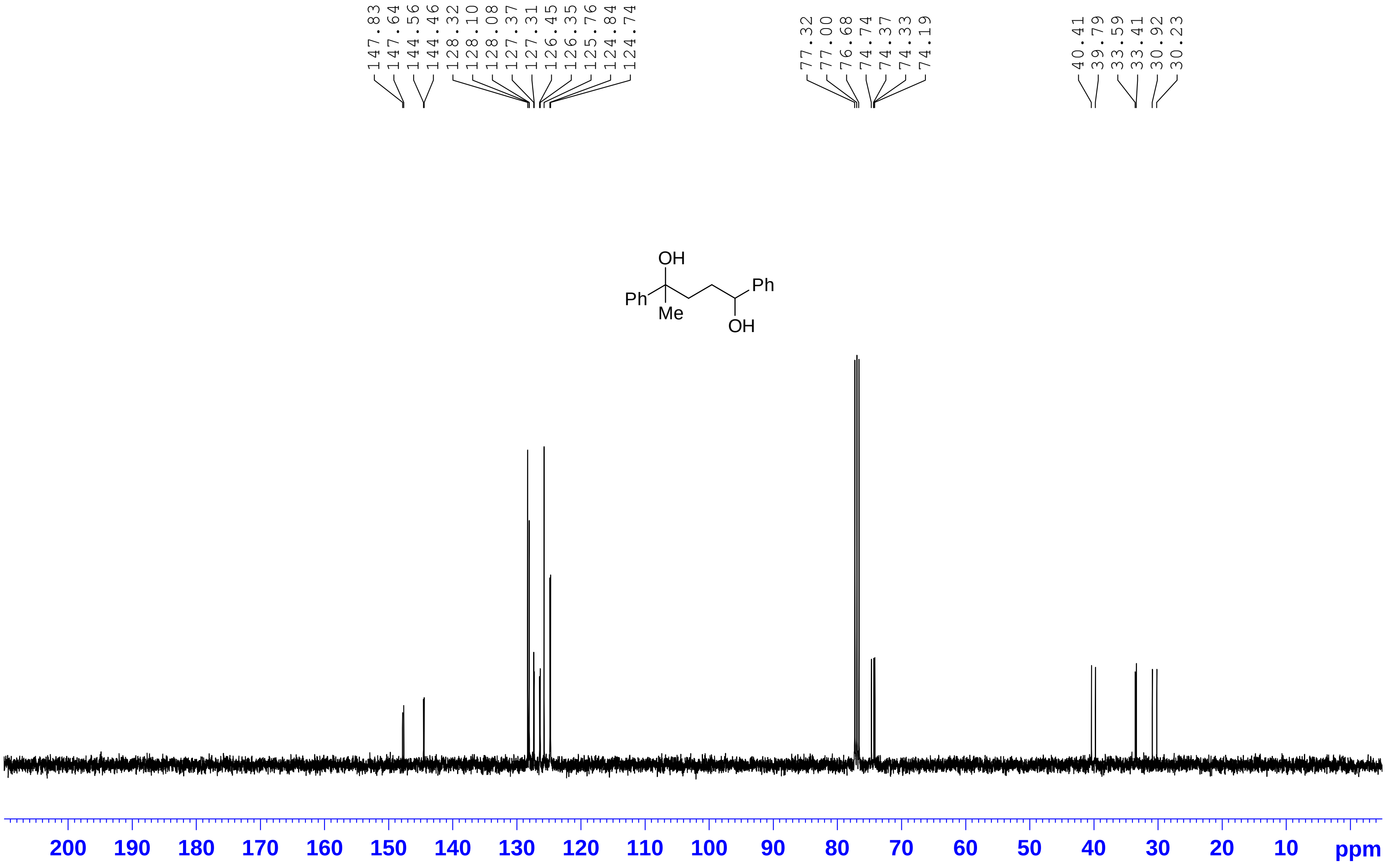

4. ¹H and ¹³C NMR spectrum of new compounds

^{1}H NMR spectrum of **3d** (400 MHz, $CDCl_3$)

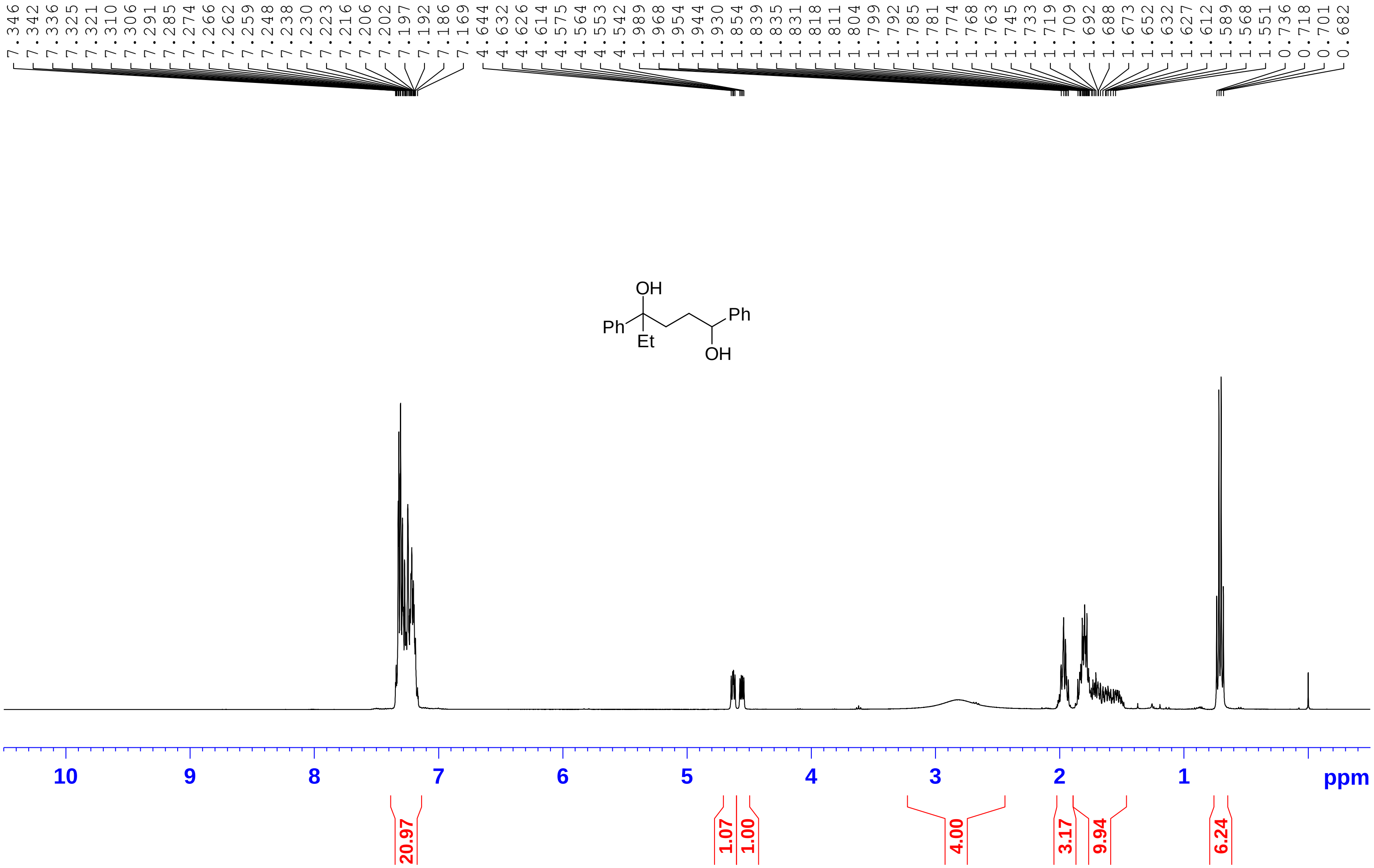

4. ^{1}H and ^{13}C NMR spectrum of new compounds

^{13}C NMR spectrum of **3d** (100 MHz, $CDCl_3$)

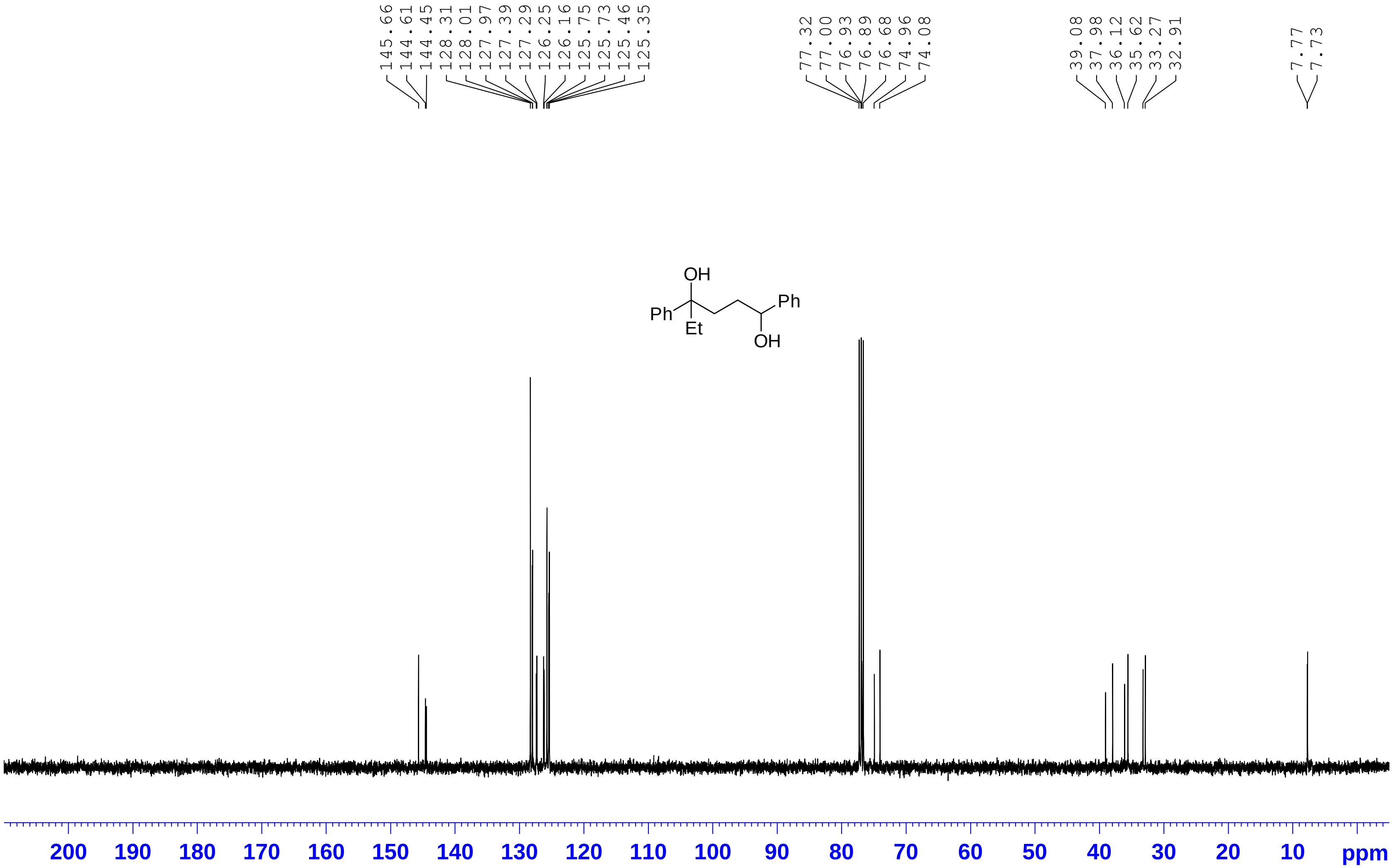

4. ^{1}H and ^{13}C NMR spectrum of new compounds

^{1}H NMR spectrum of **3f** (400 MHz, $CDCl_3$)

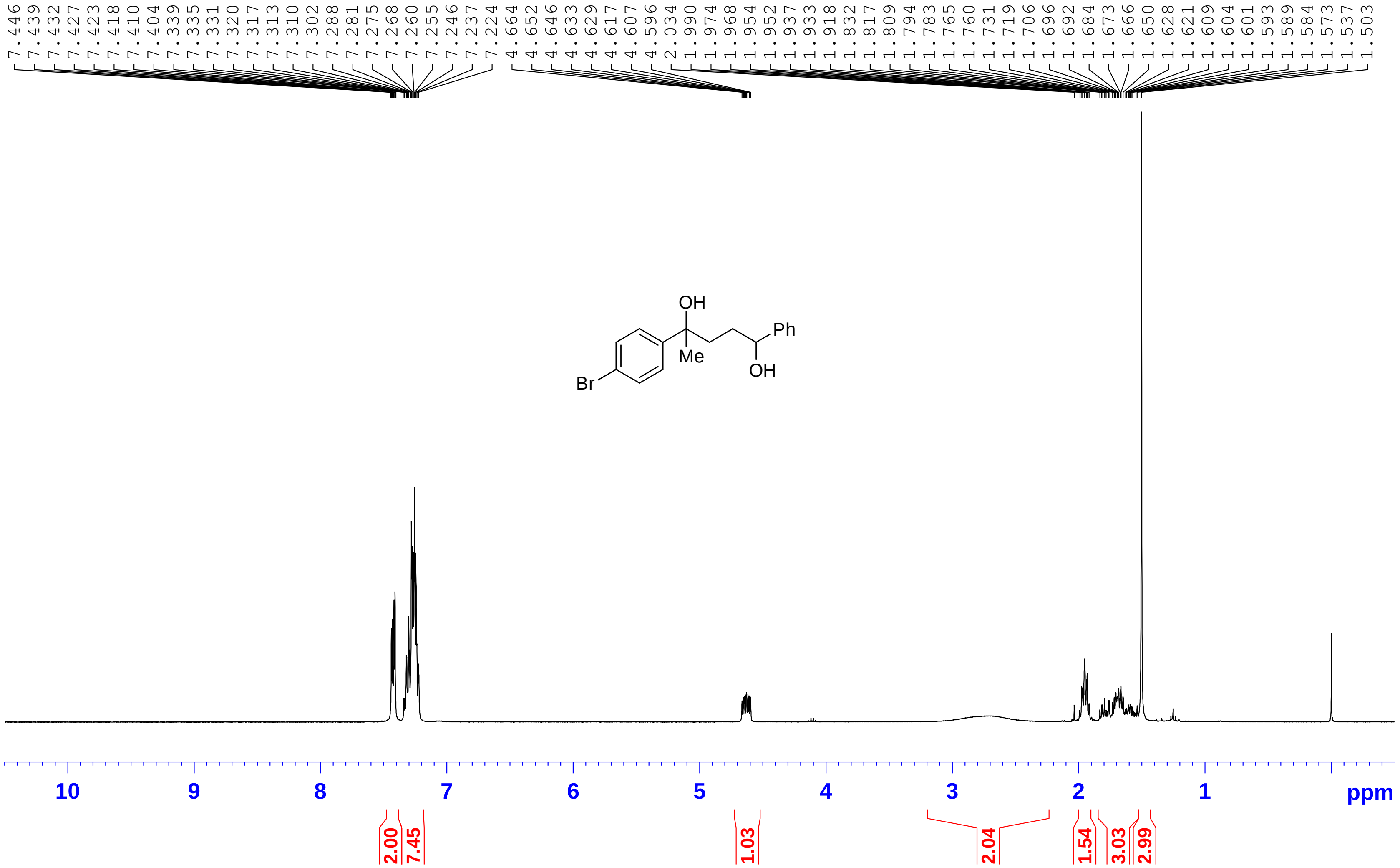

4. ^{1}H and ^{13}C NMR spectrum of new compounds

^{13}C NMR spectrum of **3f** (100 MHz, $CDCl_3$)

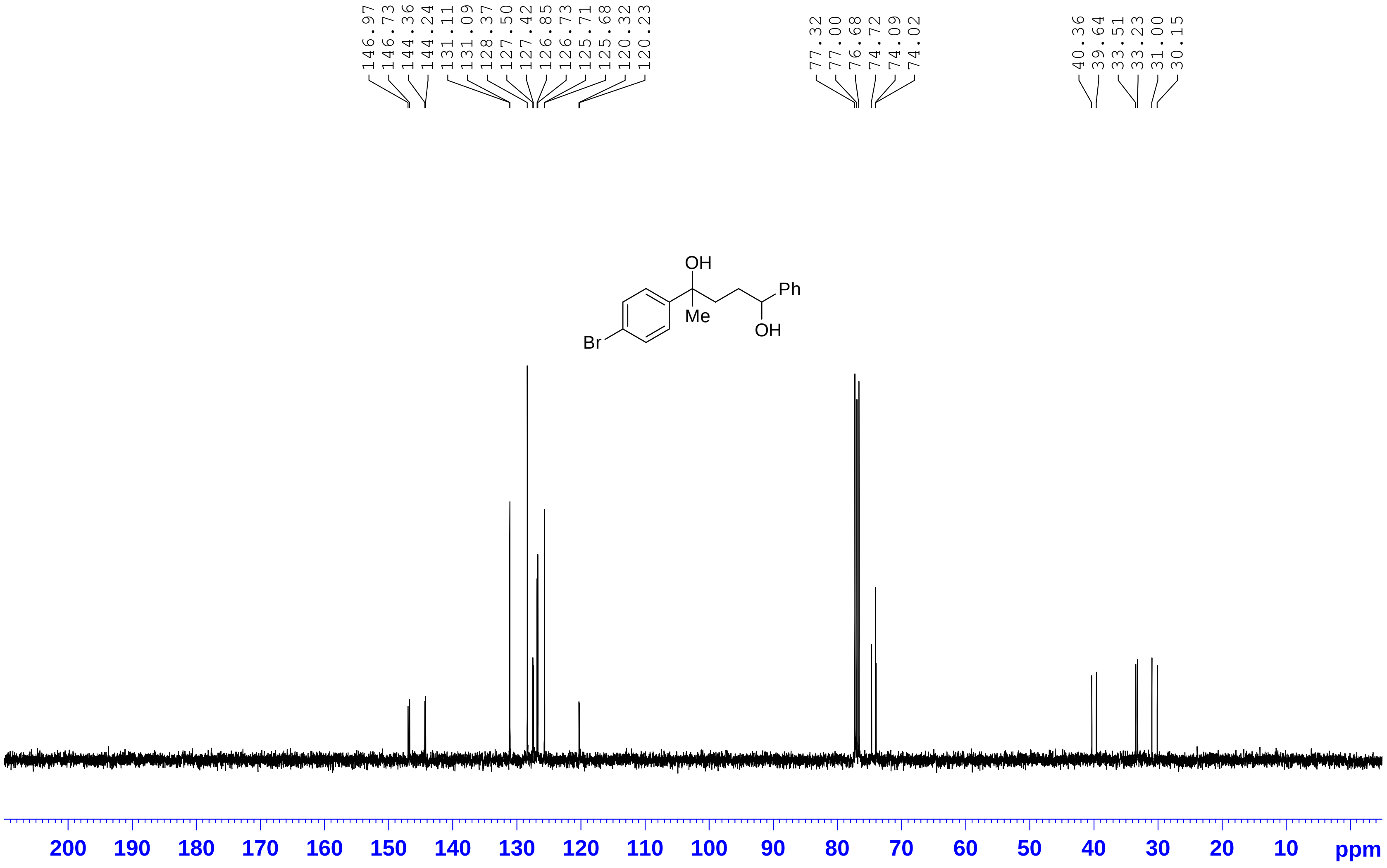

4. ^{1}H and ^{13}C NMR spectrum of new compounds

^{1}H NMR spectrum of **3g** (400 MHz, $CDCl_3$)

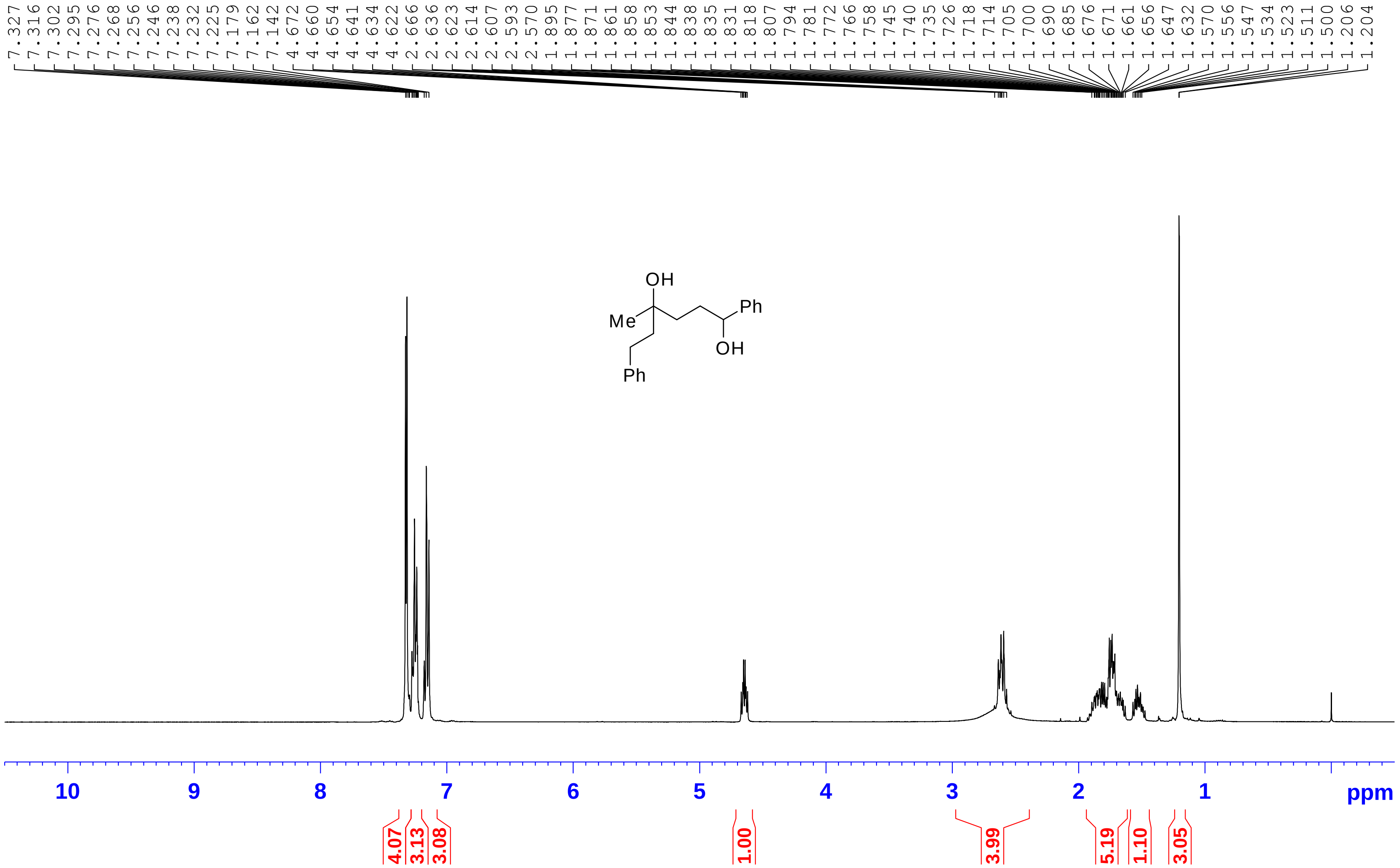

4. ^{1}H and ^{13}C NMR spectrum of new compounds

^{13}C NMR spectrum of **3g** (100 MHz, $CDCl_3$)

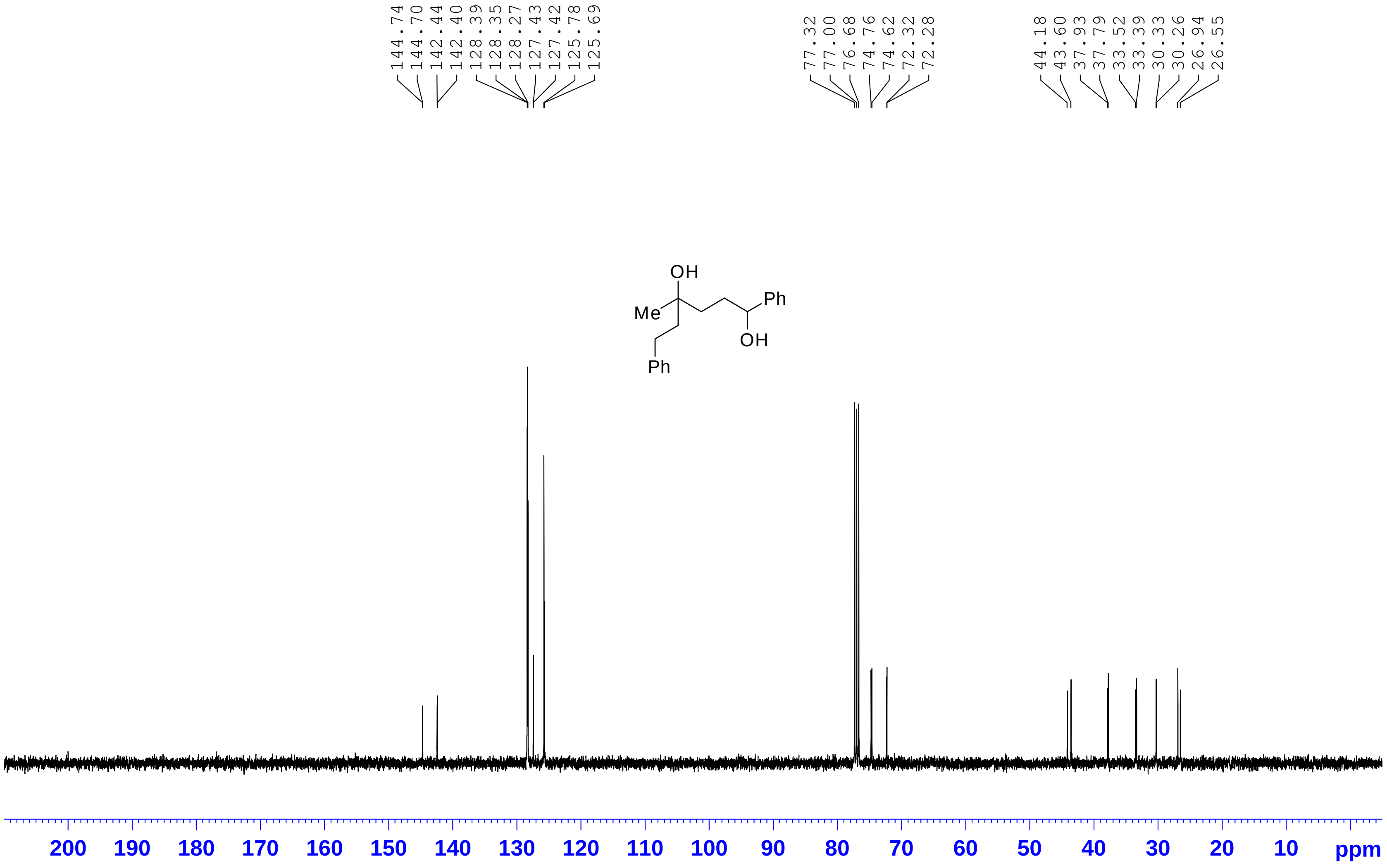

4. ^{1}H and ^{13}C NMR spectrum of new compounds

^{1}H NMR spectrum of **3h** (400 MHz, $CDCl_3$)

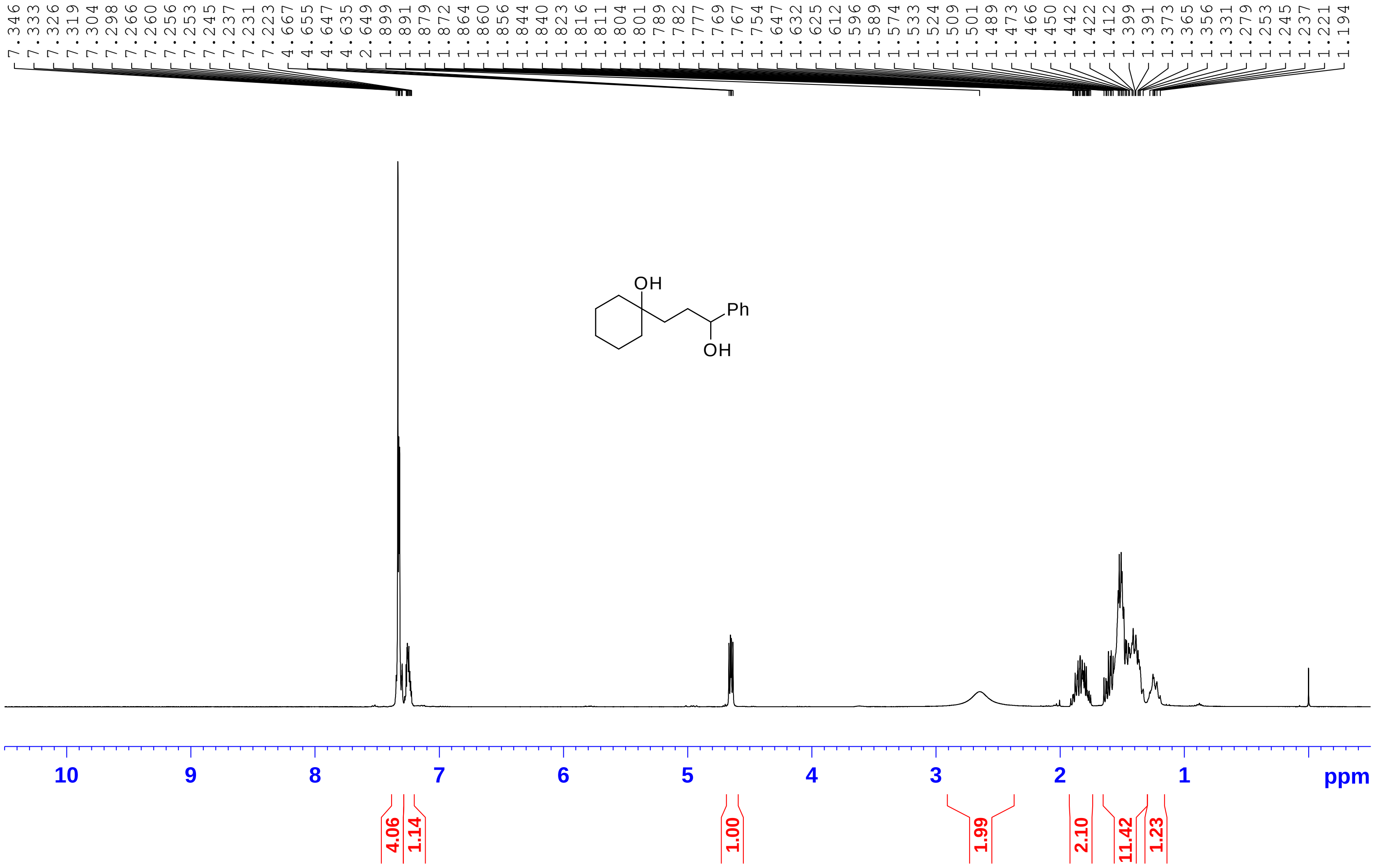

4. ^{1}H and ^{13}C NMR spectrum of new compounds

^{13}C NMR spectrum of **3h** (100 MHz, $CDCl_3$)

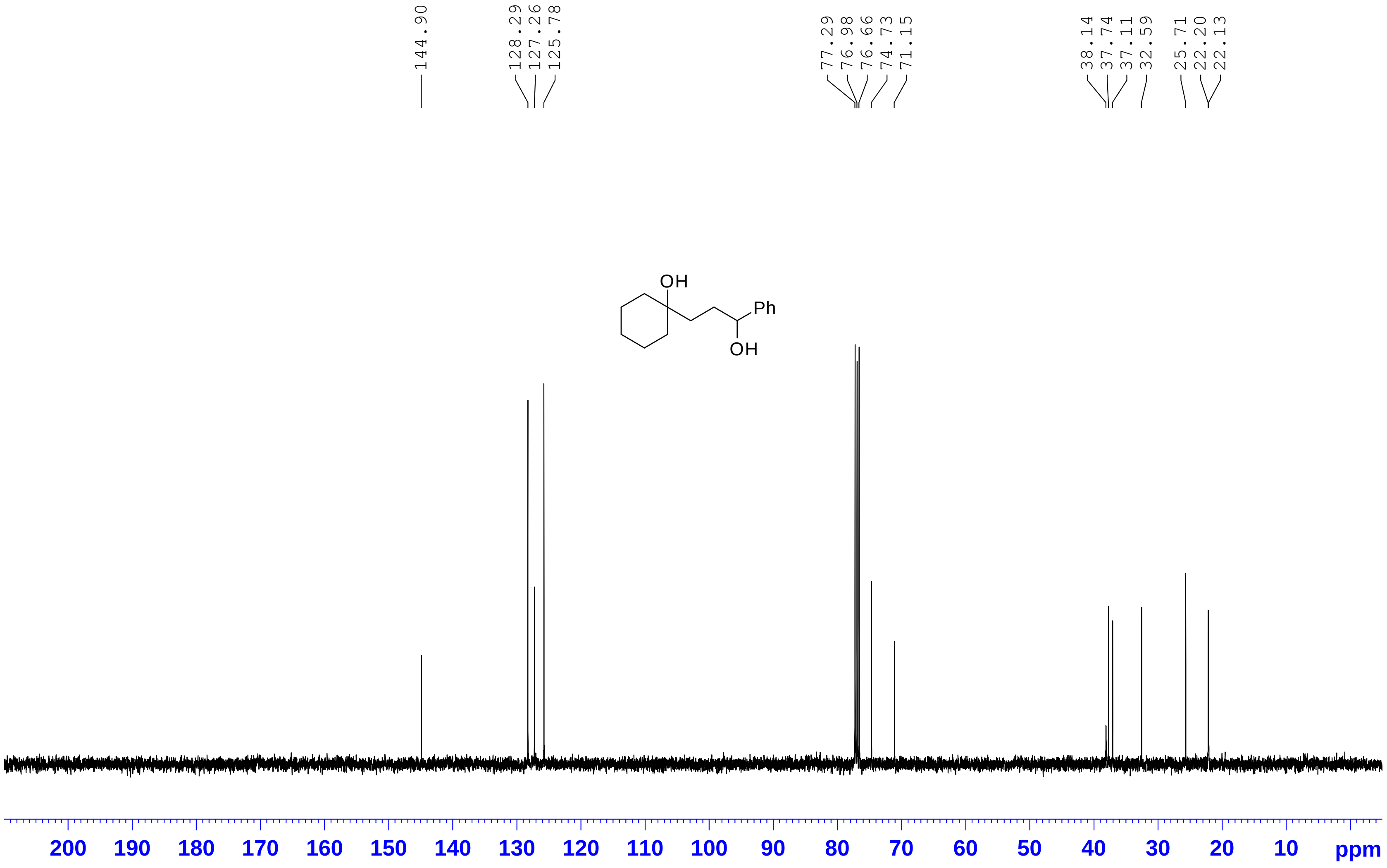

4. ^{1}H and ^{13}C NMR spectrum of new compounds

^{1}H NMR spectrum of **3i** (400 MHz, $CDCl_3$)

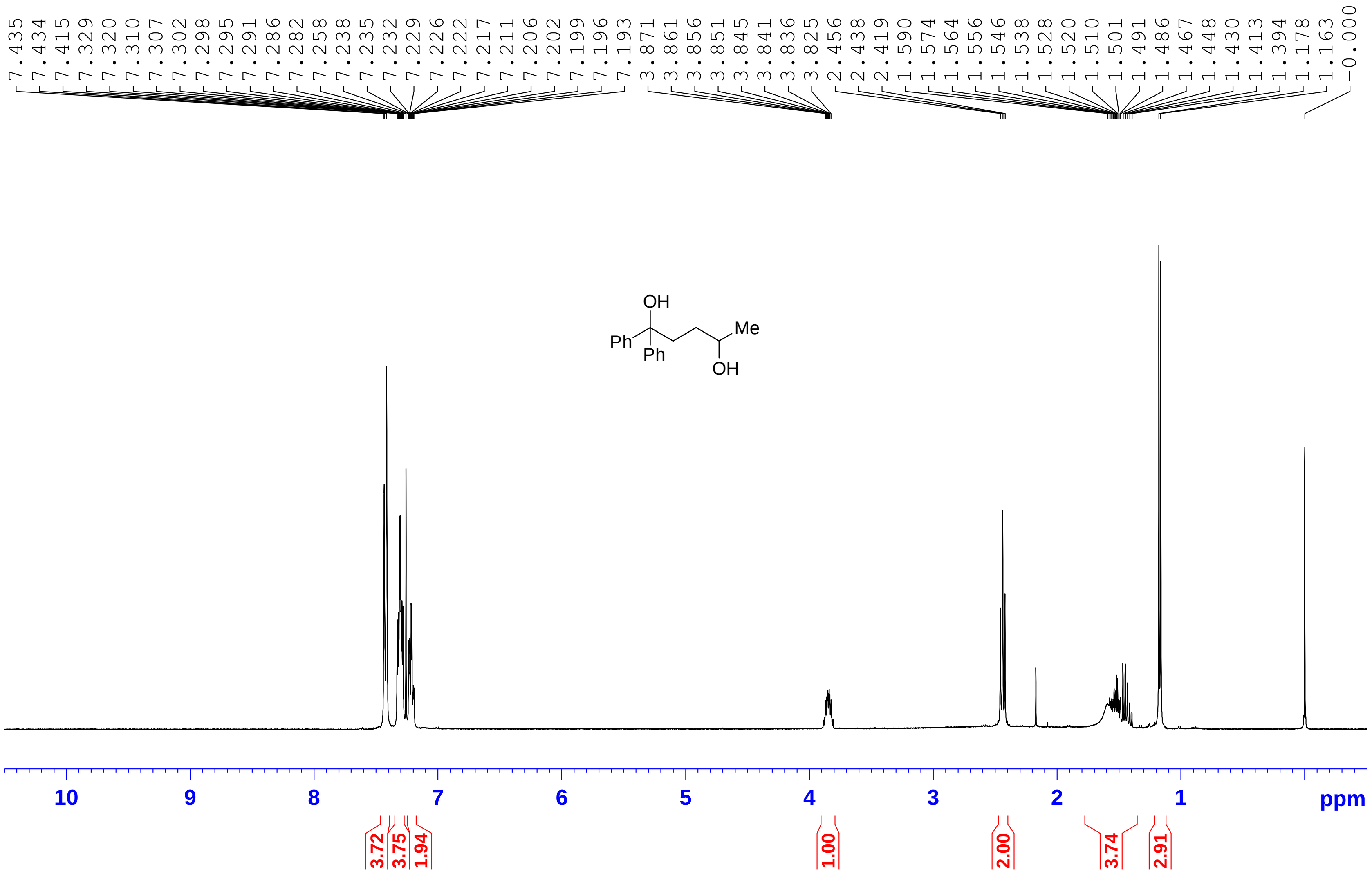

4. ^{1}H and ^{13}C NMR spectrum of new compounds

^{13}C NMR spectrum of **3i** (100 MHz, $CDCl_3$)

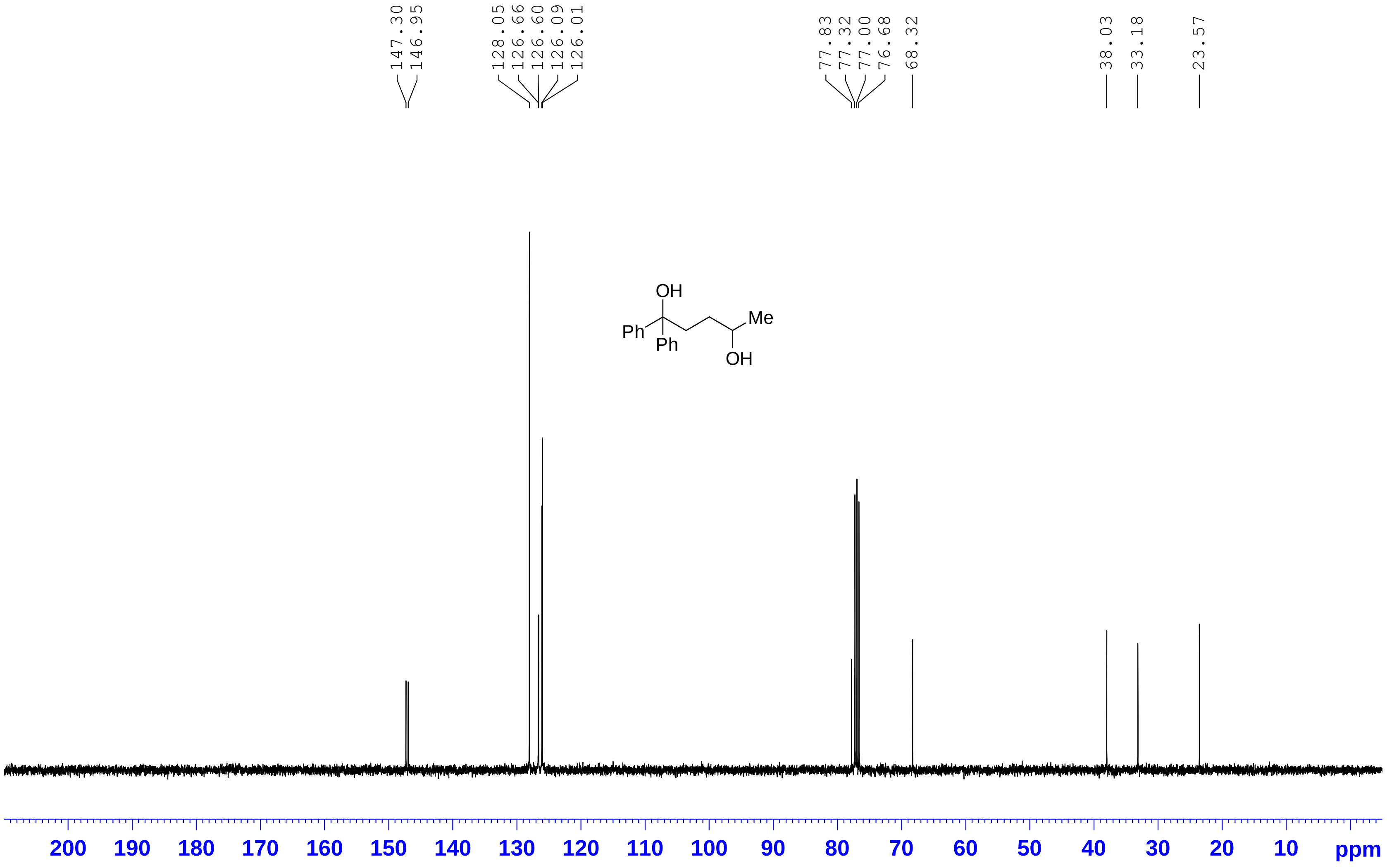

4. ^{1}H and ^{13}C NMR spectrum of new compounds

^{1}H NMR spectrum of **3j** (400 MHz, $CDCl_3$)

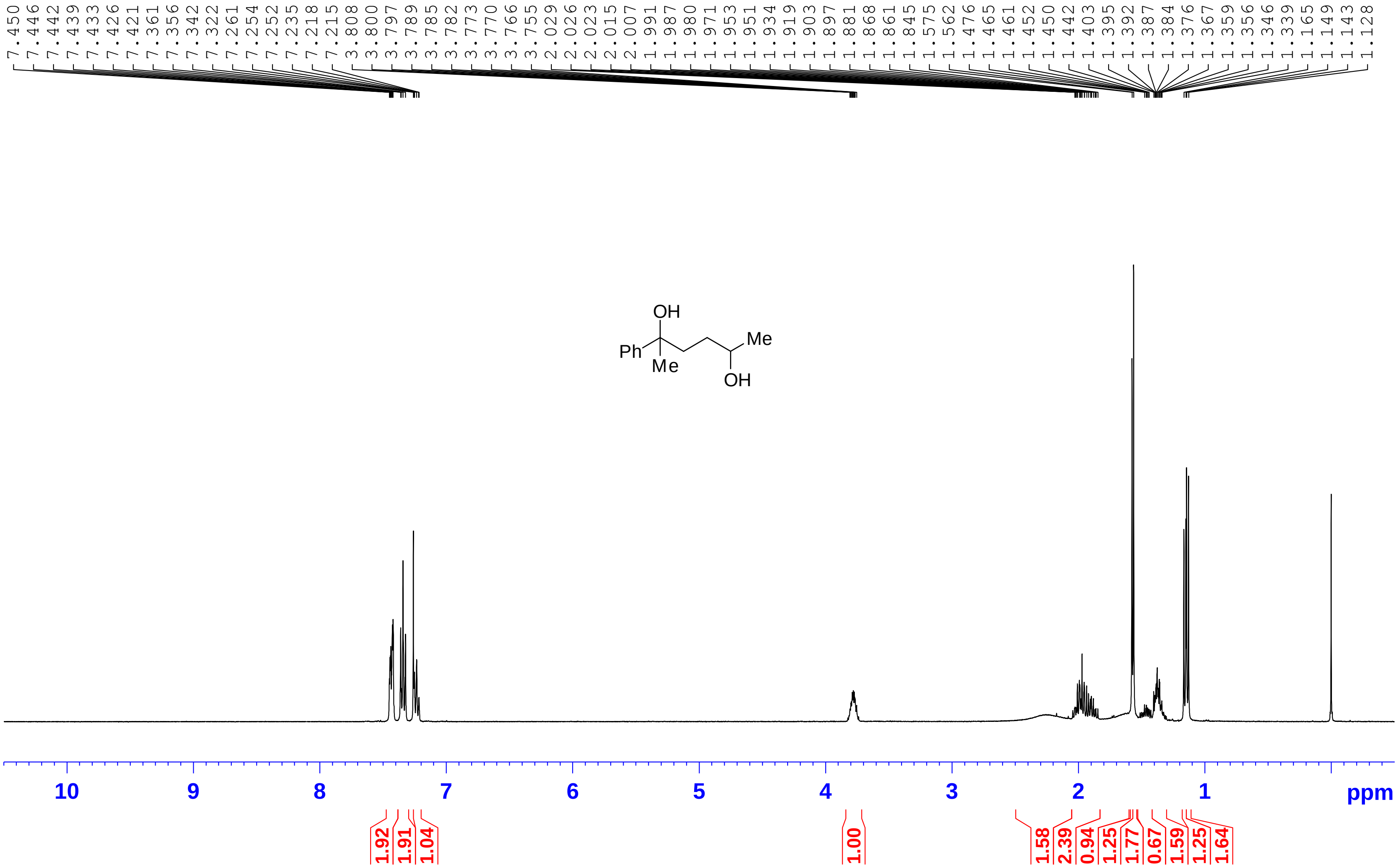

4. ^{1}H and ^{13}C NMR spectrum of new compounds

^{13}C NMR spectrum of **3j** (100 MHz, $CDCl_3$)

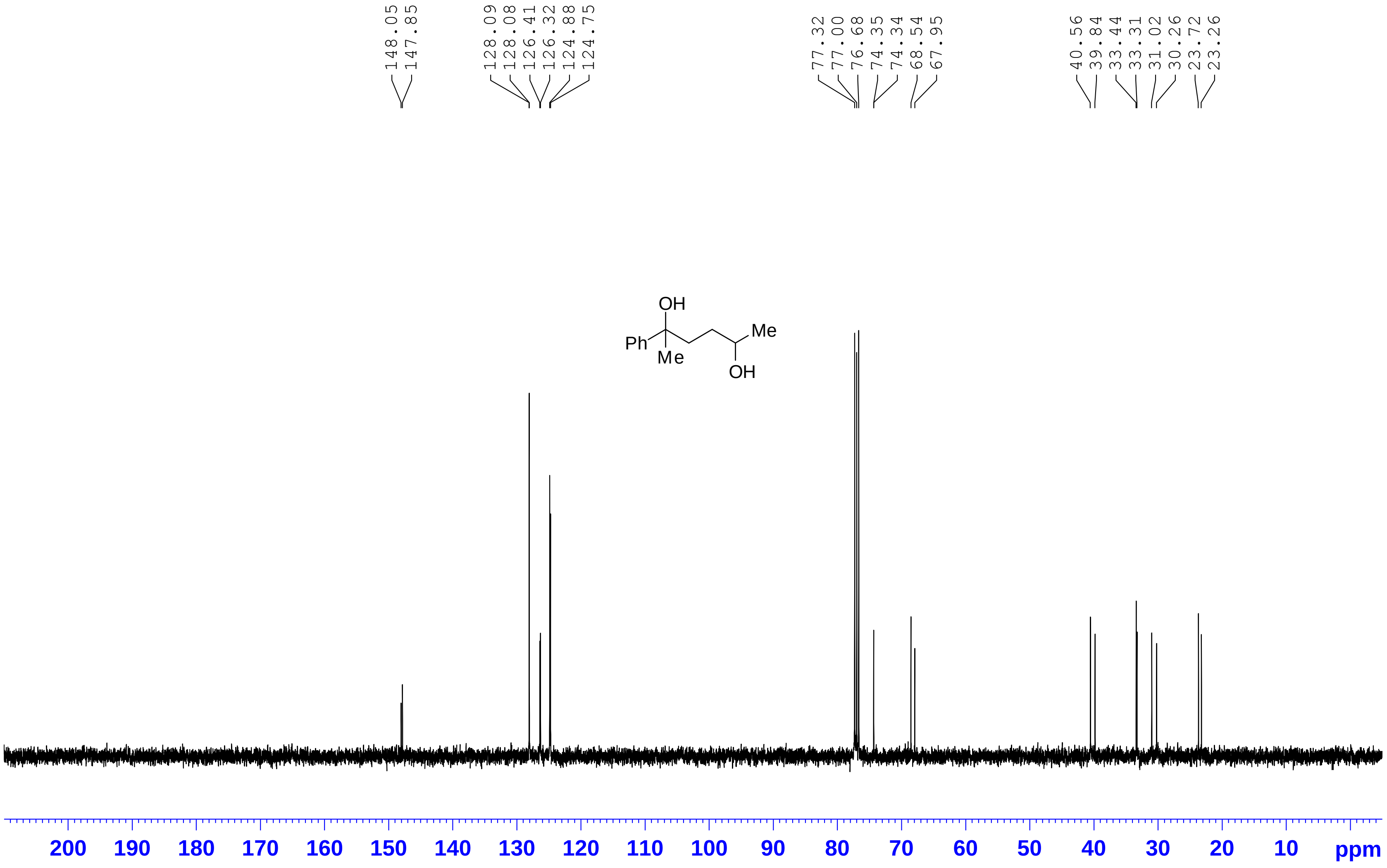

4. ^{1}H and ^{13}C NMR spectrum of new compounds

^{1}H NMR spectrum of **3k** (400 MHz, $CDCl_3$)

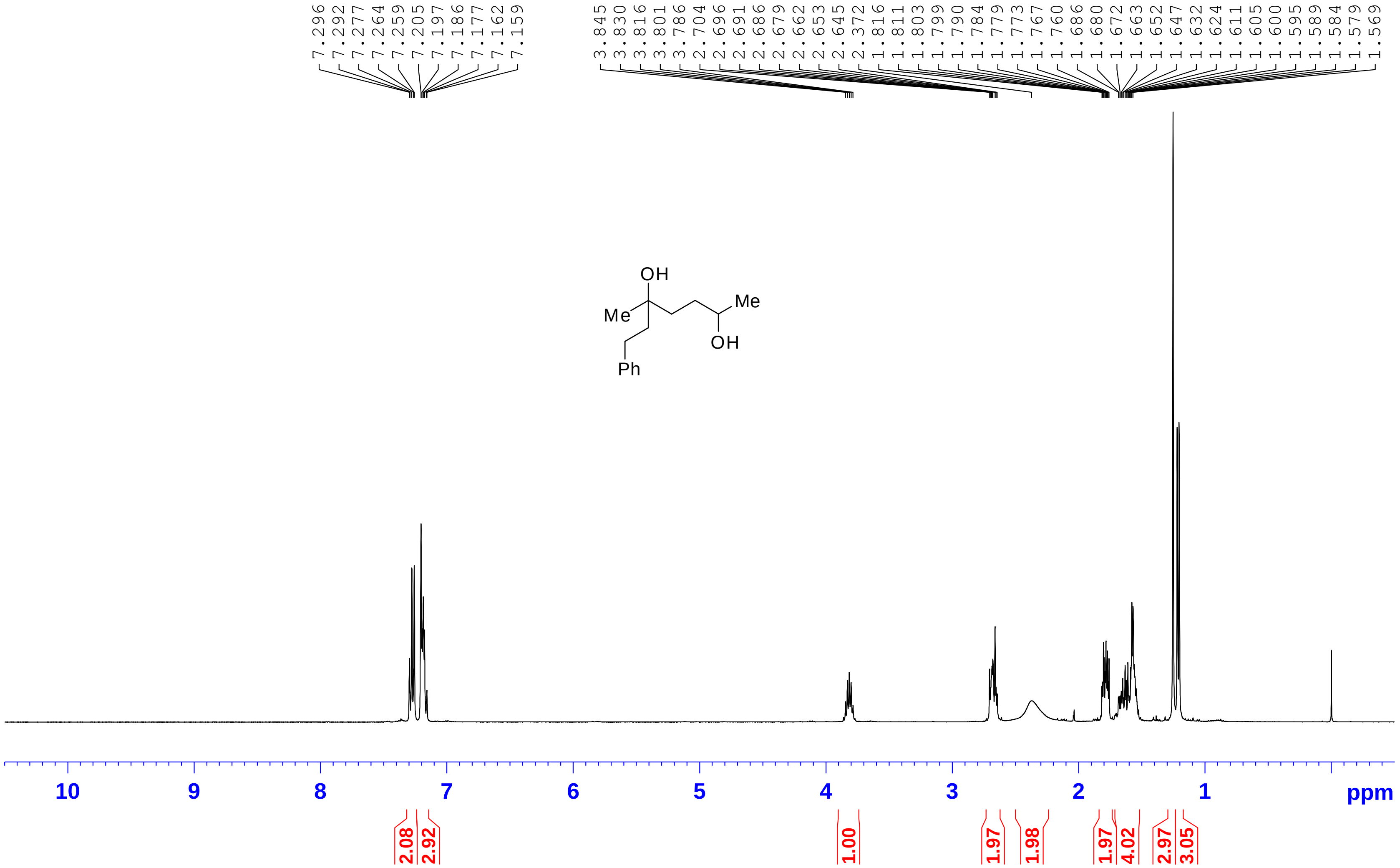

4. ^{1}H and ^{13}C NMR spectrum of new compounds

^{13}C NMR spectrum of **3k** (100 MHz, $CDCl_3$)

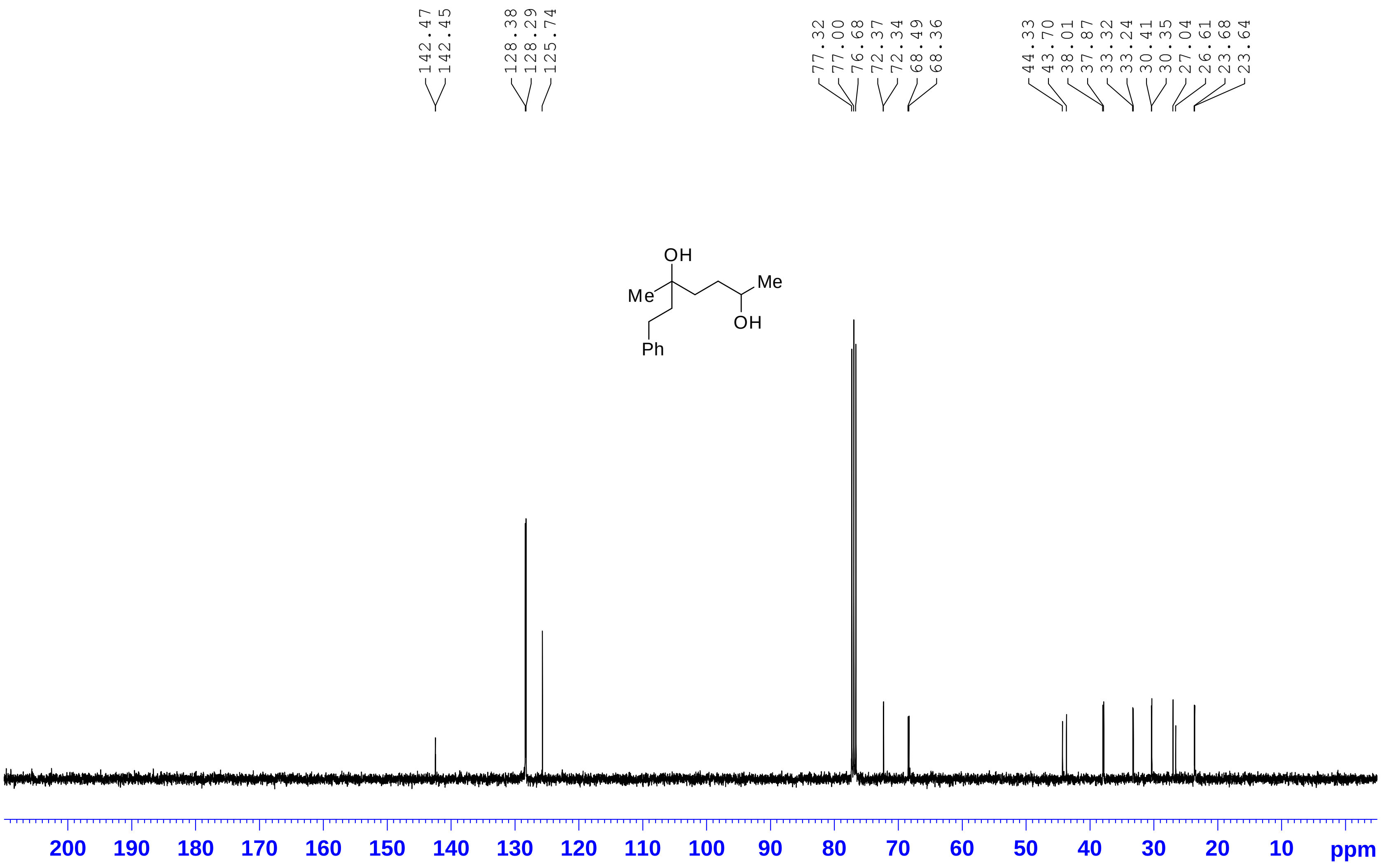

4. ^{1}H and ^{13}C NMR spectrum of new compounds

^{1}H NMR spectrum of **3l** (400 MHz, $CDCl_3$)

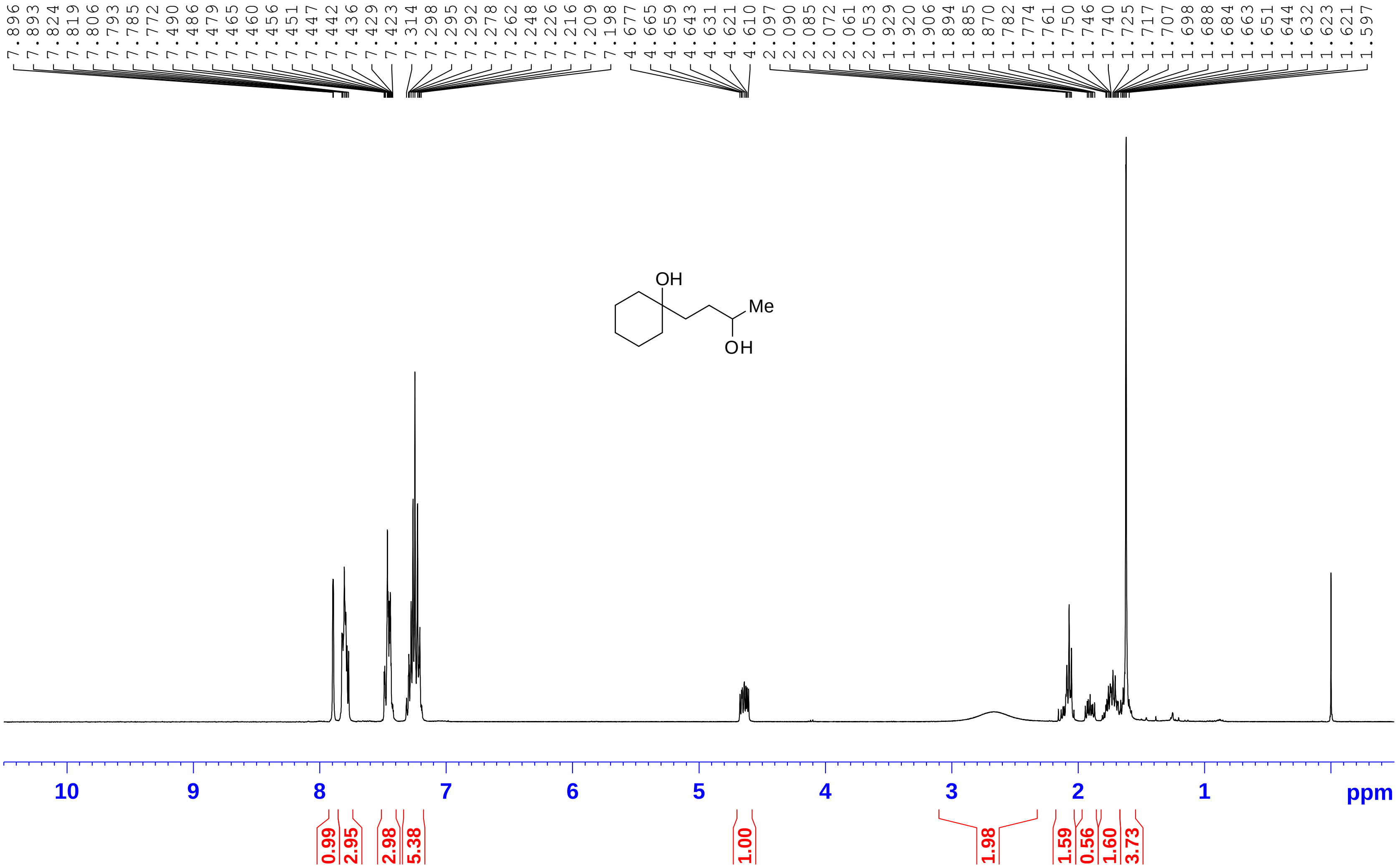

4. ¹H and ¹³C NMR spectrum of new compounds

^{13}C NMR spectrum of **3l** (100 MHz, $CDCl_3$)

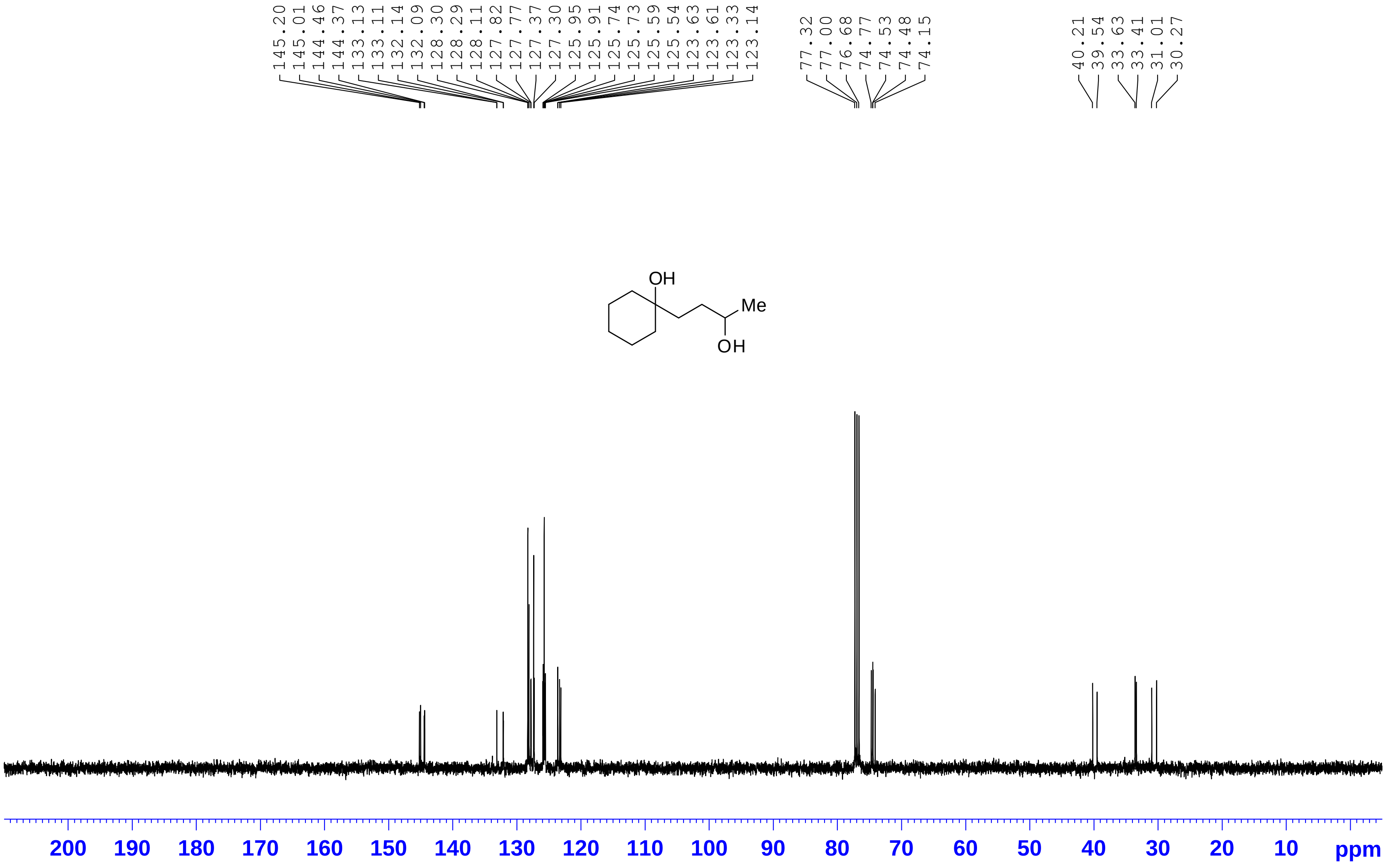

4. ^{1}H and ^{13}C NMR spectrum of new compounds

^{1}H NMR spectrum of **3m** (400 MHz, $CDCl_3$)

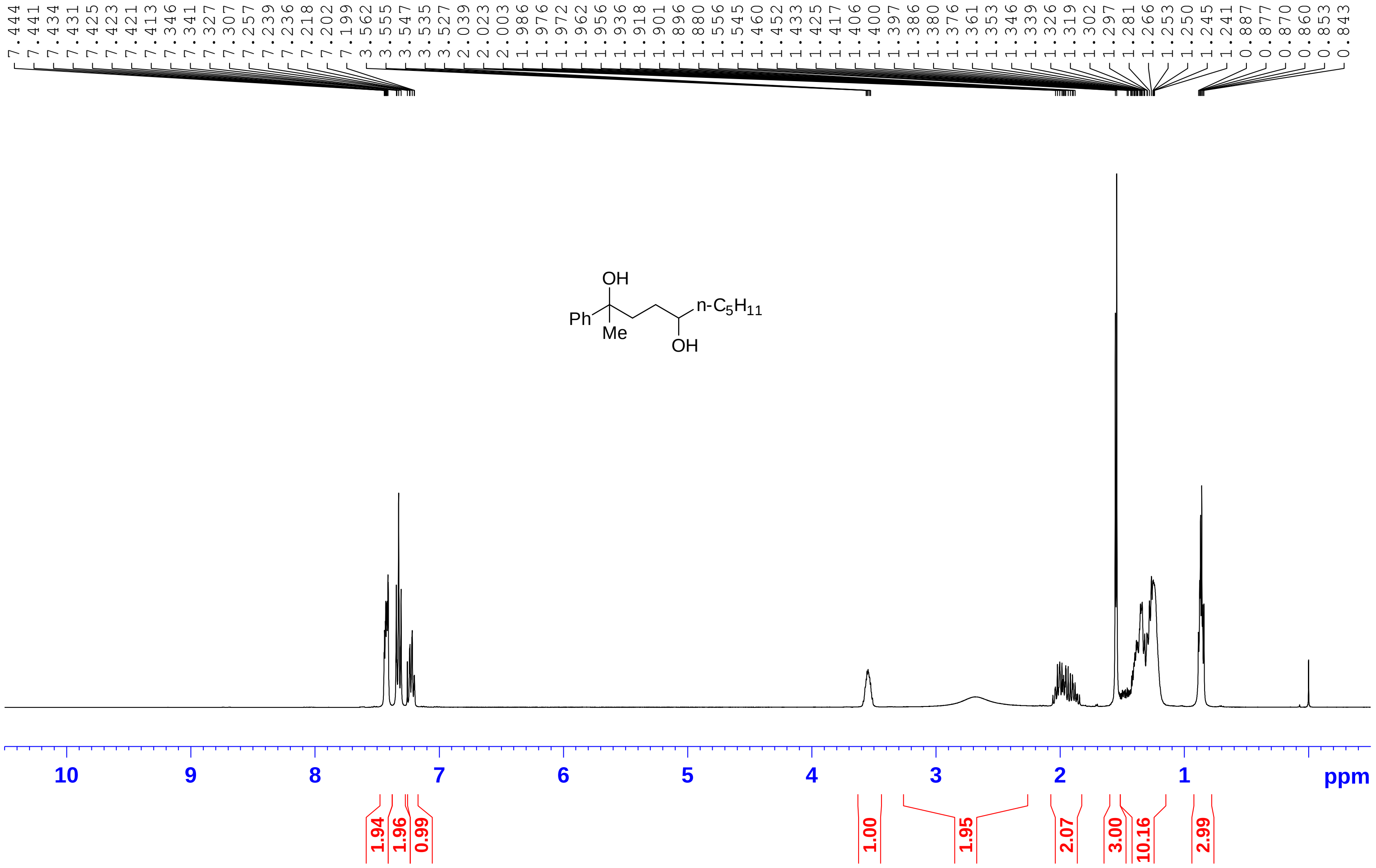

4. ¹H and ¹³C NMR spectrum of new compounds

^{13}C NMR spectrum of **3m** (100 MHz, $CDCl_3$)

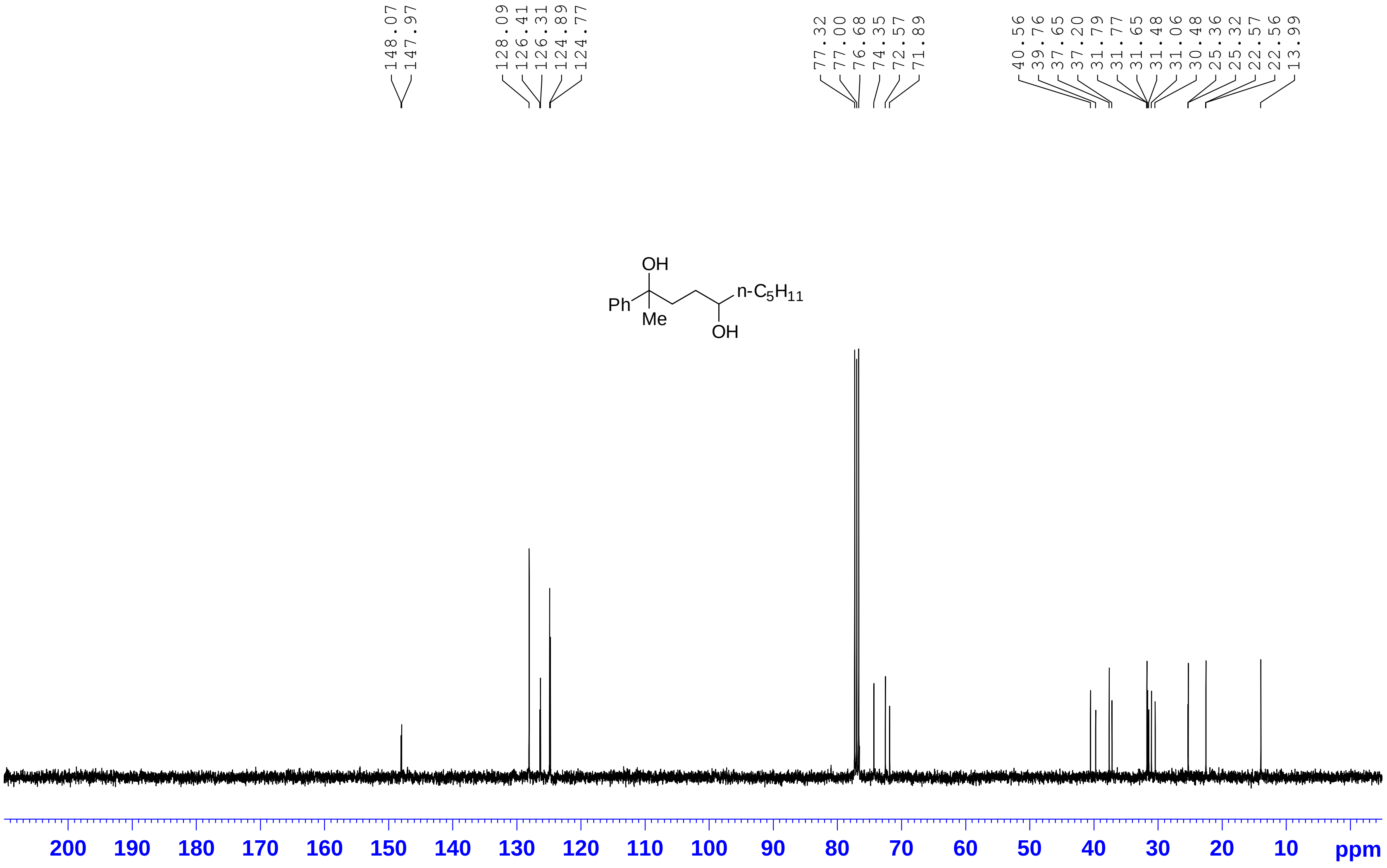

4. ^{1}H and ^{13}C NMR spectrum of new compounds

^{1}H NMR spectrum of **3n-major** (400 MHz, $CDCl_3$)

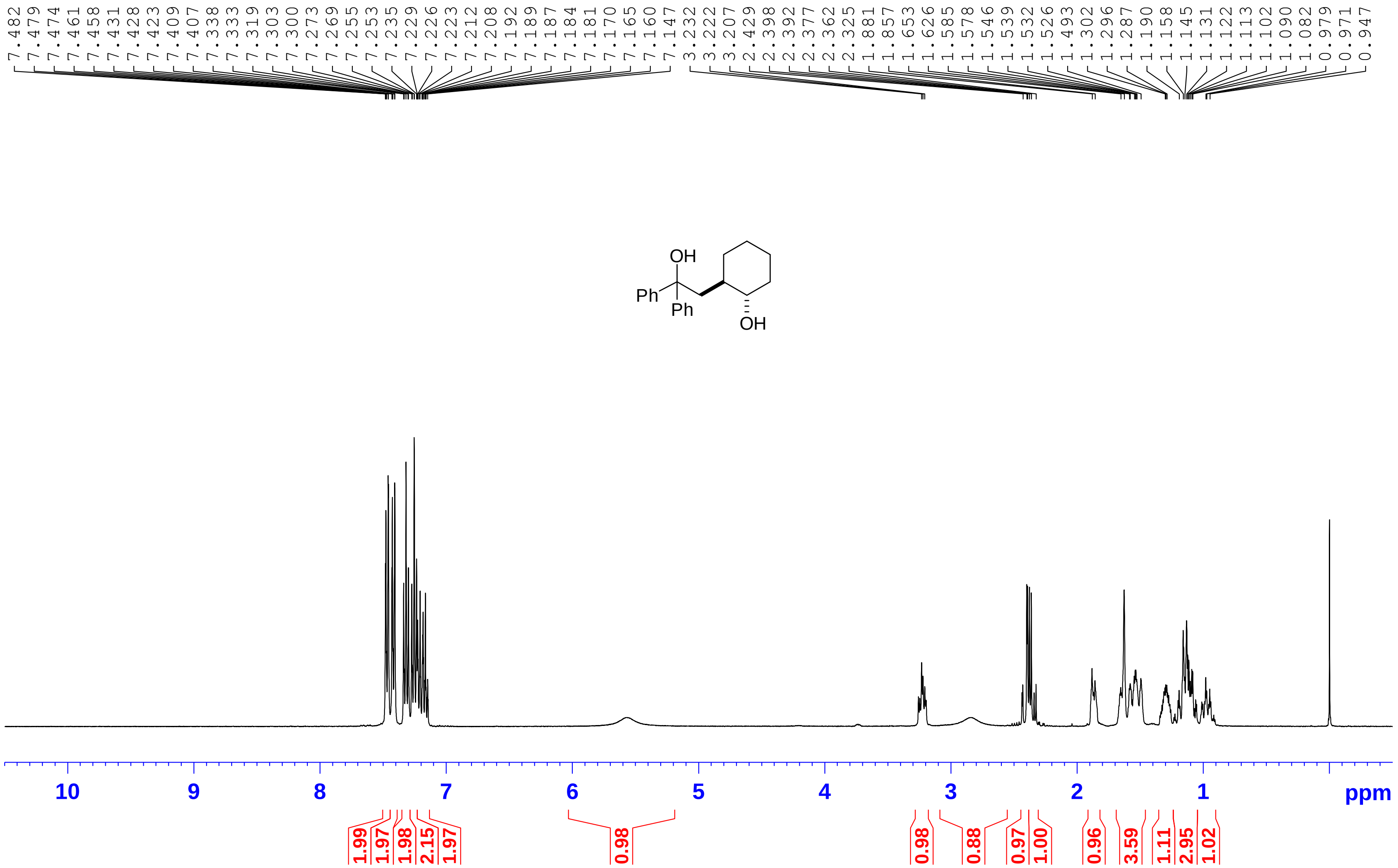

4. ^{1}H and ^{13}C NMR spectrum of new compounds

^{13}C NMR spectrum of **3n-major** (100 MHz, $CDCl_3$)

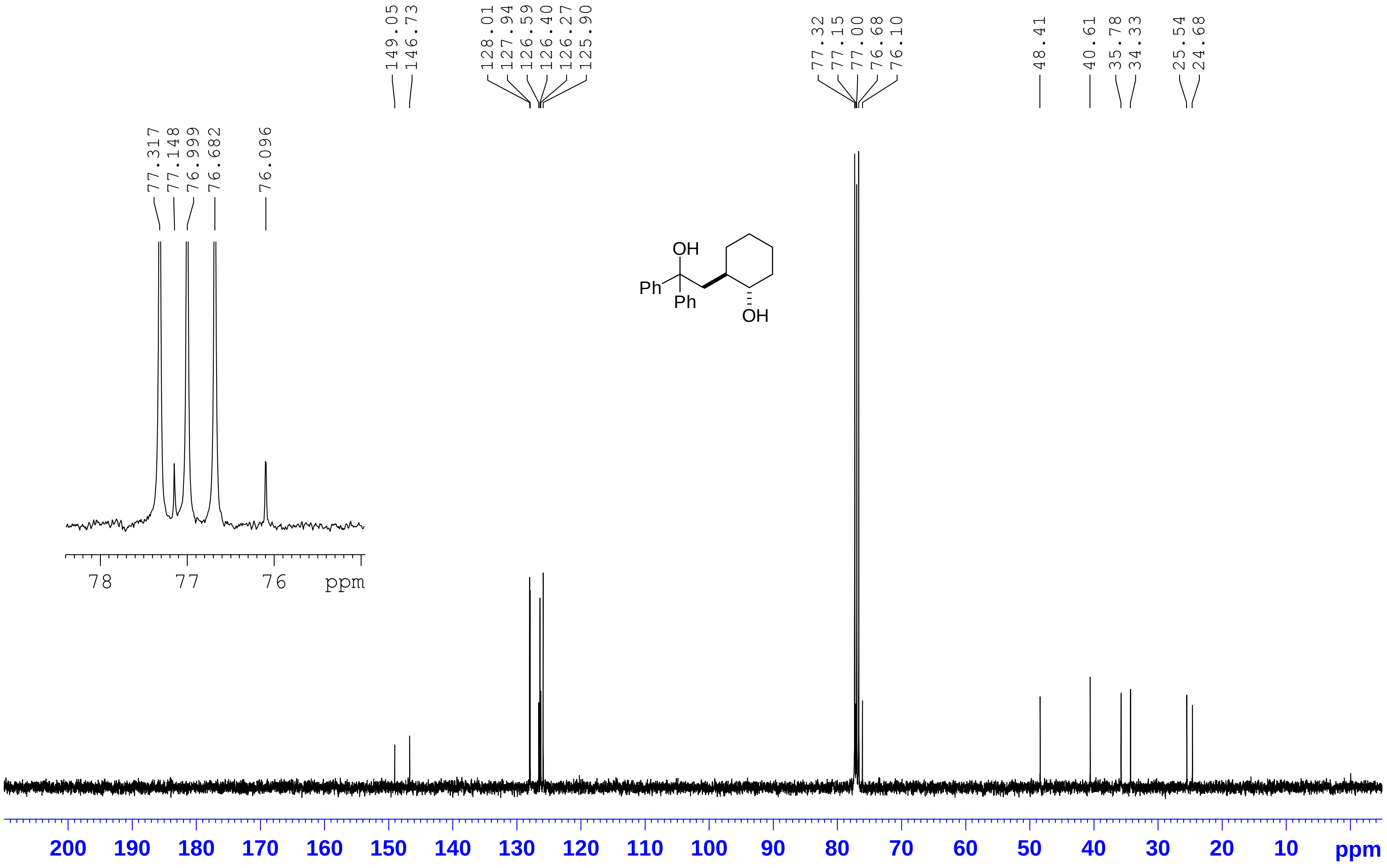

4. ^{1}H and ^{13}C NMR spectrum of new compounds

^{1}H NMR spectrum of **3p** (400 MHz, $CDCl_3$)

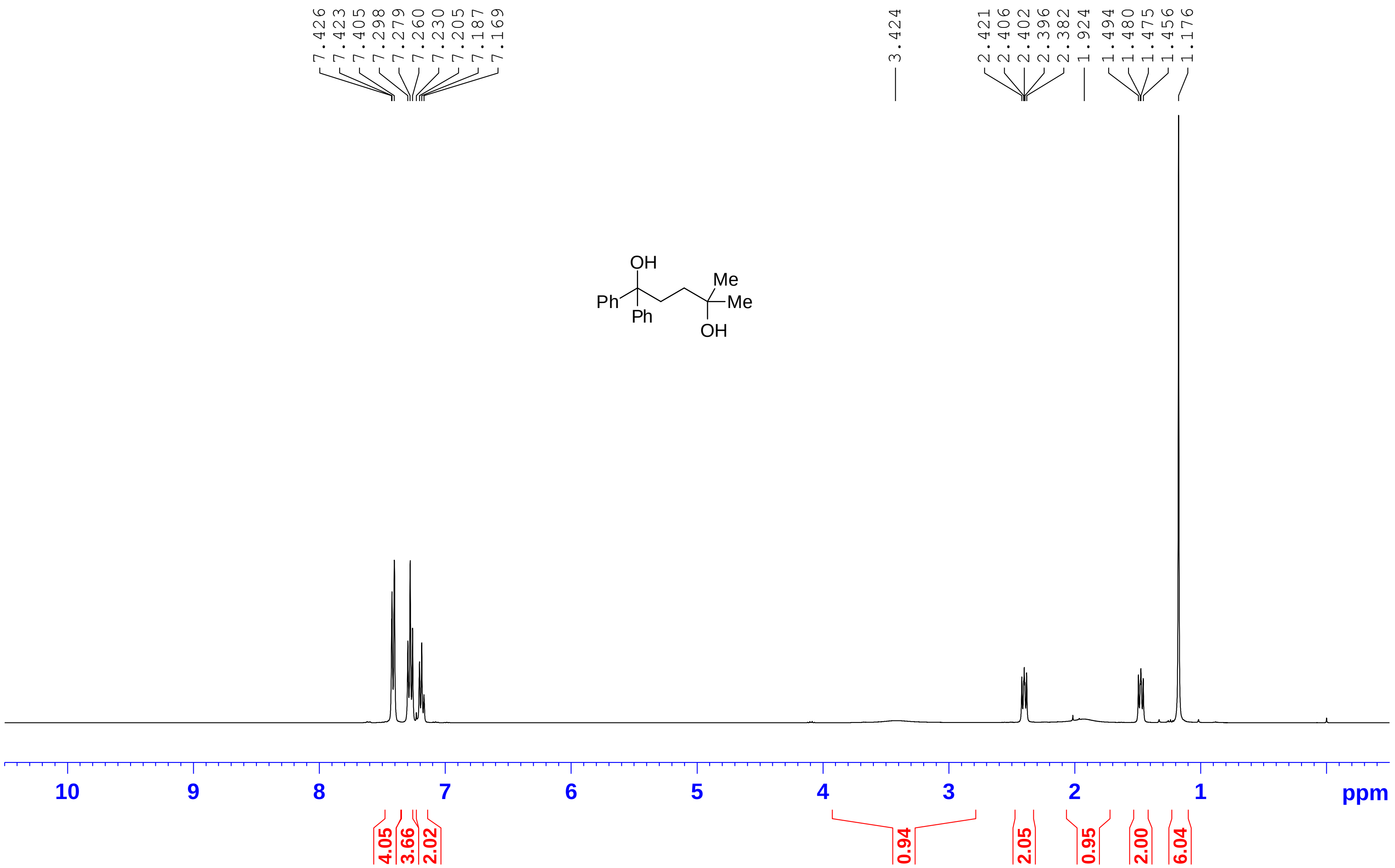

4. ^{1}H and ^{13}C NMR spectrum of new compounds

^{13}C NMR spectrum of **3p** (100 MHz, $CDCl_3$)

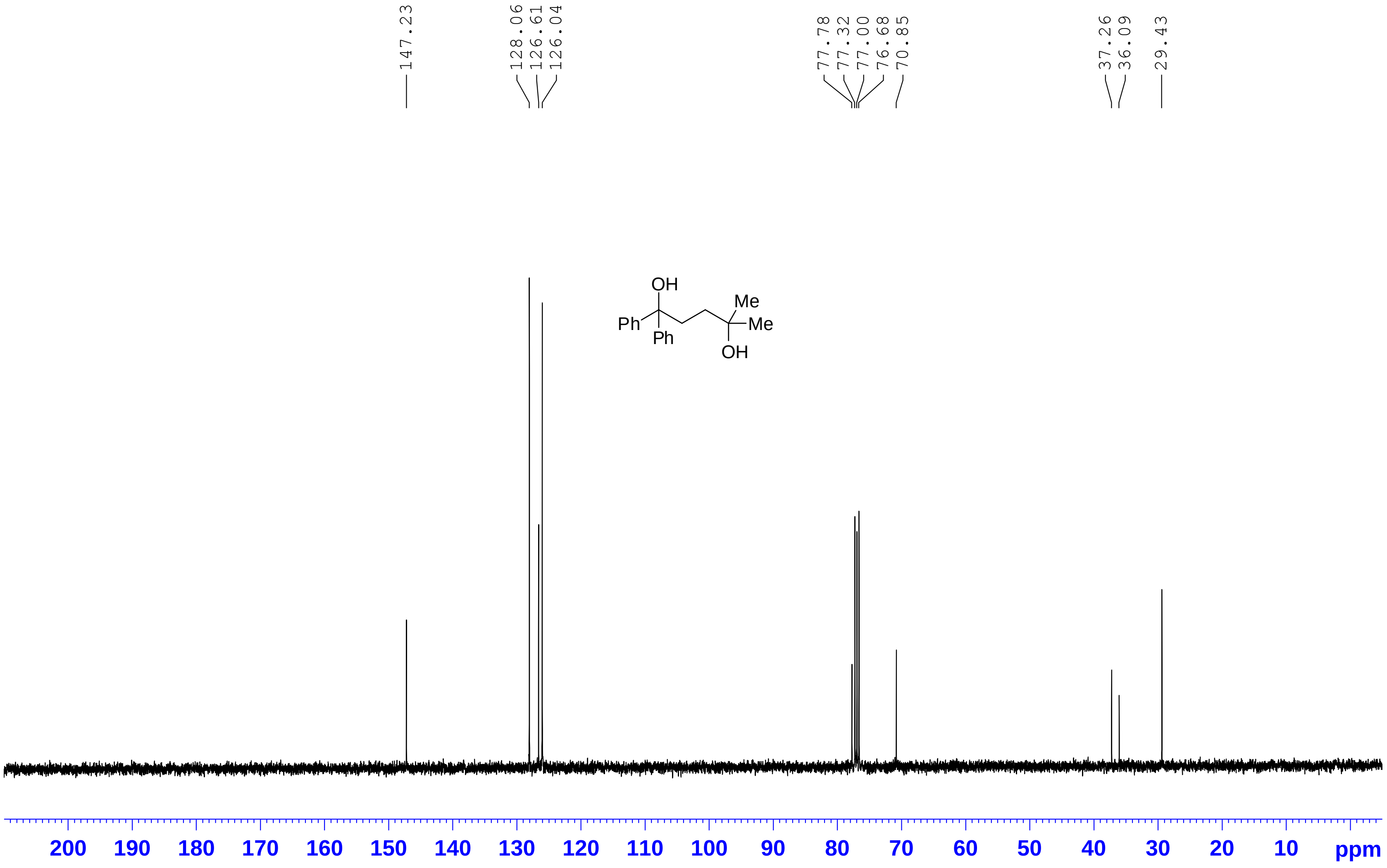

4. ^{1}H and ^{13}C NMR spectrum of new compounds

^{1}H NMR spectrum of **3q** (400 MHz, $CDCl_3$)

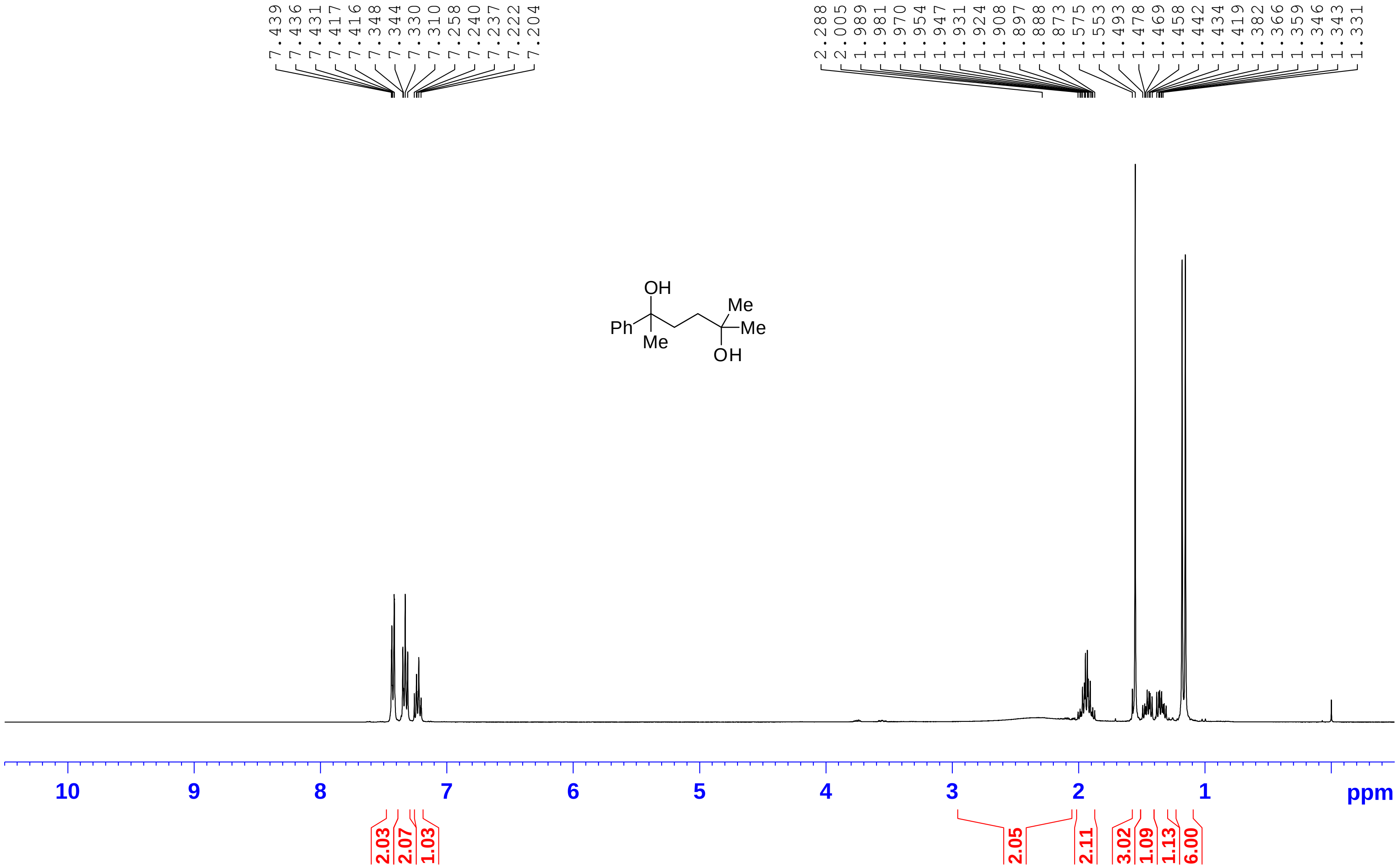

4. ^{1}H and ^{13}C NMR spectrum of new compounds

^{13}C NMR spectrum of **3q** (100 MHz, $CDCl_3$)

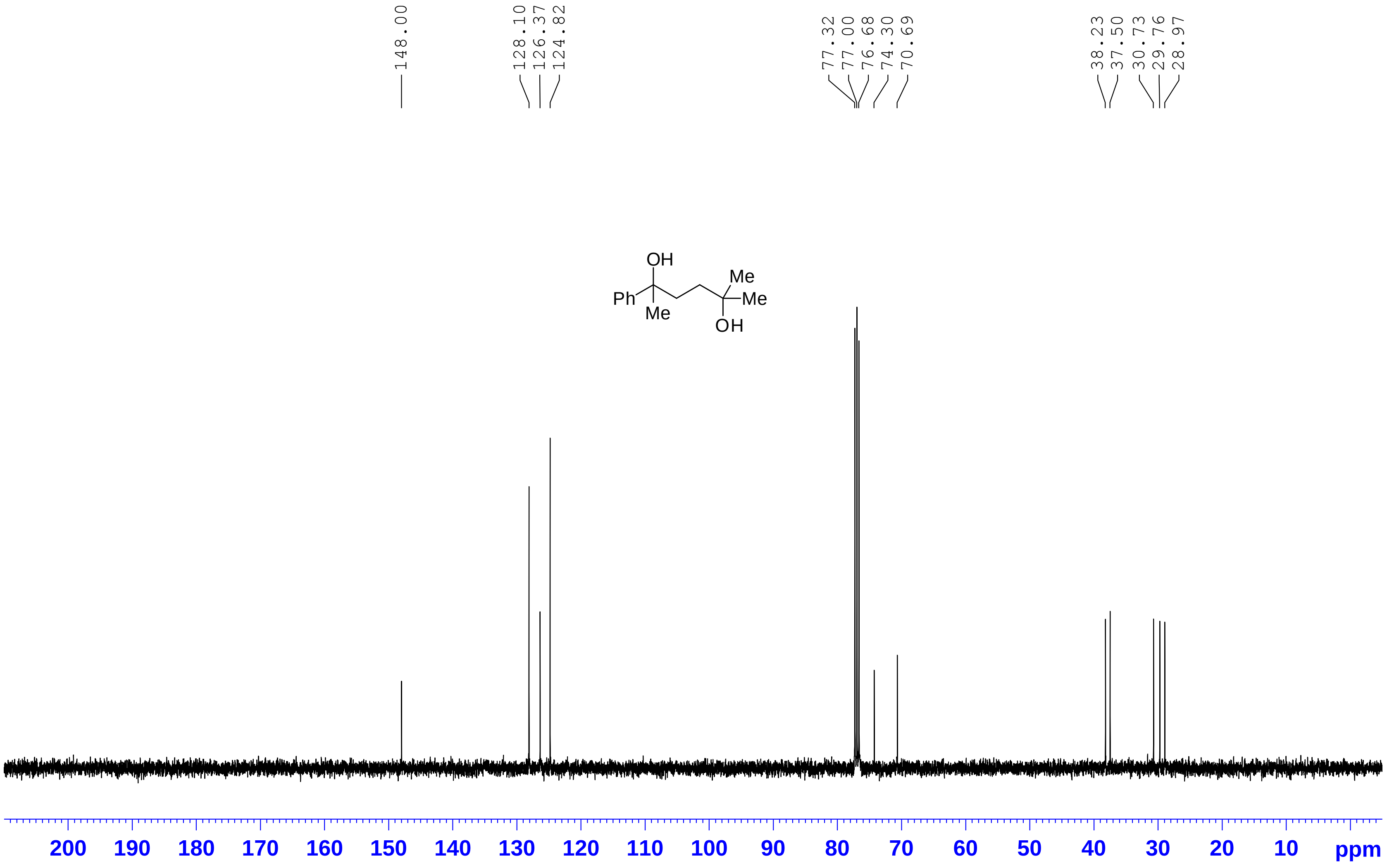

4. ^{1}H and ^{13}C NMR spectrum of new compounds

^{1}H NMR spectrum of **3r** (400 MHz, $CDCl_3$)

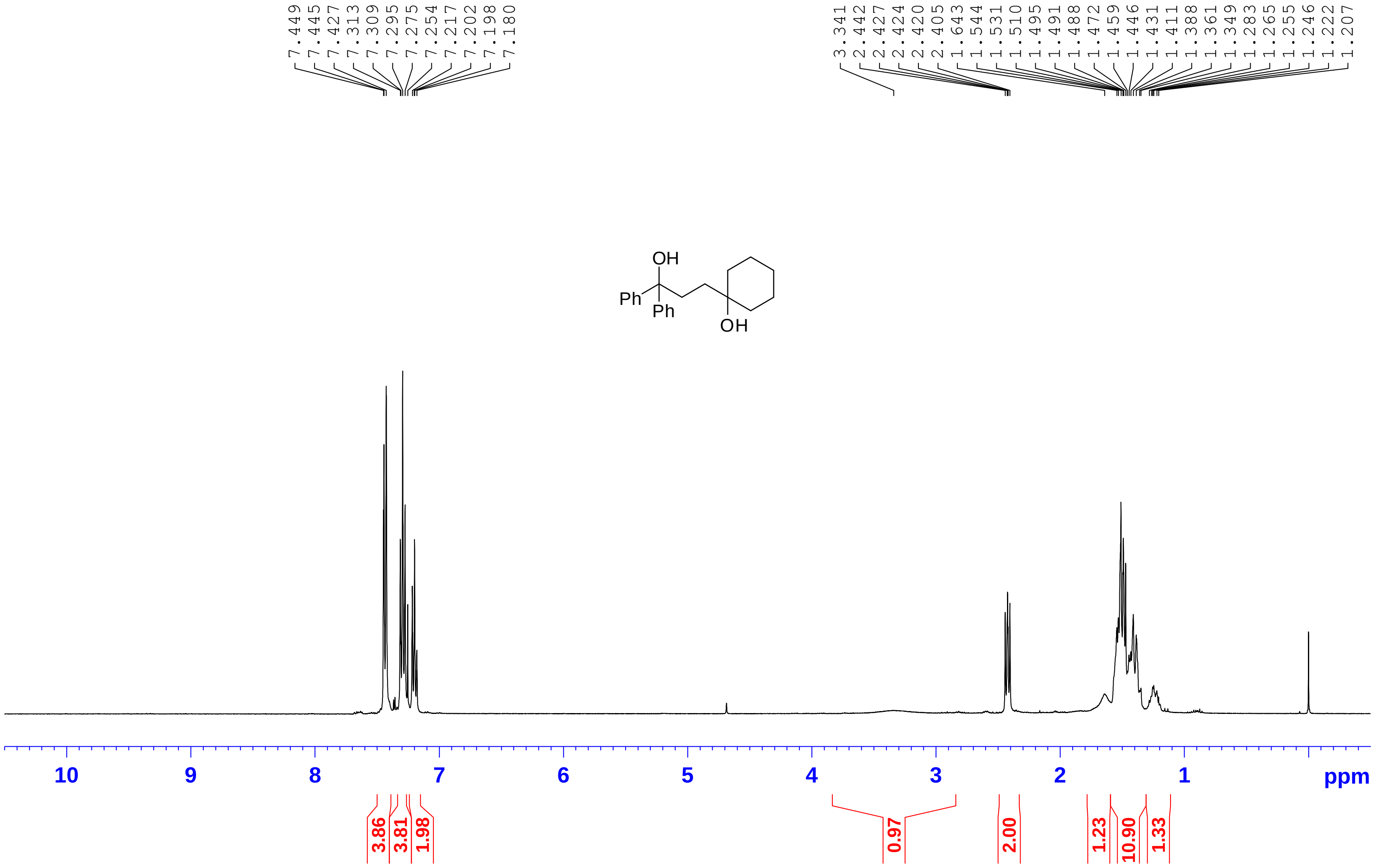

4. ^{1}H and ^{13}C NMR spectrum of new compounds

^{13}C NMR spectrum of **3r** (100 MHz, $CDCl_3$)

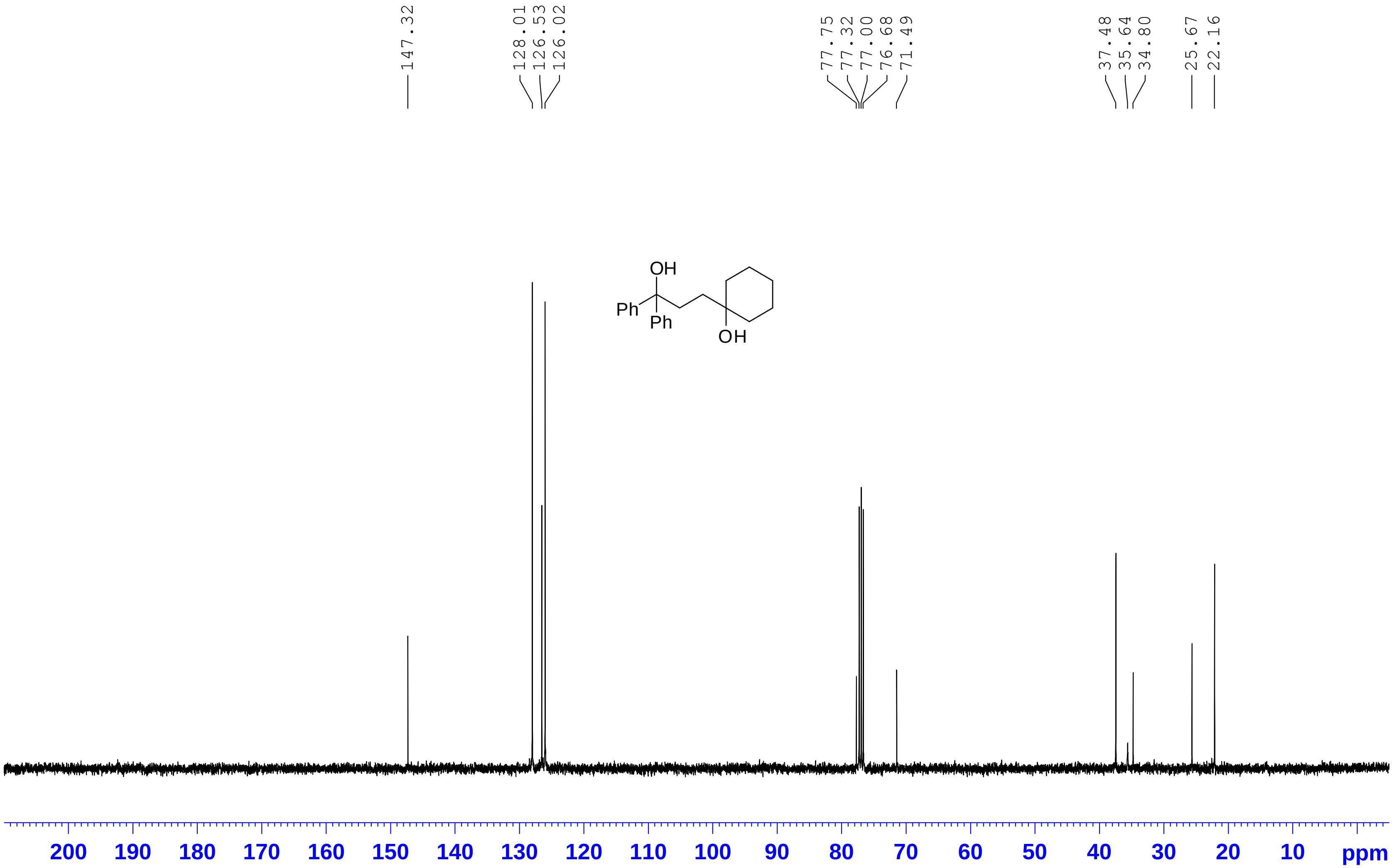

4. ^{1}H and ^{13}C NMR spectrum of new compounds

^{1}H NMR spectrum of **3s** (400 MHz, $CDCl_3$)

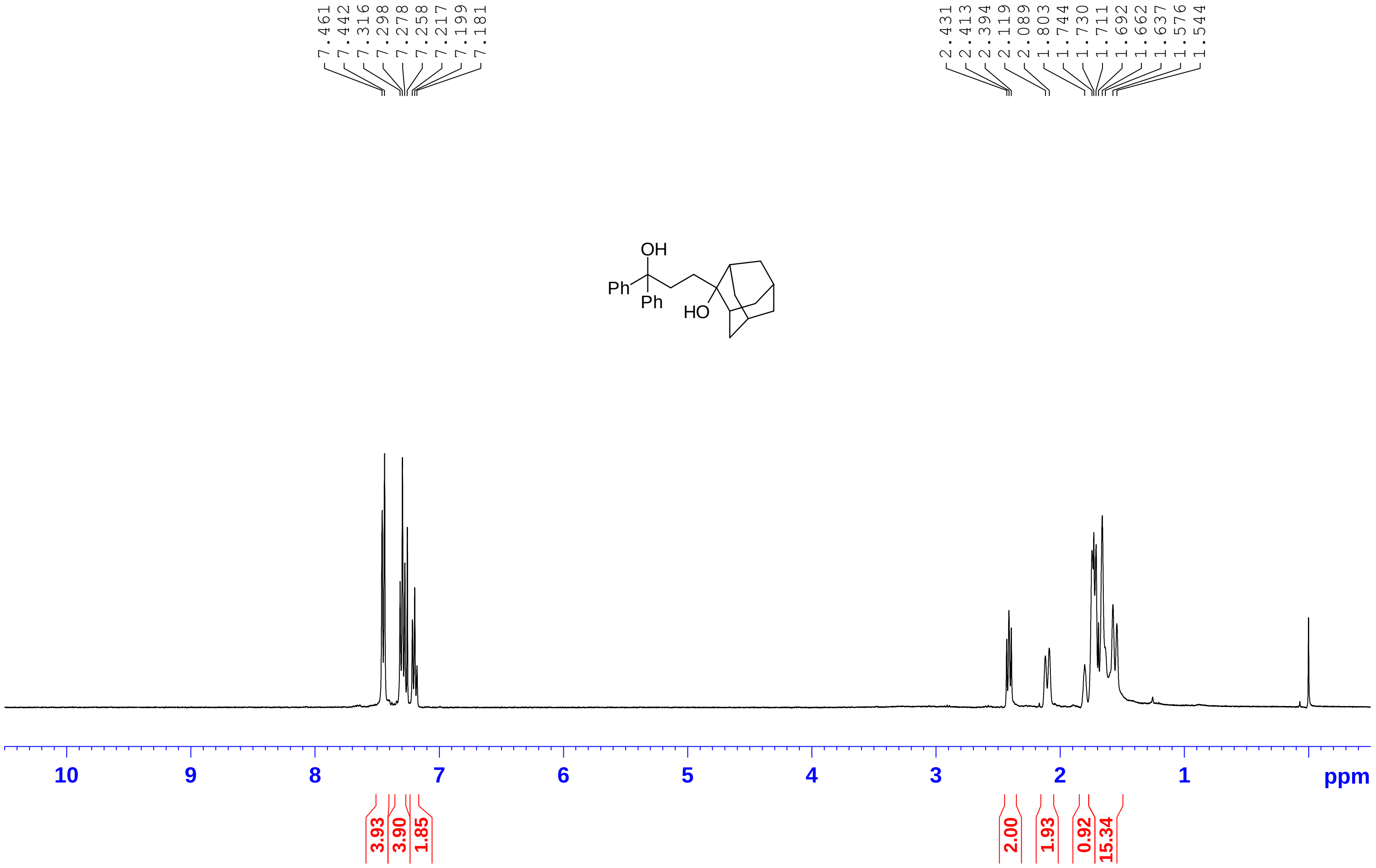

4. ^{1}H and ^{13}C NMR spectrum of new compounds

^{13}C NMR spectrum of **3s** (100 MHz, $CDCl_3$)

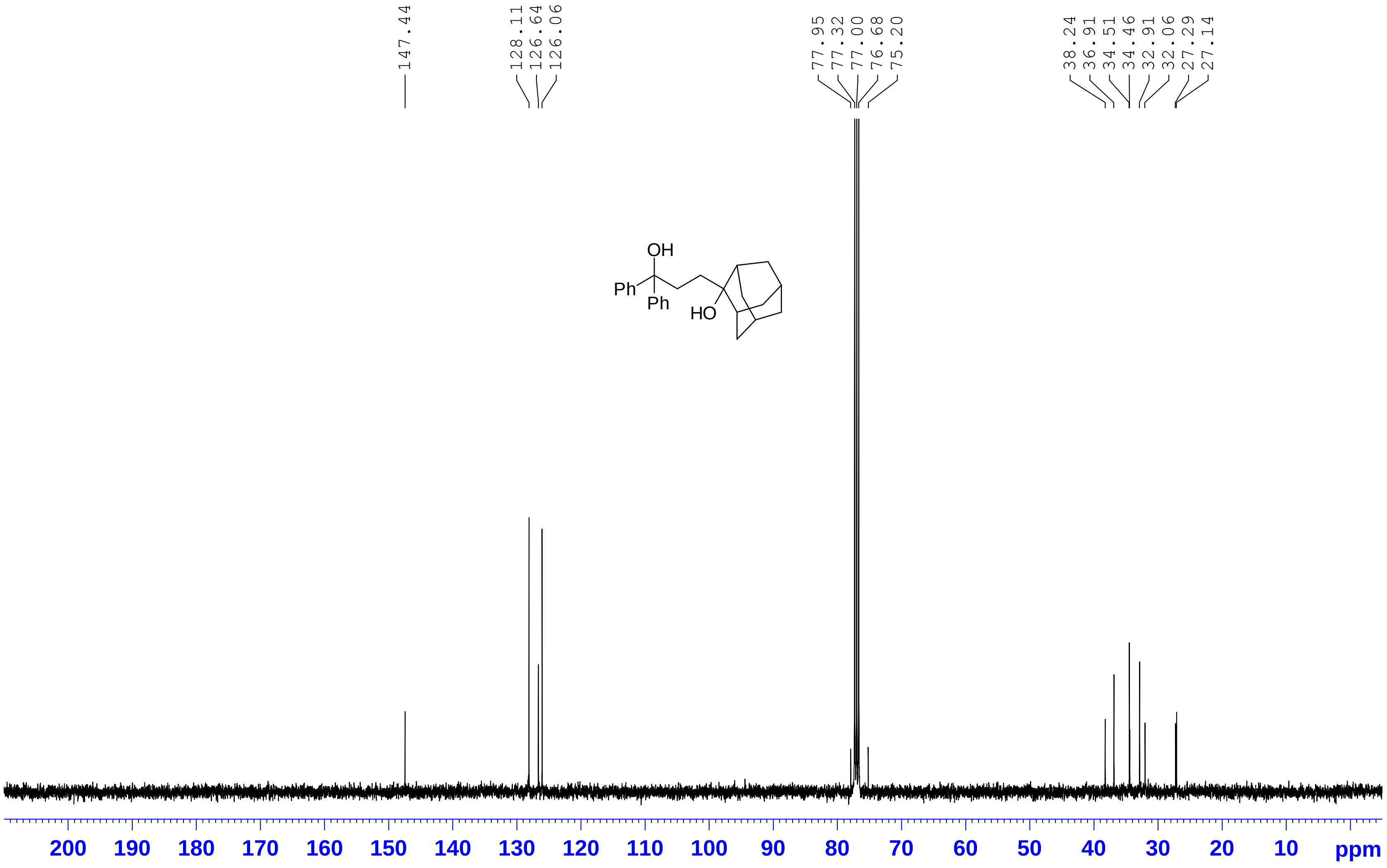

4. ^{1}H and ^{13}C NMR spectrum of new compounds

^{1}H NMR spectrum of **5c** (400 MHz, $CDCl_3$)

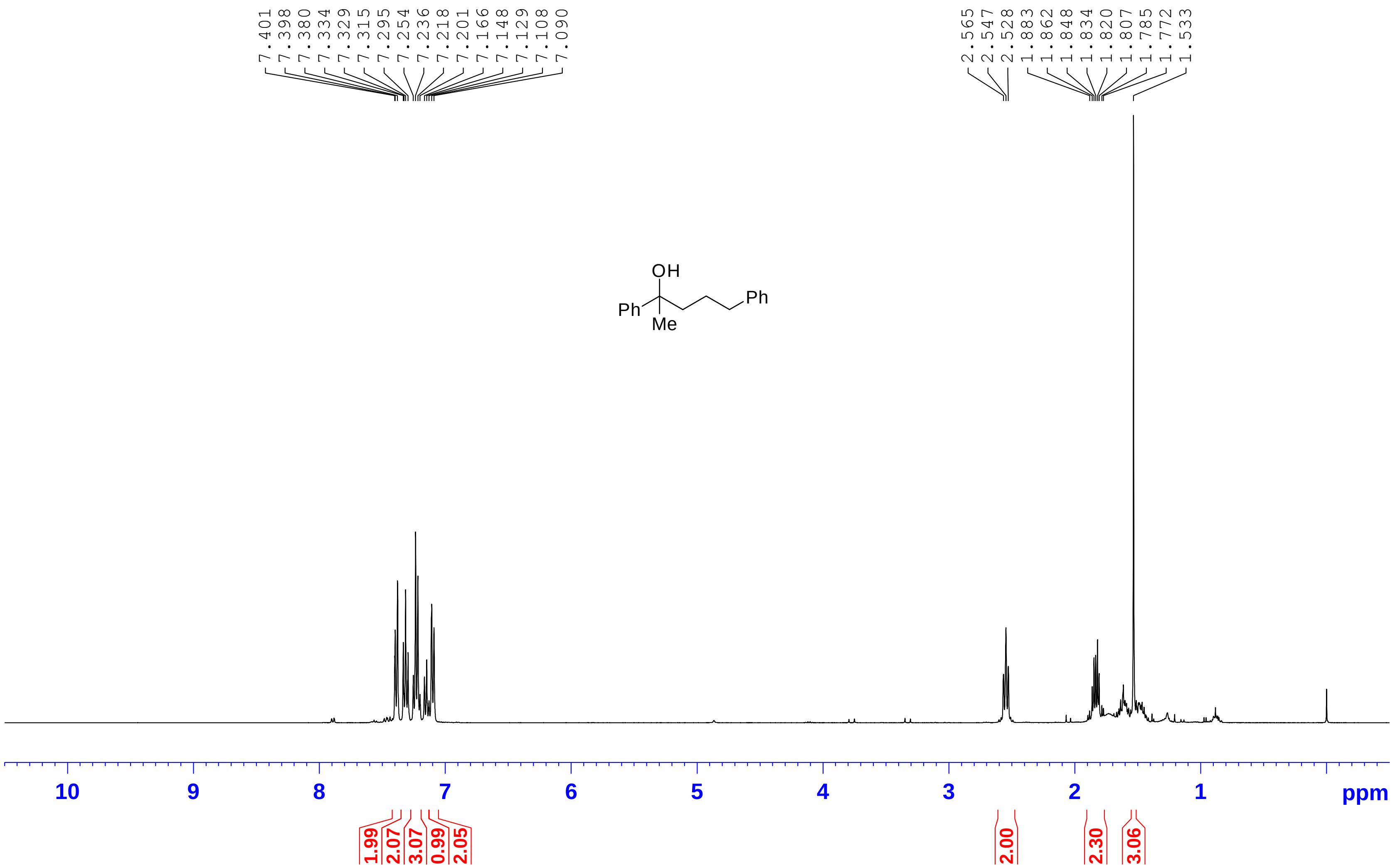

4. ^{1}H and ^{13}C NMR spectrum of new compounds

^{13}C NMR spectrum of **5c** (100 MHz, $CDCl_3$)

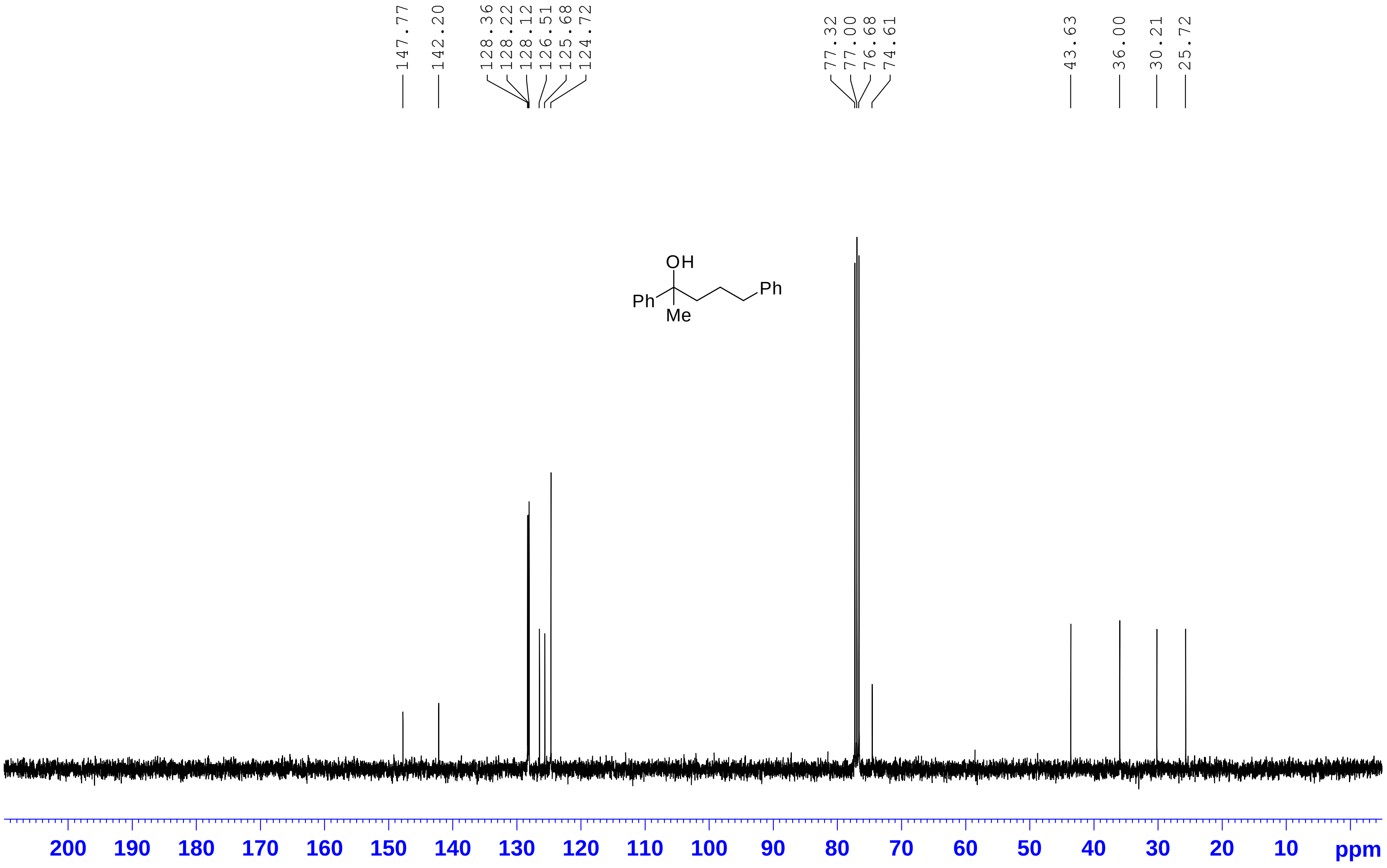

4. ^{1}H and ^{13}C NMR spectrum of new compounds

^{1}H NMR spectrum of **5d** (400 MHz, $CDCl_3$)

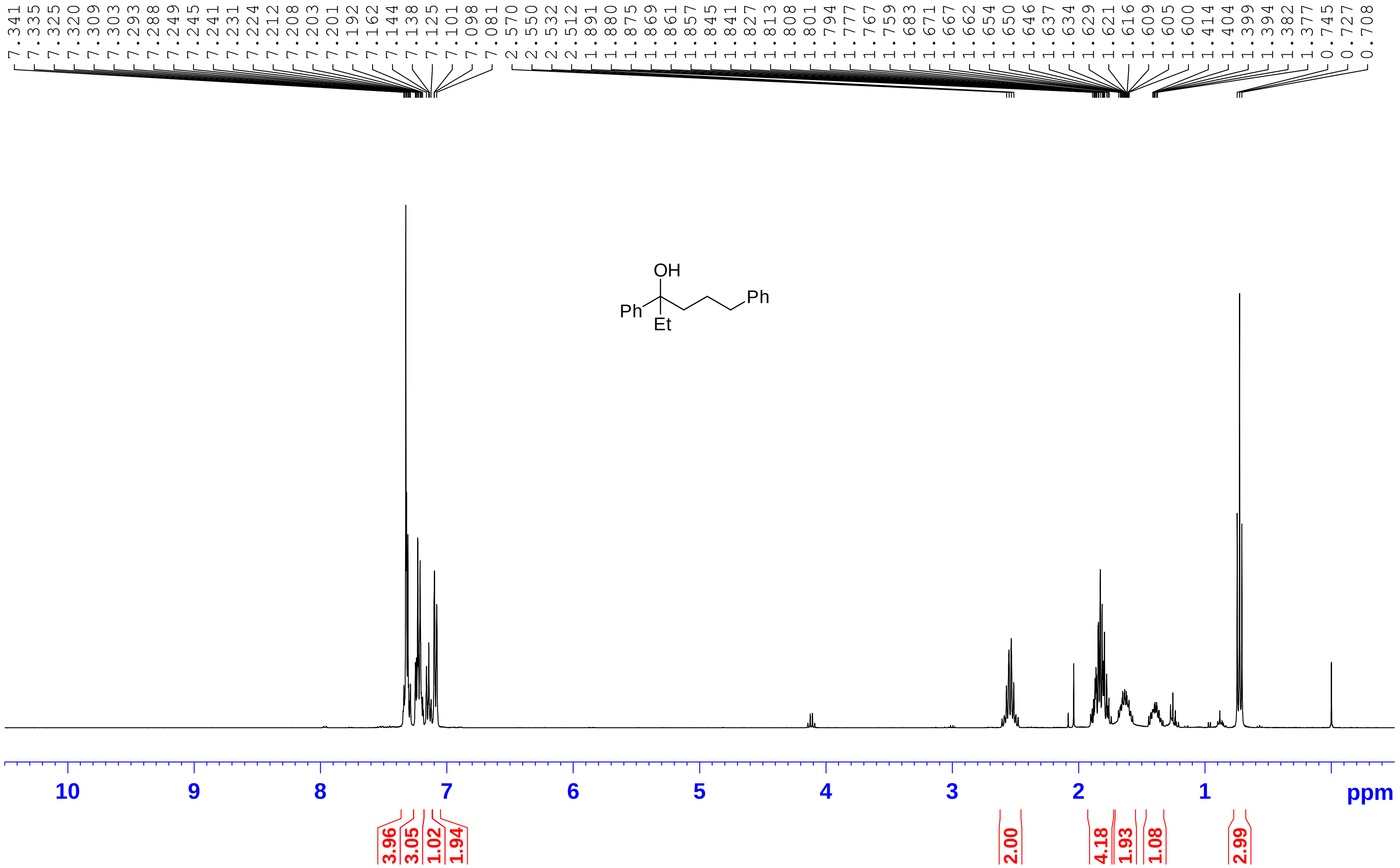

4. ^{1}H and ^{13}C NMR spectrum of new compounds

^{13}C NMR spectrum of **5d** (100 MHz, $CDCl_3$)

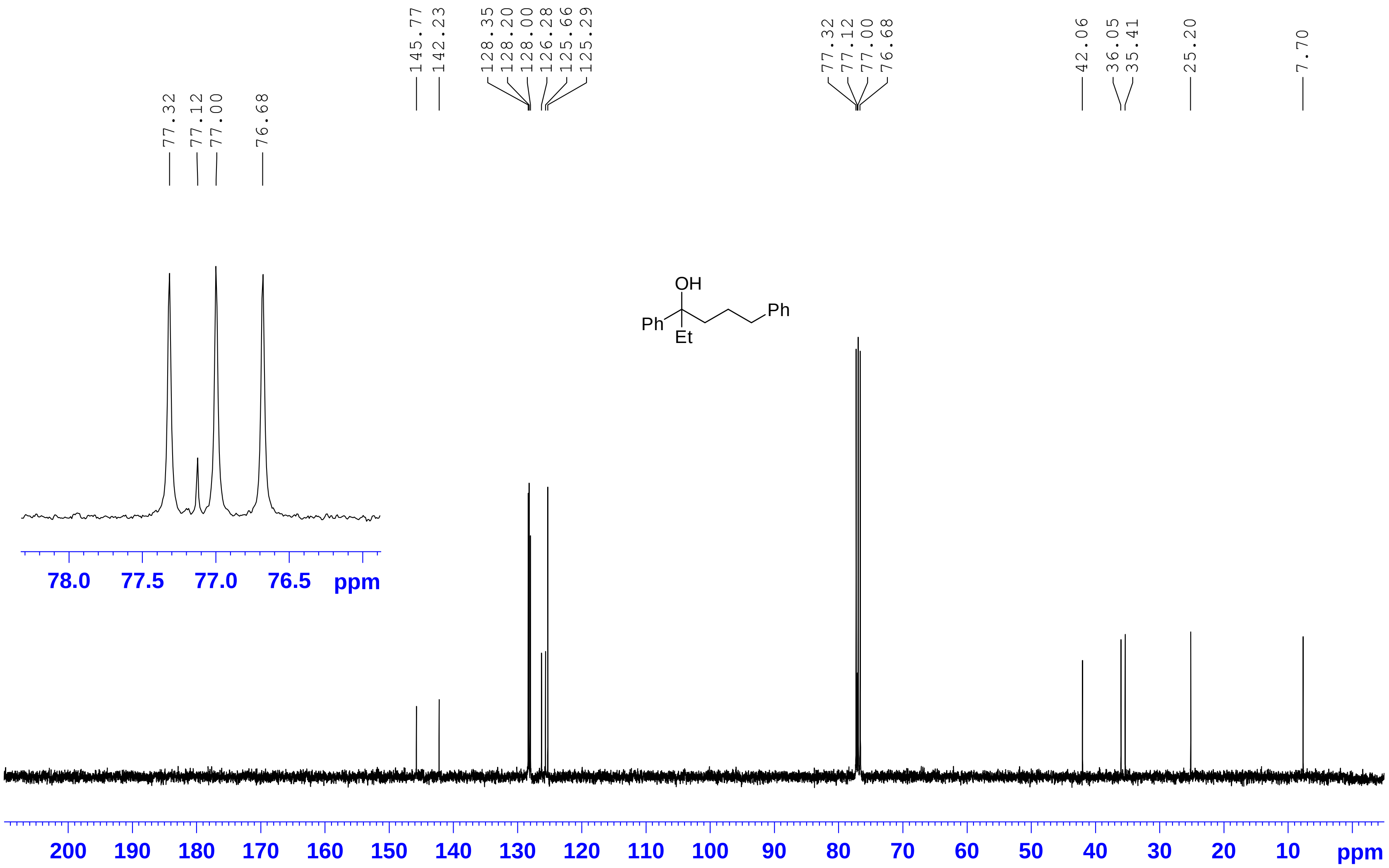

4. ^{1}H and ^{13}C NMR spectrum of new compounds

^{1}H NMR spectrum of **5e** (400 MHz, $CDCl_3$)

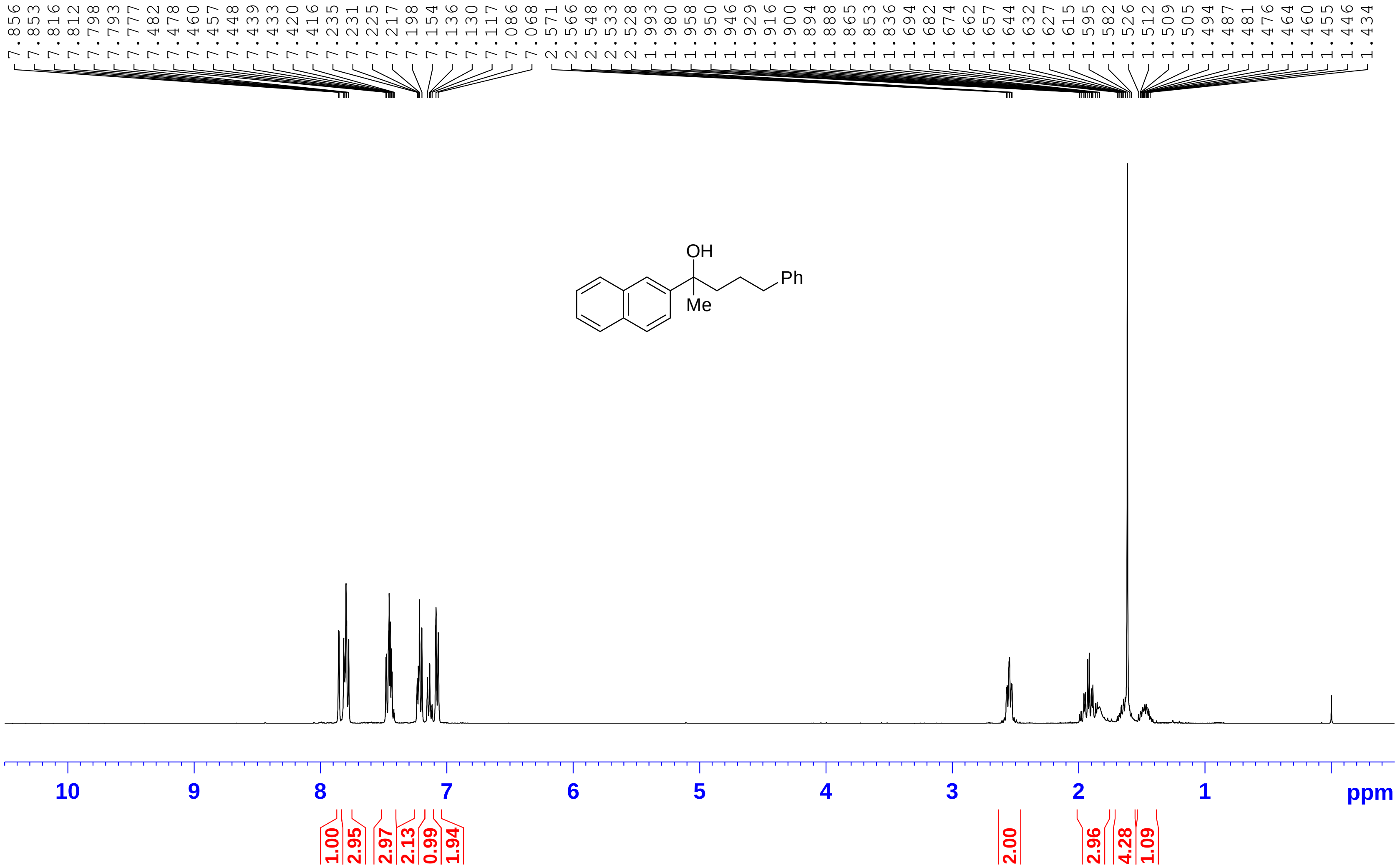

4. ^{1}H and ^{13}C NMR spectrum of new compounds

^{13}C NMR spectrum of **5e** (100 MHz, $CDCl_3$)

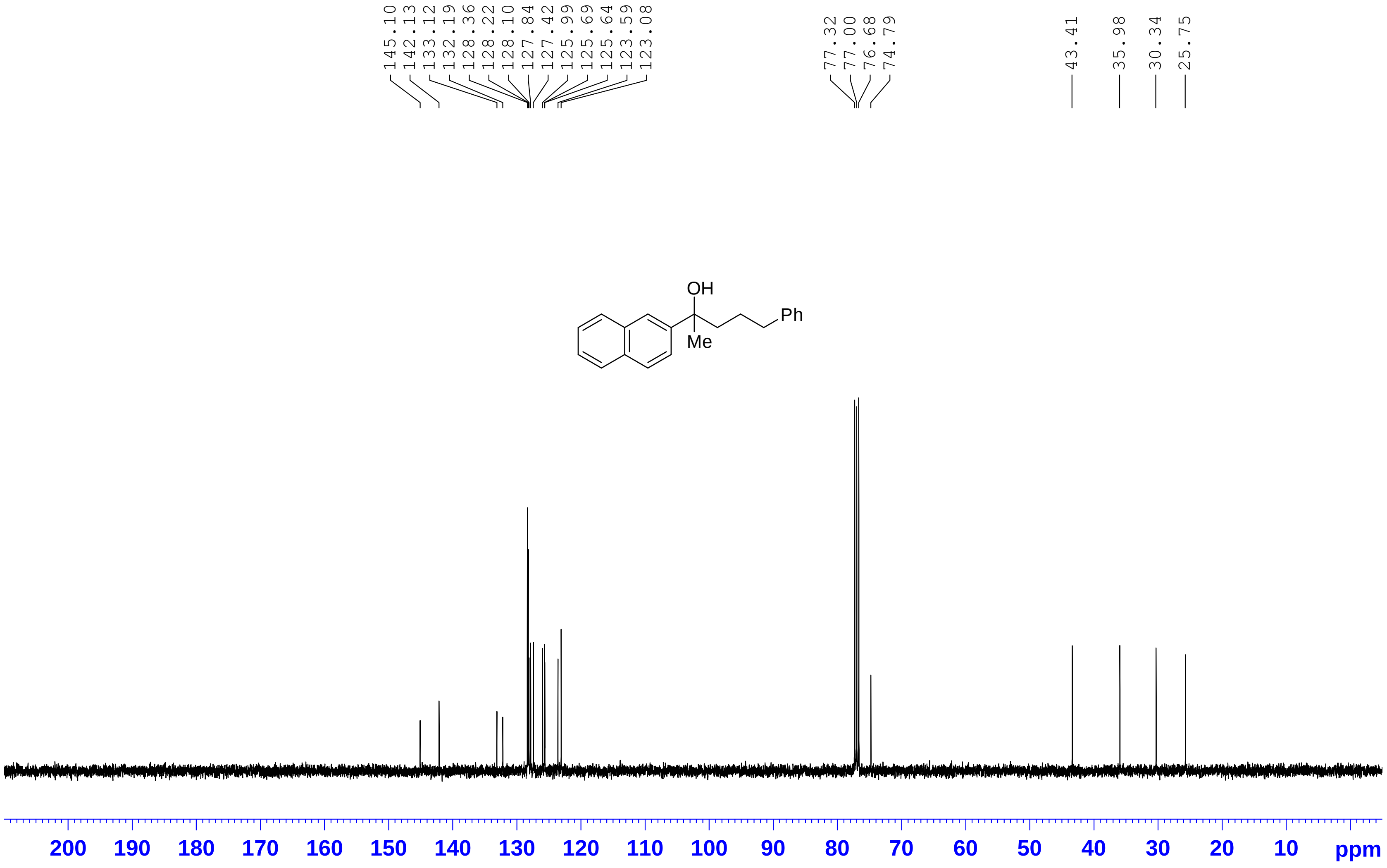

4. ^{1}H and ^{13}C NMR spectrum of new compounds

^{1}H NMR spectrum of **5f** (400 MHz, $CDCl_3$)

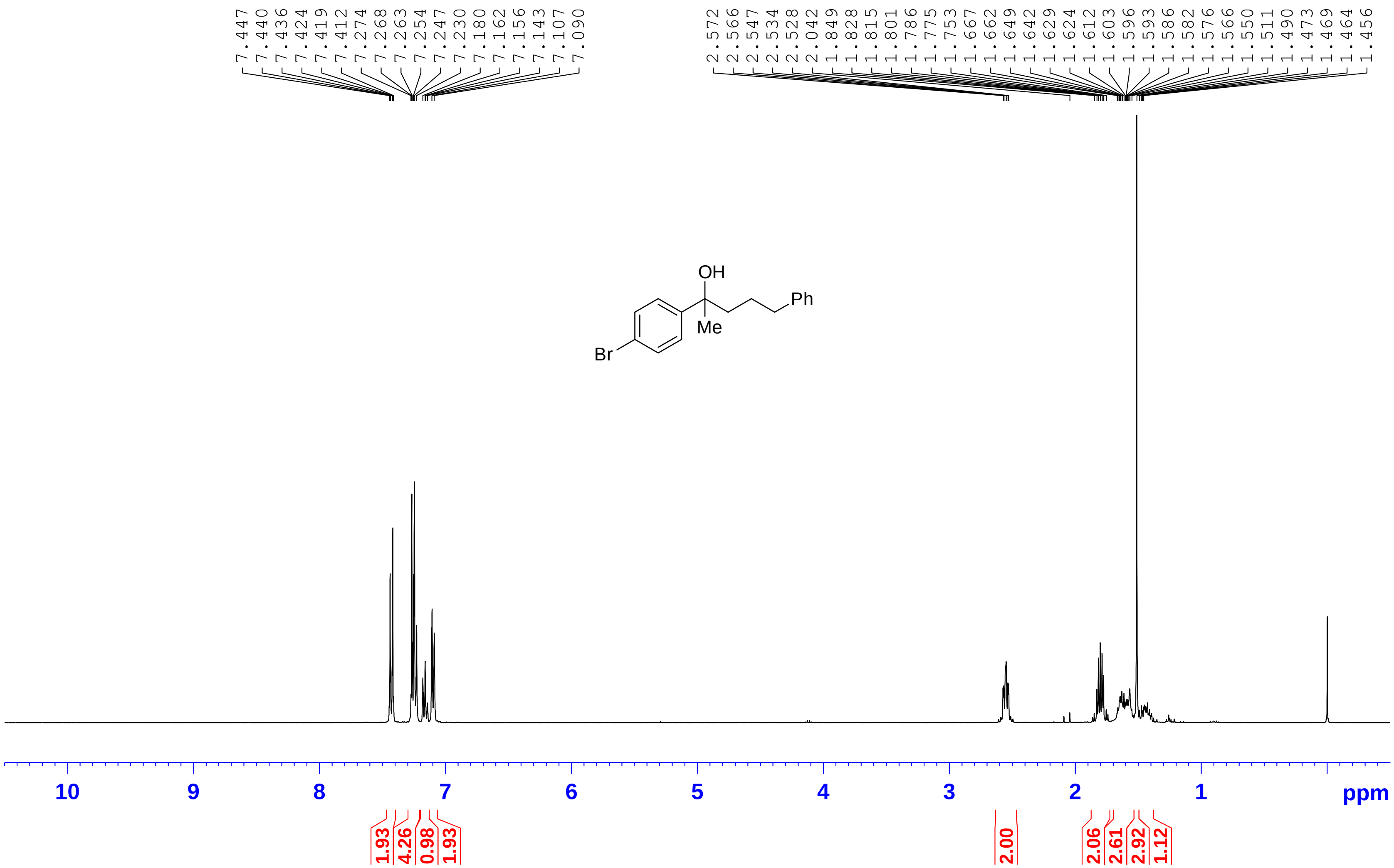

4. ^{1}H and ^{13}C NMR spectrum of new compounds

^{13}C NMR spectrum of **5f** (100 MHz, $CDCl_3$)

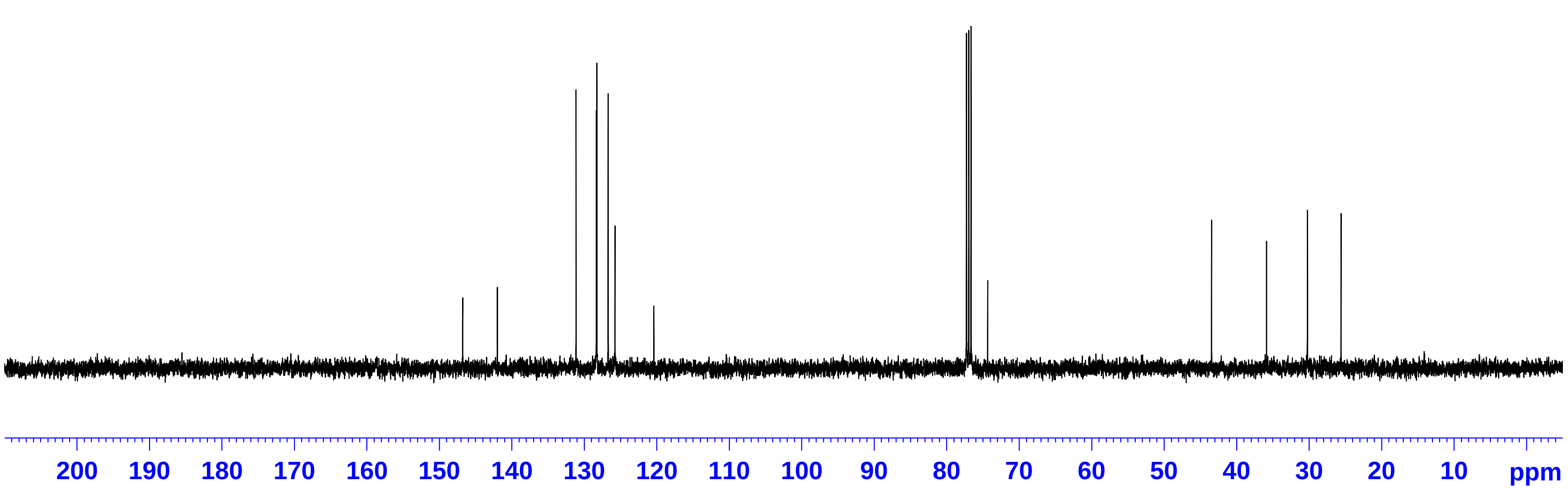

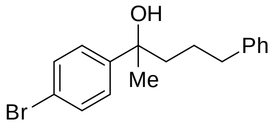

4. ¹H and ¹³C NMR spectrum of new compounds

¹H NMR spectrum of **5g** (400 MHz, $CDCl_3$)

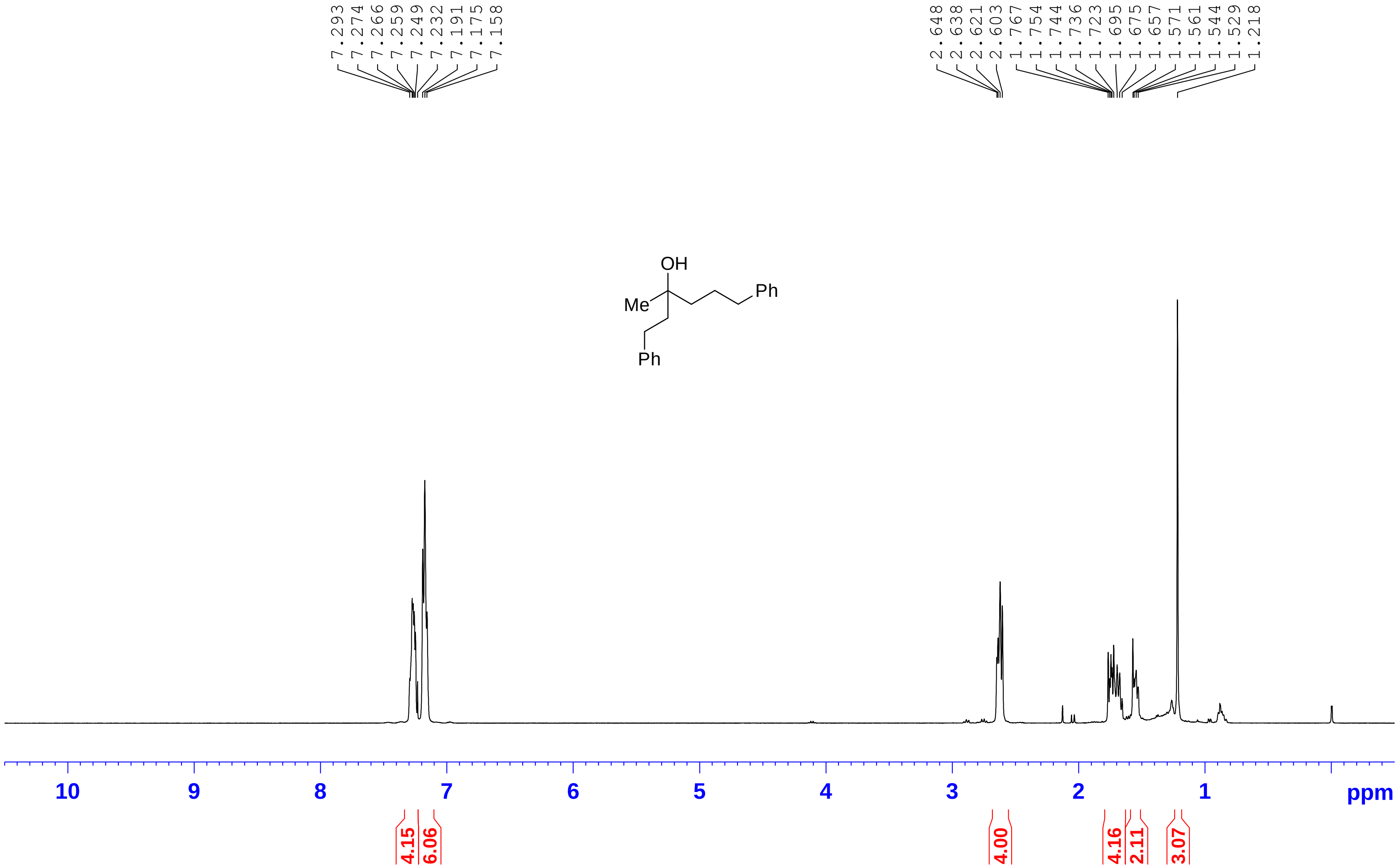

4. ^{1}H and ^{13}C NMR spectrum of new compounds

^{13}C NMR spectrum of **5g** (100 MHz, $CDCl_3$)

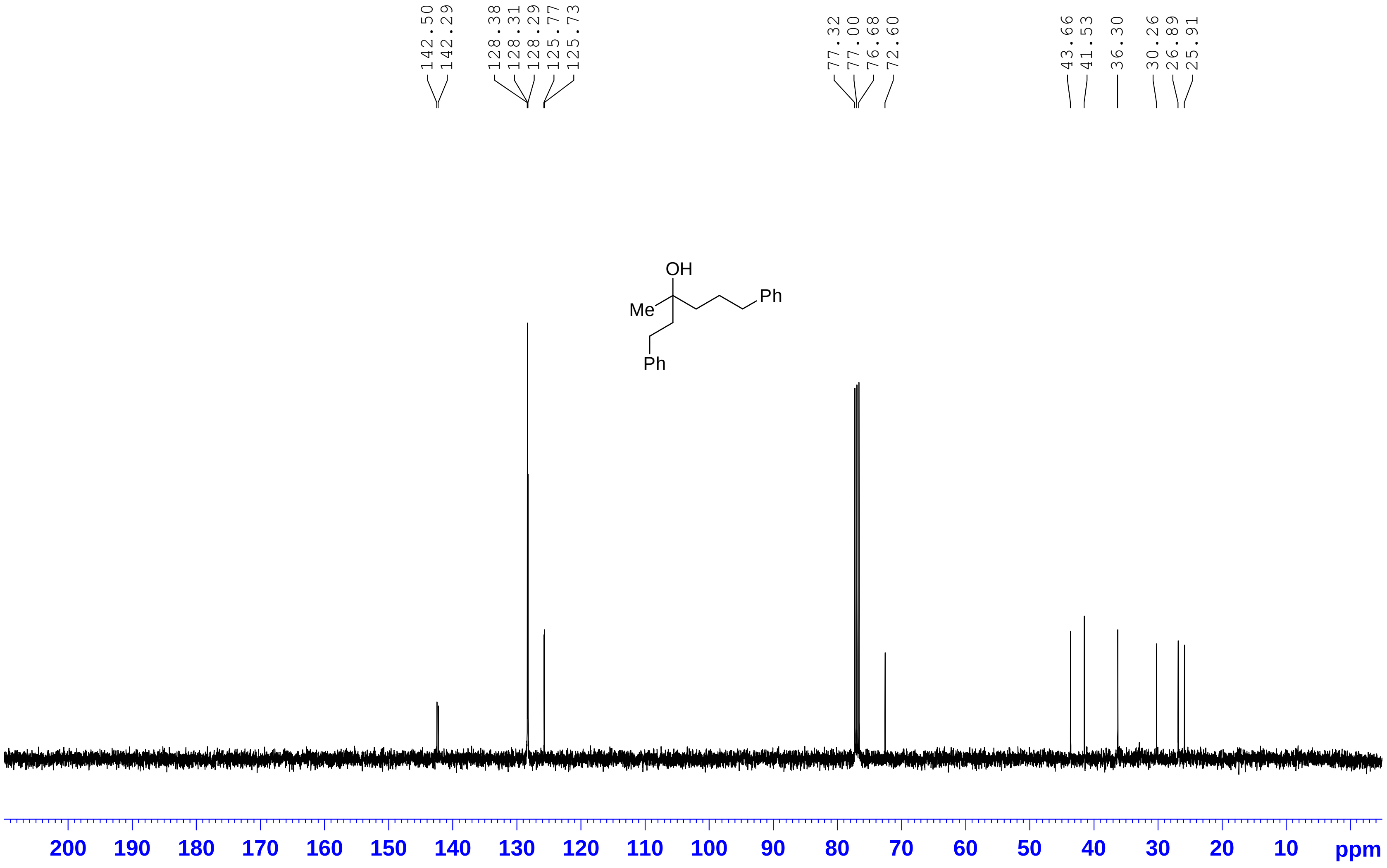

4. ^{1}H and ^{13}C NMR spectrum of new compounds

^{1}H NMR spectrum of **5h** (400 MHz, $CDCl_3$)

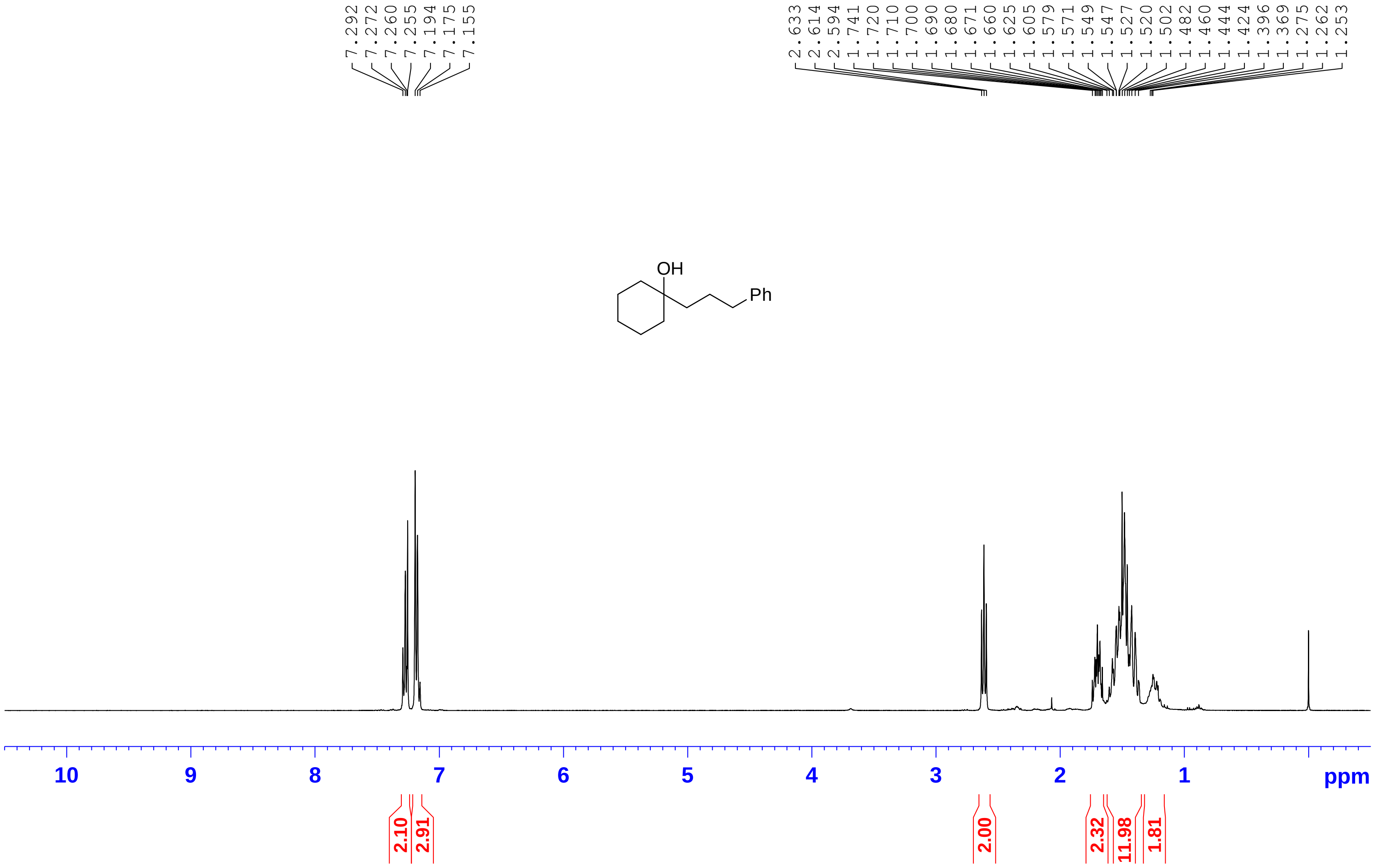

4. ^{1}H and ^{13}C NMR spectrum of new compounds

^{13}C NMR spectrum of **5h** (100 MHz, $CDCl_3$)

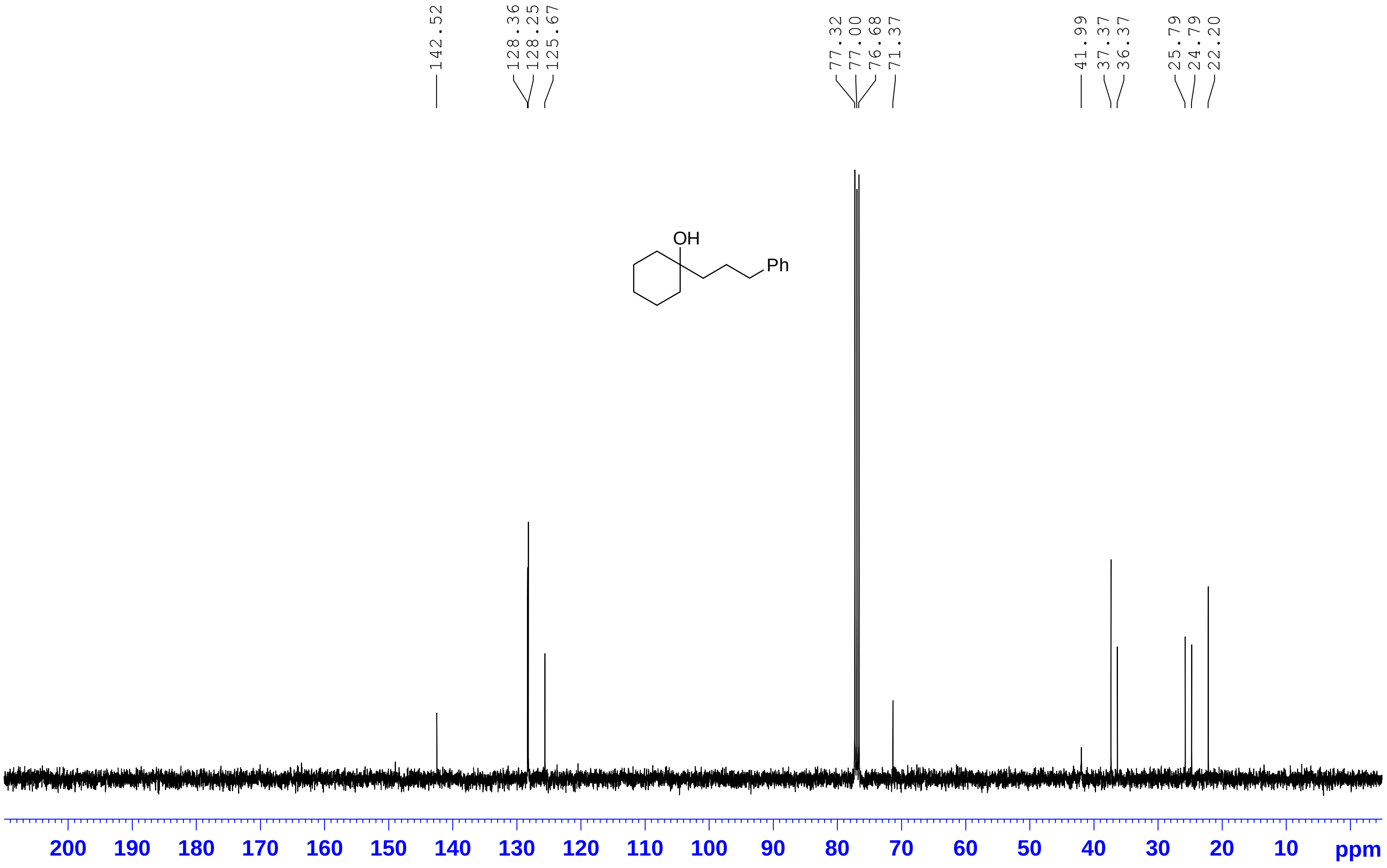

4. ^{1}H and ^{13}C NMR spectrum of new compounds

^{1}H NMR spectrum of **5n** (400 MHz, $CDCl_3$)

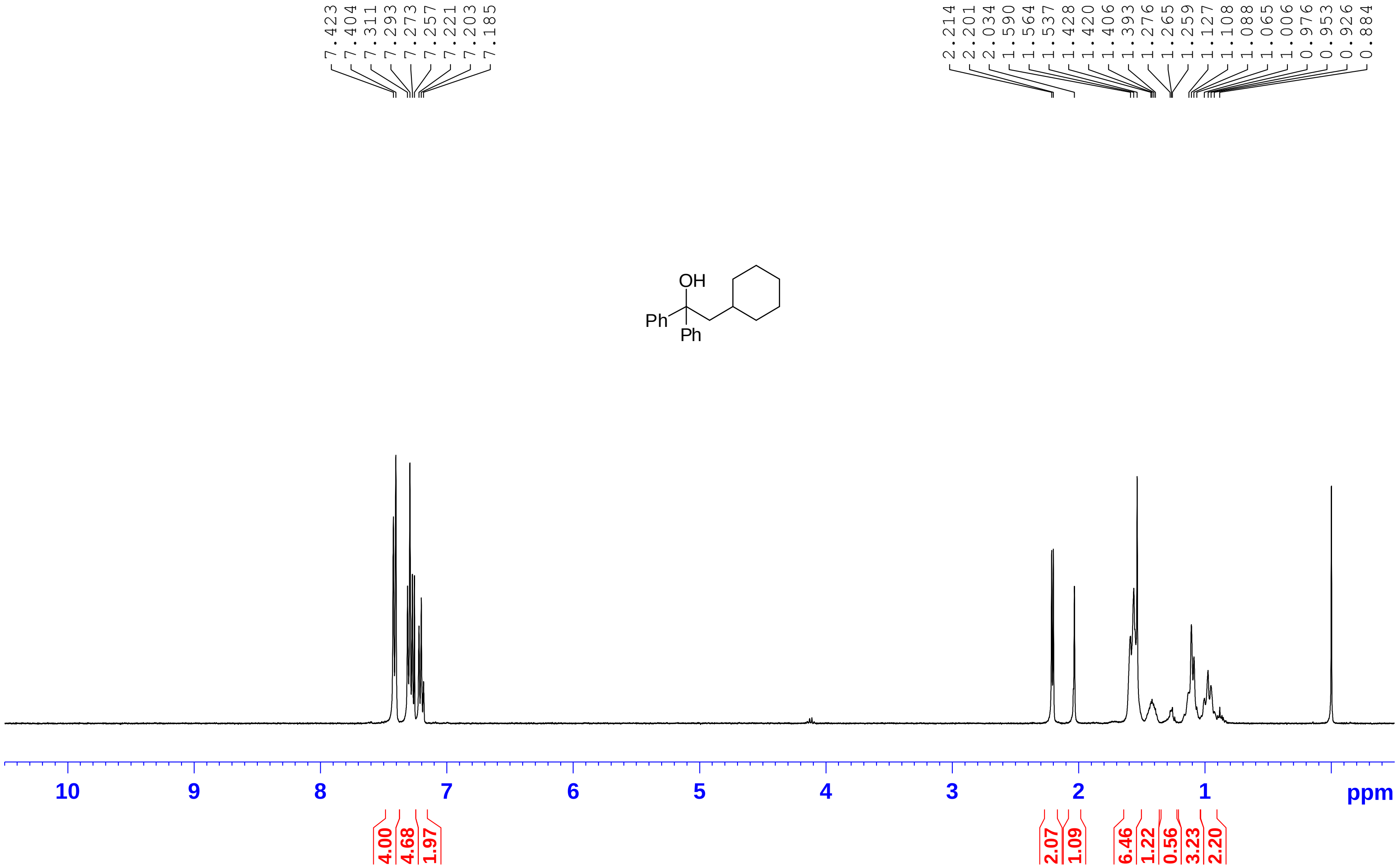

4. ^{1}H and ^{13}C NMR spectrum of new compounds

^{13}C NMR spectrum of **5n** (100 MHz, $CDCl_3$)

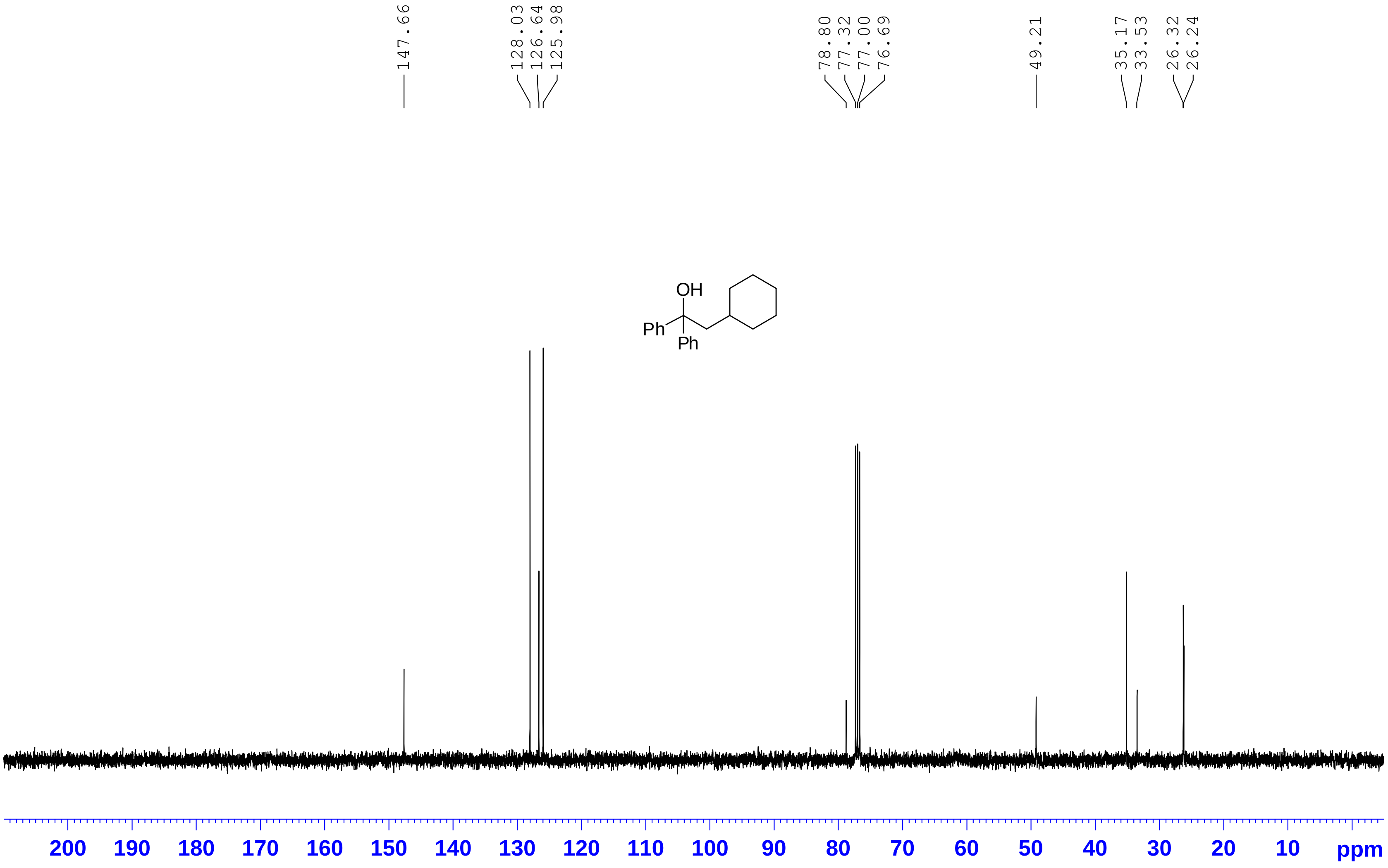

4. ^{1}H and ^{13}C NMR spectrum of new compounds

^{1}H NMR spectrum of **5o** (400 MHz, $CDCl_3$

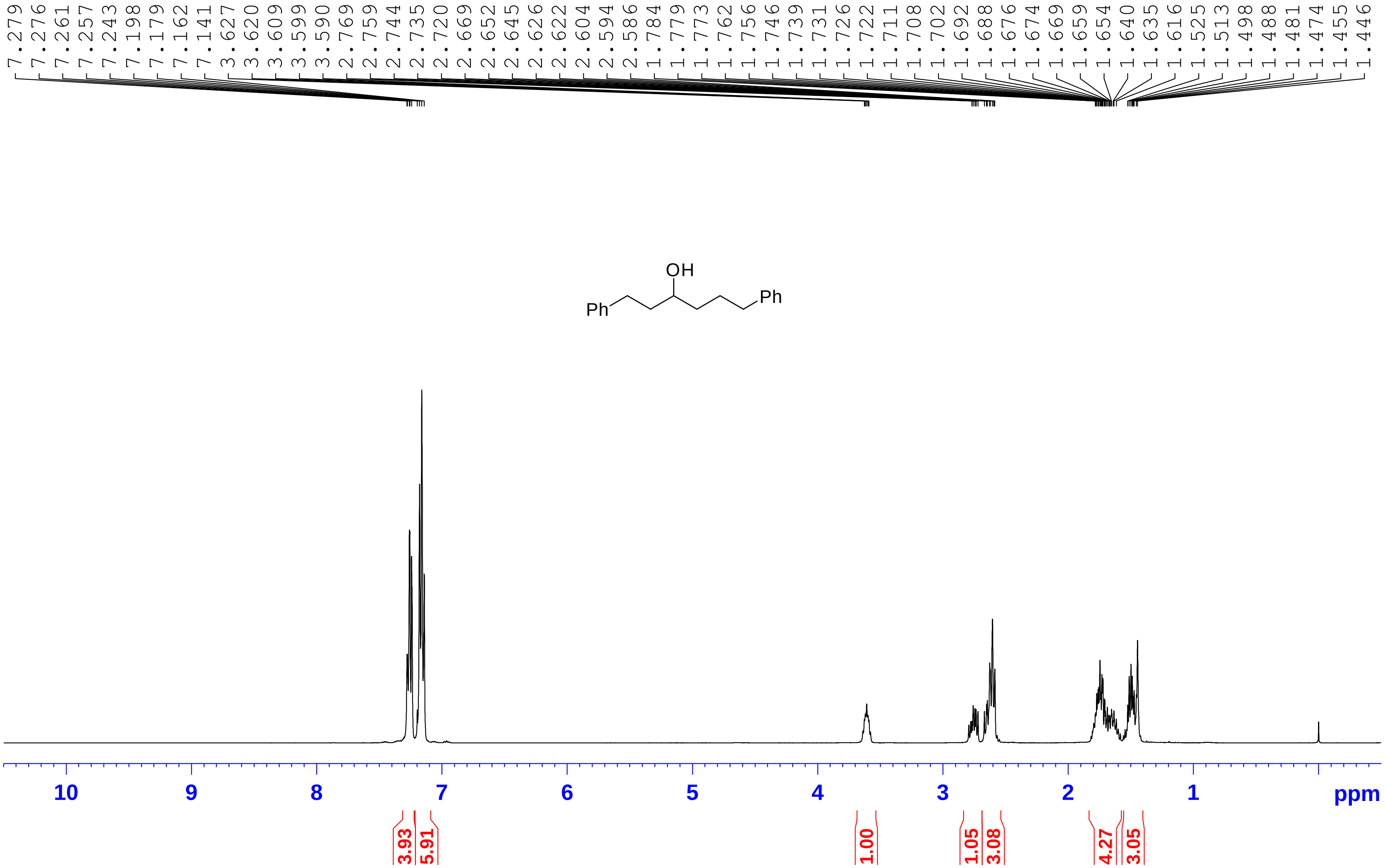

4. ^{1}H and ^{13}C NMR spectrum of new compounds

^{13}C NMR spectrum of **5o** (100 MHz, $CDCl_3$)

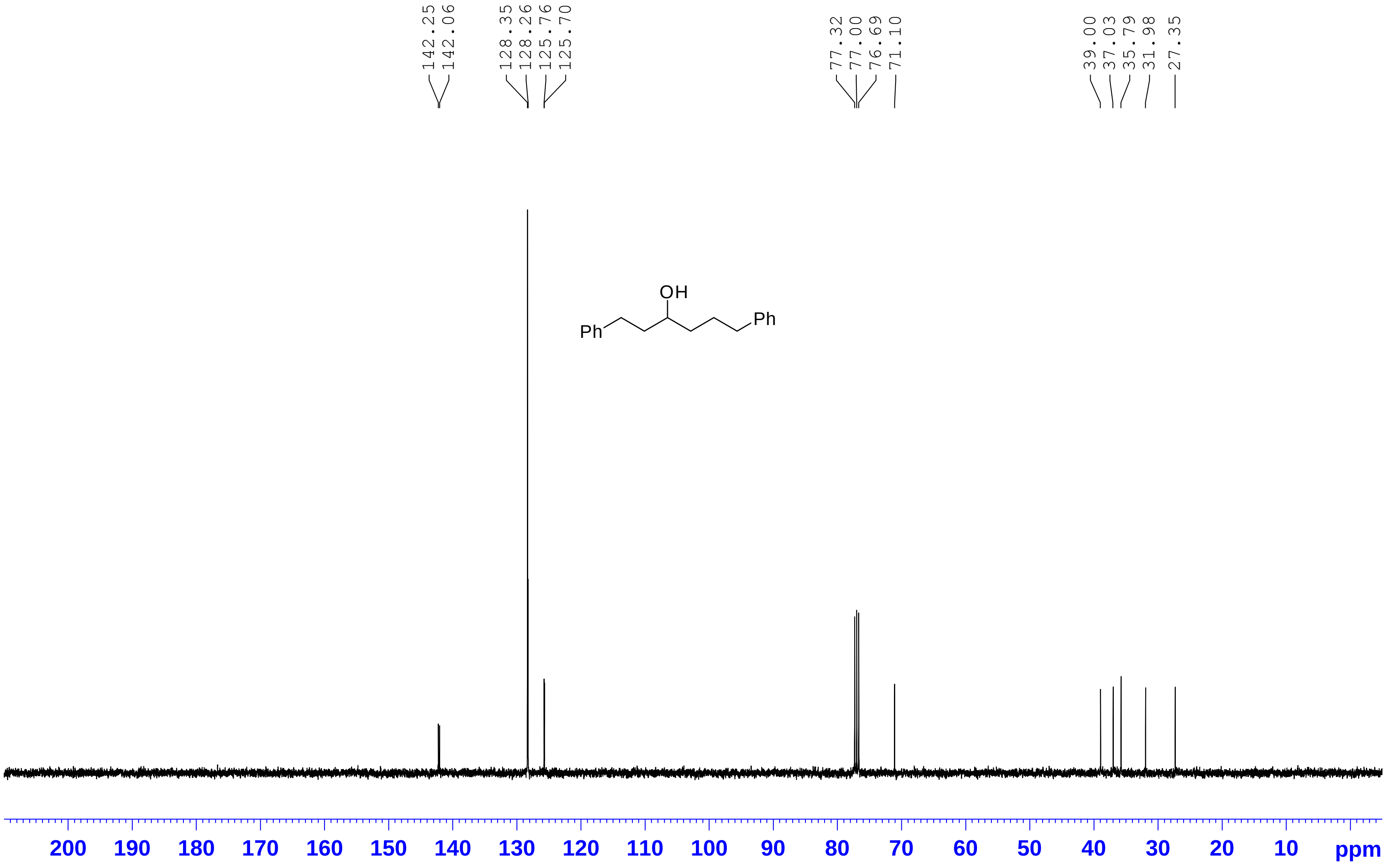

4. ¹H and ¹³C NMR spectrum of new compounds

¹H NMR spectrum of **5p** (400 MHz, $CDCl_3$)

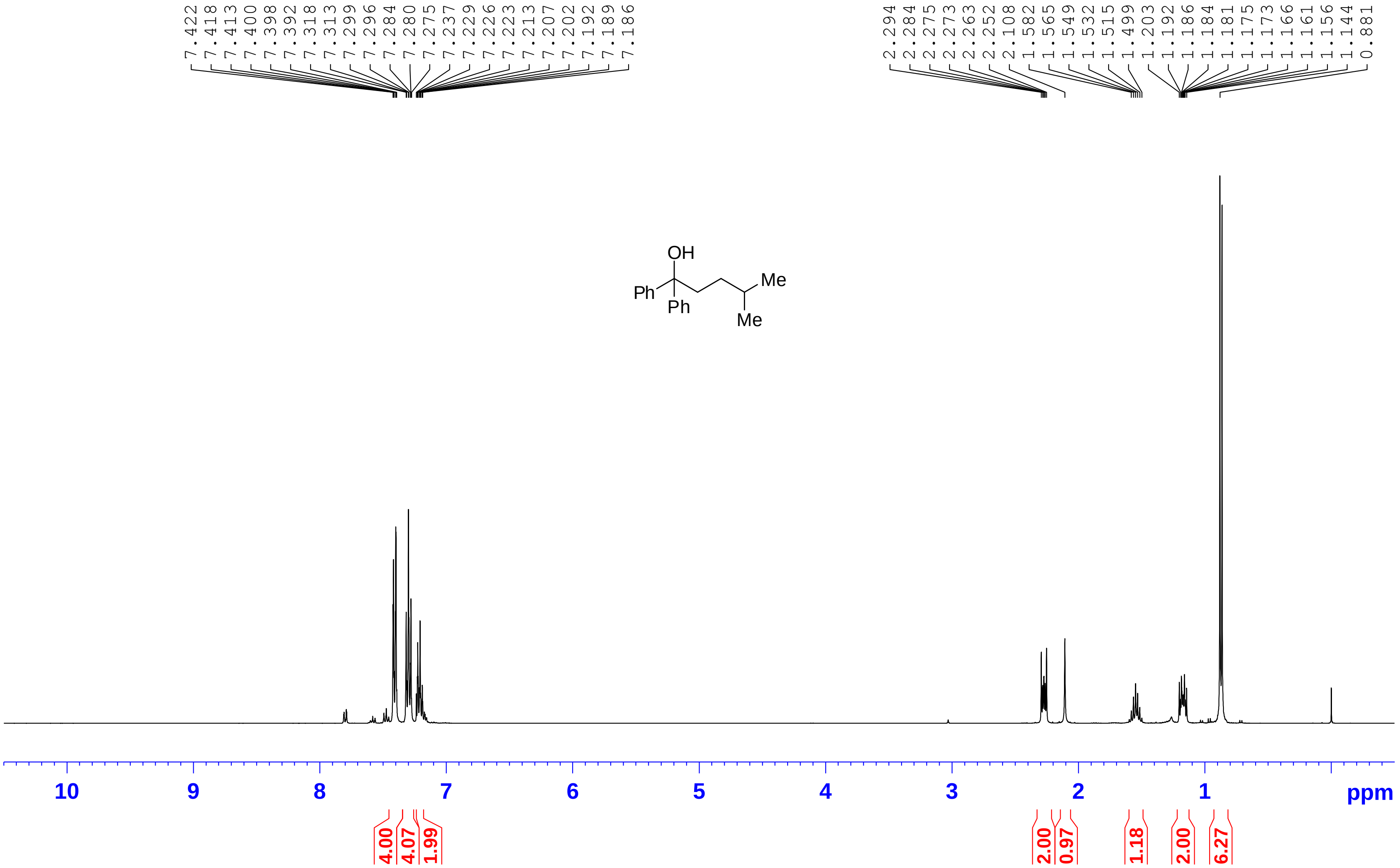

4. ^{1}H and ^{13}C NMR spectrum of new compounds

^{13}C NMR spectrum of **5p** (100 MHz, $CDCl_3$)

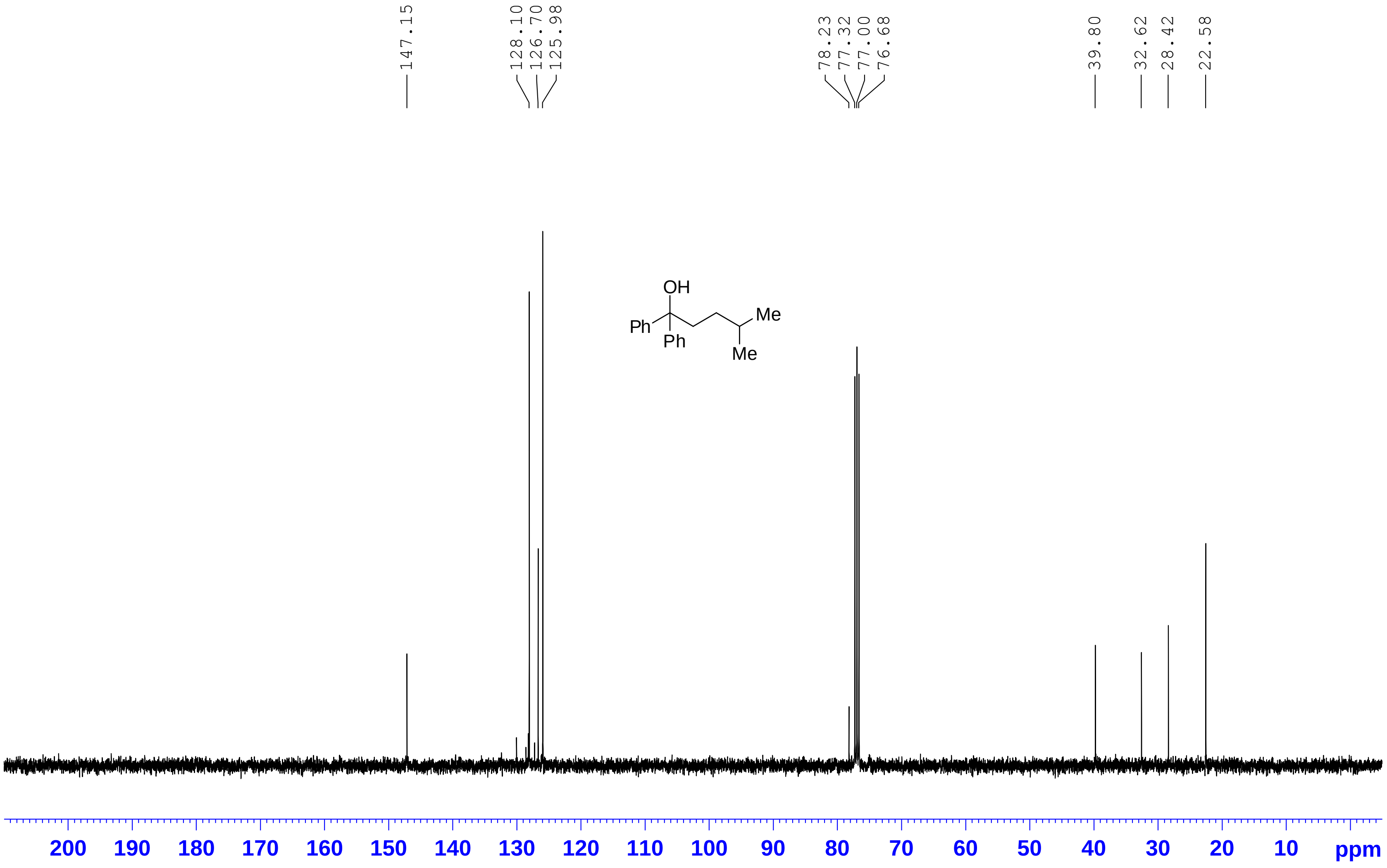

4. ^{1}H and ^{13}C NMR spectrum of new compounds

^{1}H NMR spectrum of **5r** (400 MHz, $CDCl_3$)

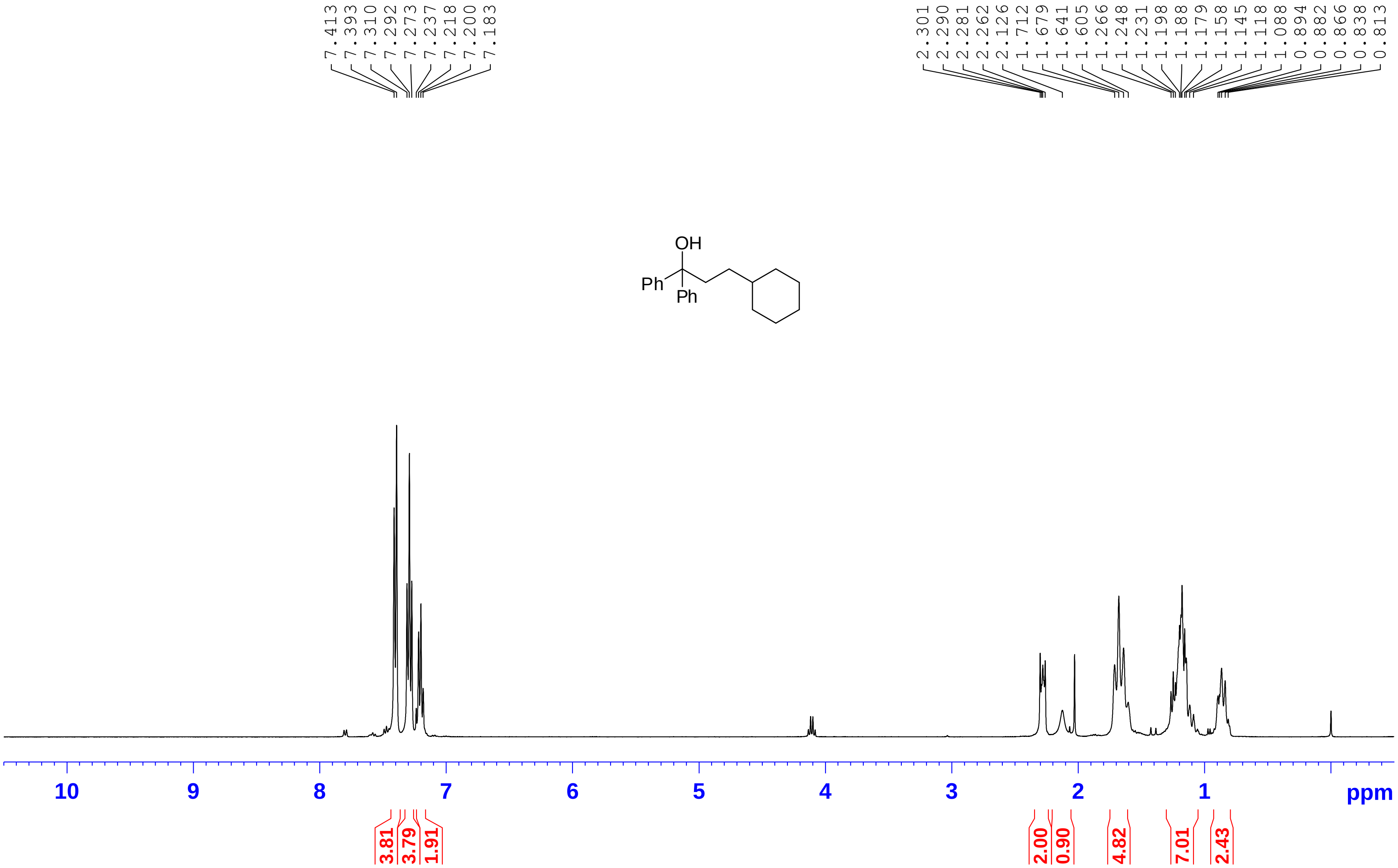

4. ^{1}H and ^{13}C NMR spectrum of new compounds

^{13}C NMR spectrum of **5r** (100 MHz, $CDCl_3$)

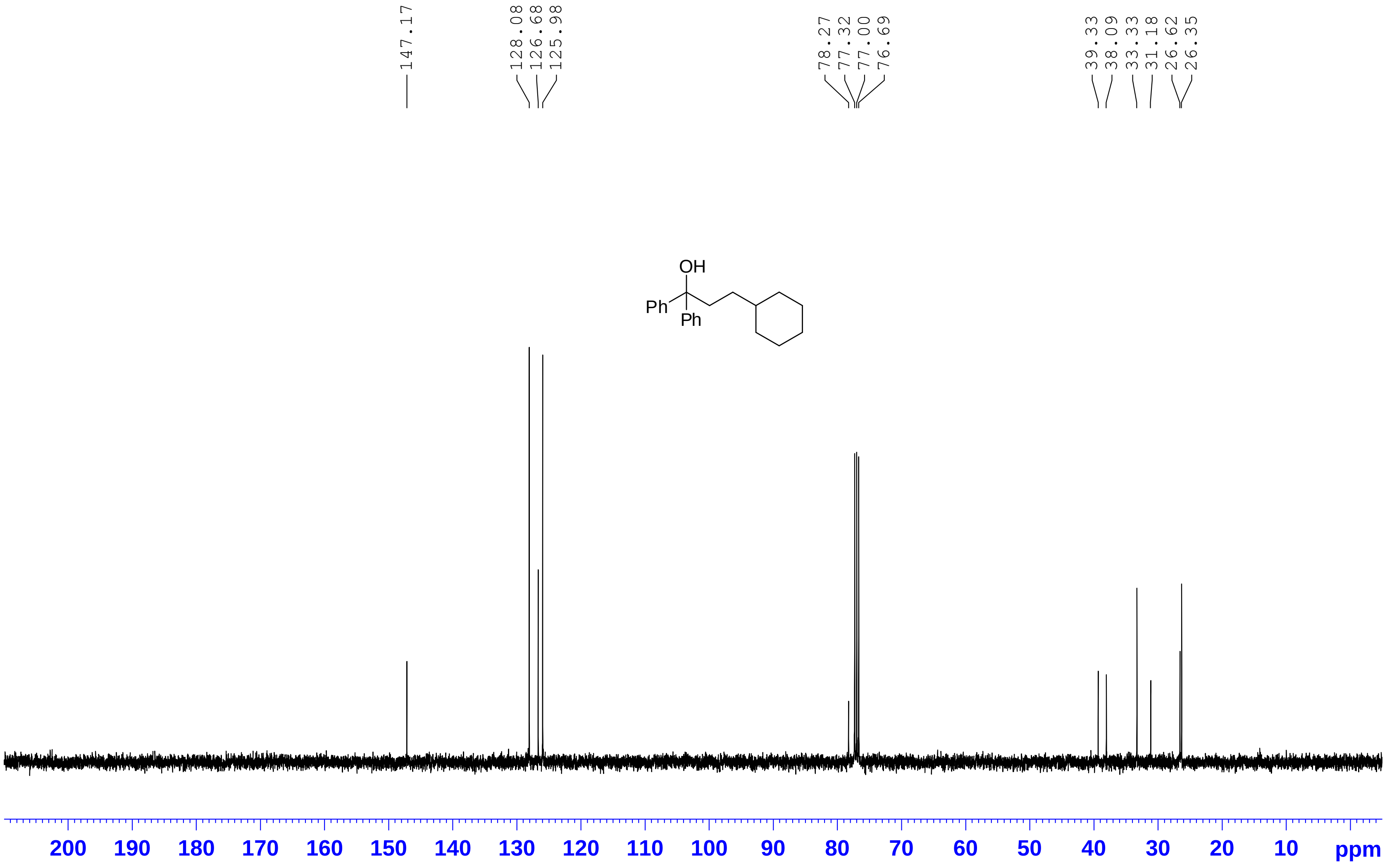

4. ^{1}H and ^{13}C NMR spectrum of new compounds

^{1}H NMR spectrum of **5s** (400 MHz, $CDCl_3$)

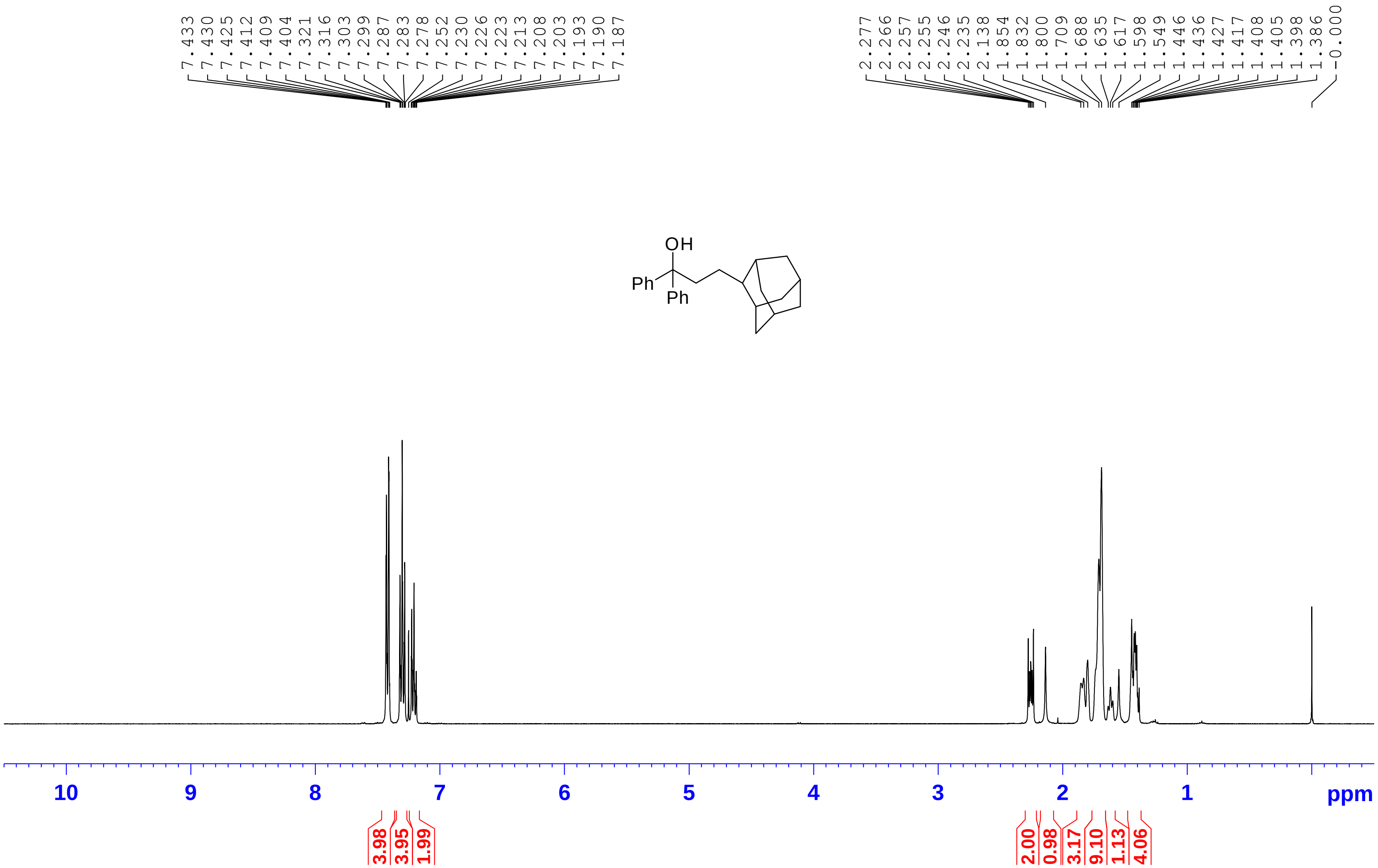

4. ^{1}H and ^{13}C NMR spectrum of new compounds

^{13}C NMR spectrum of **5s** (100 MHz, $CDCl_3$)

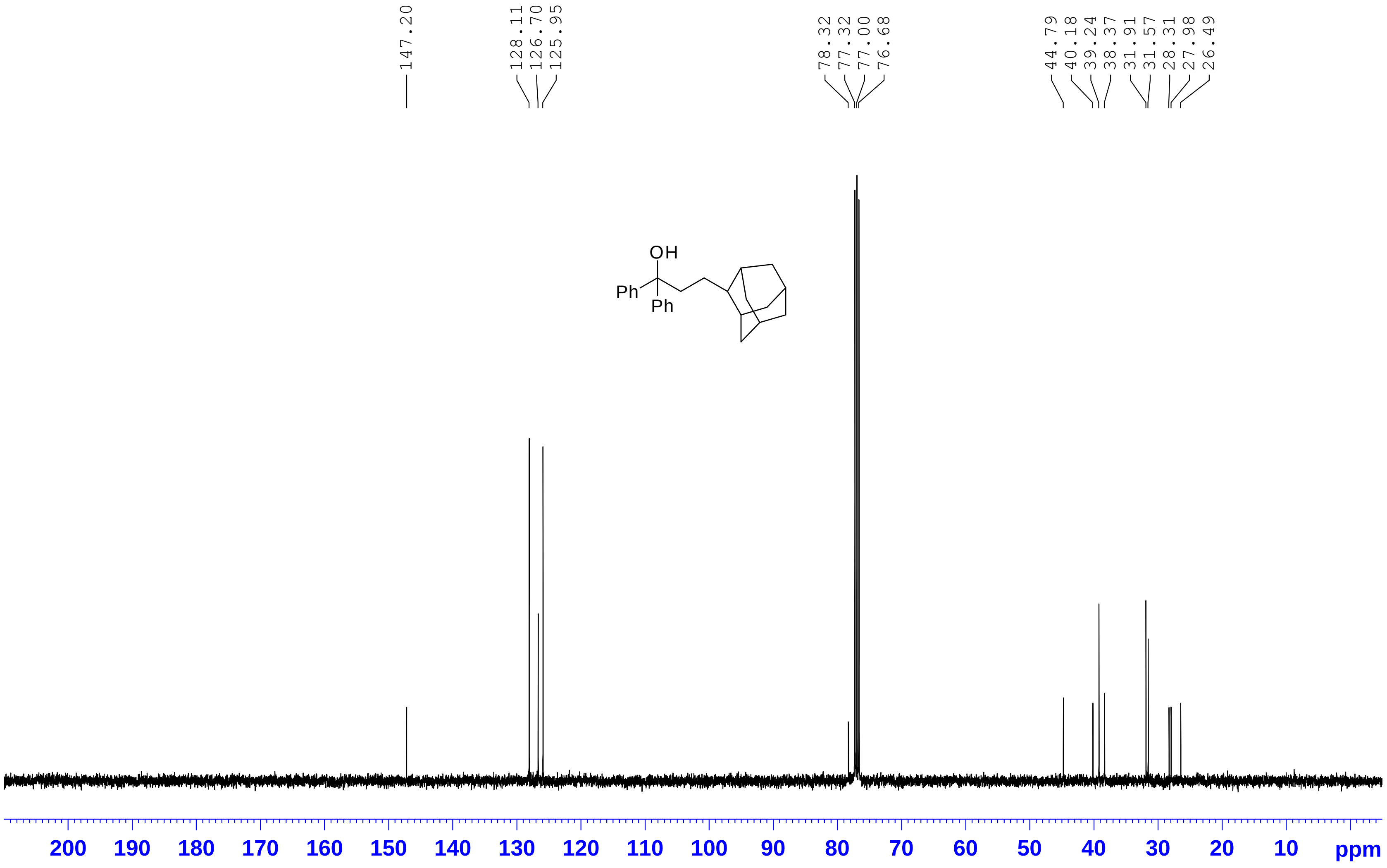

4. ¹H and ¹³C NMR spectrum of new compounds

¹H NMR spectrum of **8o** (400 MHz, $CDCl_3$)

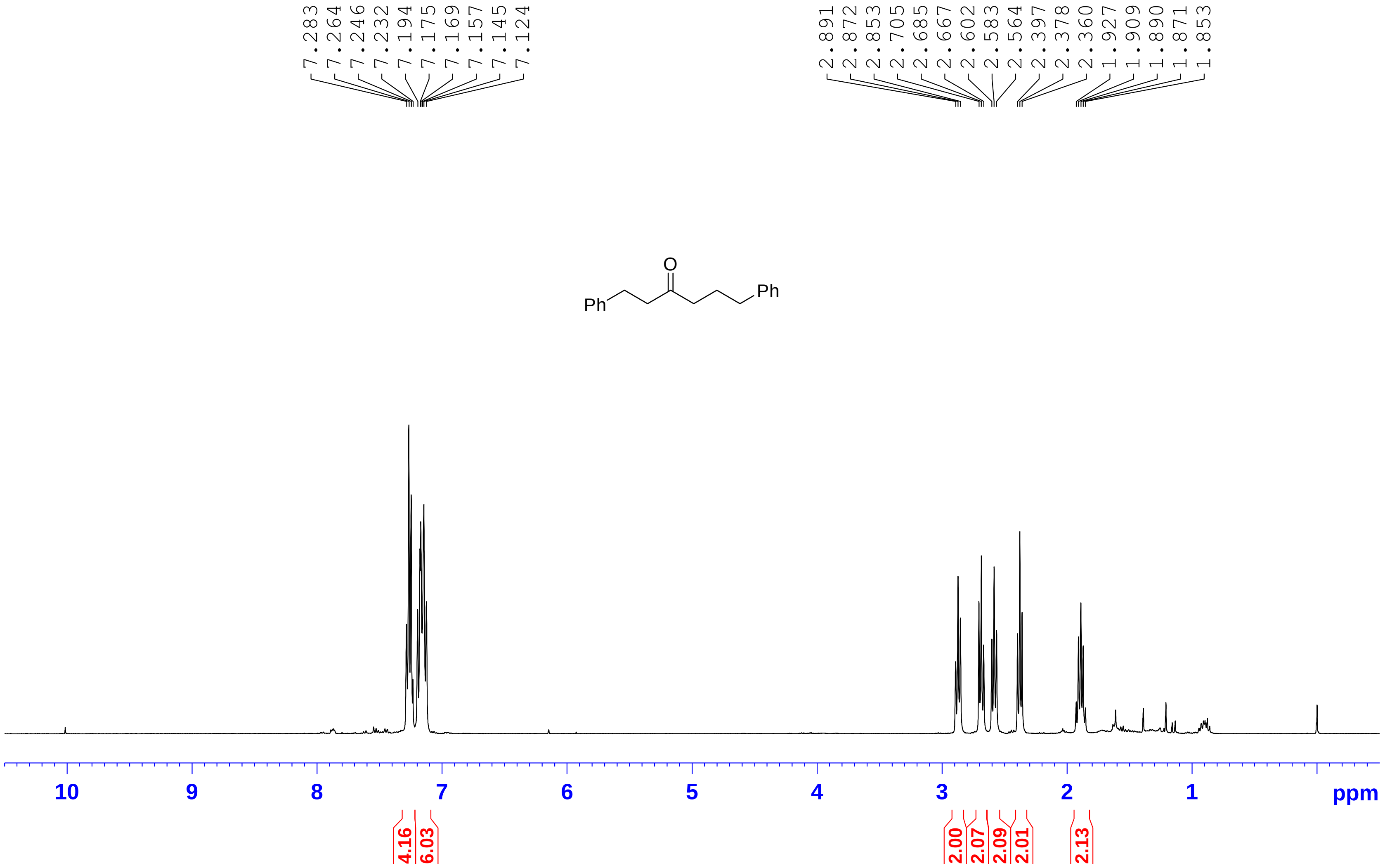

4. ^{1}H and ^{13}C NMR spectrum of new compounds

^{13}C NMR spectrum of **8o** (100 MHz, $CDCl_3$)

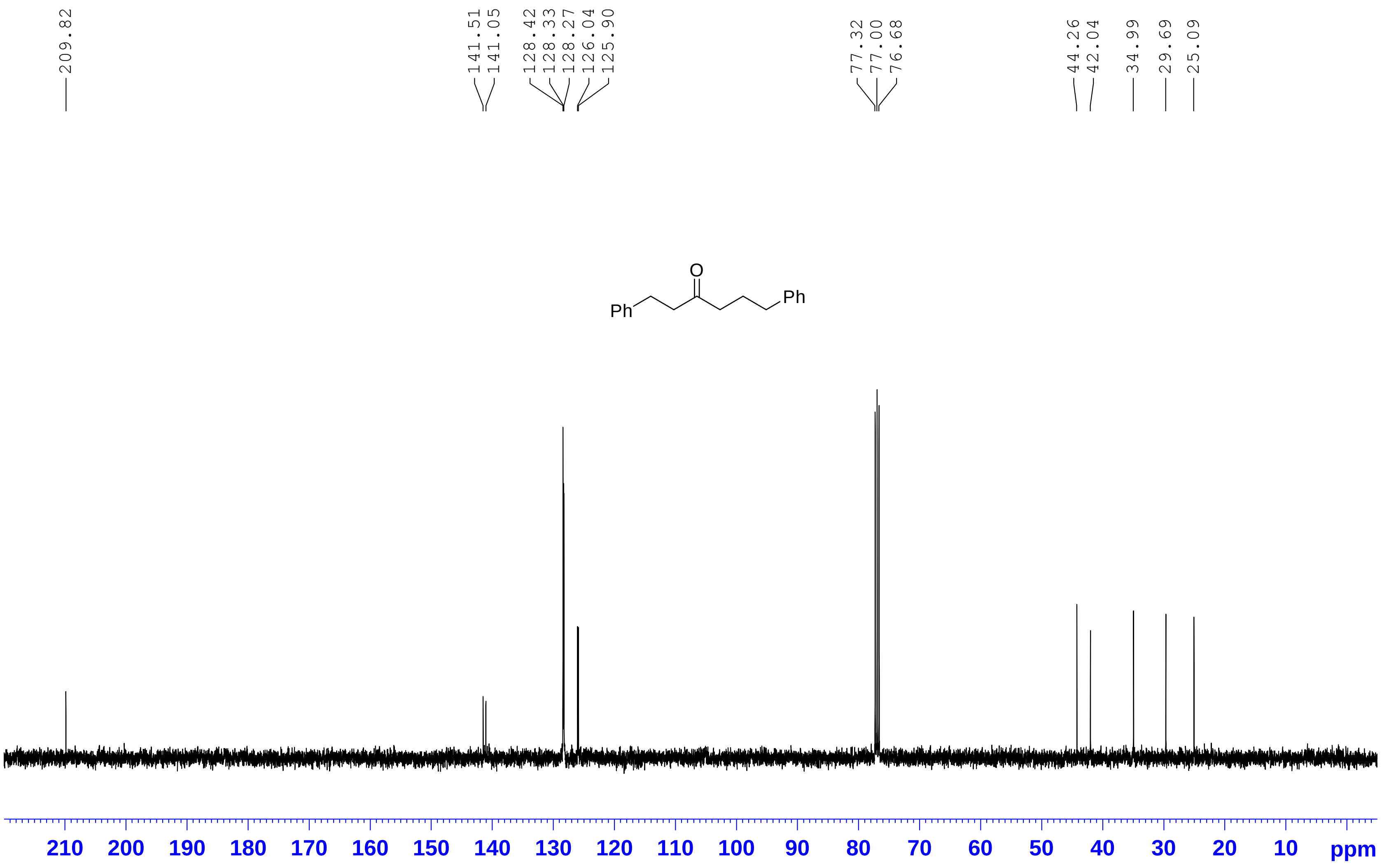

4. ^{1}H and ^{13}C NMR spectrum of new compounds

^{1}H NMR spectrum of **10** (400 MHz, $CDCl_3$)

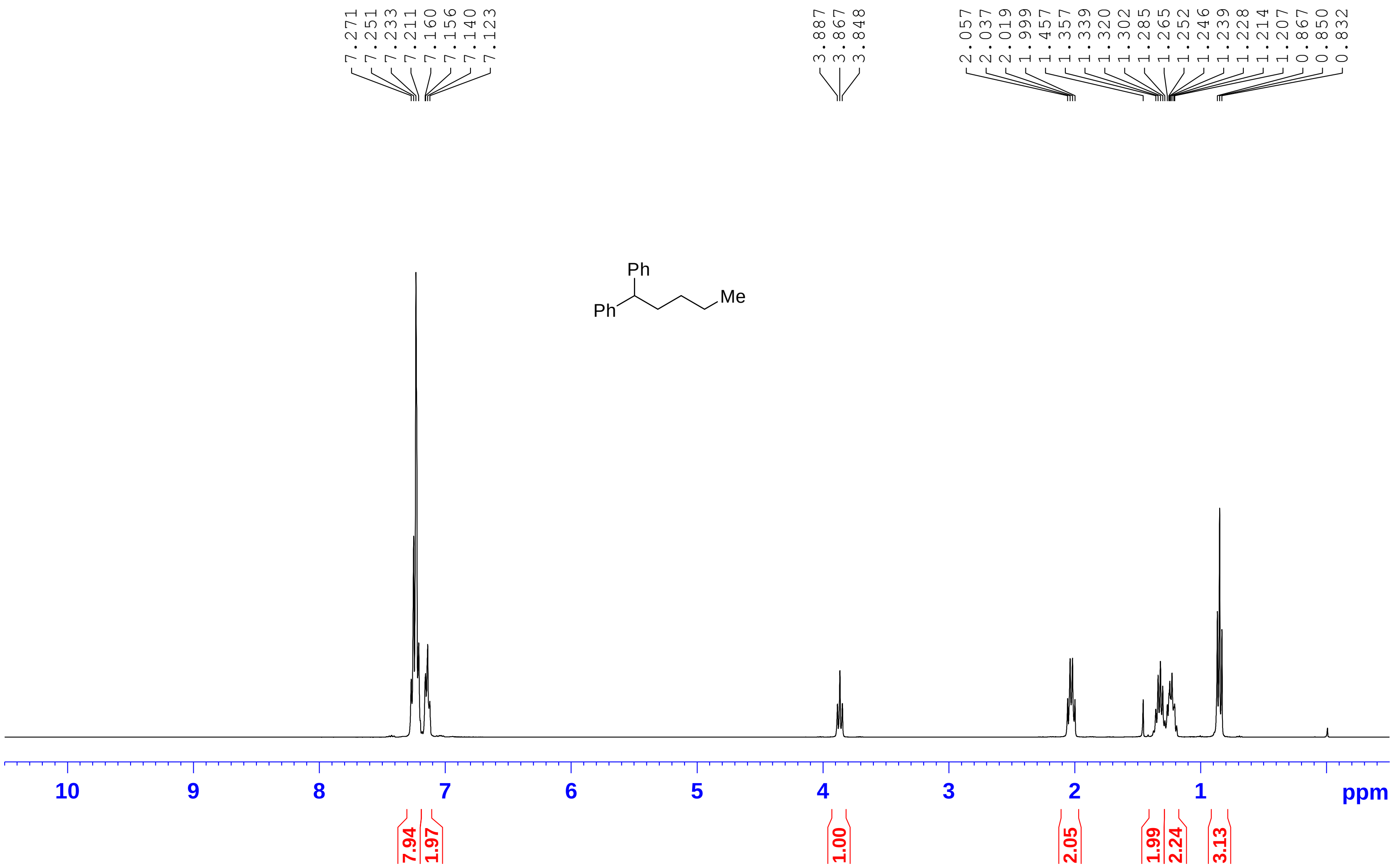

4. ^{1}H and ^{13}C NMR spectrum of new compounds

^{13}C NMR spectrum of **10** (100 MHz, $CDCl_3$)

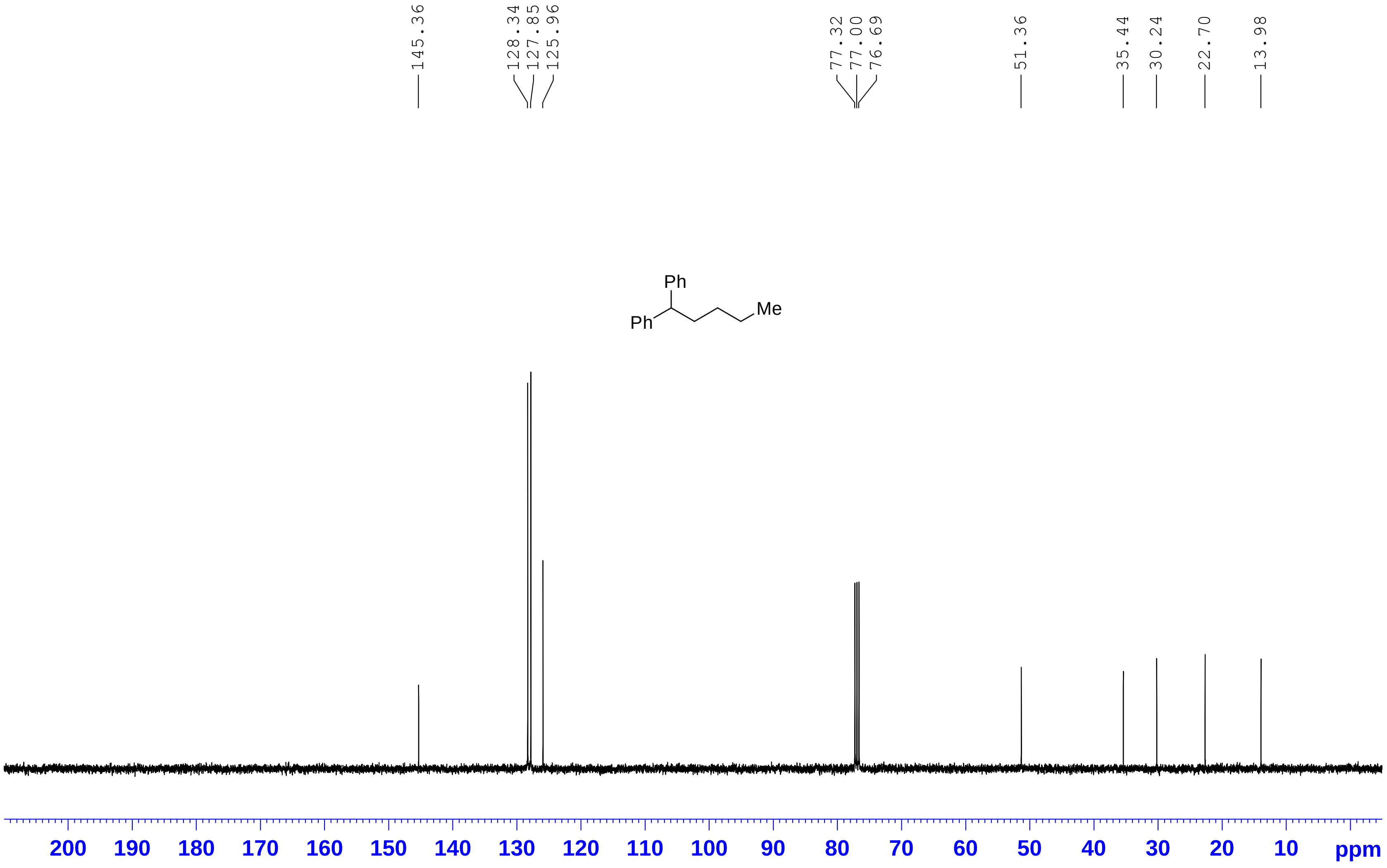

4. ^{1}H and ^{13}C NMR spectrum of new compounds

^{1}H NMR spectrum of **3-(adamantan-2-yl)-1-phenylpropan-1-one** (400 MHz, $CDCl_3$)

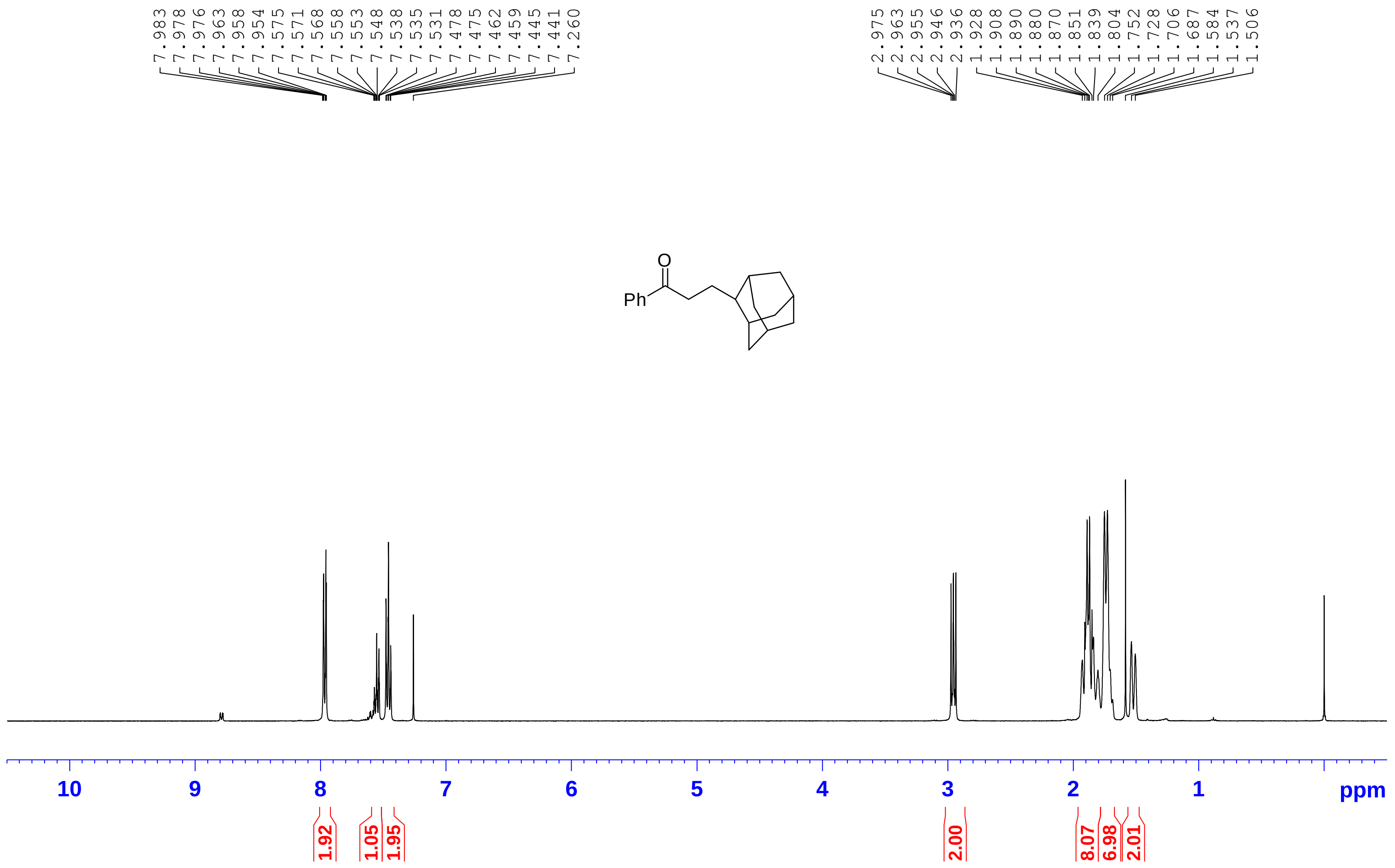

4. ^{1}H and ^{13}C NMR spectrum of new compounds

^{13}C NMR spectrum of **3-(adamantan-2-yl)-1-phenylpropan-1-one** (100 MHz, $CDCl_3$)

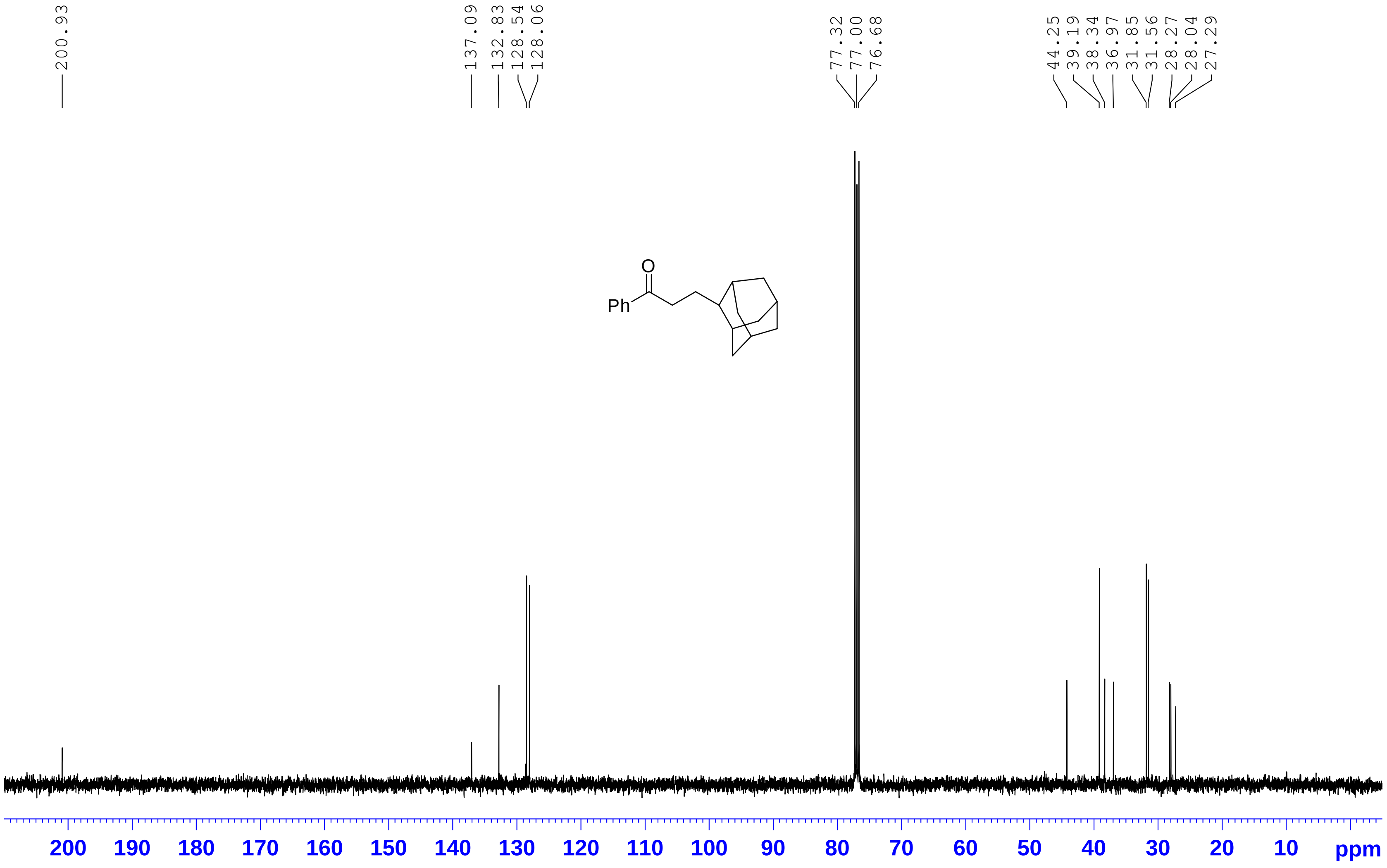

Supplement: File 1 — Full experimental details and analytical data. [file Beilstein_J_Org_Chem-09-1217-s001.pdf]
